# Diester Prodrugs of a Phosphonate Butyrophilin Ligand Display Improved Cell Potency, Plasma Stability, and Payload Internalization

**DOI:** 10.1021/acs.jmedchem.3c01358

**Published:** 2023-11-07

**Authors:** Umed Singh, Girija Pawge, Sarita Rani, Chia-Hung Christine Hsiao, Andrew J. Wiemer, David F. Wiemer

**Affiliations:** † Department of Chemistry, University of Iowa, Iowa City, Iowa 52242-1294, United States; ‡ Department of Pharmaceutical Sciences, University of Connecticut, Storrs, Connecticut 06269-3092, United States; § Institute for Systems Genomics, University of Connecticut, Storrs, Connecticut 06269-3092, United States; ∥ Department of Pharmacology, University of Iowa, Iowa City, Iowa 52242-1109, United States

## Abstract

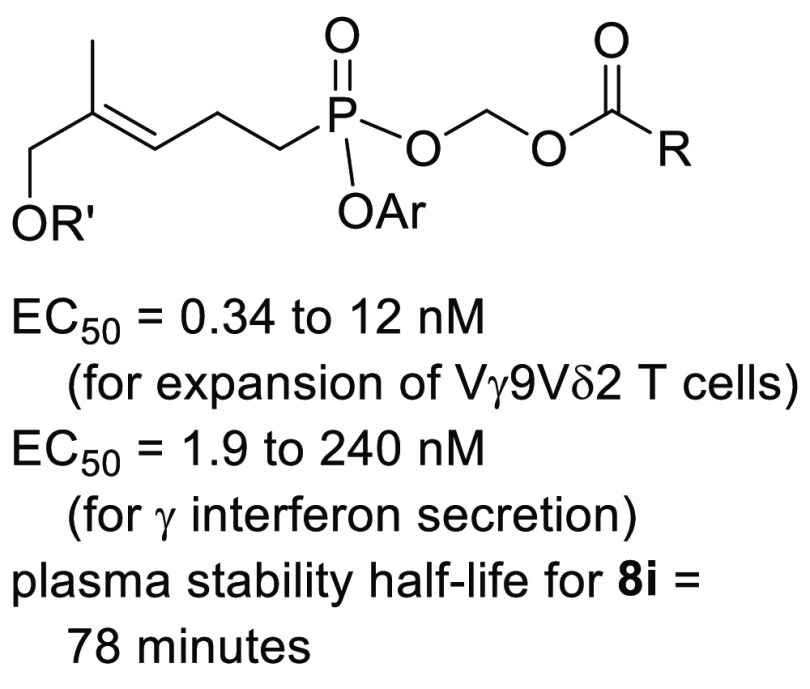

Activation of Vγ9Vδ2 T cells with butyrophilin 3A1 (BTN3A1) agonists such as (*E*)-4-hydroxy-3-methyl-but-2-enyl diphosphate (HMBPP) has the potential to boost the immune response. Because HMBPP is highly charged and metabolically unstable, prodrugs may be needed to overcome these liabilities, but the prodrugs themselves may be limited by slow payload release or low plasma stability. To identify effective prodrug forms of a phosphonate agonist of BTN3A1, we have prepared a set of diesters bearing one aryl and one acyloxymethyl group. The compounds were evaluated for their ability to stimulate Vγ9Vδ2 T cell proliferation, increase production of interferon γ, resist plasma metabolism, and internalize into leukemia cells. These bioassays have revealed that varied aryl and acyloxymethyl groups can decouple plasma and cellular metabolism and have a significant impact on bioactivity (>200-fold range) and stability (>10 fold range), including some with subnanomolar potency. Our findings increase the understanding of the structure–activity relationships of mixed aryl/acyloxymethyl phosphonate prodrugs.

## Introduction

**Figure 1:**
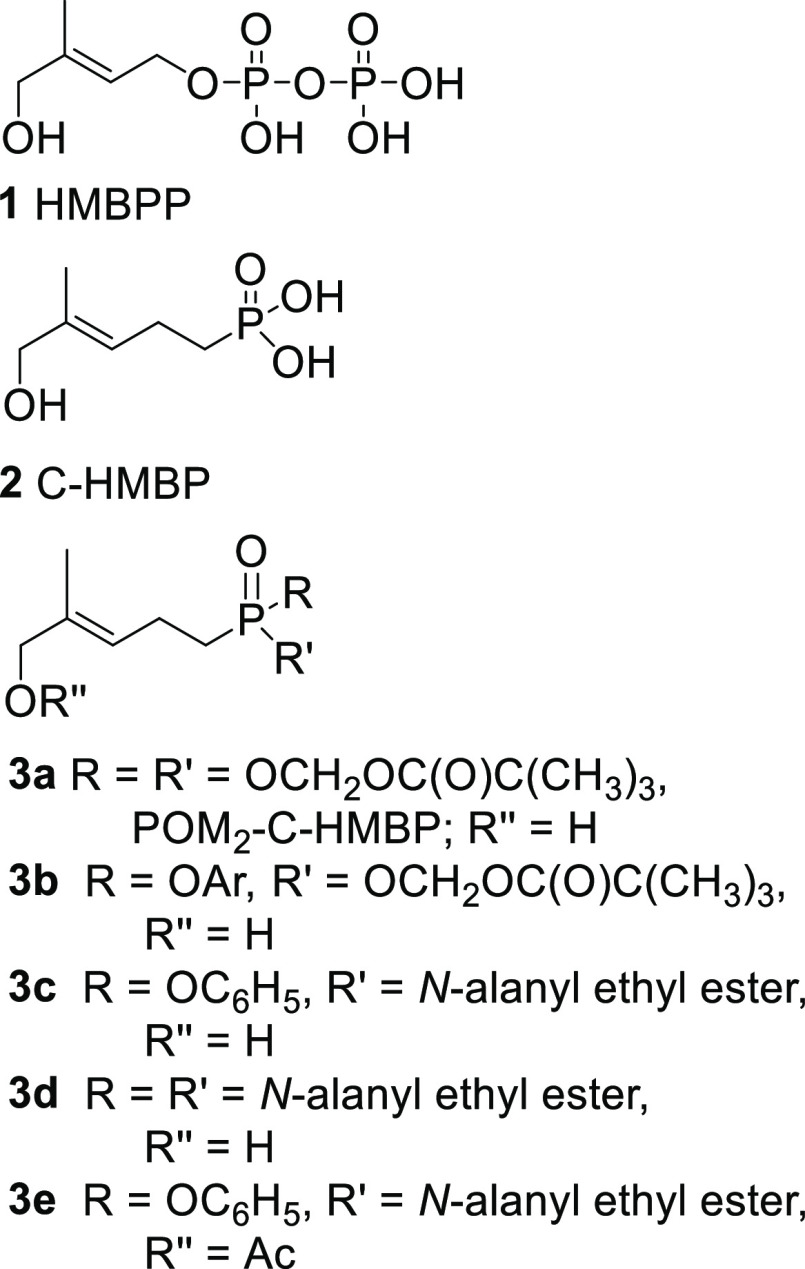
Natural phosphoantigen (HMBPP, **1**), a phosphonate analog (**2**), and selected phosphonate prodrugs (**3a**–**3e**).

The human immune system presents a multifaceted defense against microbial infections and malignancies. Arguably, the best known facet of the human adaptive immune system would be the αβ T cells, which use their T cell receptors (TCRs) to recognize foreign peptides with the assistance of the major histocompatibility complex (MHC).^1^ A T cell population of lesser abundance, which is more primitive in an evolutionary sense, employs Vγ9Vδ2 TCRs to recognize a limited set of small organophosphorus compounds.^2^ For example, the isoprenoid (*E*)-4-hydroxy-3-methyl-but-2-enyl diphosphate (HMBPP, **1**, Figure 1) is a highly potent stimulant of Vγ9Vδ2 T cell proliferation, and it has become the centerpiece of a family of compounds known collectively as phosphoantigens (pAgs).^3^ Small molecule diphosphates are critical to the growth and survival of bacteria.^4^ The diphosphate HMBPP, which is the last intermediate in isoprenoid biosynthesis in bacteria that is not found in human metabolism, serves as an effective prompt for the human immune system to respond to intracellular bacterial infections. While Vγ9Vδ2 T cells may have evolved to fight bacterial infections, this system has remained relatively unexplored; its therapeutic manipulation may have the potential to address other challenges to human health including malignancies.

While the mechanisms that lead to Vγ9Vδ2 T cell proliferation after exposure to pAgs are not yet completely understood,^2,5−11^ pAg binding to the transmembrane protein butyrophilin 3A1 (BTN3A1) is essential.^12^ Perhaps surprisingly, binding occurs within the cell at the B30.2 domain of BTN3A1 rather than on the exterior surface of the cell.^13−15^ HMBPP binding to the internal domain of BTN3 dimers^16,17^ promotes their interaction with the internal domain of a BTN2A1 homodimer,^18,19^ which precedes extracellular detection of the tetrameric complex (also known as the HMBPP receptor) by the Vγ9Vδ2 TCR.^20^ Thus, effective ligands for this protein must be able to transit the cell membrane, which is best accomplished by lipophilic molecules with little or no charge.^21,22^ Furthermore, diphosphates such as HMBPP have little or no serum stability,^23^ undergoing hydrolysis so rapidly that they are not attractive drug candidates.

To address this second factor, numerous studies have employed phosphonates such as compound **2** (C-HMBP), where isosteric replacement of the phosphate ester oxygen with carbon results in compounds with much greater metabolic stability. While phosphonate **2** itself serves as a pAg, it has only moderate cellular potency as its charge constitutes a barrier to cell entry. Significantly improved potency can be obtained through use of neutral phosphonate derivatives if they function as prodrugs. Many strategies have been developed to enhance the membrane permeability of phosphates and phosphonates,^21,22^ but until our initial report on compound **3a** (POM_2_ C-HMBP), no one had explored a prodrug strategy with BTN3A1 ligands.^15^ The prodrug **3a** displays a potency about 740-fold greater than the sodium salt of phosphonate **2**.

After our first report on compound **3a,** a number of other prodrug forms have been explored, including mixed aryl acyloxy diesters (**3b**),^24,25^ aryl phosphonamidates (e.g., **3c**),^26,27^ and bisamidates (e.g., **3d**),^28^ as well as double prodrugs that included acetate protection of the allylic alcohol (e.g., **3e**).^29^ While several of these compounds have cellular potency in the low nanomolar or even high picomolar range, for improved in vivo capabilities the ideal prodrug form would have fast cellular uptake and high serum stability and yield benign fragments upon drug release.

Of the prodrug forms that we have studied, the dimethyl ester of the parent phosphonate **2** was found to have high plasma stability but little or no potency, perhaps because it is too stable to metabolism. The bis-POM compound **3a** has good potency but rapidly undergoes hydrolysis in plasma. As a group, the aryl amidates **3c** have activity in the low nanomolar range upon 72 h exposure time, but that potency drops significantly when exposure time is restricted.^30^ Furthermore, the stereogenic center within the alanyl unit together with the stereogenic center at phosphorus renders the initially prepared compounds mixtures of diastereomers, which may complicate determination of the kinetics of drug release. The mixed esters (**3b**) that include one aryl group and one acyloxy group have attractive potency and fast target engagement in cell assays. They may have real potential for in vivo use if esterase-mediated hydrolysis by intracellular enzymes is faster than that mediated by esterases found in plasma, assuming high permeability of the compounds as a prerequisite. In addition, the mixed esters also do not necessarily include stereocenters, other than at phosphorus. With this in mind, we initiated a new study on mixed aryl/acyloxymethyl esters with the goal of improving potency and/or plasma stability of compounds such as **3b**,^25^ the current leads in this series.

## Results

### Synthesis of New Aryl Analogs of Compound **8a**

**Scheme 1:**
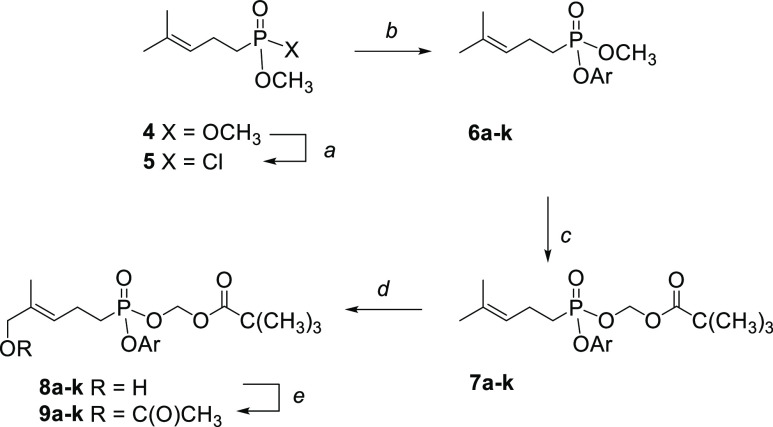
Synthesis of Mixed Aryl Pivaloyloxymethyl Phosphonate Esters Reagents and conditions: (a) (COCl)_2_, CH_2_Cl_2_ (anhyd), DMF (cat.), 0 °C to rt; (b) ArOH, Et_3_N (anhyd), 0 °C to rt, 24 to 48 h, 60–85% (over two steps); (c) POMCl, NaI, CH_3_CN (anhyd), 80 °C, 24 to 48 h, 30–45%; (d) SeO_2_, *t*-BuOOH, 4-hydroxybenzoic acid, CH_2_Cl_2_, 0 °C, 3–4 days, 5–45%; for compounds **8c**, **8d**, **8g**, and **8k**, workup included treatment with NaBH_4_ to reduce an aldehyde formed by overoxidation. (e) Ac_2_O, Et_3_N, rt, overnight, 90–98%.

Synthesis of this family of new phosphonates employed a reaction sequence from the known dimethyl homoprenylphosphonate **4** (Scheme 1).^15,31^ After conversion of the dimethyl ester to the acid chloride **5** through reaction with oxalyl chloride and DMF and minimal purification, treatment with a series of phenols gave the desired mixed esters **6a**–**k**. Because the methyl ester is significantly more reactive than the aryl ester, subsequent reaction with pivaloyloxymethyl chloride (POMCl)^32^ in the presence of sodium iodide gave the desired mixed diesters **7a**–**k** in modest yields. Final oxidation with catalytic selenium dioxide and *t*-butyl hydroperoxide introduced the allylic alcohol in low yield but with complete selectivity for formation of the necessary *E*-allylic alcohols **8a**–**k**.^33^ In several cases (compounds **8c**, **8d**, **8g**, and **8k**), treatment with NaBH_4_ was included as part of the workup after TLC analysis suggested overoxidation of the allylic alcohol to the corresponding aldehyde. No attempt was made to control the absolute stereochemistry at phosphorus: on the one hand, hydrolysis delivers a ligand with no phosphorus stereochemistry, and on the other hand, if intriguing biological activity was observed, separation of the stereoisomers could be pursued at a later stage of these studies.

After preparation of the known mixed esters derived from phenol (**7a**) and 1-naphthol (**7b**) to serve as positive controls,^25^ nine new aryl esters were prepared (**7c**–**k**, Table 1). Reaction of the acid chloride **5** with methyl salicylate and *p*-acetamidophenol ultimately led to the mixed aryl/acyloxy esters **8c** and **8d**, while reaction with a protected tyrosine derivative gave the mixed ester **8e**. Because substituents at the para position of the aromatic ring appeared to be well-tolerated, we proceeded to prepare the corresponding phosphonates from *p*-cresol (**8f**), its *p*-trifluoromethyl analogue (**8g**), and *p*-isopropylphenol (**8i**). The methyl substituent in compound **8f** should be modestly electron-donating, have a minimal steric demand, and provide a potential site for ultimate metabolic degradation. In contrast, the *p*-trifluoromethyl compound **8g** bears a strong electron-withdrawing group with a steric requirement more like an isopropyl or *sec*-butyl substituent.^34−36^ To mimic this steric requirement but avoid the stereogenic center of a *sec*-butyl group, we prepared the *p*-isopropyl compound **8i**. Finally, we prepared phosphonate derivatives from *o*-trifluoromethylphenol (**8h**) and *o*-isopropylphenol (**8j**) as well as 2,6-diisopropylphenol (**8k**), where parallel steric and electronic arguments can be made.

**Table 1:**
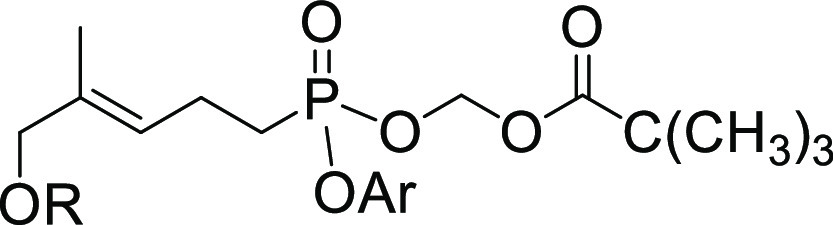

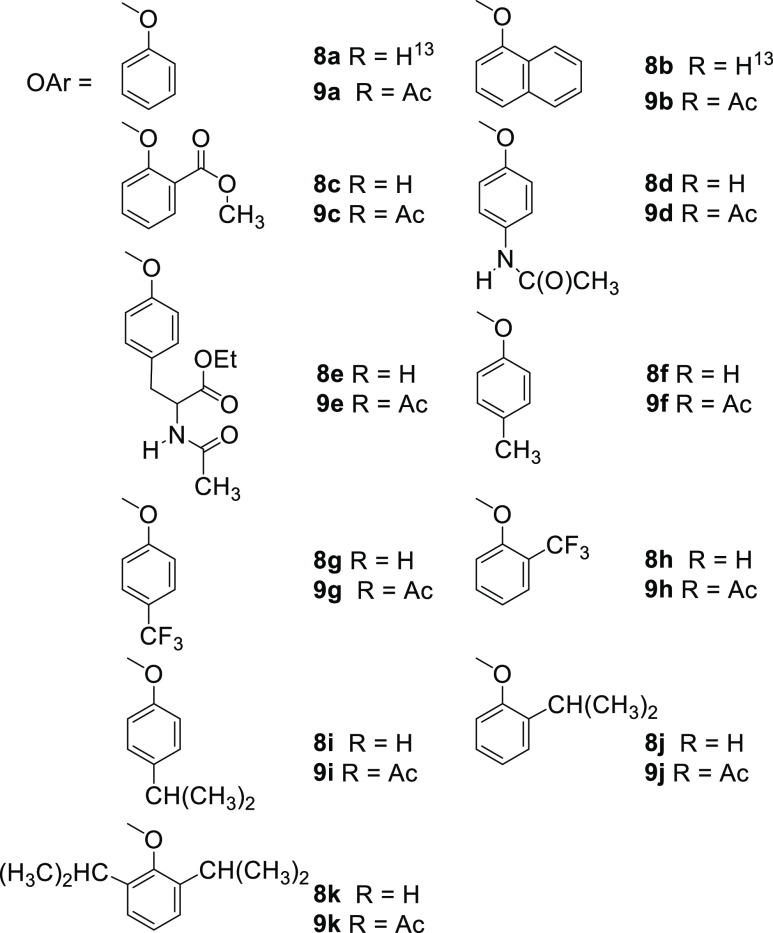
Mixed Aryl/Pivaloyloxymethyl Phosphonate Esters

Once the set of 10 new mixed aryl/pivaloyloxymethyl esters was in hand, each of the allylic alcohols **8a**–**k** was treated with acetic anhydride to obtain the new allylic acetates **9a**–**k**. While prior studies had suggested the allylic acetate might improve potency,^29^ the synthesis of this larger set of compounds in both alcohol and acetate form would allow for a definitive analysis of the impact of the allylic acetate on potency and metabolism.

### Synthesis of New Acyloxymethyl Analogs of Compound **8a**

**Scheme 2:**
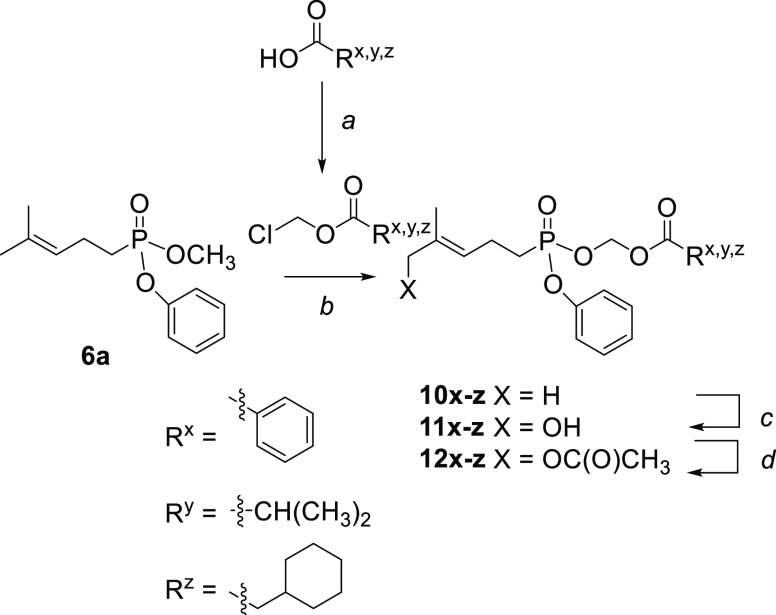
Synthesis of Mixed Phenyl Acyloxymethyl Phosphonate Esters Reagents and conditions: (a) (i) NaOH, MeOH; (ii) chloromethylchlorosulfate, NaHCO_3_, Bu_4_NHSO_4_, CH_2_Cl_2_/H_2_O 1:1; (b) R^x,y,z^COCH_2_OCl, NaI, CH_3_CN (anhyd), reflux, 24 to 48 h, 30–40%; (c) SeO_2_, 4-hydroxybenzoic acid, *t*-BuOOH, CH_2_Cl_2_, 0 °C, 3–4 days, 5–45%; (d) Ac_2_O, Et_3_N, rt, overnight, 90–98%.

In addition to the impact of the aryl group, the structure of the acyloxymethyl group also may have an impact on the rate of hydrolysis.^37^ Few groups have examined this position, with most opting to use POM and POC forms found in clinical phosphonate prodrugs. One key study reported a 40-fold variation in the rate of hydrolysis as a function of the acyl group incorporated in the acyloxymethyl ester.^38^ To initiate investigation of this factor, we selected acyl groups at the extremes of stability and lability from the work of Dickson et al.^38^ Thus, benzoic acid (R^x^), isobutyric acid (R^y^), and cyclohexylacetic acid (R^z^) were converted to their respective chloromethyl esters through reaction with chloromethyl chlorosulfate,^39,40^ and the resulting reagents were used to prepare the respective acyloxymethyl esters **10x**–**z** of phosphonate **6a** (Scheme 2). After installation of the acyloxymethyl group, oxidation with selenium dioxide was used to introduce the allylic alcohols (i.e., **11x**–**z**), and a final reaction with acetic anhydride gave the double prodrugs **12x**–**z**.

### Analogs of Compound **8a** Potently Stimulate Proliferation of Primary Human Vγ9Vδ2 T Cells

**Figure 2:**
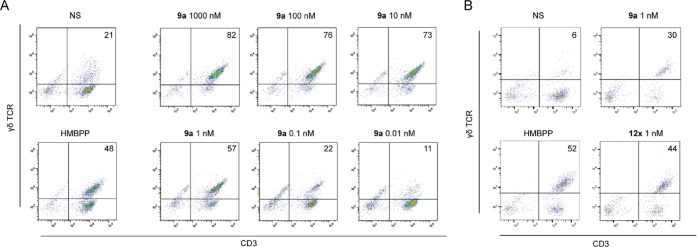
Selected data for stimulation of Vγ9Vδ2 T cell proliferation. (A) Dose response of compound **9a** relative to positive (100 nM HMBPP) and negative controls (NS = not stimulated). (B) Stimulation by 1 nM of compounds **9a** and **12x** relative to positive (100 nM HMBPP) and negative controls (NS).

The compounds were tested for their ability to stimulate proliferation of primary human Vγ9Vδ2 T cells (Figure 2, Table 2). In this assay, peripheral blood mononuclear cells from healthy human donors are exposed to test compounds for 72 h, washed, and allowed to proliferate for 11 additional days, after which the Vγ9Vδ2 T cell population is quantified by flow cytometry (as in Figure 2A). Of the aryl modifications, all compounds stimulated proliferation in the low nanomolar to high picomolar range. An approximately 25-fold range of potency was observed, with the most potent compound being **9c** (proliferation EC_50_ = 0.48 nM) and the least potent compound being **8k** (EC_50_ = 12 nM). In this set, the presence of the allylic acetate improved activity of 10 of 11 compounds relative to the allylic alcohol form by an average of 1.9-fold. With the exception of compounds **9c** and **8j**, all the analogs tested were similarly or less potent than the lead compounds **8a/9a**.

**Table 2:** EC_50_ Values for Stimulation of Vγ9Vδ2 T Cell Proliferation

	*c* log *P*	72 h EC_50_ [nM] (95% CI)	fold improvement vs **8a**	fold improvement OAc/OH
HMBPP (**1**)^15^	–4.59 (dianion)	0.51	NA	NA
C-HMBP (**2**)^15^	–3.29 (monoanion)	4000	NA	NA
POM2-C-HMBP (**3a**)^15^	3.42	5.4	NA	NA
**3c**(26)	2.38	0.28	NA	NA
**8a**	3.56	1.0 (0.56 to 1.9)	1.0	
**9a**	4.26	0.80 (0.32 to 2.0)	1.3	1.3
**8b**	4.72	2.5 (1.1 to 6.1)	0.4	
**9b**	5.42	1.4 (0.79 to 2.5)	0.7	1.8
**8c**	3.77	2.3 (0.42 to 13)	0.4	
**9c**	4.47	0.48 (0.008 to 29)	2.1	4.8
**8d**	2.78	5.0 (0.045 to 540)	0.2	
**9d**	3.48	1.8 (0.73 to 4.2)	0.6	2.8
**8e**	3.11	5.3 (2.6 to 11)	0.2	
**9e**	3.82	2.5 (1.3 to 4.6)	0.4	2.1
**8f**	4.01	1.9 (0.81 to 4.4)	0.5	
**9f**	4.71	1.5 (0.089 to 25)	0.7	1.3
**8g**	4.46	7.1 (4.0 to 13)	0.1	
**9g**	5.16	4.5 (3.2 to 6.3)	0.2	1.6
**8h**	4.41	1.2 (0.0049 to 295)	0.8	
**9h**	5.11	0.94 (0.14 to 6.2)	1.1	1.3
**8i**	5.07	1.9 (0.63 to 6.1)	0.5	
**9i**	5.78	1.5 (0.54 to 4.5)	0.7	1.3
**8j**	4.55	0.76 (0.0096 to 59)	1.3	
**9j**	5.26	1.3 (0.50 to 3.3)	0.8	0.6
**8k**	5.54	12 (0.3 to 490)	0.1	
**9k**	6.25	7 (0.21 to 240)	0.1	1.7
**11x**	3.79	0.34 (0.33 to 0.35)	2.9	
**12x**	4.50	0.47 (0.45 to 0.50)	2.1	0.7
**11y**	2.98	5.2 (1.0 to 26)	0.2	
**12y**	3.69	2.2 (1.5 to 3.2)	0.5	2.4
**11z**	4.29	0.6 (0.18 to 2.1)	1.7	
**12z**	5.00	0.38 (0.09 to 1.6)	2.6	1.6

Of the three pairs of acyloxymethyl modifications, an approximately 15-fold range of potency was observed. The phenyl analog **11x** (EC_50_ = 0.34 nM) improved potency in this assay relative to compound **8a**, as did compound **12x** versus **9a** (Figure 2B). Likewise, the cyclohexyl analogs **11z/12z** were also about 2-fold more potent than **8/9a** in this assay. The isopropyl analogs **11y** and **12y** were less potent relative to the POM version. Taken together, we found modifications to all three positions (aryl, acyloxymethyl, and allylic alcohol) could improve the potency of compound **8a** in 72 h stimulation experiments. Furthermore, the compounds as a group exceeded the potency of the original bis-POM form **3a** and several compounds approached the potency of the most potent aryl amidate **3c**.

### Short Exposures of Test Compounds Enable Leukemia Cells to Stimulate Interferon γ Production by Vγ9Vδ2 T Cells

While the 72 h proliferation assay allows for full internalization of test compounds and is routinely used for determination of pAg activity in primary cells, the SAR of these highly potent compounds for triggering response to leukemia cells is better assessed at a reduced exposure time.^30^ This is because the prodrugs are designed to increase the rate of internalization and meaningful differences in internalization rate are best quantified during the time frame of peak internalization, rather than at a long end point. Therefore, the compounds were next tested for their ability to enable leukemia cells to stimulate T cell production of interferon γ (Table 3). In this assay, K562 leukemia cells were exposed to test compounds for only 1 h, washed, and mixed with purified Vγ9Vδ2 T cells. After an overnight incubation, the amount of interferon γ secreted by the T cells was measured by ELISA. This assay provides an alternative means to assess the compound SAR, where the cell line reduces biological variability, and the shorter 1 h exposure time better captures the speed of action for these fast-acting prodrugs.

**Table 3:** EC_50_ Values for Secretion of Interferon γ by T Cells in Response to Compound Loaded K562 Cells

compound	1 h EC_50_ [nM] (95% CI)	fold improvement vs **8a**	fold improvement OAc/OH
HMBPP (**1**)^15^	>100,000	NA	NA
POM2-C-HMBP (**3a**)^15^	30	NA	NA
**3c**(26)	79	NA	NA
**8a**	11 (7.0 to 17)	1.0	
**9a**	9.4 (6.2 to 14)	1.2	1.2
**8b**	18 (10 to 33)	0.6	
**9b**	11 (7.5 to 15)	1.0	1.6
**8c**	21 (12 to 35)	0.5	
**9c**	6.6 (0.38 to 12)	1.7	3.2
**8d**	76 (24 to 240)	0.1	
**9d**	57 (24 to 130)	0.2	1.3
**8e**	27 (17 to 44)	0.4	
**9e**	32 (18 to 59)	0.3	0.8
**8f**	2.4 (1.6 to 3.5)	4.6	
**9f**	3.0 (1.4 to 6.3)	3.7	0.8
**8g**	56 (0.12 to 25)	0.2	
**9g**	6.0 (0.49 to 75)	1.8	9.3
**8h**	8.0 (5.2 to 12)	1.4	
**9h**	13 (2.9 to 61)	0.8	0.6
**8i**	3.8 (1.9 to 7.6)	2.9	
**9i**	1.9 (1.3 to 2.9)	5.8	2.0
**8j**	8.0 (1.4 to 45)	1.4	
**9j**	9.3 (2.2 to 38)	1.2	0.9
**8k**	200 (45 to 870)	0.1	
**9k**	240 (150 to 390)	0.0	0.8
**11x**	4.1 (1.6 to 10)	2.7	
**12x**	2.4 (0.64 to 8.9)	4.6	1.7
**11y**	17 (5.8 to 48)	0.6	
**12y**	4.9 (2.2 to 11)	2.2	3.5
**11z**	7.2 (4.7 to 11)	1.5	
**12z**	2.4 (1.9 to 3.0)	4.6	3.0

**Figure 3:**
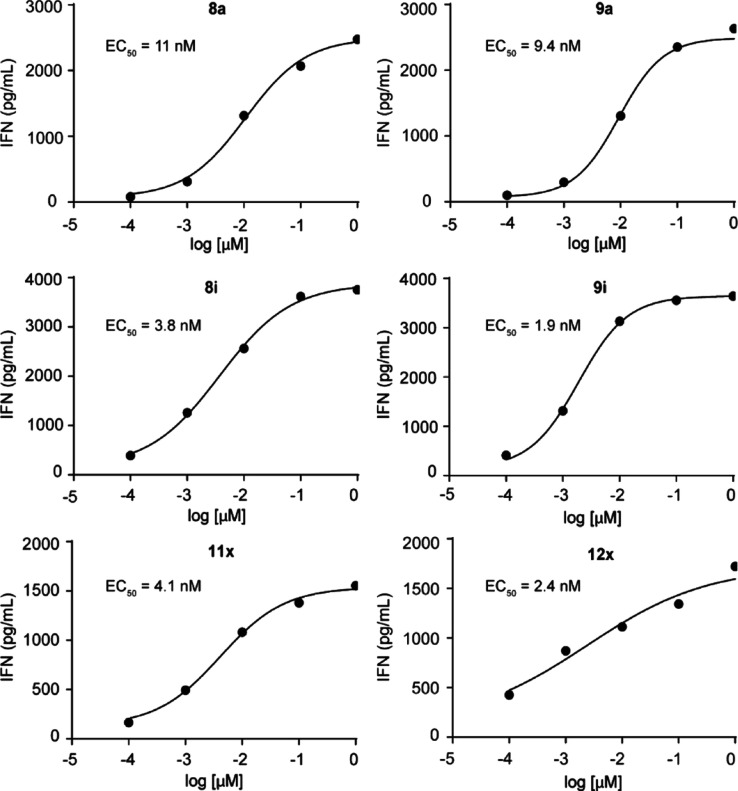
Amount of interferon-γ produced by Vγ9Vδ2 T cells after exposure to loaded K562 cells. Interferon-γ was quantified by ELISA after 1 h K562 loading and 20 h coculture (mean, *n* = 4).

Of the aryl group modifications, all compounds stimulated cytokine production in the mid to low nanomolar range. The compounds appear slightly less potent in this assay relative to the proliferation assay, which was expected given the much shorter incubation period. An approximately 120-fold range of potency was observed, with the most potent compound being **9i** (ELISA EC_50_ = 1.9 nM) (Figure 3) and the least potent compound being **9k** (ELISA EC_50_ = 240 nM) (Table 3). The range was larger relative to the range in the proliferation assay, while a good correlation was observed between the compound activity in the two orthogonal assays (*r* = 0.78, Pearson). Of the acyloxymethyl modifications, an ∼7-fold range of potency was observed. The benzoic acid analog **12x** (ELISA EC_50_ = 2.4 nM) provided an 4.6-fold improved potency in this assay relative to compound **8a**. Compound **12x** was also ∼33-fold more potent than our previously reported value for the aryl amidate **3c**. Likewise, **12z** also showed potency gains in this assay. The allylic acetates also generally increased potency by ∼2-fold, similar to the results in the proliferation assay.

### Decoupling of Cell Potency and Plasma Stability in Esterase Labile Prodrugs

**Figure 4:**
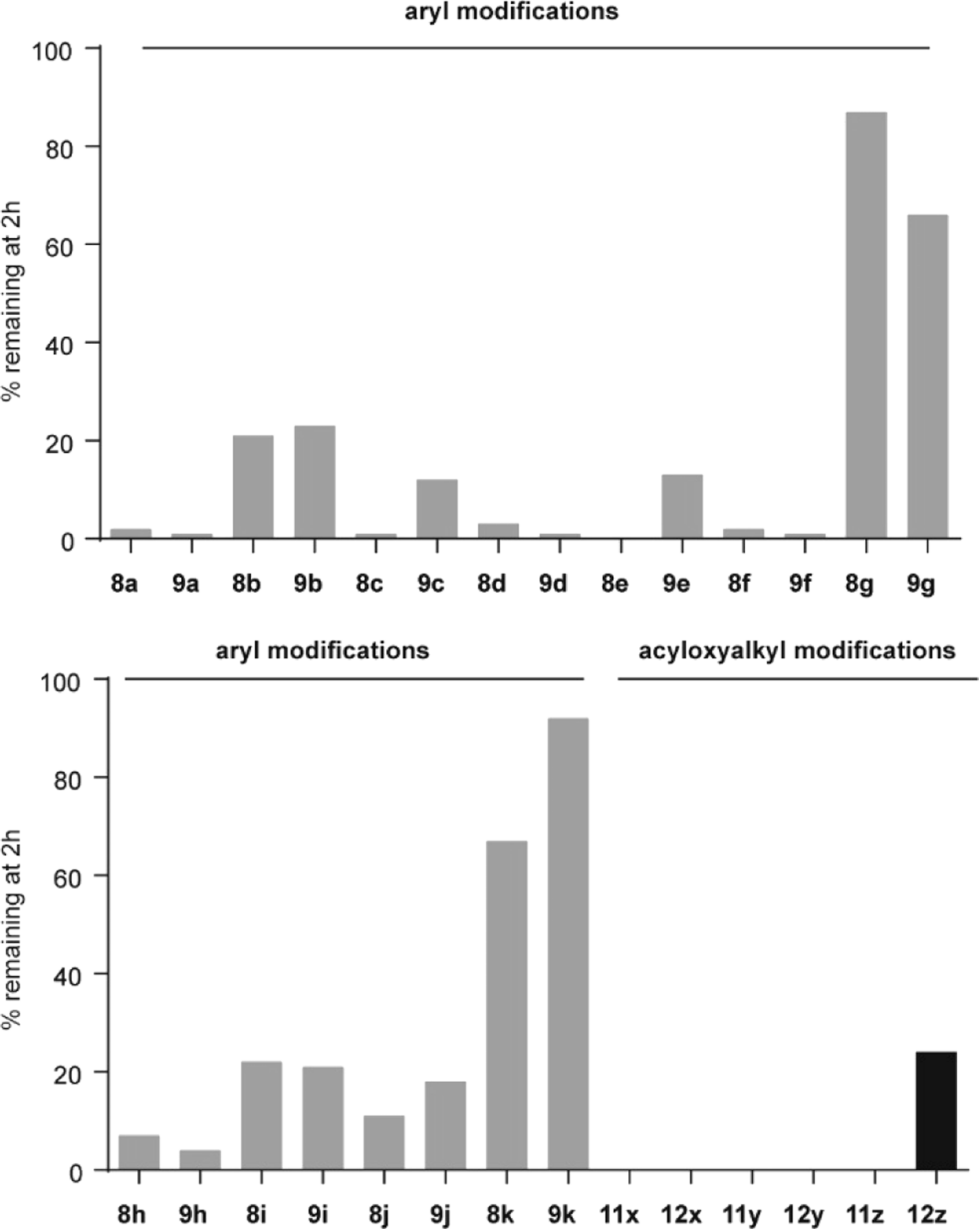
Metabolism of synthesized compounds by 50% pooled human plasma in tris-buffered saline.

In addition to improving cell potency, we also wanted to determine whether plasma stability could be improved in these compounds, as it is possible that these two features are intricately linked to esterase susceptibility. The compounds were evaluated for human plasma stability. We first screened all of the compounds for stability after a 2 or 24 h exposure to pooled human plasma, 50% in tris-buffered saline (Figures 4 and S1). As expected, compound **8a** was almost entirely metabolized after 2 h plasma exposure (<2% detectable compound remaining). Of the aryl analogs, multiple compounds were more stable in plasma than compound **8a**. The most stable was compound **9k**, with 92% remaining after 2 h of incubation. Likewise, 87% of compound **8g** remained after 2 h. Unfortunately, compounds **8g** and **9k** were also among the least potent of the compounds in the activity assays. At the same time, significant improvement of plasma stability relative to **8a** was noted with compounds **8i** and **9i**, which themselves were also more potent than **8a** in the ELISA. The acyloxymethyl analogs **11x** and **12x** were also fully metabolized. However, analog **12z** was not fully metabolized at 2 h, and this was one of the more potent compounds in the activity assays. There was no observable difference between allylic alcohols and allylic acetates at this time point, with some being metabolized better than others in each direction. However, these findings suggest that compounds such as **8i/9i** and **12z** can boost both plasma stability and cellular potency.

### Addition of *para*-Isopropyl Group Significantly Improves Plasma Stability of the Aryl/POM Prodrug

**Figure 5:**
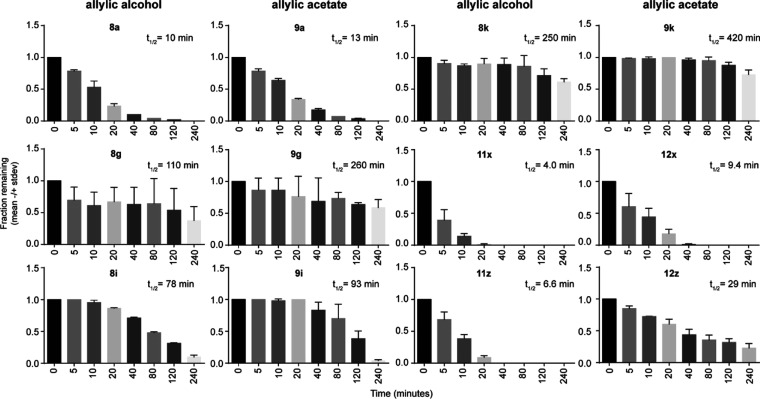
Time course study of the stability in 50% human plasma of selected compounds.

Some of the more interesting compounds were selected for more detailed analysis of plasma stability, again using 50% pooled human plasma in tris-buffered saline (Figure 5). Here, we examined compounds **8i** and **9i** due to their apparent improvements in both stability and potency. These compounds were tested at 8 time points between 0 and 4 h for precise determination of plasma half-lives. The half-life of **8a** was 10 min. Importantly, compound **8i** (half-life = 78 min) was about 7.8-fold more stable in plasma relative to **8a**. As **8i** was more potent than **8a** in the ELISA and they were similarly potent in the proliferation assay, this supports the idea that stability of these compounds can be improved without sacrificing potency.

### Cyclohexyl-Containing Acyloxymethyl Modification Significantly Improves Plasma Stability

It was surprising that compound **12z** was the only acyloxymethyl modification with significant plasma stability in the initial screen. This compound was also more potent than compound **9a** in the activity assays. More detailed time course experiments determined that the half-life of this compound is 29 min (Figure 5), a real improvement relative to compound **9a** (13 min). Again, this further supports the idea that stability of these compounds can be improved without sacrificing potency, this time through modification at the acyloxymethyl position.

### Increased Half-Lives of Allylic Acetates

In our detailed study, we determined half-lives of four pairs of promising allylic alcohols and allylic acetates. In the allylic acetate forms, it is clear that the acyloxymethyl group is removed by plasma esterases faster than the allylic acetate, based on the intermediates observed. Interestingly, half-lives of allylic acetates **9a** (13 min), **9i** (93 min), **12x** (9.4 min), and **12z** (29 min) were all higher than those of their respective free allylic alcohols, suggesting that modifications at that end of the molecule can impact overall metabolism. The half-life of compound **12x** was similar to that of alcohol **8a** and the half-life of **12z** was above that of **8a**, while at the same time, they were more potent in the activity assays. The allylic acetate **9i** only modestly improved the half-life compared to **8i**, from 78 to 93 min, suggesting that there are limitations to this approach. Taken together, the allylic acetate seems to improve plasma stability as well as cell potency.

### Range of Plasma Stability

We also determined half-lives for two pairs of high plasma stability compounds in order to describe the range of a variety of phosphonate prodrugs more completely. At the 240 min time point, a significant portion of compounds **8k**, **9k**, **8g**, and **9g** was still unmetabolized. **8k/9k** were both more stable than **8g/9g**. The half-life of the most stable compound, **9k**, was determined to be 420 min. When compared to the least stable POM-containing compound for which a half-life was determined (**8a**, 10 min), this represents a range of at least 40-fold stability of the POM group boosted by modifications to the aryl substituent.

### Benzoyloxymethyl Group Significantly Improves Cellular Payload Delivery

**Figure 6:**
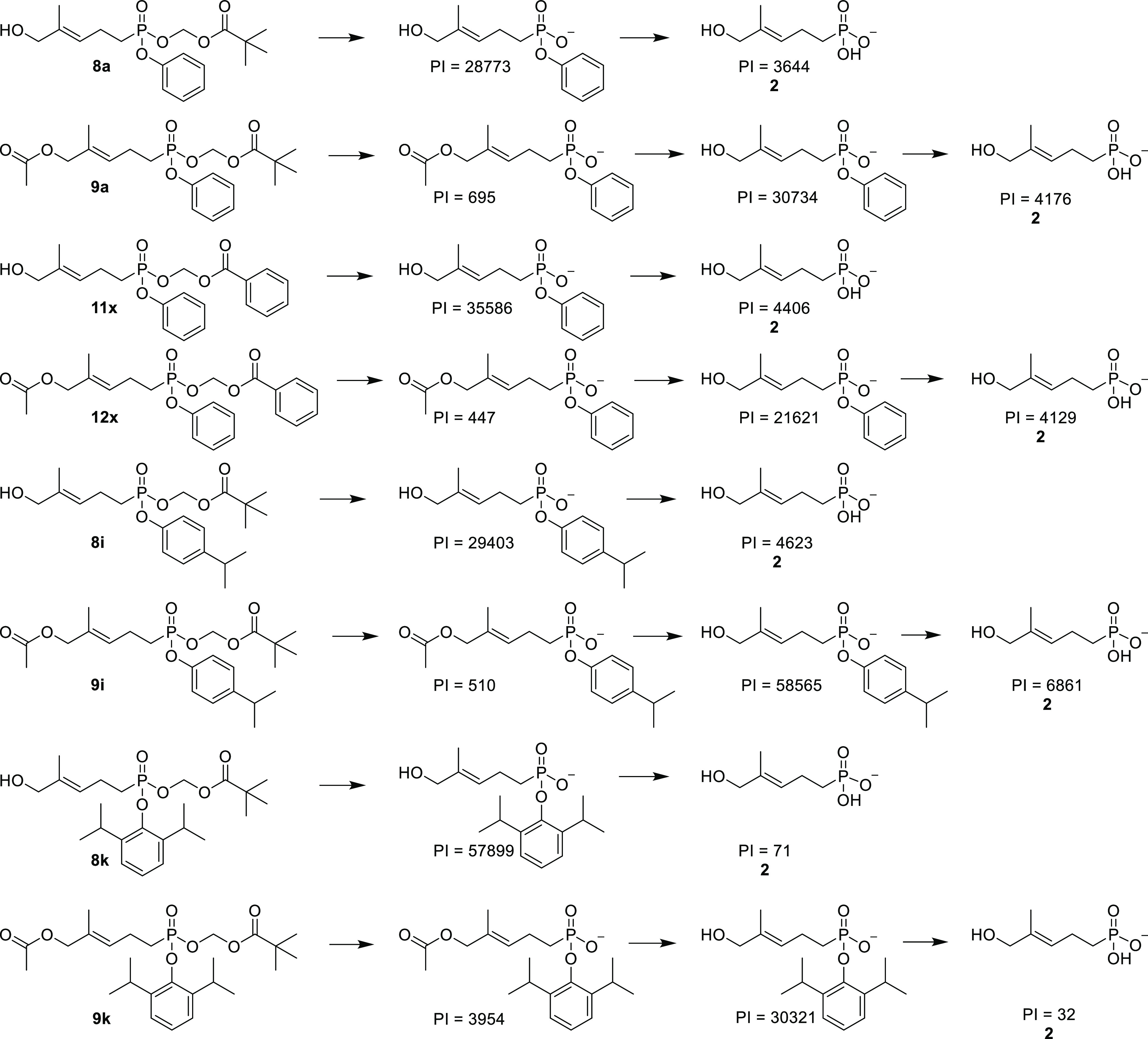
Internalization of the phosphonate payload into K562 cells by selected compounds. PI = integrated peak intensity value.

We next tested the ability of the compounds to deliver the phosphonate payload into K562 cells. The cells were treated for 1 h with compounds **8a**, **9a**, **8k**, **9k**, **8i**, **9i**, **11x**, **12x**, **11z**, or **12z**, and then metabolites were extracted and analyzed by LCMS (Figure 6). Consistent with the activity data, **8i**, **9i**, **11x,** and **12x** all delivered similar if not slightly higher levels of the phosphonate payload relative to **8a** and **9a**. Specifically, the integrated peak intensity of compound **2** extracted from cells treated with compound **8i** and **9i** was 1.3-fold and 1.6 fold higher than cell treated with compounds **8a** and **9a**, respectively. These increased payload levels are correlated with a high potency of **8/9i** relative to **8/9a** in the ELISA. As a control, compounds **8k** and **9k**, which were among the weakest for stimulation, delivered strikingly low levels of free phosphonate payload. Compounds **11z/12z** also delivered meaningful levels (Figure S1). The ratio of payload **2** to the corresponding monoanionic intermediate is displayed in Table S1, which shows that for the more potent compounds, the free compound **2** ranged from 11 to 19% of the monoanion intermediate after incubation in K562 cells, while for the least potent compounds **8/9k,** this value was under 1%. Therefore, the intracellular payload amount seems to at least loosely correspond with cellular potency.

For the active compounds, meaningful levels of the aryl anions but not the acyloxymethyl anions were observed, indicating that like in the plasma, the acyloxymethyl group is metabolically released prior to the aryl group. In contrast to the plasma experiments, in cells, only very low levels of the allylic acetate were observed, suggesting faster cellular cleavage versus plasma cleavage at this site. Thus, the allylic acetate has three advantages over the alcohol: increased potency, increased plasma stability, and selectivity for intracellular metabolism.

**Figure 7:**
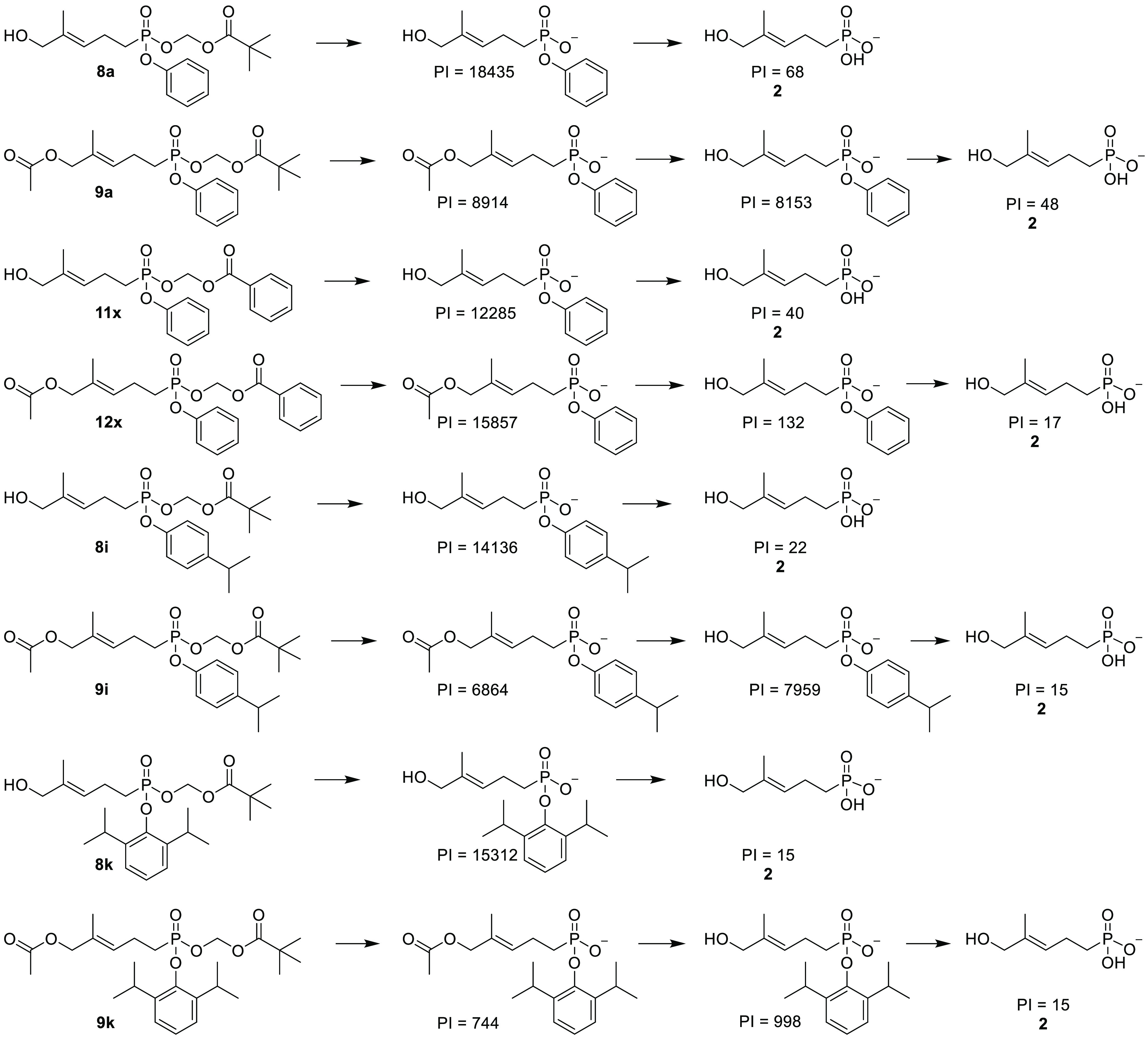
Metabolites produced by plasma metabolism of selected compounds. PI = integrated peak intensity value.

This subset of compounds was evaluated further in the plasma metabolism assay to determine whether the metabolites produced by the plasma were the same as those produced by the cells (Figures 7 and S2). Interestingly, plasma esterases were able to effectively remove the acyloxymethyl esters to produce the monoanionic intermediates, as judged by the appearance of the corresponding peaks. However, they were unable to fully release the active dianionic payload **2** as near zero peak intensities were observed. The ratio of payload **2** to the corresponding monoanionic intermediate is displayed in Table S2, which shows that the free compound **2** is less than 1% of the monoanion intermediate after incubation in plasma, which is much less than the cellular study. This finding clearly demonstrates that cleavage of the phosphonate aryl ester is enzymatic rather than spontaneous, and only cells but not plasma have the requisite enzyme to accomplish the payload release, at least within the relevant 1 h time frame.

### Discussion

Here, we have described the synthesis and evaluation of 28 prodrugs of the BTN3A1 ligand C-HMBP (**2**) including the prior lead compound **8a**.^25^ The compounds were evaluated for potency in models of T cell proliferation and cytokine production, as well as evaluated for metabolism in models of human plasma stability and cell payload release. Several compounds showed notable improvements over the lead **8a**: most notable were the *para*-isopropyl compound **9i**, the cyclohexyl analog **12z,** and the benzoyl analog **12x**.

A primary goal of our study was to identify compounds that have improved potency relative to compound **8a**. These studies revealed that compound **11x** was about 3-fold more potent than **8a** in both the proliferation assay and the ELISA (Tables 2 and 3). With a proliferation EC_50_ of 0.34 nM, it is among the most potent known pAg prodrugs, similar to the prior aryl amidates. In the 1 h ELISA assay, **12x** is approximately 33-fold more potent than the best prior aryl amidate **3c** (Table 3). Similar *c* log *P* values versus **8a/9a** suggest these changes are likely due to rate of cellular metabolism rather than internalization.

A second goal of our study was to determine whether it was possible to reduce plasma metabolism while increasing cellular metabolism. Indeed, we found that the *para*-isopropyl compound **9i** showed a 5-fold improvement versus **9a** in the ELISA assay and a 7-fold increase in plasma stability, or a 35-fold overall improvement. Therefore, the isopropyl modification potentially improves permeability and rapid cellular release by esterases, while decreasing metabolism. Similarly, compound **12z** showed a 4-fold improvement in the ELISA and a 2-fold improvement in plasma stability, resulting in an 8-fold overall improvement. Also notable are *ortho*-isopropyl compounds **8j/9j**, which displayed similar potencies relative to **8/9a** in the activity assays while also more stable in the plasma. Taken together, we found that it is possible to modify these prodrug groups to boost cellular activity and improve plasma stability at the same time, though modifications at either phosphonester position.

It is also informative to compare the *para* isopropyl analogs **8/9i** to the *para* trifluoromethyl analogs **8/9g**. These compounds are roughly similar in size but different in their electronics. Interestingly, both compounds improved plasma stability, suggesting that size rather than electronegativity has a stronger impact on plasma metabolism. However, the cell activity of **8/9i** was consistently stronger than **8/9g**, suggesting that electronegativity at this position decreases cellular metabolism.

Our study also was able to assess the impact of an allylic acetate on the potency and stability of the pAgs. In general, we found about a 2-fold increase in potency of the allylic acetates compared to allylic alcohol forms, which we had predicted. What we did not predict was that they also improved plasma stability in key compounds and that they had selectivity for cellular versus plasma metabolism. However, this impact was variable in different molecules, and further studies would be needed to detail this effect.

In cells, the active compounds are clearly metabolized quickly to release the phosphonate payload. Based on the intermediates that were observed, the acyloxymethyl group is usually liberated first, followed by the release of the aryl substituent. Compounds **8/9i** drove a higher intracellular level of the free acid payload compound **2** relative to **8/9a**, corresponding to their increased potency in the ELISA. This result again illustrates that the isopropyl modification improves cellular release while decreasing plasma metabolism. Notably, the aryl ester linkages were not broken following incubation in plasma, while they were following cellular incubation. Therefore, hydrolysis of the aryl ester is most likely enzymatic rather than spontaneous. Furthermore, hydrolysis of the aryl ester is likely to be catalyzed by a different enzyme than that which cleaves the acyloxymethyl side chain, and the aryl esterase is not found in plasma.

There were some limitations to our study. Specifically, at this time, we do not know which enzymes are responsible for compound metabolism. Further studies will be necessary to determine this, and it remains possible that there are multiple cellular enzymes capable of the prodrug release. A second limitation is that the payload release in the 1 h cell metabolism experiments did not completely correlate with the potency in the 1 h ELISA experiments, as compound **12x** was more potent but delivered similar payload amounts as **9a**. One possibility for the difference is the longer processing time of the ELISA experiment after the 1 h incubation, but at this time, we cannot fully determine the reason why it did not align for this compound. A third limitation is that we are at this time unsure whether the phosphonate payload is phosphorylated to C-HMBPP in cells. We have previously looked for this phosphorylated analog using our LCMS approach but have not found it. It remains possible that C-HMBPP is the active species.

Our findings have implications for future design of phosphonate prodrugs. Both bis-POM (adefovir dipivoxil) and aryl amidate (tenofovir alafenamide) phosphonate prodrugs have achieved clinical use. Our study shows that, at least when applied to the phosphoantigen payload C-HMBP (**2**), aryl acyloxymethyl prodrugs may provide improved plasma stability relative to the bis-POM forms while providing increased potency relative to the aryl amidate forms. However, at this time, while gains were made in the plasma stability, it has not yet reached the level of the aryl amidates, which can demonstrate plasma half-lives in excess of 24 h or more. Thus, further study is needed in this regard and may need to be individualized for each phosphonate-containing drug.

## Experimental Section

### Chemical Synthesis

#### General Experimental Procedures

Diethyl ether, toluene and tetrahydrofuran were freshly distilled from sodium/benzophenone, while acetonitrile and methylene chloride were distilled from calcium hydride prior to use. All other reagents were purchased from commercial sources and used without further purification. All the reactions in nonaqueous solvents were conducted in flame-dried glassware under a positive pressure of nitrogen and with magnetic stirring. All NMR spectra were obtained at 400 or 500 MHz for ^1^H, 101 or 126 MHz for ^13^C, 162 or 203 MHz for ^31^P, and 471 MHz for ^19^F with internal standards of (CH_3_)_4_Si (^1^H, 0.00) or CDCl_3_ (^1^H, 7.27; ^13^C, 77.2 ppm) for nonaqueous samples. The ^31^P chemical shifts were reported in ppm relative to 85% H_3_PO_4_ (external standard). High resolution mass spectra (HRMS) were obtained at the University of Iowa Mass Spectrometry Facility (Thermo Q-Exactive Orbitrap). Silica gel (60 Å, 0.040–0.063 mm) was used for flash chromatography. The purity profile of each assayed compound was evaluated with an Agilent 1220 series HPLC (100% methanol, Column dimension: Restek ultrasilica, 5 μm, C18, 250 × 4.6 mm (analytical), flow rate: 1.0 mL/min) and all assayed compounds had a purity >95%.

##### ((*E*)-(((5-Acetoxy-4-methylpent-3-en-1-yl)(phenoxy)phosphoryl)oxy)methyl pivalate) (**9a**)

Alcohol **8a**(25) (54 mg, 0.14 mmol), acetic anhydride (22 mg, 0.21 mmol), and triethylamine (29 mg, 0.29 mmol) were dissolved in freshly distilled methylene chloride (5 mL), and the resultant reaction mixture was allowed to react overnight at room temperature. The reaction mixture was diluted by the addition of water (5 mL), quenched with sodium bicarbonate (2 mL), and extracted with methylene chloride (3 × 5 mL). The combined extracts were dried (Na_2_SO_4_) and filtered through Celite, and the filtrate was concentrated in vacuo. The residue was purified by column chromatography (silica gel, 100% hexanes −50% EtOAc in hexanes), and the resulting product **9a** was isolated as an oil in 98% yield (59 mg): ^1^H NMR (500 MHz, CDCl_3_): δ 7.39–7.29 (m, 2H), 7.25–7.12 (m, 3H), 5.73 (dd, *J*_PH_ = 13.6, *J*_HH_ = 5.0 Hz, 1H), 5.66 (dd, *J*_PH_ = 12.4, *J*_HH_ = 5.2 Hz, 1H), 5.46 (td, *J* = 7.2, 1.4 Hz, 1H), 4.45 (s, 2H), 2.53–2.37 (m, 2H), 2.07 (s, 3H), 2.05–1.97 (m, 2H), 1.67 (s, 3H), 1.18 (s, 9H): ^13^C NMR (126 MHz, CDCl_3_): δ 176.7, 170.6, 149.8 (d, *J*_PC_ = 10.1 Hz), 131.7, 129.6 (2C), 126.6 (d, *J*_PC_ = 17.6 Hz), 125.0, 120.3 (2C), 81.6 (d, *J*_PC_ = 6.3 Hz), 69.3, 38.5, 26.6 (3C), 25.2 (d, *J*_PC_ = 139.9 Hz), 20.7, 20.4 (d, *J*_PC_ = 5.0 Hz), 13.7; ^31^P NMR (203 MHz, CDCl_3_) 28.8 ppm. HRMS (ESI^+^) *m*/*z*: calcd for C_20_H_29_NaO_7_P (M + Na)^+^, 435.1549; found, 435.1535. HPLC purity 96% (*t*_R_ = 3.3).

##### (*E*)-(((5-Acetoxy-4-methylpent-3-en-1-yl)(naphthalen-1-yloxy)phosphoryl)oxy)methyl pivalate) (**9b**)

Alcohol **8b**(25) (60 mg, 0.14 mmol), acetic anhydride (22 mg, 0.21 mmol), and triethylamine (29 mg, 0.28 mmol) were dissolved in freshly distilled methylene chloride (5 mL), and the resultant reaction mixture was allowed to react overnight at room temperature. The reaction mixture was diluted by the addition of water (5 mL), quenched with sodium bicarbonate (2 mL), and extracted with methylene chloride (3 × 5 mL). The combined extracts were dried (Na_2_SO_4_) and filtered through Celite, and the filtrate was concentrated in vacuo. The residue was purified by column chromatography (silica gel, 100% hexane −50% EtOAc in hexane), and the resulting product **9b** was isolated as an oil in 98% yield (65 mg): ^1^H NMR (500 MHz, CDCl_3_): δ 8.14–8.07 (m, 1H), 7.85 (dd, *J* = 7.7, 2.4 Hz, 1H), 7.67 (d, *J* = 9.3 Hz, 1H), 7.57–7.49 (m, 3H), 7.45–7.37 (m, 1H), 5.76 (dd, *J*_PH_ = 13.8, *J*_HH_ = 5.1 Hz, 1H), 5.66 (dd, *J*_PH_ = 12.3, *J*_HH_ = 5.1 Hz, 1H), 5.48 (td, *J* = 7.2, 1.4 Hz, 1H), 4.42 (s, 2H), 2.56–2.44 (m, 2H), 2.20–2.10 (m, 2H), 2.04 (s, 3H), 1.63 (s, 3H), 1.11 (s, 9H); ^13^C NMR (126 MHz, CDCl_3_): δ 176.8, 170.6, 145.8 (d, *J*_PC_ = 10.1 Hz), 134.7, 131.8, 127.6, 126.6, 126.5, 126.5, 126.3, 126.3, 125.4, 124.9, 121.4, 115.5, 81.7 (d, *J*_PC_ = 6.3 Hz), 69.3, 38.5, 26.6 (3C), 26.4 (d, *J*_PC_ = 139.9 Hz), 20.7, 20.5 (d, *J*_PC_ = 5.04 Hz), 13.7; ^31^P NMR (203 MHz, CDCl_3_) 29.1 ppm. HRMS (ESI^+^) *m*/*z*: calcd for C_24_H_31_NaO_7_P (M + Na)^+^, 485.1705; found, 485.1691. HPLC purity >98% (*t*_R_ = 3.3).

##### Methyl (*E*)-2-(((5-Hydroxy-4-methylpent-3-en-1-yl)((pivaloyloxy)methoxy)phosphoryl)oxy) Benzoate (**8c**)

The olefin **7c** (520 mg, 1.2 mmol), prepared through a reaction sequence parallel to those previously reported for compounds **7a** and **7b**,^25^ was added to a solution of selenium dioxide (104 mg, 0.9 mmol) and a 70% solution of *tert*-butyl hydroperoxide (0.509 mL, 3.7 mmol) in dichloromethane (10 mL). This reaction mixture was stirred at room temperature and allowed to react for 3 days. The reaction mixture was diluted by the addition of water (5 mL), quenched with Na_2_SO_3_ (5 mL), extracted with dichloromethane (3 × 10 mL), and concentrated in vacuo. The resulting oil was dissolved in anhydrous THF (10 mL) and allowed to react with sodium borohydride (48 mg, 1.25 mmol) for 1 h. The reaction mixture was quenched by the addition of saturated ammonium chloride (5 mL) and extracted with diethyl ether (3 × 10 mL). The combined extracts were dried (Na_2_SO_4_) and filtered through Celite, and the filtrate was concentrated in vacuo. The resulting red oil was purified by column chromatography (silica, 100% hexane −55% EtOAc in hexane) to give the desired product **8c** as an oil in 4.6% yield (25 mg): ^1^H NMR (500 MHz, CDCl_3_): δ 7.88 (dd, *J* = 7.9, 1.1 Hz, 1H), 7.53–7.42 (m, 2H), 7.24 (t, *J* = 7.0 Hz, 1H), 5.56 (dd, *J*_PH_ = 12.4, *J*_HH_ = 5.1 Hz, 1H), 5.51 (dd, *J*_PH_ = 12.7, *J*_HH_ = 5.0 Hz, 1H), 5.29 (td, *J* = 7.2, 1.4 Hz, 1H), 3.99 (s, 2H), 3.90 (s, 3H), 2.59–2.40 (m, 2H), 2.18–1.98 (m, 2H), 1.68 (s, 3H), 1.18 (s, 9H); ^13^C NMR (126 MHz, CDCl_3_): δ 177.0, 165.3, 149.0 (d, *J*_PC_ = 8.8 Hz), 136.6, 133.6, 131.8, 124.9, 123.3 (d, *J*_PC_ = 16.2 Hz), 123.2 (d, *J*_PC_ = 6.2 Hz), 122.2 (d, *J*_PC_ = 3.8 Hz), 81.9 (d, *J*_PC_ = 6.3 Hz), 68.3, 52.2, 38.7, 26.8 (3C), 26.4 (d, *J*_PC_ = 138.8 Hz), 20.3 (d, *J*_PC_ = 5.1 Hz), 13.6; ^31^P NMR (203 MHz, CDCl_3_) 29.4 ppm. HRMS (ESI^+^) *m*/*z*: calcd for C_20_H_29_NaO_8_P (M + Na)^+^, 451.1498; found, 451.1490. HPLC purity >99% (*t*_R_ = 3.34).

##### Methyl (*E*)-2-(((5-Acetoxy-4-methylpent-3-en-1-yl)((pivaloyloxy)methoxy)phosphoryl)oxy) Benzoate (**9c**)

Alcohol **8c** (15 mg, 0.035 mmol), acetic anhydride (5 mg, 0.052 mmol), and triethylamine (7 mg, 0.070 mmol) were dissolved in freshly distilled methylene chloride (3 mL), and the resultant reaction mixture was allowed to react overnight at room temperature. The reaction mixture was diluted by the addition of water (5 mL), quenched with sodium bicarbonate (2 mL), and extracted with methylene chloride (3 × 5 mL). The combined extracts were dried (Na_2_SO_4_) and filtered through Celite, and the filtrate was concentrated in vacuo. The residue was purified by column chromatography (silica gel, 100% hexane −30% EtOAc in hexane), and the resulting product **9c** was isolated as an oil in 97% yield (16 mg): ^1^H NMR (500 MHz, CDCl_3_): δ 7.98–7.82 (m, 1H), 7.56–7.41 (m, 2H), 7.24 (t, *J* = 8.1 Hz, 1H), 5.71 (dd, *J*_PH_ = 12.7, *J*_HH_ = 5.0 Hz, 1H), 5.65 (dd, *J*_PH_ = 12.7, *J*_HH_ = 5.2 Hz, 1H), 5.47 (td, *J* = 7.2, 1.4 Hz, 1H), 4.45 (s, 2H), 3.90 (s, 3H), 2.61–2.40 (m, 2H), 2.17–2.00 (m, 5H), 1.68 (s, 3H), 1.18 (s, 9H); ^13^C NMR (126 MHz, CDCl_3_): δ 176.8, 170.8, 165.2, 149.0 (d, *J*_PC_ = 8.8 Hz), 133.6, 131.8, 131.8, 127.1 (d, *J*_PC_ = 18.9 Hz), 125.0, 123.2 (d, *J*_PC_ = 5.1 Hz), 122.3 (d, *J*_PC_ = 2.5 Hz), 81.9 (d, *J*_PC_ = 6.3 Hz), 69.6, 52.2, 38.7, 26.8 (3C), 26.2 (d, *J*_PC_ = 139.9 Hz), 20.9, 20.5 (d, *J*_PC_ = 3.8 Hz), 13.9; ^31^P NMR (203 MHz, CDCl_3_) 29.1 ppm. HRMS (ESI^+^) *m*/*z*: calcd for C_22_H_31_NaO_9_P (M + Na)^+^, 493.1603; found, 493.1594. HPLC purity >99% (*t*_R_ = 3.33).

##### (*E*)-(((4-Acetamidophenoxy)(5-hydroxy-4-methylpent-3-en-1-yl)phosphoryl)oxy)methyl Pivalate (**8d**)

The olefin **7d** (420 mg, 1.02 mmol) was added to a solution of selenium dioxide (90 mg, 0.75 mmol) and a 70% solution of *tert*-butyl hydroperoxide (0.421 mL, 3.0 mmol) in dichloromethane (10 mL). This reaction mixture was stirred at room temperature and was allowed to react for 2 days. The reaction mixture was diluted by the addition of water (5 mL), quenched with Na_2_SO_3_ (5 mL), extracted with dichloromethane (3 × 10 mL), and concentrated in vacuo. The resulting reddish oil was dissolved in THF (5 mL) and allowed to react with sodium borohydride (38 mg, 1.0 mmol) for 30 min. The reaction mixture was quenched by the addition of saturated ammonium chloride (5 mL) and extracted with diethyl ether (3 × 10 mL). The combined extracts were dried (Na_2_SO_4_) and filtered through Celite, and the filtrate was concentrated in vacuo. The resulting oil was purified by column chromatography (silica, 100% ether −3% MeOH in ether) to give the desired product **8d** as an oil in 16% yield (70 mg): ^1^H NMR (500 MHz, CDCl_3_): δ 8.09 (s, 1H), 7.46 (d, *J* = 9.0 Hz, 2H), 7.10 (d, *J* = 9.0 Hz, 2H), 5.70 (dd, *J*_PH_ = 13.0, *J*_HH_ = 5.0 Hz, 1H), 5.63 (dd, *J*_PH_ = 12.5, *J*_HH_ = 5.0 Hz, 1H), 5.41 (td, *J* = 7.2, 1.5 Hz, 1H), 3.99 (s, 2H), 2.44–2.33 (m, 2H), 2.14 (s, 3H), 2.09–1.94 (m, 2H), 1.66 (s, 3H), 1.19 (s, 9H); ^13^C NMR (126 MHz, CDCl_3_): δ 176.9, 168.6, 145.6 (d, *J*_PC_ = 8.8 Hz), 136.6, 135.5, 122.7 (d, *J*_PC_ = 16.3 Hz), 121.2 (2C), 120.7 (d, *J*_PC_ = 3.8 Hz, 2C), 81.6 (d, *J*_PC_ = 6.3 Hz), 67.9, 38.6, 26.7 (3C), 25.9 (d, *J*_PC_ = 137.5 Hz), 24.1, 20.3 (d, *J*_PC_ = 5.0 Hz), 13.5; ^31^P NMR (203 MHz, CDCl_3_) 29.5 ppm. HRMS (ESI^+^) *m*/*z*: calcd for C_20_H_30_NNaO_7_P (M + Na)^+^, 450.1658; found, 450.1644. HPLC purity >97% (*t*_R_ = 3.23).

##### (*E*)-(((4-Acetamidophenoxy)(5-acetoxy-4-methylpent-3-en-1-yl)phosphoryl)oxy)methyl Pivalate (**9d**)

Alcohol **8d** (25 mg, 0.058 mmol), acetic anhydride (9 mg, 0.087 mmol), and triethylamine (12 mg, 0.12 mmol) were dissolved in freshly distilled methylene chloride (5 mL), and the resultant reaction mixture was allowed to react overnight at room temperature. The reaction mixture was diluted by the addition of water (5 mL), quenched with sodium bicarbonate (2 mL), and extracted with methylene chloride (3 × 5 mL). The combined extracts were dried (Na_2_SO_4_) and filtered through Celite, and the filtrate was concentrated in vacuo. The residue was purified by column chromatography (silica gel, 100% hexanes −50% EtOAc in hexanes), and the resulting product **9d** was isolated as an oil in 98% yield (27 mg): ^1^H NMR (500 MHz, CDCl_3_): δ 8.08 (s, 1H), 7.45 (d, *J* = 9.0 Hz, 2H), 7.10 (d, *J* = 8.9 Hz, 2H), 5.71 (dd, *J*_PH_ = 13.1, *J*_HH_ = 5.2 Hz, 1H), 5.63 (dd, *J*_PH_ = 12.4, *J*_HH_ = 5.1 Hz, 1H), 5.45 (td, *J* = 7.2, 1.4 Hz, 1H), 4.44 (s, 2H), 2.49–2.38 (m, 2H), 2.14 (s, 3H), 2.06 (s, 3H), 2.02–1.97 (m, 2H), 1.67 (s, 3H), 1.19 (s, 9H); ^13^C NMR (126 MHz, CDCl_3_): δ 176.8, 170.7, 168.4, 145.6 (d, *J*_PC_ = 8.8 Hz), 135.5, 131.9, 126.5 (d, *J*_PC_ = 17.6 Hz), 121.2 (2C), 120.7, (d, *J*_PC_ = 3.8 Hz, 2C), 81.6 (d, *J*_PC_ = 6.3 Hz), 69.3, 38.6, 26.7 (3C), 25.7 (d, *J*_PC_ = 139.8 Hz), 24.2, 20.8, 20.4 (d, *J*_PC_ = 5.0 Hz), 13.8; ^31^P NMR (203 MHz, CDCl_3_) 29.1 ppm. HRMS (ESI^+^) *m*/*z*: calcd for C_22_H_32_NNaO_8_P (M + Na)^+^, 492.1763; found, 492.1749. HPLC purity >97% (*t*_R_ = 3.23).

##### (*E*)-(((4-(2-Acetamido-3-ethoxy-3-oxopropyl)phenoxy)(5-hydroxy-4-methylpent-3-en-1-yl)phosphoryl)oxy)methyl Pivalate (**8e**)

The round-bottom flask was charged with olefin **7e** (130 mg, 0.25 mmol), selenium dioxide (22 mg, 0.2 mmol, 0.8 equiv), and 4-hydroxybenzoic acid (4.9 mg, 0.035 mmol) in dichloromethane (5 mL). At 0 °C, *tert*-butyl hydroperoxide (70% wt in H_2_O, 0.139 mL, 1.0 mmol, 4.0 equiv) was slowly added to the reaction mixture. The resulting reaction mixture was stirred at 0 °C for 3 days. The reaction mixture was diluted by the addition of water (5 mL), quenched with Na_2_SO_3_ (5 mL), and extracted with dichloromethane (3 × 10 mL). The combined extracts were dried (Na_2_SO_4_) and filtered through Celite, and the filtrate was concentrated in vacuo. The resulting oil was purified by column chromatography (silica, 100% ether −3% MeOH in ether) to give the desired product **8e** as an oil in 45% yield (60 mg): ^1^H NMR (500 MHz, CDCl_3_): δ 7.16–7.04 (m, 4H), 6.03 (s, 1H), 5.69 (ddd, *J*_PH_ = 13.0, *J*_HH_ = 5.0, 2.7 Hz, 1H), 5.63 (ddd, *J*_PH_ = 12.7, *J*_HH_ = 5.1, 1.1 Hz, 1H), 5.40 (td, *J* = 7.1 Hz, 1H), 4.19–4.14 (m, 2H), 3.97 (s, 2H), 3.08 (qd, *J* = 14.0, 5.9 Hz, 2H), 2.46–2.36 (m, 2H), 2.05–1.99 (m, 2H), 1.98 (s, 3H), 1.65 (s, 3H), 1.24 (t, *J* = 6.7 Hz, 3H), 1.18 (s, 9H); ^13^C NMR (126 MHz, CDCl_3_): δ 176.9, 171.3, 169.5, 148.9 (d, *J*_PC_ = 5.0 Hz), 136.5, 132.8, 130.5 (2C), 122.9 (d, *J*_PC_ = 15.1 Hz), 120.4, 120.3, 81.5 (dd, *J*_PC_ = 6.3 Hz), 68.0, 61.4, 52.9, 38.5, 37.0, 26.6 (3C), 26.0 (d, *J*_PC_ = 139.8 Hz), 23.0, 20.3 (d, *J*_PC_ = 6.3 Hz), 13.9, 13.5; ^31^P NMR (203 MHz, CDCl_3_) 29.3 ppm. HRMS (ESI^+^) *m*/*z*: calcd for C_25_H_38_NNaO_9_P (M + Na)^+^, 550.2182; found, 550.2168. HPLC purity >98% (*t*_R_ = 3.23).

##### (*E*)-(((4-(2-Acetamido-3-ethoxy-3-oxopropyl)phenoxy)(5-acetoxy-4-methylpent-3-en-1-yl)phosphoryl)oxy)methyl Pivalate (**9e**)

Alcohol **8e** (23 mg, 0.043 mmol), acetic anhydride (9 mg, 0.087 mmol), and triethylamine (12 mg, 0.11 mmol) were dissolved in freshly distilled methylene chloride (5 mL), and the resultant reaction mixture was allowed to react overnight at room temperature. The reaction mixture was diluted by the addition of water (5 mL), quenched with sodium bicarbonate (2 mL), and extracted with methylene chloride (3 × 5 mL). The combined extracts were dried (Na_2_SO_4_) and filtered through Celite, and the filtrate was concentrated in vacuo. The residue was purified by column chromatography (silica gel, 100% hexane −50% EtOAc in hexane), and the resulting product **9e** was isolated as an oil in 96% yield (24 mg): ^1^H NMR (500 MHz, CDCl_3_): δ 7.17–7.07 (m, 4H), 5.94 (s, 1H), 5.70 (ddd, *J*_PH_ = 13.0, *J*_HH_ = 5.0, 2.9 Hz, 1H), 5.64 (ddd, *J*_PH_ = 12.7, *J*_HH_ = 5.1, 1.1 Hz, 1H), 5.45 (td, *J* = 7.2, 1.4 Hz, 1H), 4.88–4.80 (m, 1H), 4.44 (s, 2H), 4.24–4.11 (m, 2H), 3.16–3.04 (m, 2H), 2.49–2.38 (m, 2H), 2.07 (s, 3H), 2.01 (s, 3H), 2.04–1.96 (m, 2H), 1.67 (s, 3H), 1.27–1.23 (t, 3H), 1.19 (s, 9H); ^13^C NMR (126 MHz, CDCl_3_): δ 176.7, 171.3, 170.6, 169.4, 148.9 (d, *J*_PC_ = 5.0 Hz), 132.8, 131.8, 130.5 (2C), 126.7 (d, *J*_PC_ = 17.6 Hz), 120.4, 120.3, 81.6 (dd, *J*_PC_ = 6.3 Hz), 69.3, 61.4, 52.9, 38.5, 37.0, 26.7 (3C), 25.8 (d, *J*_PC_ = 139.8 Hz), 23.0, 20.8, 20.4 (d, *J*_PC_ = 6.3 Hz), 14.0, 13.8; ^31^P NMR (203 MHz, CDCl_3_) 29.9 ppm. HRMS (ESI^+^) *m*/*z*: calcd for C_27_H_40_NNaO_10_P (M + Na)^+^, 592.2288; found, 592.2274. HPLC purity >99% (*t*_R_ = 3.36).

##### (*E*)-(((5-Hydroxy-4-methylpent-3-en-1-yl)(*p*-tolyloxy)phosphoryl)oxy)methyl Pivalate (**8f**)

A round-bottom flask was charged with olefin **7f** (370 mg, 1.0 mmol), selenium dioxide (89 mg, 0.96 mmol), and 4-hydroxybenzoic acid (19.4 mg, 0.14 mmol) in dichloromethane (10 mL). At 0 °C, *tert*-butyl hydroperoxide (70% wt in H_2_O, 0.554 mL, 4.0 mmol) was added slowly to the stirred reaction mixture. The resulting reaction mixture was stirred at 0 °C and was allowed to react for 3 days. The reaction mixture was diluted by the addition of water (5 mL), quenched with Na_2_SO_3_ (5 mL), and extracted with dichloromethane (3 × 10 mL). The combined extracts were dried (Na_2_SO_4_) and filtered through Celite, and the filtrate was concentrated in vacuo. The resulting oil was purified by column chromatography (silica, 100% ether −3% MeOH in ether) to give the desired product **8f** as an oil in 23% yield (90 mg): ^1^H NMR (500 MHz, CDCl_3_): δ 7.15–7.04 (m, 4H), 5.71 (dd, *J*_PH_ = 11.3 Hz, *J*_HH_ = 5.0 Hz, 1H), 5.64 (dd, *J*_PH_ = 13.6 Hz, *J*_HH_ = 4.9 Hz, 1H), 5.41 (td, *J* = 7.0, 1.4 Hz, 1H), 3.99 (s, 2H), 2.47–2.41 (m, 2H), 2.31 (s, 3H), 2.05–1.94 (m, 2H), 1.67 (s, 3H), 1.18 (s, 9H); ^13^C NMR (126 MHz, CDCl_3_): δ 176.9, 147.6 (d, *J*_PC_ = 8.82 Hz), 136.5, 134.7, 130.1 (2C), 123.2 (d, *J*_PC_ = 16.4 Hz), 120.1 (2C), 81.6 (d, *J*_PC_ = 6.3 Hz), 68.1, 38.6, 26.7 (3C), 25.9 (d, *J*_PC_ = 138.6 Hz), 20.6, 20.3 (d, *J*_PC_ = 5.0 Hz), 13.5; ^31^P NMR (203 MHz, CDCl_3_) 29.2 ppm. HRMS (ESI^+^) *m*/*z*: calcd for C_19_H_30_O_6_P (M + H)^+^, 385.1780; found, 385.1768. HPLC purity >95% (*t*_R_ = 3.32).

##### (*E*)-(((5-Acetoxy-4-methylpent-3-en-1-yl)(*p*-tolyloxy)phosphoryl)oxy)methyl Pivalate (**9f**)

Alcohol **8f** (25 mg, 0.065 mmol), acetic anhydride (10 mg, 0.096 mmol), and triethylamine (13 mg, 0.13 mmol) were dissolved in freshly distilled methylene chloride (4 mL), and the resultant reaction mixture was allowed to react overnight at room temperature. The reaction mixture was diluted by the addition of water (5 mL), quenched with sodium bicarbonate (2 mL), and extracted with methylene chloride (3 × 5 mL). The combined extracts were dried (Na_2_SO_4_) and filtered through Celite, and the filtrate was concentrated in vacuo. The residue was purified by column chromatography (silica gel, 100% hexane −50% EtOAc in hexane), and the resulting product **9f** was isolated as an oil in 97% yield (30 mg): ^1^H NMR (500 MHz, CDCl_3_): δ 7.15–7.05 (m, 4H), 5.71 (dd, *J*_PH_ = 11.3 Hz, *J*_HH_ = 5.0 Hz, 1H), 5.64 (dd, *J*_PH_ = 13.6 Hz, *J*_HH_ = 4.9 Hz, 1H), 5.41 (td, *J* = 7.0, 1.4 Hz, 1H), 4.44 (s, 2H), 2.49–2.35 (m, 2H), 2.31 (s, 3H), 2.07 (s, 3H), 2.03–1.94 (m, 2H), 1.67 (s, 3H), 1.18 (s, 9H); ^13^C NMR (126 MHz, CDCl_3_): δ 177.0, 170.8, 147.7(d, *J*_PC_ = 8.8 Hz), 134.8, 131.9, 130.3 (2C), 127.0 (d, *J*_PC_ = 17.6 Hz), 120.2 (2C), 81.8 (d, *J*_PC_ = 6.3 Hz), 69.6, 38.7, 26.8 (3C), 25.9 (d, *J*_PC_ = 138.6 Hz), 20.9, 20.7, 20.6 (d, *J*_PC_ = 5.04 Hz), 14.0; ^31^P NMR (203 MHz, CDCl_3_) 28.8 ppm. HRMS (ESI^+^) *m*/*z*: calcd for C_21_H_32_O_7_P (M + H)^+^, 427.1886; found, 427.1876. HPLC purity >95% (*t*_R_ = 3.30).

##### (*E*)-(((5-Hydroxy-4-methylpent-3-en-1-yl)(4-(trifluoromethyl)phenoxy)phosphoryl)oxy)methyl pivalate) (**8g**)

The olefin **7g** (420 mg, 0.99 mmol) was added to a solution of selenium dioxide (82 mg, 0.73 mmol) and a 70% solution of *tert*-butyl hydroperoxide (0.409 mL, 2.9 mmol) in dichloromethane (10 mL). This reaction mixture was stirred at room temperature and allowed to react for 3 days. The reaction mixture was diluted by the addition of water (5 mL), quenched with Na_2_SO_3_ (5 mL), and extracted with dichloromethane (3 × 10 mL). The combined extracts were dried (Na_2_SO_4_) and concentrated in vacuo. The resulting reddish oil was dissolved in THF (10 mL) and allowed to react with sodium borohydride (38 mg, 1.0 mmol) for 1 h. The reaction mixture was quenched by the addition of saturated ammonium chloride (5 mL) and extracted with diethyl ether (3 × 10 mL). The combined extracts were dried (Na_2_SO_4_) and filtered through Celite, and the filtrate was concentrated in vacuo. The resulting oil was purified by column chromatography (silica, 100% hexane −50% EtOAc in hexane) to give the desired product **8g** as an oil in 12.5% yield (55 mg): ^1^H NMR (500 MHz, CDCl_3_): δ 7.62 (d, *J* = 9.0 Hz, 2H), 7.35 (d, *J* = 8.5 Hz, 2H), 5.71 (dd, *J*_PH_ = 13.6, *J*_HH_ = 5.2 Hz, 1H), 5.64 (dd, *J*_PH_ = 12.4, *J*_HH_ = 5.2 Hz, 1H), 5.43 (td, *J* = 7.2, 1.5 Hz, 1H), 4.00 (s, 2H), 2.56–2.35 (m, 2H), 2.14–2.02 (m, 2H), 1.67 (s, 3H), 1.16 (s, 9H); ^13^C NMR (126 MHz, CDCl_3_): δ 176.8, 152.4 (d, *J*_PC_ = 10.1 Hz), 136.7, 127.1 (q, *J*_CF_ = 3.7 Hz, 2C), 127.1 (q, *J*_CF_ = 37.8 Hz), 123.6 (q, *J*_CF_ = 272.2 Hz), 122.5 (d, *J*_PC_ = 16.4 Hz), 120.7 (d, *J*_PC_ = 5.0 Hz, 2C), 81.6 (d, *J*_PC_ = 6.3 Hz), 67.9, 38.5, 26.6 (3C), 26.1 (d, *J*_PC_ = 139.86 Hz), 20.2 (d, *J*_PC_ = 5.0 Hz), 13.5; ^19^F NMR (471 MHz, CDCl_3_) −62.2 ppm; ^31^P NMR (203 MHz, CDCl_3_) 29.8 ppm. HRMS (ESI^+^) *m*/*z*: calcd for C_19_H_26_F_3_NaO_6_P (M + Na)^+^, 461.1317; found, 461.1307. HPLC purity >99% (*t*_R_ = 3.26).

##### (*E*)-(((5-Acetoxy-4-methylpent-3-en-1-yl)(4-(trifluoromethyl)phenoxy)phosphoryl)oxy)methyl Pivalate (**9g**)

Alcohol **8g** (50 mg, 0.057 mmol), acetic anhydride (9 mg, 0.085 mmol), and triethylamine (11 mg, 0.11 mmol) were dissolved in freshly distilled methylene chloride (5 mL), and the resultant reaction mixture was allowed to react overnight at room temperature. The reaction mixture was diluted by the addition of water (5 mL), quenched with sodium bicarbonate (2 mL), and extracted with methylene chloride (3 × 5 mL). The combined extracts were dried (Na_2_SO_4_) and filtered through Celite, and the filtrate was concentrated in vacuo. The residue was purified by column chromatography (silica gel, 100% hexane −50% EtOAc in hexane), and the resulting product **9g** was isolated as an oil in 98% yield (54 mg): ^1^H NMR (500 MHz, CDCl_3_): δ 7.62 (d, *J* = 8.9 Hz, 2H), 7.35 (d, *J* = 8.7 Hz, 2H), 5.57 (dd, *J*_PH_ = 13.8, *J*_HH_ = 5.1 Hz, 1H), 5.50 (dd, *J*_PH_ = 12.2, *J*_HH_ = 5.0 Hz, 1H), 5.31 (td, *J* = 7.2, 1.4 Hz, 1H), 4.45 (s, 2H), 2.51–2.40 (m, 2H), 2.10–2.00 (m, 5H), 1.68 (s, 3H), 1.16 (s, 9H); ^13^C NMR (126 MHz, CDCl_3_): δ 176.9, 170.8, 152.6 (d, *J*_PC_ = 10.1 Hz), 132.2, 127.4 (q, *J*_CF_ = 37.8 Hz), 127.2 (q, *J*_CF_ = 3.8 Hz, 2C), 126.4 (d, *J*_PC_ = 16.4 Hz), 125.9 (q, *J*_CF_ = 272.2 Hz), 120.9 (d, *J*_PC_ = 3.8 Hz, 2C), 81.8 (d, *J*_PC_ = 5.0 Hz), 69.4, 38.7, 26.7 (3C), 26.1 (d, *J*_PC_ = 138.6 Hz), 20.9, 20.5 (d, *J*_PC_ = 5.0 Hz), 13.9; ^31^P NMR (203 MHz, CDCl_3_) 29.4 ppm; ^19^F NMR (471 MHz, CDCl_3_) −62.2 ppm. HRMS (ESI^+^) *m*/*z*: calcd for C_21_H_28_F_3_NaO_7_P (M + Na)^+^, 503.1422; found, 503.1407. HPLC purity >97% (*t*_R_ = 3.23).

##### (*E*)-(((5-Hydroxy-4-methylpent-3-en-1-yl)(2 (trifluoromethyl)phenoxy)phosphoryl)oxy)methyl Pivalate (**8h**)

A round-bottom flask was charged with olefin **7h** (330 mg, 0.78 mmol), selenium dioxide (69 mg, 0.62 mmol), and 4-hydroxybenzoic acid (15 mg, 0.11 mmol) in dichloromethane (10 mL). At 0 °C, *tert*-butyl hydroperoxide (70% wt in H_2_O, 0.431 mL, 3.1 mmol) was added slowly to the stirred reaction mixture. The resulting reaction mixture was stirred at 0 °C and was allowed to react for 3 days. The reaction mixture was diluted by the addition of water (5 mL), quenched with Na_2_SO_3_ (5 mL), and extracted with dichloromethane (3 × 10 mL). The combined extracts were dried (Na_2_SO_4_) and filtered through Celite, and the filtrate was concentrated in vacuo. The resulting oil was purified by column chromatography (silica, 100% ether −3% MeOH in ether) to give the desired product **8h** as an oil in 17% yield (60 mg): ^1^H NMR (400 MHz, CDCl_3_): δ 7.62–7.56 (m, 2H), 7.48–7.45 (m, 1H), 7.21–7.17 (m, 1H), 5.70 (dd, *J*_PH_ = 11.3 Hz, *J*_HH_ = 5.0 Hz, 1H), 5.60 (dd, *J*_PH_ = 13.6 Hz, *J*_HH_ = 4.9 Hz, 1H), 5.36 (td, *J* = 7.0, 1.4 Hz, 1H), 3.93 (s, 2H), 2.45–2.36 (m, 2H), 2.04–1.95 (m, 2H), 1.60 (s, 3H), 1.09 (s, 9H); ^13^C NMR (126 MHz, CDCl_3_): δ 176.7, 148.0, 136.6, 133.3, 127.1 (q, *J*_CF_ = 5.0 Hz), 124.4, 122.9 (d, *J*_PC_ = 17.6 Hz), 122.8 (q, *J*_CF_ = 272.2 Hz), 121.7 (2C), 81.6 (d, *J*_PC_ = 6.3 Hz), 68.1, 38.5, 26.6 (3C), 26.4 (d, *J*_PC_ = 139.9 Hz), 20.7 (d, *J*_PC_ = 5.0 Hz), 13.4; ^31^P NMR (162 MHz, CDCl_3_) 29.6 ppm; ^19^F NMR (471 MHz, CDCl_3_) −61.6 ppm. HRMS (ESI^+^) *m*/*z*: calcd for C_19_H_27_O_6_F_3_P (M + H)^+^, 439.1497; found, 439.1485. HPLC purity >95% (*t*_R_ = 3.30).

##### (*E*)-(((5-Acetoxy-4-methylpent-3-en-1-yl)(2-trifluoromethylphenoxy)phosphoryl)oxy)methyl Pivalate (**9h**)

Alcohol **8h** (15 mg, 0.034 mmol), acetic anhydride (5 mg, 0.05 mmol), and triethylamine (7 mg, 0.07 mmol) were dissolved in freshly distilled methylene chloride (3 mL), and the resultant reaction mixture was allowed to react overnight at room temperature. The reaction mixture was diluted by addition of water (3 mL), quenched with sodium bicarbonate (2 mL), and extracted with methylene chloride (3 × 4 mL). The combined extracts were dried (Na_2_SO_4_) and filtered through Celite, and the filtrate was concentrated in vacuo. The residue was purified by column chromatography (silica gel, 100% hexane −50% EtOAc in hexane), and the resulting product **9h** was isolated as an oil in 98% yield (16 mg): ^1^H NMR (400 MHz, CDCl_3_): δ 7.64–7.56 (m, 2H), 7.48–7.44 (m, 1H), 7.21–7.18 (m, 1H), 5.70 (dd, *J*_PH_ = 11.3 Hz, *J*_HH_ = 5.0 Hz, 1H), 5.61 (dd, *J*_PH_ = 13.6 Hz, *J*_HH_ = 4.9 Hz, 1H), 5.39 (td, *J* = 7.0, 1.4 Hz, 1H), 4.37 (s, 2H), 2.45–2.34 (m, 2H), 2.01 (s, 3H), 2.04–1.95 (m, 2H), 1.60 (s, 3H), 1.08 (s, 9H); ^13^C NMR (126 MHz, CDCl_3_): δ 176.8, 170.9, 148.0, 133.5, 132.1, 127.3 (q, *J*_CF_ = 5.04 Hz), 126.6, 124.5, 122.8 (q, *J*_CF_ = 272.2 Hz), 121.2 (2C), 81.8 (d, *J*_PC_ = 6.1 Hz), 69.5, 38.7, 26.8 (3C), 26.3 (d, *J*_PC_ = 140.4 Hz), 21.0, 20.3 (d, *J*_PC_ = 5.0 Hz), 13.9; ^31^P NMR (162 MHz, CDCl_3_) 28.8 ppm; ^19^F NMR (471 MHz, CDCl_3_) −61.6 ppm. HRMS (ESI^+^) *m*/*z*: calcd for C_21_H_29_O_7_F_3_P (M + H)^+^, 481.1603; found, 481.1591. HPLC purity >95% (*t*_R_ = 3.36).

##### (*E*)-(((5-Hydroxy-4-methylpent-3-en-1-yl)(4-isopropylphenoxy)phosphoryl)oxy)methyl Pivalate (**8i**)

A round-bottom flask was charged with olefin **7i** (740 mg, 1.8 mmol), selenium dioxide (165 mg, 1.4 mmol), and 4-hydroxybenzoic acid (36 mg, 0.26 mmol) in dichloromethane (10 mL). At 0 °C, *tert*-butyl hydroperoxide (70% wt in H_2_O, 1.0 mL, 7.45 mmol) was added slowly to the stirred reaction mixture. The resulting reaction mixture was stirred at 0 °C and was allowed to react for 3 days. The reaction mixture was diluted by the addition of water (5 mL), quenched with Na_2_SO_3_ (5 mL), and extracted with dichloromethane (3 × 10 mL). The combined extracts were dried (Na_2_SO_4_) and filtered through Celite, and the filtrate was concentrated in vacuo. The resulting oil was purified by column chromatography (silica, 100% ether −3% MeOH in ether) to give the desired product **8i** as an oil in 20% yield (150 mg): ^1^H NMR (500 MHz, CDCl_3_): δ 7.17 (d, *J* = 8.6 Hz, 2H), 7.10 (d, *J* = 8.6 Hz, 2H), 5.71 (dd, *J*_PH_ = 11.3 Hz, *J*_HH_ = 5.0 Hz, 1H), 5.64 (dd, *J*_PH_ = 13.6 Hz, *J*_HH_ = 4.9 Hz, 1H), 5.41 (td, *J* = 7.0, 1.4 Hz, 1H), 3.98 (s, 2H), 2.92–2.84 (m, 1H), 2.48–2.40 (m, 2H), 2.10–1.98 (m, 2H), 1.68 (s, 3H), 1.21 (d, *J* = 5 Hz, 6H), 1.15 (s, 9H); ^13^C NMR (126 MHz, CDCl_3_): δ 176.9, 147.7 (d, *J*_PC_ = 9.1 Hz), 145.6, 136.4, 127.5 (2C), 122.9 (d, *J*_PC_ = 15.3 Hz), 120.1 (2C), 81.5 (d, *J*_PC_ = 6.1 Hz), 68.0, 38.5, 33.3, 26.6 (3C), 25.9 (d, *J*_PC_ = 139.2 Hz), 23.8 (2C), 20.3 (d, *J*_PC_ = 5.0 Hz), 13.5; ^31^P NMR (203 MHz, CDCl_3_) 29.2 ppm. HRMS (ESI^+^) *m*/*z*: calcd for C_21_H_34_O_6_P (M + H)^+^, 413.2093; found, 413.2081. HPLC purity >95% (*t*_R_ = 3.30).

##### (*E*)-(((5-Acetoxy-4-methylpent-3-en-1-yl)(4-isopropylphenoxy)phosphoryl)oxy)methyl Pivalate (**9i**)

Alcohol **8i** (40 mg, 0.096 mmol), acetic anhydride (15 mg, 0.14 mmol), and triethylamine (19 mg, 0.18 mmol) were dissolved in freshly distilled methylene chloride (4 mL), and the resultant reaction mixture was allowed to react overnight at room temperature. The reaction mixture was diluted by the addition of water (5 mL), quenched with sodium bicarbonate (2 mL), and extracted with methylene chloride (3 × 5 mL). The combined extracts were dried (Na_2_SO_4_) and filtered through Celite, and the filtrate was concentrated in vacuo. The residue was purified by column chromatography (silica gel, 100% hexane −50% EtOAc in hexane), and the resulting product **9i** was isolated as an oil in 94% yield (40 mg): ^1^H NMR (500 MHz, CDCl_3_): δ 7.17 (d, *J* = 8.6 Hz, 2H), 7.11 (d, *J* = 8.6 Hz, 2H), 5.72 (dd, *J*_PH_ = 11.3 Hz, *J*_HH_ = 5.0 Hz, 1H), 5.65 (dd, *J*_PH_ = 13.6, *J*_HH_ = 4.9 Hz, 1H), 5.46 (td, *J* = 7.0, 1.4 Hz, 1H), 4.45 (s, 2H), 2.94–2.85 (m, 1H), 2.48–2.40 (m, 2H), 2.06 (s, 3H), 2.04–1.97 (m, 2H), 1.67 (s, 3H), 1.21 (d, *J* = 5 Hz, 6H), 1.17 (s, 9H); ^13^C NMR (126 MHz, CDCl_3_): δ 176.9, 170.8, 147.8 (d, *J*_PC_ = 9.2 Hz), 145.8, 131.9, 127.7 (2C), 126.9 (d, *J*_PC_ = 17.5 Hz), 120.2 (2C), 81.8 (d, *J*_PC_ = 6.0 Hz), 69.6, 38.7, 33.5, 26.8 (3C), 25.9 (d, *J*_PC_ = 139.6 Hz), 24.0 (2C), 20.9, 20.6 (d, *J*_PC_ = 4.8 Hz), 13.9; ^31^P NMR (203 MHz, CDCl_3_) 28.9 ppm. HRMS (ESI^+^) *m*/*z*: calcd for C_23_H_36_O_7_P (M + H)^+^, 455.2199; found, 455.2187. HPLC purity >95% (*t*_R_ = 3.30).

##### (*E*)-(((5-Hydroxy-4-methylpent-3-en-1-yl)(2-isopropylphenoxy)phosphoryl)oxy)methyl Pivalate (**8j**)

A round-bottom flask was charged with olefin **7j** (420 mg, 1.0 mmol), selenium dioxide (94 mg, 0.85 mmol), and 4-hydroxybenzoic acid (20 mg, 0.14 mmol) in dichloromethane (10 mL). At 0 °C, *tert*-butyl hydroperoxide (70% wt in H_2_O, 0.584 mL, 4.2 mmol) was added slowly to the stirred reaction mixture. The resulting reaction mixture was stirred at 0 °C and was allowed to react for 3 days. The reaction mixture was diluted by the addition of water (5 mL), quenched with Na_2_SO_3_ (5 mL), and extracted with dichloromethane (3 × 10 mL). The combined extracts were dried (Na_2_SO_4_) and filtered through Celite, and the filtrate was concentrated in vacuo. The resulting oil was purified by column chromatography (silica, 100% ether −3% MeOH in ether) to give the desired product **8j** as an oil in 14% yield (60 mg): ^1^H NMR (400 MHz, CDCl_3_): δ 7.30–7.19 (m, 2H), 7.10–7.04 (m, 2H), 5.64 (dd, *J*_PH_ = 11.3 Hz, *J*_HH_ = 5.0 Hz, 1H), 5.54 (dd, *J*_PH_ = 13.6 Hz, *J*_HH_ = 4.9 Hz, 1H), 5.37 (td, *J* = 7.0, 1.4 Hz, 1H), 3.93 (s, 2H), 3.25–3.18 (m, 1H), 2.45–2.35 (m, 2H), 2.02–1.93 (m, 2H), 1.60 (s, 3H), 1.16 (d, *J* = 5.0 Hz, 6H), 1.09 (s, 9H); ^13^C NMR (101 MHz, CDCl_3_): δ 177.1, 147.5 (d, *J*_PC_ = 11.3 Hz), 139.3, 136.6, 126.8 (2C), 125.3, 123.2 (d, *J*_PC_ = 16.2 Hz), 120.2, 81.8 (d, *J*_PC_ = 6.1 Hz), 68.2, 38.7, 26.9, 26.8 (3C), 26.6 (d, *J*_PC_ = 141.4 Hz), 22.9 (2C), 20.5 (d, *J*_PC_ = 5.0 Hz), 13.6; ^31^P NMR (162 MHz, CDCl_3_) 28.3 ppm. HRMS (ESI^+^) *m*/*z*: calcd for C_21_H_34_O_6_P (M + H)^+^, 413.2093; found, 413.2080. HPLC purity >95% (*t*_R_ = 3.30).

##### (*E*)-(((5-Acetoxy-4-methylpent-3-en-1-yl)(2-isopropylphenoxy)phosphoryl)oxy)methyl Pivalate (**9j**)

Alcohol **8j** (16 mg, 0.038 mmol), acetic anhydride (6 mg, 0.058 mmol), and triethylamine (8 mg, 0.077 mmol) were dissolved in freshly distilled methylene chloride (4 mL), and the resultant reaction mixture was allowed to react overnight at room temperature. The reaction mixture was diluted by the addition of water (5 mL), quenched with sodium bicarbonate (2 mL), and extracted with methylene chloride (3 × 5 mL). The combined extracts were dried (Na_2_SO_4_) and filtered through Celite, and the filtrate was concentrated in vacuo. The residue was purified by column chromatography (silica gel, 100% hexane −50% EtOAc in hexane), and the resulting product **9j** was isolated as an oil in 91% yield (16 mg): ^1^H NMR (400 MHz, CDCl_3_): δ 7.30–7.19 (m, 2H), 7.10–7.05 (m, 2H), 5.64 (dd, *J*_PH_ = 11.3 Hz, *J*_HH_ = 5.0 Hz, 1H), 5.53 (dd, *J*_PH_ = 13.6 Hz, *J*_HH_ = 4.9 Hz, 1H), 5.39 (td, *J* = 7.0, 1.4 Hz, 1H), 4.37 (s, 2H), 3.25–3.17 (m, 1H), 2.46–2.33 (m, 2H), 1.99 (s, 3H), 1.98–1.92 (m, 2H), 1.60 (s, 3H), 1.14 (d, *J* = 5 Hz, 6H), 1.08 (s, 9H); ^13^C NMR (101 MHz, CDCl_3_): δ 175.9, 169.8, 146.4 (d, *J*_PC_ = 9.1 Hz), 138.2, 130.9, 125.9 (2C), 125.8 (d, *J*_PC_ = 19.1 Hz), 124.3, 119.1, 80.9 (d, *J*_PC_ = 6.1 Hz), 68.5, 37.6, 25.9, 25.8 (3C), 25.3 (d, *J*_PC_ = 140.4 Hz), 21.9 (2C), 19.9, 19.6 (d, *J*_PC_ = 4.0 Hz), 12.9; ^31^P NMR (162 MHz, CDCl_3_) 28.0 ppm. HRMS (ESI^+^) *m*/*z*: calcd for C_23_H_36_O_7_P (M + H)^+^, 455.2199; found, 455.2187. HPLC purity >95% (*t*_R_ = 3.32).

##### (*E*)-(((2,6-Diisopropylphenoxy)(5-hydroxy-4-methylpent-3-en-1-yl)phosphoryl)oxy)methyl Pivalate (**8k**)

The round-bottom flask was charged with olefin **7k** (530 mg, 1.2 mmol), selenium dioxide (107 mg, 0.96 mmol), and 4-hydroxybenzoic acid (23 mg, 0.16 mmol) in dichloromethane (10 mL). At 0 °C, *tert*-butyl hydroperoxide (70% wt in H_2_O, 0.666 mL, 4.8 mmol) was added slowly to the stirred solution. The resulting reaction mixture was stirred at 0 °C and was allowed to react for 5 days. The reaction mixture was diluted by the addition of water (5 mL), quenched with Na_2_SO_3_ (5 mL), and extracted with dichloromethane (3 × 10 mL). The combined extracts were dried (Na_2_SO_4_) and concentrated in vacuo. The resulting oil was dissolved in THF (5 mL) and allowed to react with sodium borohydride (45 mg, 1.2 mmol) for 1 h monitored by thin layer chromatography. The reaction mixture was quenched by the addition of saturated ammonium chloride (5 mL) and extracted with diethyl ether (3 × 10 mL). The combined extracts were dried (Na_2_SO_4_) and filtered through Celite, and the filtrate was concentrated in vacuo. The resulting oil was purified by column chromatography (silica, 100% ether −3% MeOH in ether) to give the desired product **8k** as an oil in 16% yield (90 mg): ^1^H NMR (500 MHz, CDCl_3_): δ 7.12 (s, 3H), 5.63 (dd, *J*_PH_ = 11.3, *J*_HH_ = 5.0 Hz, 1H), 5.51 (dd, *J*_PH_ = 13.6, *J*_HH_ = 4.9 Hz, 1H), 5.46 (td, *J* = 7.0, 1.4 Hz, 1H), 4.01 (s, 2H), 3.48–3.41 (m, 2H), 2.55–2.45 (m, 2H), 2.13–2.01 (m, 2H), 1.69 (s, 3H), 1.24–1.19 (m, 12H), 1.14 (s, 9H); ^13^C NMR (126 MHz, CDCl_3_): δ 176.8, 144.4 (d, *J*_PC_ = 12.6 Hz), 140.5 (2C), 136.4, 125.7, 124.1 (2C), 123.2 (d, *J*_PC_ = 17.6 Hz), 82.0 (d, *J*_PC_ = 5.0 Hz), 68.1, 38.5, 27.0 (2C), 26.8 (d, *J*_PC_ = 139.8 Hz), 26.6 (3C), 23.4 (2C), 23.3 (2C), 20.5 (d, *J*_PC_ = 5.0 Hz), 13.5; ^31^P NMR (203 MHz, CDCl_3_) 27.6 ppm. HRMS (ESI^+^) *m*/*z*: calcd for C_24_H_39_NaO_6_P (M + Na)^+^, 477.2382; found, 477.2375. HPLC purity >95% (*t*_R_ = 3.26).

##### (*E*)-(((5-Acetoxy-4-methylpent-3-en-1-yl)(2,6-diisopropylphenoxy)phosphoryl)oxy)methyl Pivalate (**9k**)

Alcohol **8k** (30 mg, 0.067 mmol), acetic anhydride (10 mg, 0.097 mmol), and triethylamine (13 mg, 0.13 mmol) were dissolved in freshly distilled methylene chloride (4 mL), and the resultant reaction mixture was allowed to react overnight at room temperature. The reaction mixture was diluted by the addition of water (5 mL), quenched with sodium bicarbonate (2 mL), and extracted with methylene chloride (3 × 5 mL). The combined extracts were dried (Na_2_SO_4_) and filtered through Celite, and the filtrate was concentrated in vacuo. The residue was purified by column chromatography (silica gel, 100% hexane −50% EtOAc in hexane), and the resulting product **9k** was isolated as an oil in 97% yield (32 mg). ^1^H NMR (500 MHz, CDCl_3_): δ 7.13 (s, 3H), 5.64 (dd, *J*_PH_ = 11.3, *J*_HH_ = 5.0 Hz, 1H), 5.54 (dd, *J*_PH_ = 13.6, *J*_HH_ = 4.9 Hz, 1H), 5.49 (td, *J* = 7.2 Hz, 1H), 4.46 (s, 2H), 3.50–3.41 (m, 2H), 2.46–2.55 (m, 2H), 2.11–2.06 (m, 2H), 2.07 (s, 3H), 1.7 (s, 3H), 1.28–1.20 (m, 12H), 1.15 (s, 9H); ^13^C NMR (126 MHz, CDCl_3_): δ 176.8, 170.8, 144.5 (d, *J*_PC_ = 11.3 Hz), 140.7, 140.6, 131.9, 127.1 (d, *J*_PC_ = 18.9 Hz), 125.8, 124.3 (2C), 82.2 (d, *J*_PC_ = 5.0 Hz), 69.5, 38.6, 27.2 (2C), 26.8 (3C), 26.1 (d, *J*_PC_ = 142.4 Hz), 23.6 (2C), 23.4 (2C), 20.9, 20.8 (d, *J*_PC_ = 5.04 Hz), 13.9; ^31^P NMR (203 MHz, CDCl_3_) 27.2 ppm. HRMS (ESI^+^) *m*/*z*: calcd for C_26_H_41_NaO_7_P (M + Na)^+^, 519.2488; found, 519.2476. HPLC purity >95% (*t*_R_ = 3.29).

##### (*E*)-(((5-Hydroxy-4-methylpent-3-en-1-yl)(phenoxy)phosphoryl)oxy)methyl Benzoate (**11x**)

A round-bottom flask was charged with olefin **10x** (590 mg, 1.57 mmol), selenium dioxide (139 mg, 1.25 mmol), and 4-hydroxybenzoic acid (30 mg, 0.21 mmol) in dichloromethane (10 mL). At 0 °C, *tert*-butyl hydroperoxide (70% wt in H_2_O, 0.869 mL, 6.3 mmol) was added slowly to the stirred reaction mixture and it was allowed to react at 0 °C for 2 days. The reaction mixture was diluted by the addition of water (5 mL), quenched with Na_2_SO_3_ (5 mL), and extracted with dichloromethane (3 × 10 mL). The combined extracts were dried (Na_2_SO_4_) and filtered through Celite, and the filtrate was concentrated in vacuo. The resulting oil was purified by column chromatography (silica, 100% ether −3% MeOH in ether) to give the desired product **11x** as an oil in 16% yield (100 mg): ^1^H NMR (500 MHz, CDCl_3_): δ 7.98–7.94 (m, 2H), 7.63–7.57 (m, 1H), 7.44–7.40 (m, 2H), 7.26–7.17 (m, 4H), 7.10–7.06 (m, 1H), 5.94 (dd, *J*_PH_ = 14.0 Hz, *J*_HH_ = 5.2 Hz, 1H), 5.85 (dd, *J*_PH_ = 13.0 Hz, *J*_HH_ = 5.2 Hz, 1H), 5.40 (td, *J* = 7.1, 1.4 Hz, 1H), 3.95 (s, 2H), 2.48–2.40 (m, 2H), 2.09–2.01 (m, 2H), 1.60 (s, 3H); ^13^C NMR (126 MHz, CDCl_3_): δ 165.0, 149.9 (d, *J*_PC_ = 8.8 Hz), 136.7, 133.8, 130.0 (2C), 129.7 (2C), 128.6, 128.5 (2C), 125.2, 123.1 (d, *J*_PC_ = 16.4 Hz), 120.6 (2C), 82.0 (d, *J*_PC_ = 6.3 Hz), 68.2, 26.2 (d, *J*_PC_ = 138.6 Hz), 20.5 (d, *J*_PC_ = 5.0 Hz), 13.6; ^31^P NMR (203 MHz, CDCl_3_) 29.7 ppm. HRMS (ESI^+^) *m*/*z*: calcd for C_20_H_24_O_6_P (M + H)^+^, 391.1311; found, 391.1300. HPLC purity 98% (*t*_R_ = 3.41).

##### (*E*)-(((5-Acetoxy-4-methylpent-3-en-1-yl)(phenoxy)phosphoryl)oxy)methyl Benzoate (**12x**)

Alcohol **11x** (20 mg, 0.051 mmol), acetic anhydride (8 mg, 0.08 mmol), and triethylamine (9.8 mg, 0.096 mmol) were dissolved in freshly distilled methylene chloride (3 mL), and the resultant reaction mixture was allowed to react overnight at room temperature. The reaction mixture was diluted by the addition of water (3 mL), quenched with sodium bicarbonate (2 mL), and extracted with methylene chloride (3 × 4 mL). The combined extracts were dried (Na_2_SO_4_) and filtered through Celite, and the filtrate was concentrated in vacuo. The residue was purified by column chromatography (silica gel, 100% hexane −50% EtOAc in hexane), and the resulting product **12x** was isolated as an oil in 90% yield (20 mg): ^1^H NMR (500 MHz, CDCl_3_): δ 7.98–7.95 (m, 2H), 7.61–7.57 (m, 1H), 7.44–7.41 (m, 2H), 7.27–7.18 (m, 4H), 7.10–7.07 (m, 1H), 5.95 (dd, *J*_PH_ = 14.6 Hz, *J*_HH_ = 5.2 Hz, 1H), 5.86 (dd, *J*_PH_ = 12.5 Hz, *J*_HH_ = 5.2 Hz, 1H), 5.44 (td, *J* = 7.1, 1.4 Hz, 1H), 4.41 (s, 2H), 2.49–2.42 (m, 2H), 2.08–2.01 (m, 2H), 2.05 (s, 3H), 1.60 (s, 3H); ^13^C NMR (126 MHz, CDCl_3_): δ 170.8, 164.9, 149.8 (d, *J*_PC_ = 10.0 Hz), 133.8, 131.9, 130.0 (2C), 129.7 (2C), 128.7, 128.5 (2C), 126.8 (d, *J*_PC_ = 17.6 Hz), 125.1, 120.5 (2C), 82.0 (d, *J*_PC_ = 6.3 Hz), 69.5, 26.0 (d, *J*_PC_ = 141.1 Hz), 20.9, 20.6 (d, *J*_PC_ = 5.0 Hz), 13.9; ^31^P NMR (203 MHz, CDCl_3_) 29.4 ppm. HRMS (ESI^+^) *m*/*z*: calcd for C_22_H_25_O_7_P (M + H)^+^, 433.1416; found, 433.1407. HPLC purity 98% (*t*_R_ = 3.39).

##### (*E*)-(((5-Hydroxy-4-methylpent-3-en-1-yl)(phenoxy)phosphoryl)oxy)methyl Isobutyrate (**11y**)

A round-bottom flask was charged with olefin **10y** (110 mg, 0.320 mmol), selenium dioxide (28 mg, 0.25 mmol), and 4-hydroxybenzoic acid (6 mg, 0.045 mmol) in dichloromethane (10 mL). At 0 °C, *tert*-butyl hydroperoxide (70% wt in H_2_O, 0.178 mL, 1.28 mmol) was added slowly to the stirred reaction mixture and it was allowed to react at 0 °C for 2 days. The reaction mixture was diluted by the addition of water (5 mL), quenched with Na_2_SO_3_ (5 mL), and extracted with dichloromethane (3 × 10 mL). The combined extracts were dried (Na_2_SO_4_) and filtered through Celite, and the filtrate was concentrated in vacuo. The resulting oil was purified by column chromatography (silica, 100% ether −3% MeOH in ether) to give the desired product **11y** as an oil in 32% yield (38 mg): ^1^H NMR (400 MHz, CDCl_3_): δ 7.30–7.24 (m, 2H), 7.15–7.09 (m, 3H), 5.64 (dd, *J*_PH_ = 14.0 Hz, *J*_HH_ = 5.1 Hz, 1H), 5.56 (dd, *J*_PH_ = 12.9 Hz, *J*_HH_ = 5.1 Hz, 1H), 5.36 (td, *J* = 7.1, 1.4 Hz, 1H), 3.92 (s, 2H), 2.44–2.33 (m, 3H), 1.99–1.91 (m, 2H), 1.60 (s, 3H), 1.08–1.05 (m, 6H); ^13^C NMR (101 MHz, CDCl_3_): δ 175.6, 150.0 (d, *J*_PC_ = 9.1 Hz), 136.7, 129.8 (2C), 125.2, 123.1 (d, *J*_PC_ = 15.1 Hz), 120.5 (2C), 81.5 (d, *J*_PC_ = 7.0 Hz), 68.2, 33.7, 26.1 (d, *J*_PC_ = 139.4 Hz), 20.5 (d, *J*_PC_ = 5.0 Hz), 18.5 (2C), 13.7; ^31^P NMR (162 MHz, CDCl_3_) 29.0 ppm. HRMS (ESI^+^) *m*/*z*: calcd for C_17_H_26_O_6_P (M + H)^+^, 357.1467; found, 357.1457. HPLC purity >95% (*t*_R_ = 3.38).

##### (*E*)-(((5-Acetoxy-4-methylpent-3-en-1-yl)(phenoxy)phosphoryl)oxy)methyl Isobutyrate (**12y**)

Alcohol **11y** (16 mg, 0.045 mmol), acetic anhydride (7 mg, 0.068 mmol), and triethylamine (9 mg, 0.089 mmol) were dissolved in freshly distilled methylene chloride (3 mL), and the resultant reaction mixture was allowed to react overnight at room temperature. The reaction mixture was diluted by the addition of water (3 mL), quenched with sodium bicarbonate (2 mL), and extracted with methylene chloride (3 × 4 mL). The combined extracts were dried (Na_2_SO_4_) and filtered through Celite, and the filtrate was concentrated in vacuo. The residue was purified by column chromatography (silica gel, 100% hexane −50% EtOAc in hexane), and the resulting product **12y** was isolated as an oil in 95% yield (17 mg): ^1^H NMR (400 MHz, CDCl_3_): δ 7.30–7.24 (m, 2H), 7.15–7.09 (m, 3H), 5.64 (dd, *J*_PH_ = 14.0 Hz, *J*_HH_ = 5.1 Hz, 1H), 5.56 (dd, *J*_PH_ = 12.9 Hz, *J*_HH_ = 5.1 Hz, 1H), 5.39 (td, *J* = 7.1, 1.4 Hz, 1H), 4.38 (s, 2H), 2.45–2.33 (m, 3H), 2.00 (s, 3H), 1.98–1.90 (m, 2H), 1.60 (s, 3H), 1.08–1.05 (m, 6H); ^13^C NMR (101 MHz, CDCl_3_): δ 175.5, 170.8, 149.9 (d, *J*_PC_ = 9.1 Hz), 131.9, 129.8 (2C), 126.8 (d, *J*_PC_ = 18.2 Hz), 125.2, 120.5 (2C), 81.5 (d, *J*_PC_ = 6.1 Hz), 69.5, 33.7, 25.9 (d, *J*_PC_ = 140.4 Hz), 20.9, 20.5 (d, *J*_PC_ = 5.0 Hz), 18.5 (2C), 13.9; ^31^P NMR (162 MHz, CDCl_3_) 28.7 ppm. HRMS (ESI^+^) *m*/*z*: calcd for C_19_H_28_O_7_P (M + H)^+^, 399.1573; found, 399.1562. HPLC purity >95% (*t*_R_ = 3.37).

##### (*E*)-(((5-Hydroxy-4-methylpent-3-en-1-yl)(phenoxy)phosphoryl)oxy)methyl 2-Cyclohexylacetate (**11z**)

A round-bottom flask was charged with olefin **10z** (415 mg, 1.05 mmol), selenium dioxide (93 mg, 0.83 mmol), and 4-hydroxybenzoic acid (20 mg, 0.144 mmol) in dichloromethane (10 mL). At 0 °C, *tert*-butyl hydroperoxide (70% wt in H_2_O, 0.592 mL, 4.2 mmol) was added slowly to the stirred reaction mixture and it was allowed to react at 0 °C for 2 days. The reaction mixture was diluted by the addition of water (5 mL), quenched with Na_2_SO_3_ (5 mL), and extracted with dichloromethane (3 × 10 mL). The combined extracts were dried (Na_2_SO_4_) and filtered through Celite, and the filtrate was concentrated in vacuo. The resulting oil was purified by column chromatography (silica, 100% ether −3% MeOH in ether) to give the desired product **11z** as an oil in 17% yield (70 mg): ^1^H NMR (400 MHz, CDCl_3_): δ 7.35–7.31 (m, 2H), 7.21–7.15 (m, 3H), 5.64 (dd, *J*_PH_ = 14.2 Hz, *J*_HH_ = 5.2 Hz, 1H), 5.56 (dd, *J*_PH_ = 12.1 Hz, *J*_HH_ = 5.2 Hz, 1H), 5.41 (td, *J* = 7.1, 1.4 Hz, 1H), 3.98 (s, 2H), 2.49–2.39 (m, 2H), 2.11 (d, *J* = 7.0 Hz, 2H), 2.12–1.96 (m, 2H), 1.60 (s, 3H), 1.72–1.60 (m, 6H), 1.27–1.09 (m, 3H), 0.96–0.86 (m, 2H); ^13^C NMR (101 MHz, CDCl_3_): δ 171.6, 150.0 (d, *J*_PC_ = 9.1 Hz), 136.7, 129.8 (2C), 125.2, 123.1 (d, *J*_PC_ = 16.2 Hz), 120.5 (2C), 81.3 (d, *J*_PC_ = 7.1 Hz), 68.2, 41.6, 34.5, 32.9 (2C), 26.1 (d, *J*_PC_ = 140.4 Hz), 26.0, 25.9 (2C), 20.5 (d, *J*_PC_ = 5.0 Hz), 13.7; ^31^P NMR (162 MHz, CDCl_3_) 29.0 ppm. HRMS (ESI^+^) *m*/*z*: calcd for C_21_H_32_O_6_P (M + H)^+^, 411.1937; found, 411.1925. HPLC purity >95% (*t*_R_ = 3.34).

##### (*E*)-(((5-Acetoxy-4-methylpent-3-en-1-yl)(phenoxy)phosphoryl)oxy)methyl 2-Cyclohexylacetate (**12z**)

Alcohol **11z** (35 mg, 0.085 mmol), acetic anhydride (13 mg, 0.13 mmol), and triethylamine (17 mg, 0.17 mmol) were dissolved in freshly distilled methylene chloride (4 mL), and the resultant reaction mixture was allowed to react overnight at room temperature. The reaction mixture was diluted by the addition of water (3 mL), quenched with sodium bicarbonate (2 mL), and extracted with methylene chloride (3 × 4 mL). The combined extracts were dried (Na_2_SO_4_) and filtered through Celite, and the filtrate was concentrated in vacuo. The residue was purified by column chromatography (silica gel, 100% hexane −50% EtOAc in hexane), and the resulting product **12z** was isolated as an oil in 96% yield (37 mg): ^1^H NMR (400 MHz, CDCl_3_): δ 7.35–7.31 (m, 2H), 7.21–7.17 (m, 3H), 5.70 (dd, *J*_PH_ = 14.3 Hz, *J*_HH_ = 5.2 Hz, 1H), 5.59 (dd, *J*_PH_ = 12.1 Hz, *J*_HH_ = 5.2 Hz, 1H), 5.45 (td, *J* = 7.1, 1.4 Hz, 1H), 4.44 (s, 2H), 2.49–2.40 (m, 2H), 2.11 (d, *J* = 7.0 Hz, 2H), 2.06 (s, 3H), 2.08–1.96 (m, 2H), 1.66 (s, 3H), 1.74–1.61 (m, 6H), 1.27–1.07 (m, 3H), 0.95–0.87 (m, 2H); ^13^C NMR (101 MHz, CDCl_3_): δ 171.5, 170.9, 150.0 (d, *J*_PC_ = 9.1 Hz), 131.9, 129.8 (2C), 126.8 (d, *J*_PC_ = 17.2 Hz), 125.2, 120.5 (2C), 81.3 (d, *J*_PC_ = 7.1 Hz), 69.5, 41.6, 34.5, 32.9 (2C), 26.1 (d, *J*_PC_ = 140.4 Hz), 26.0, 25.9 (2C), 21.0, 20.6 (d, *J*_PC_ = 4.0 Hz), 14.0; ^31^P NMR (162 MHz, CDCl_3_) 28.7 ppm. HRMS (ESI^+^) *m*/*z*: calcd for C_23_H_34_O_7_P (M + H)^+^, 453.2042; found, 453.2029. HPLC purity >95% (*t*_R_ = 3.33).

### Biological Assays

#### Materials and Supplies

Buffy coat was obtained from Research Blood Components (Boston, MA) and used to purify peripheral blood mononuclear cells (PBMCs) by density gradient centrifugation. The FITC-conjugated anti-γδ-TCR (5A6.E91) antibody was purchased from Fisher (Waltham, MA). The PE-conjugated anti-CD3 (UCHT1) antibody was purchased from Biolegend (San Diego, CA). HMBPP was purchased from Cayman Chemical (Ann Arbor, MI). K562 cells were from Sigma-Aldrich (St. Louis, MO). The TCRγ/δ+ T Cell Isolation Kit was from Miltenyi (Bergisch Gladbach, Germany). The interferon γ enzyme-linked immunosorbent assay kit was purchased from Biolegend (San Diego, CA). Pooled human plasma was purchased from Innovative Research (Novi, MI).

#### Vγ9Vδ2 T Cell proliferation

The novel compounds were evaluated for their ability to promote growth of human Vγ9Vδ2 T cells from peripheral blood.^15,27^ PBMCs were stimulated for 3 days with test compounds at various concentrations, washed, and allowed to grow for 11 additional days in the presence of IL-2. The percentage of Vγ9Vδ2 T cells was determined by flow cytometry staining for CD3 and γδ-TCR after subtraction of nonviable cells. HMBPP and POM_2_-C-HMBP (100 nM) were used as positive controls. EC_50_ values were determined as the concentration that induced 50% of the maximum proliferative effect. All experiments were performed at least three times using cells from at least two different donors. Data were analyzed using GraphPad Prism 6, and the EC_50_ values along with 95% confidence intervals are reported.

#### ELISA Assay for Interferon-γ

The interferon-γ release was quantified using the previously reported protocol.^41^ In T cell media, K562 cells were cultured with a density below 1 × 10^6^. The cells were split 1 day prior to the experiment. Purified Vγ9Vδ2 T cells were thawed and grown overnight with addition of IL-2. Then, K562 cells were treated with different prepared dilutions of test compounds in the T cell media and incubated for 1 h. Three steps of washing were done by spinning 20 s in a benchtop centrifuge with strip tube (size 250 μL). The media was aspirated from the cell pellet, and then cells were resuspended in T cell media. In a 96 well plate, 20 μL of the compound-treated K562 cells were added into T cells at a ratio of 3:1 (T cells/K562 cells) resulting in 4000 K562 cells and 12,000 effector T cells per well. The plates were incubated for 20 h and interferon-γ release was accessed by ELISA. Absorbance for tested compounds fell in the range of standard curve in the ELISA. An interferon-γ release versus concentration plot was prepared to analyze the results and determine the EC_50_ values using GraphPad Prism. ELISA experiments were performed with at least two different donors on at least two different days (*n* = 4).

#### Time Course Stability Study

The plasma stability of the prodrugs at various time points was determined using LC–MS following an incubation with human plasma at 37 °C.^27^ The human plasma was diluted to 50% using tris buffered saline at pH 7.5. The test compounds were introduced at a final concentration of 100 μM in 200 μL of volume. Compounds were incubated in plasma for various time points as indicated. For every time point, 25 μL of each sample was extracted using 75 μL of LC–MS grade acetonitrile following vigorous mixing. The precipitated debris was pelleted by centrifugation at 10,000 rcf for 2 min. Following extraction, 10 μL of extract was evaluated by LC–MS which is a Waters Synapt G2-Si mass spectrometer with positive polarity. The gradient started at 50% acetonitrile which was increased to 90% acetonitrile over 9 min. To analyze the samples, the precursor ion [M + H]^+^ and sodium adduct [M + Na]^+^ were observed based on the calculated *m*/*z* values. The integrated peak values of both the ions were added to calculate fraction remaining.

#### Metabolism Studies

The cellular metabolism of the prodrugs was determined using LC–MS post incubation with K562 cells.^42,43^ The K562 cells were resuspended at a concentration of 2.5 × 10^6^ cells in 500 μL of the K562 media. These cells were treated with 100 μM of the compound for 1 h. Post incubation, the cells were pelleted by centrifugation for 3 min at 600 rcf, and the media was aspirated. The cells were washed with cold phosphate buffer saline to remove any unabsorbed compound. The metabolites were extracted by addition of 200 μL of extraction solvent (75% LC–MS grade CH_3_CN, 25% 75 mM NH_3_OH), followed by vigorous mixing for 30 s. The cellular debris was pelleted by centrifugation at 10,000 rcf for 2 min. Following extraction, 10 μL of extract was evaluated by LCMS using a Waters Synapt G2-Si mass spectrometer with negative polarity using a C18 column. The solvent system was pure 10 mM triethylammonium acetate (A) and 10 mM triethylammonium acetate in methanol (10/90, v/v) (B). The gradient increased from 10% B to 80% B over 4 min, holding until 6.5 min. The plasma metabolites were determined using the same extraction and detection methods as the cellular metabolites following 1 h incubation in 50% pooled human plasma in tris-buffered saline.

## Supporting Information Available

NMR spectra (PDF)
Molecular formula strings (CSV)

## Abbreviations

BTN: butyrophilin
HMBPP: (*E*)-4-hydroxy-3-methyl-but-2-enyl diphosphate
POM: pivaloyloxymethyl
TCR: T cell receptor
MHC: major histocompatibility complex
