# Supplementary material for: Diester Prodrugs of a Phosphonate Butyrophilin Ligand Display Improved Cell Potency, Plasma Stability, and Payload Internalization
Source: J Med Chem. 2023 Nov 7;66(22):15309–25. doi: 10.1021/acs.jmedchem.3c01358 (PMC10683022; doi:10.1021/acs.jmedchem.3c01358)
Supplement: Supplementary file 1 — jm3c01358_si_001.pdf [file jm3c01358_si_001.pdf]

Diester prodrugs of a phosphonate butyrophilin ligand display improved cell potency, plasma stability, and payload internalization

Umed Singh,[1] Girija Pawge,[2] Sarita Rani,[2] Chia-Hung Christine Hsiao,[2]

Andrew J. Wiemer,[2,3*] and David F. Wiemer[1,4*]

[1]*Department of Chemistry, University of Iowa, Iowa City, Iowa 52242-1294, United States*

[2]*Department of Pharmaceutical Sciences, University of Connecticut, Storrs, CT 06269-3092, United States*

[3]*Institute for Systems Genomics, University of Connecticut, Storrs, CT 06269-3092, United States*

[4]*Department of Pharmacology, University of Iowa, Iowa City, Iowa 52242-1109, United States*

E-mail: *david-wiemer@uiowa.edu*

Table of Contents

^{1}H NMR Spectrum of Compound **9a** ($CDCl_3$, 500 MHz) 2

^{13}C NMR Spectrum of Compound **9a** ($CDCl_3$, 126 MHz) 2

^{31}P NMR Spectrum of Compound **9a** ($CDCl_3$, 203 MHz) 2

^{1}H NMR Spectrum of Compound **9b** ($CDCl_3$, 500 MHz) 2

^{13}C NMR Spectrum of Compound **9b** ($CDCl_3$, 126 MHz) 2

^{31}P NMR Spectrum of Compound **9b** ($CDCl_3$, 203 MHz) 2

^{1}H NMR Spectrum of Compound **8c** ($CDCl_3$, 500 MHz) 2

^{13}C NMR Spectrum of Compound **8c** ($CDCl_3$, 126 MHz) 2

^{31}P NMR Spectrum of Compound **8c** ($CDCl_3$, 203 MHz) 2

^{1}H NMR Spectrum of Compound **9c** ($CDCl_3$, 500 MHz) 2
^{13}C NMR Spectrum of Compound **9c** ($CDCl_3$, 126 MHz) 2
^{31}P NMR Spectrum of Compound **9c** ($CDCl_3$, 203 MHz) 2
^{1}H NMR Spectrum of Compound **8d** ($CDCl_3$, 500 MHz) 2
^{13}C NMR Spectrum of Compound **8d** ($CDCl_3$, 126 MHz) 2
^{31}P NMR Spectrum of Compound **8d** ($CDCl_3$, 203 MHz) 2
^{1}H NMR Spectrum of Compound **9d** ($CDCl_3$, 500 MHz) 2
^{13}C NMR Spectrum of Compound **9d** ($CDCl_3$, 126 MHz) 2
^{31}P NMR Spectrum of Compound **9d** ($CDCl_3$, 203 MHz) 2
^{1}H NMR Spectrum of Compound **8e** ($CDCl_3$, 500 MHz) 2
^{13}C NMR Spectrum of Compound **8e** ($CDCl_3$, 126 MHz) 2
^{31}P NMR Spectrum of Compound **8e** ($CDCl_3$, 203 MHz) 2
^{1}H NMR Spectrum of Compound **9e** ($CDCl_3$, 500 MHz) 2
^{13}C NMR Spectrum of Compound **9e** ($CDCl_3$, 126 MHz) 2
^{31}P NMR Spectrum of Compound **9e** ($CDCl_3$, 203 MHz) 2
^{1}H NMR Spectrum of Compound **8f** ($CDCl_3$, 500 MHz) 2
^{13}C NMR Spectrum of Compound **8f** ($CDCl_3$, 126 MHz) 2
^{31}P NMR Spectrum of Compound **8f** ($CDCl_3$, 203 MHz) 2
^{1}H NMR Spectrum of Compound **9f** ($CDCl_3$, 500 MHz) 2
^{13}C NMR Spectrum of Compound **9f** ($CDCl_3$, 126 MHz) 2
^{31}P NMR Spectrum of Compound **9f** ($CDCl_3$, 203 MHz) 2
^{1}H NMR Spectrum of Compound **8g** ($CDCl_3$, 500 MHz) 2
^{13}C NMR Spectrum of Compound **8g** ($CDCl_3$, 126 MHz) 2
^{19}F NMR Spectrum of Compound **8g** ($CDCl_3$, 471 MHz) 2
^{31}P NMR Spectrum of Compound **8g** ($CDCl_3$, 203 MHz) 2
^{1}H NMR Spectrum of Compound **9g** ($CDCl_3$, 500 MHz) 2
^{13}C NMR Spectrum of Compound **9g** ($CDCl_3$, 126 MHz) 2
^{19}F NMR Spectrum of Compound **9g** ($CDCl_3$, 471 MHz) 2
^{31}P NMR Spectrum of Compound **9g** ($CDCl_3$, 203 MHz) 2
^{1}H NMR Spectrum of Compound **8h** ($CDCl_3$, 400 MHz) 2
^{13}C NMR Spectrum of Compound **8h** ($CDCl_3$, 126 MHz) 2
^{31}P NMR Spectrum of Compound **8h** ($CDCl_3$, 162 MHz) 2
^{19}F NMR Spectrum of Compound **8h** ($CDCl_3$, 471 MHz) 2
^{1}H NMR Spectrum of Compound **9h** ($CDCl_3$, 400 MHz) 2
^{13}C NMR Spectrum of Compound **9h** ($CDCl_3$, 126 MHz) 2
^{31}P NMR Spectrum of Compound **9h** ($CDCl_3$, 162 MHz) 2
^{19}F NMR Spectrum of Compound **9h** ($CDCl_3$, 471 MHz) 2
^{1}H NMR Spectrum of Compound **8i** ($CDCl_3$, 500 MHz) 2
^{13}C NMR Spectrum of Compound **8i** ($CDCl_3$, 126 MHz) 2
^{31}P NMR Spectrum of Compound **8i** ($CDCl_3$, 203 MHz) 2

^{1}H NMR Spectrum of Compound **9i** ($CDCl_3$, 500 MHz)....2
^{13}C NMR Spectrum of Compound **9i** ($CDCl_3$, 126 MHz)....2
^{31}P NMR Spectrum of Compound **9i** ($CDCl_3$, 203 MHz)....2
^{1}H NMR Spectrum of Compound **8j** ($CDCl_3$, 400 MHz)....2
^{13}C NMR Spectrum of Compound **8j** ($CDCl_3$, 101 MHz)....2
^{31}P NMR Spectrum of Compound **8j** ($CDCl_3$, 162 MHz)....2
^{1}H NMR Spectrum of Compound **9j** ($CDCl_3$, 400 MHz)....2
^{13}C NMR Spectrum of Compound **9j** ($CDCl_3$, 101 MHz)....2
^{31}P NMR Spectrum of Compound **9j** ($CDCl_3$, 162 MHz)....2
^{1}H NMR Spectrum of Compound **8k** ($CDCl_3$, 500 MHz)....2
^{13}C NMR Spectrum of Compound **8k** ($CDCl_3$, 126 MHz)....2
^{31}P NMR Spectrum of Compound **8k** ($CDCl_3$, 203 MHz)....2
^{1}H NMR Spectrum of Compound **9k** ($CDCl_3$, 500 MHz)....2
^{13}C NMR Spectrum of Compound **9k** ($CDCl_3$, 126 MHz)....2
^{31}P NMR Spectrum of Compound **9k** ($CDCl_3$, 203 MHz)....2
^{1}H NMR Spectrum of Compound **11x** ($CDCl_3$, 500 MHz)....2
^{13}C NMR Spectrum of Compound **11x** ($CDCl_3$, 126 MHz)....2
^{31}P NMR Spectrum of Compound **11x** ($CDCl_3$, 203 MHz)....2
^{1}H NMR Spectrum of Compound **12x** ($CDCl_3$, 500 MHz)....2
^{13}C NMR Spectrum of Compound **12x** ($CDCl_3$, 126 MHz)....2
^{31}P NMR Spectrum of Compound **12x** ($CDCl_3$, 203 MHz)....2
^{1}H NMR Spectrum of Compound **11y** ($CDCl_3$, 400 MHz)....2
^{13}C NMR Spectrum of Compound **11y** ($CDCl_3$, 101 MHz)....2
^{31}P NMR Spectrum of Compound **11y** ($CDCl_3$, 162 MHz)....2
^{1}H NMR Spectrum of Compound **12y** ($CDCl_3$, 400 MHz)....2
^{13}C NMR Spectrum of Compound **12y** ($CDCl_3$, 101 MHz)....2
^{31}P NMR Spectrum of Compound **12y** ($CDCl_3$, 162 MHz)....2
^{1}H NMR Spectrum of Compound **11z** ($CDCl_3$, 400 MHz)....2
^{13}C NMR Spectrum of Compound **11z** ($CDCl_3$, 101 MHz)....2
^{31}P NMR Spectrum of Compound **11z** ($CDCl_3$, 162 MHz)....2
^{1}H NMR Spectrum of Compound **12z** ($CDCl_3$, 400 MHz)....2
^{13}C NMR Spectrum of Compound **12z** ($CDCl_3$, 101 MHz)....2
^{31}P NMR Spectrum of Compound **12z** ($CDCl_3$, 162 MHz)....2
HPLC Chromatogram of Compound **9a**....2
HPLC Chromatogram of Compound **9b**....2
HPLC Chromatogram of Compound **8c**....2
HPLC Chromatogram of Compound **9c**....2
HPLC Chromatogram of Compound **8d**....2
HPLC Chromatogram of Compound **9d**....2
HPLC Chromatogram of Compound **8e**....2

HPLC Chromatogram of Compound **9e** 2
HPLC Chromatogram of Compound **8f**...2
HPLC Chromatogram of Compound **9f**...2
HPLC Chromatogram of Compound **8g** 2
HPLC Chromatogram of Compound **9g** 2
HPLC Chromatogram of Compound **8h**...2
HPLC Chromatogram of Compound **9h**...2
HPLC Chromatogram of Compound **8i**...2
HPLC Chromatogram of Compound **9i**...2
HPLC Chromatogram of Compound **8j**...2
HPLC Chromatogram of Compound **9j**...2
HPLC Chromatogram of Compound **8k**...2
HPLC Chromatogram of Compound **9k**...2
HPLC Chromatogram of Compound **11x** 2
HPLC Chromatogram of Compound **12x** 2
HPLC Chromatogram of Compound **11y** 2
HPLC Chromatogram of Compound **12y** 2
HPLC Chromatogram of Compound **11z** 2
HPLC Chromatogram of Compound **12z** 2
Figure S1. Stability in 50% human plasma of all tested compounds at 2- and 24-hour time points…………..……113
Figure S2. K562 and plasma metabolism study of compounds 11z and 12z at the 1 hour time point………...……114
Table S1. K562 uptake and metabolism data……………………………………………………………..……115
Table S2. Plasma Metabolism data………………………………………………………………….……116

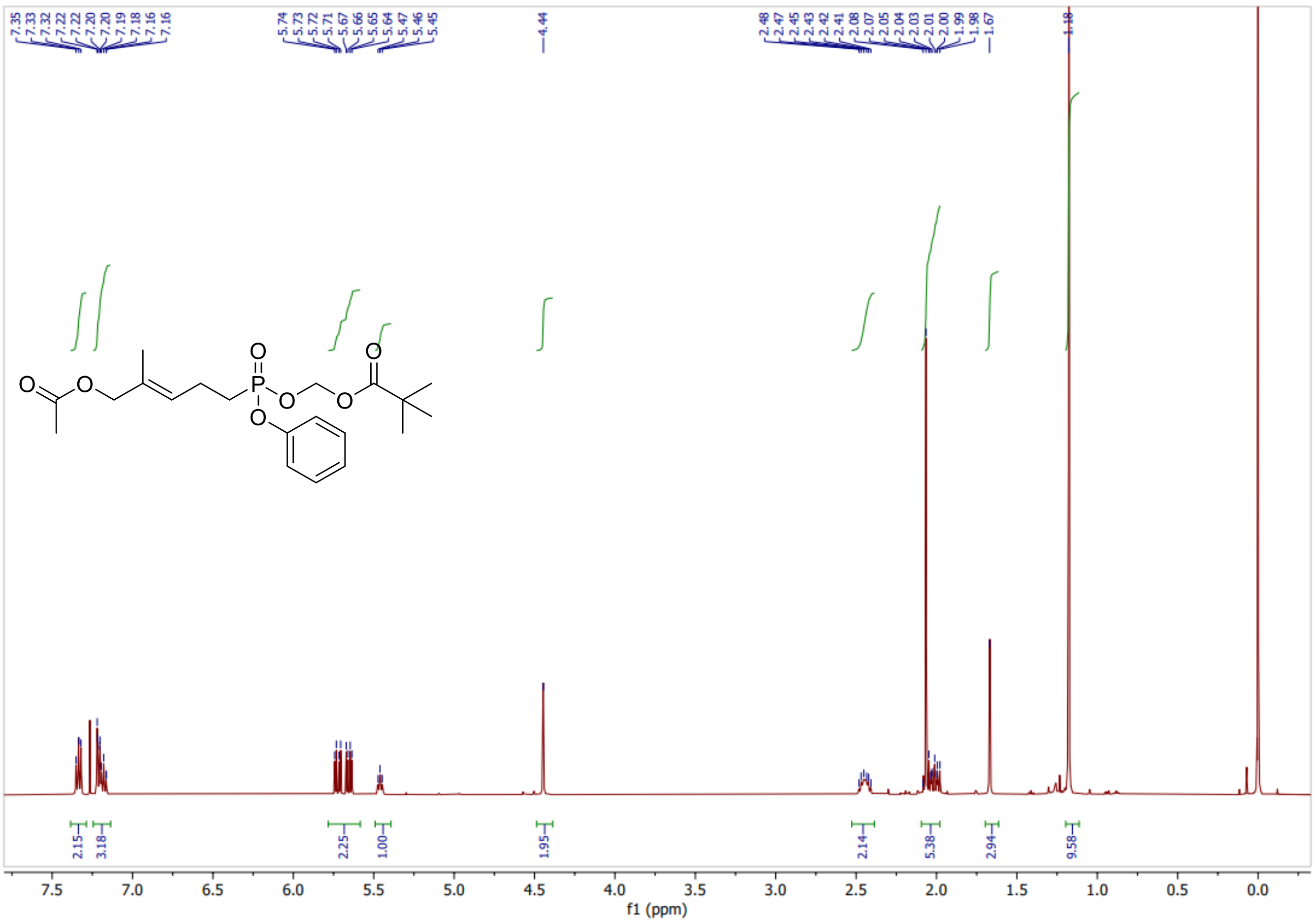

^{1}H NMR Spectrum of Compound **9a** ($CDCl_3$, 500 MHz)

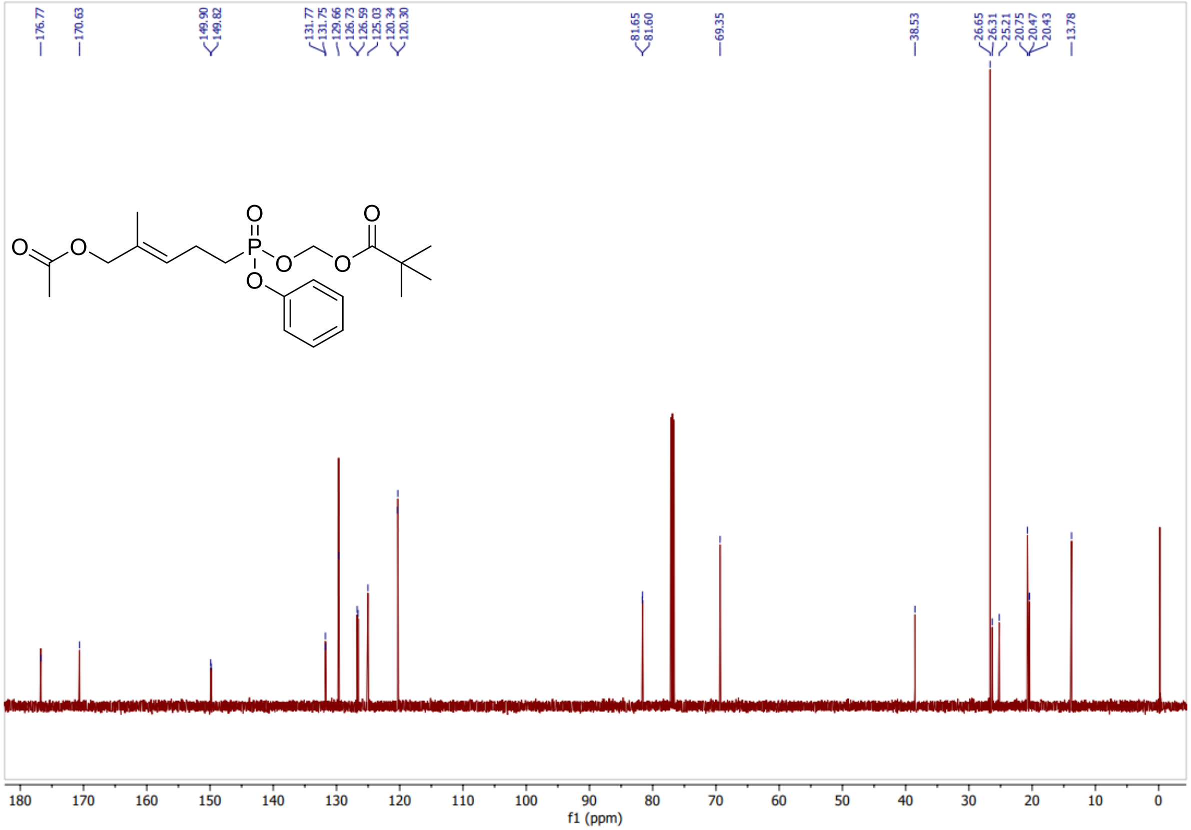

^{13}C NMR Spectrum of Compound **9a** ($CDCl_3$, 126 MHz)

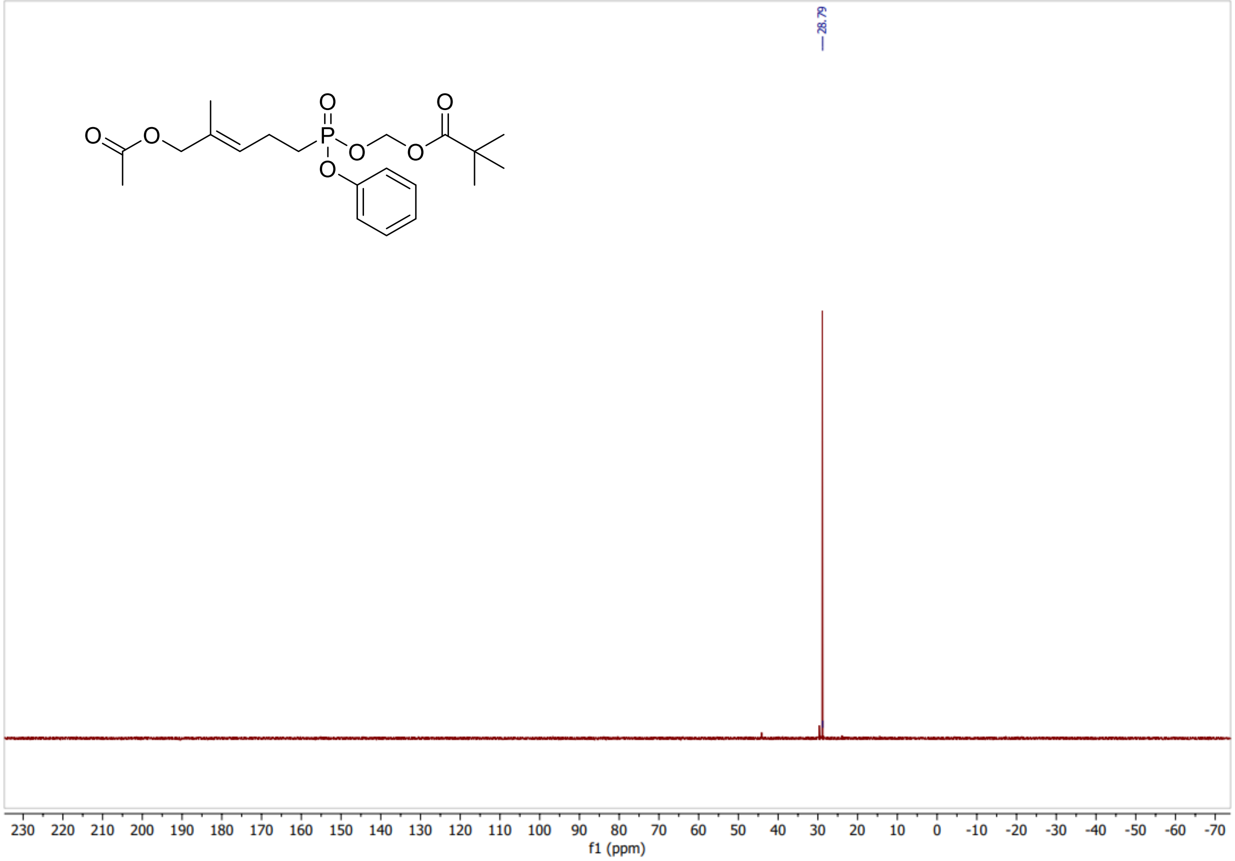

^{31}P NMR Spectrum of Compound **9a** ($CDCl_3$, 203 MHz)

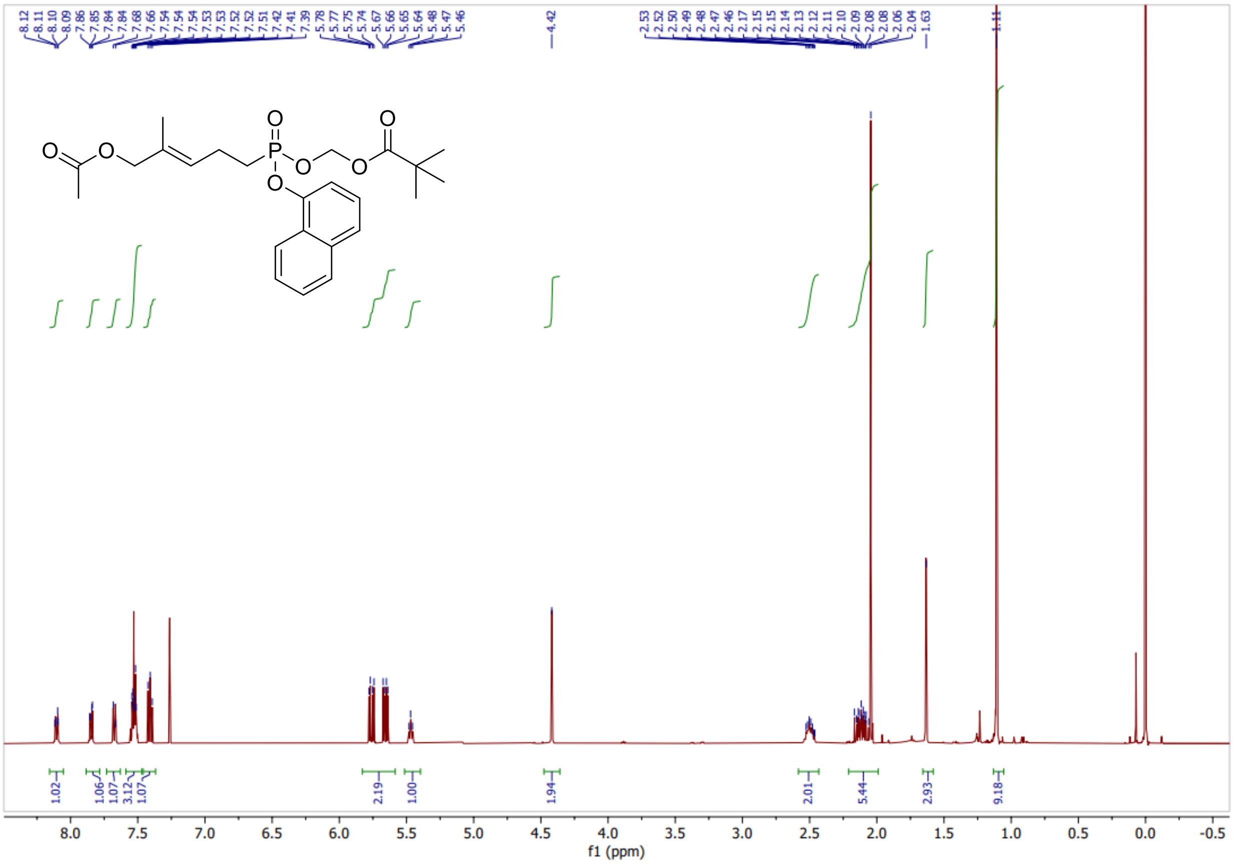

^{1}H NMR Spectrum of Compound **9b** ($CDCl_3$, 500 MHz)

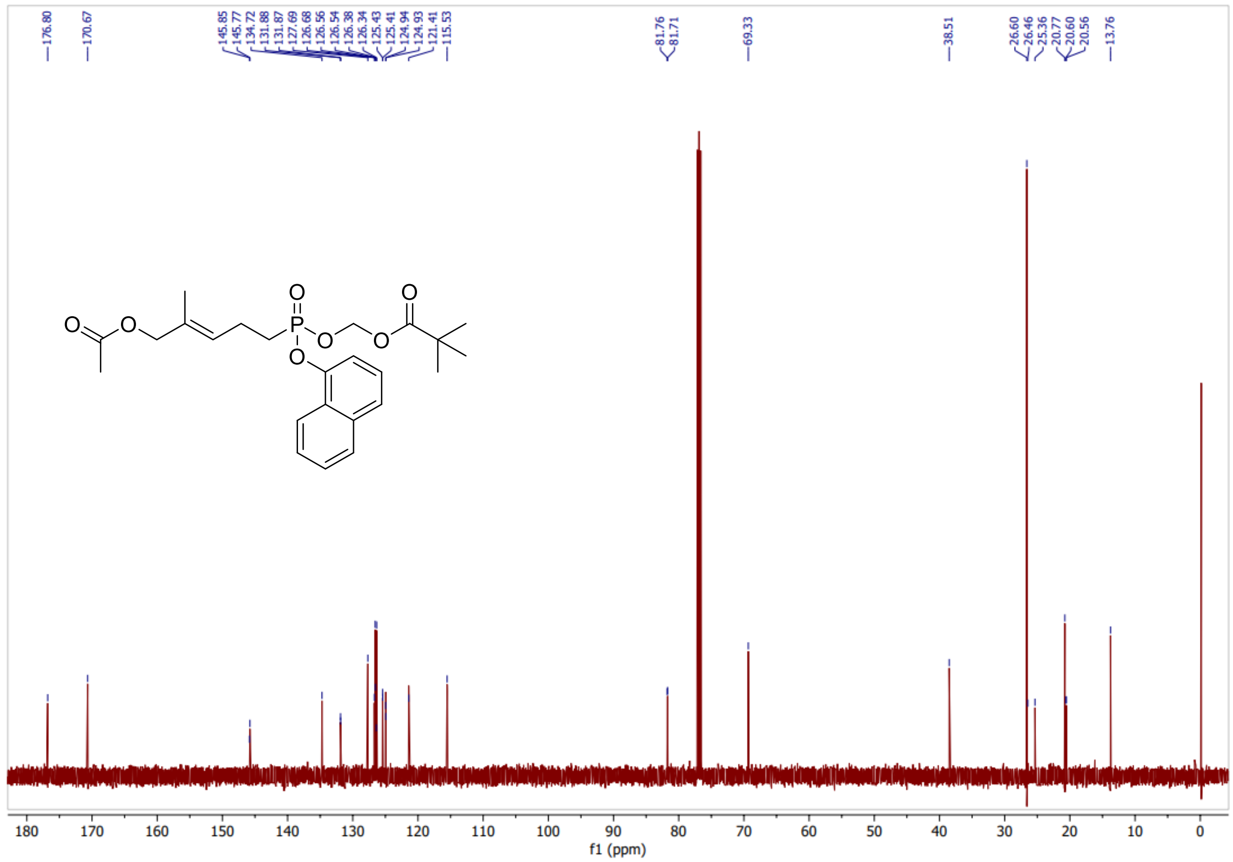

^{13}C NMR Spectrum of Compound **9b** ($CDCl_3$, 126 MHz)

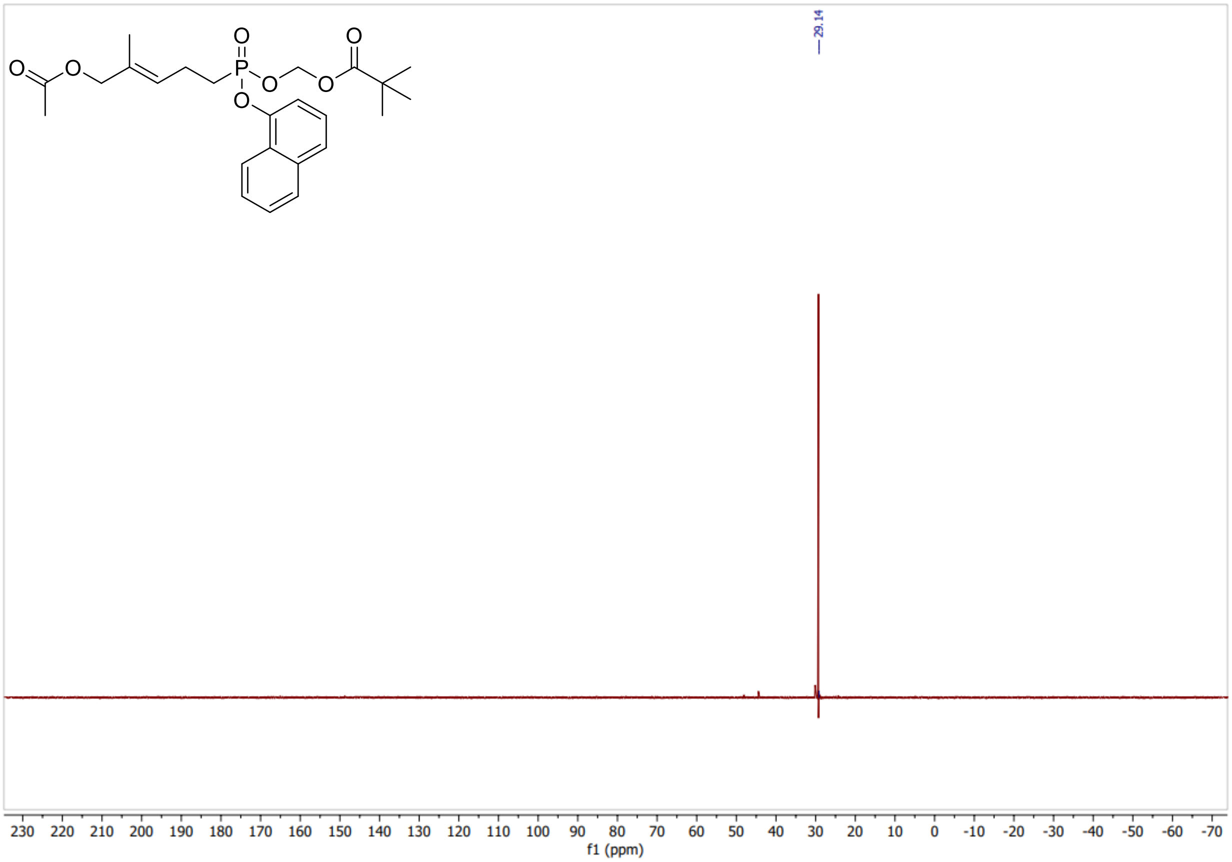

^{31}P NMR Spectrum of Compound **9b** ($CDCl_3$, 203 MHz)

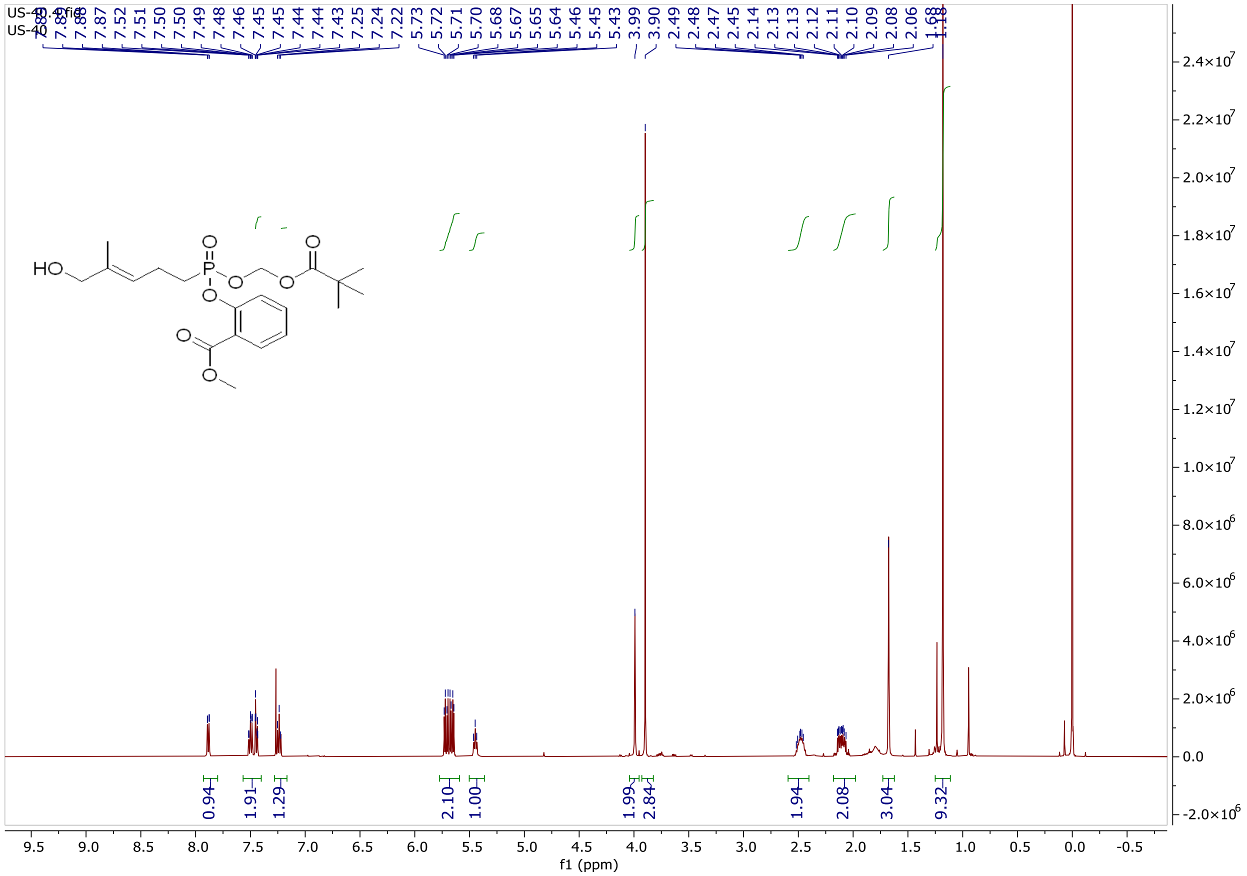

^{1}H NMR Spectrum of Compound **8c** ($CDCl_3$, 500 MHz)

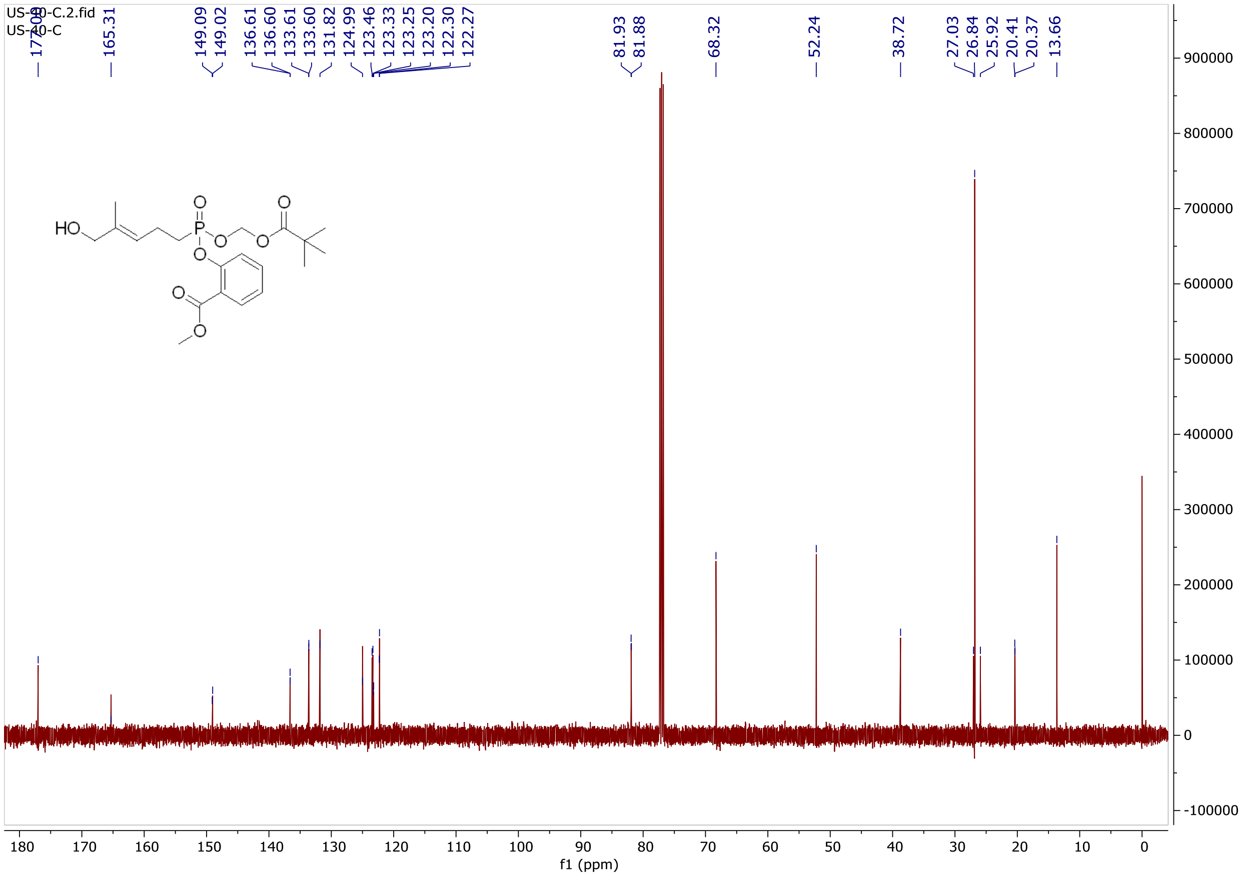

^{13}C NMR Spectrum of Compound **8c** ($CDCl_3$, 126 MHz)

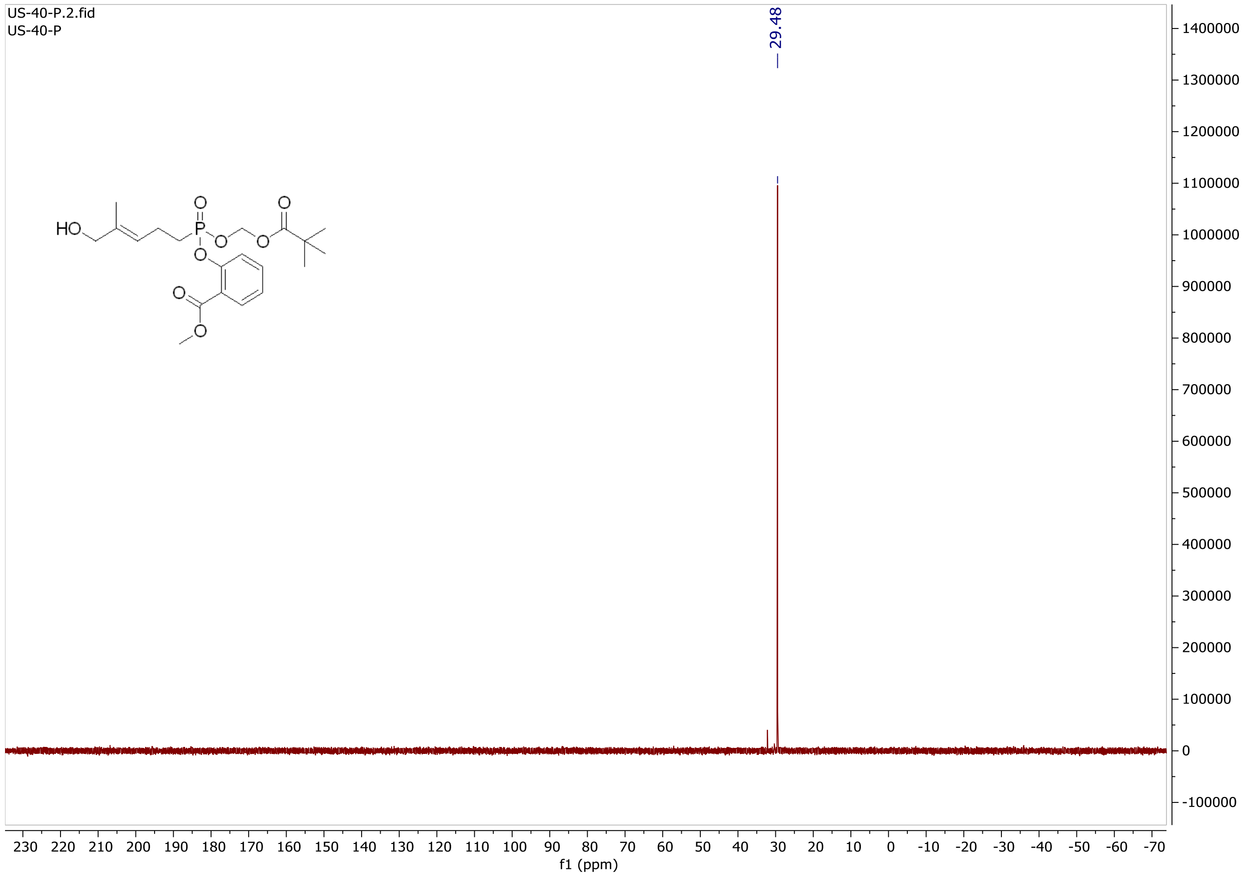

^{31}P NMR Spectrum of Compound **8c** ($CDCl_3$, 203 MHz)

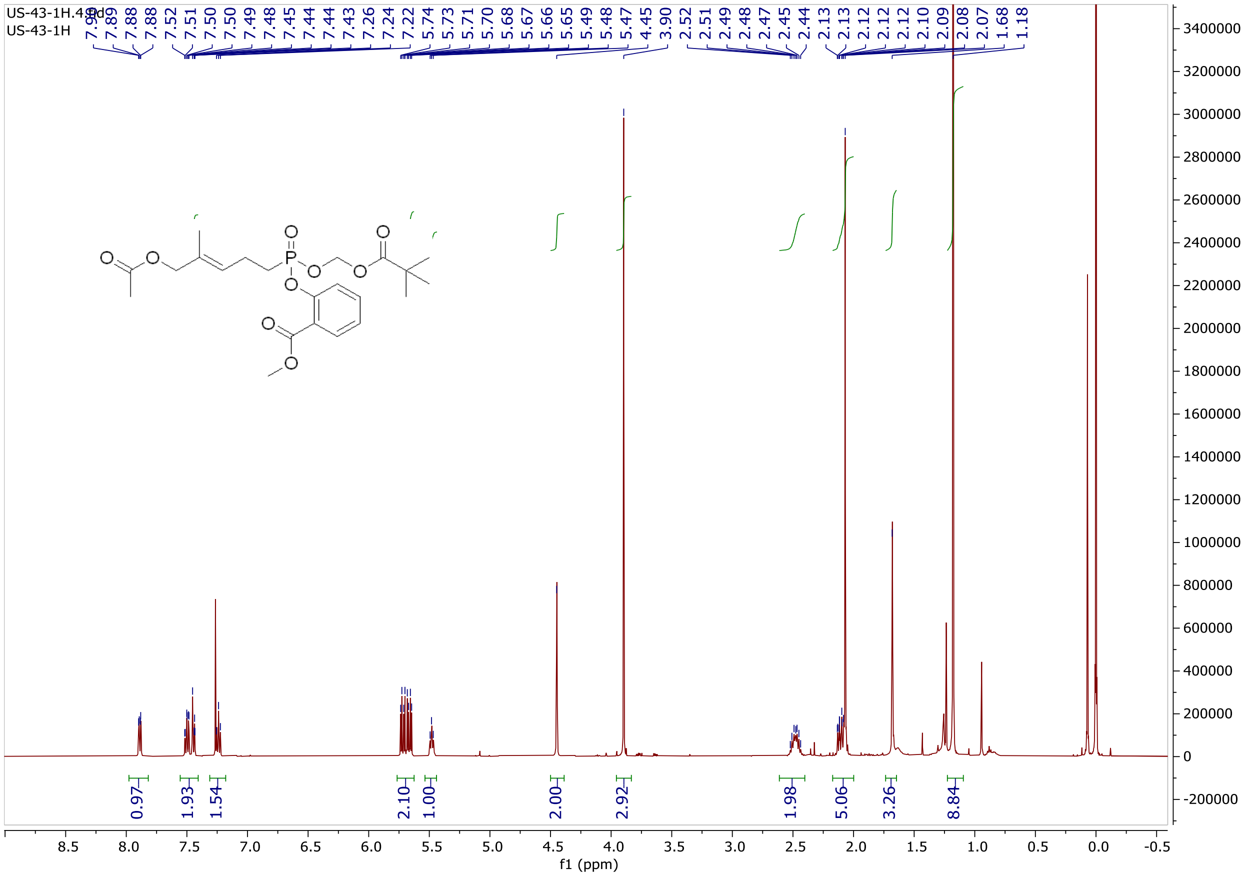

^{1}H NMR Spectrum of Compound **9c** ($CDCl_3$, 500 MHz)

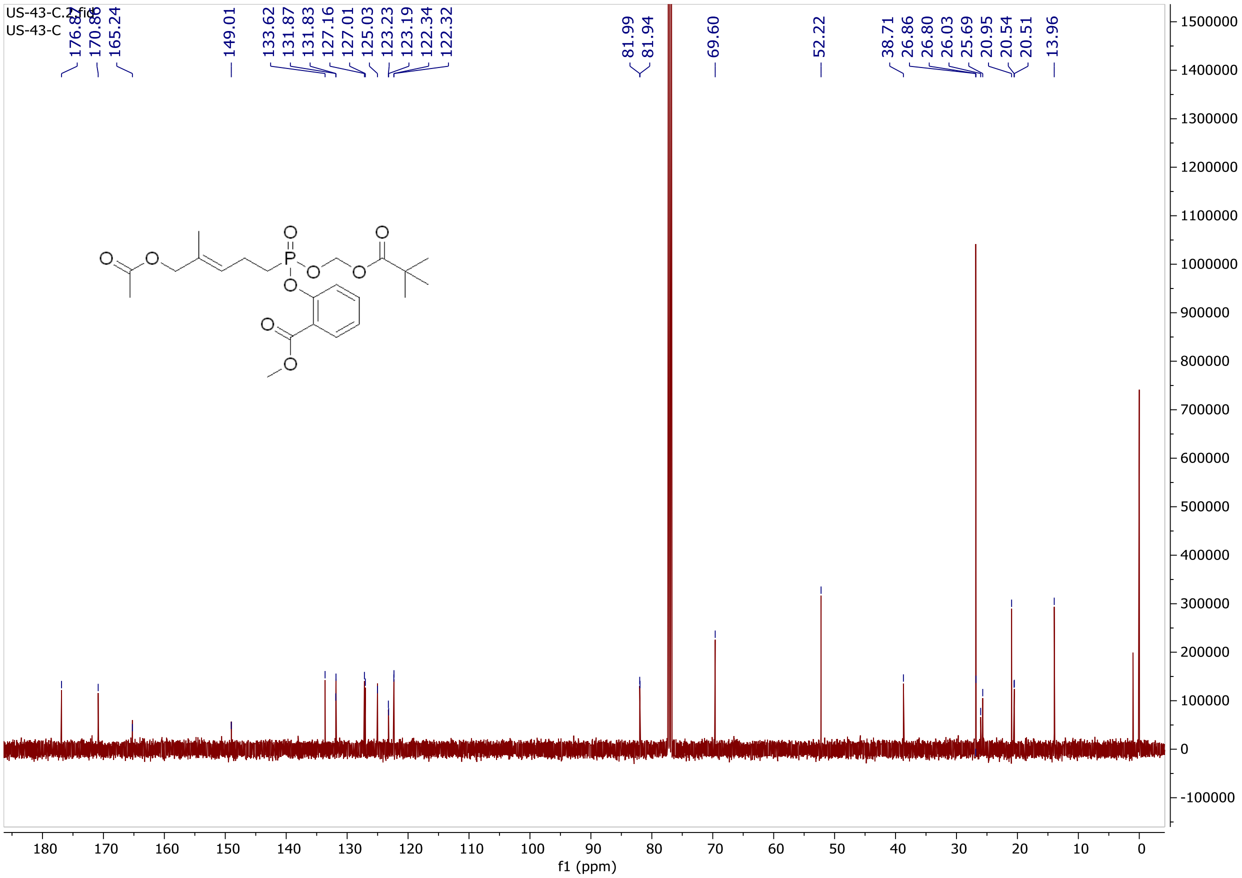

^{13}C NMR Spectrum of Compound **9c** ($CDCl_3$, 126 MHz)

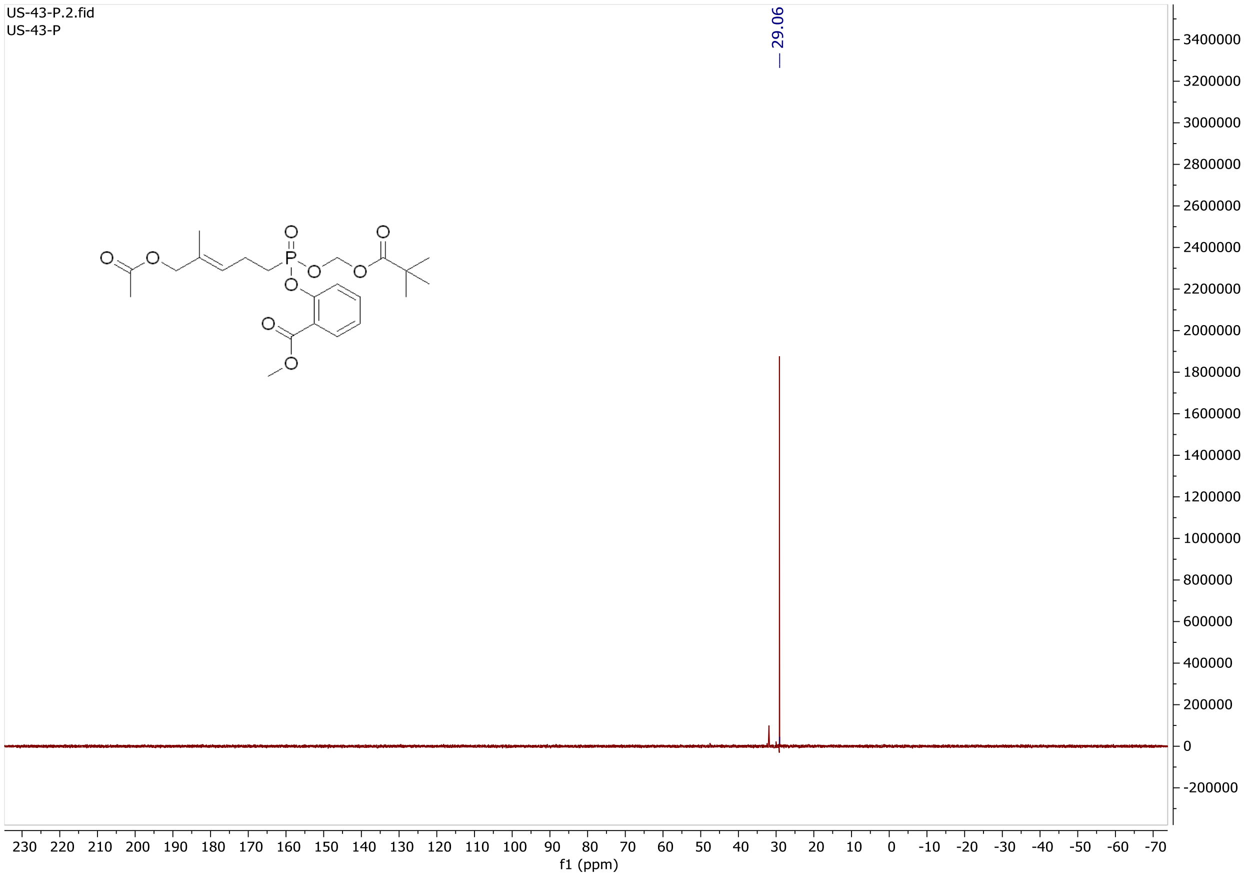

^{31}P NMR Spectrum of Compound **9c** ($CDCl_3$, 203 MHz)

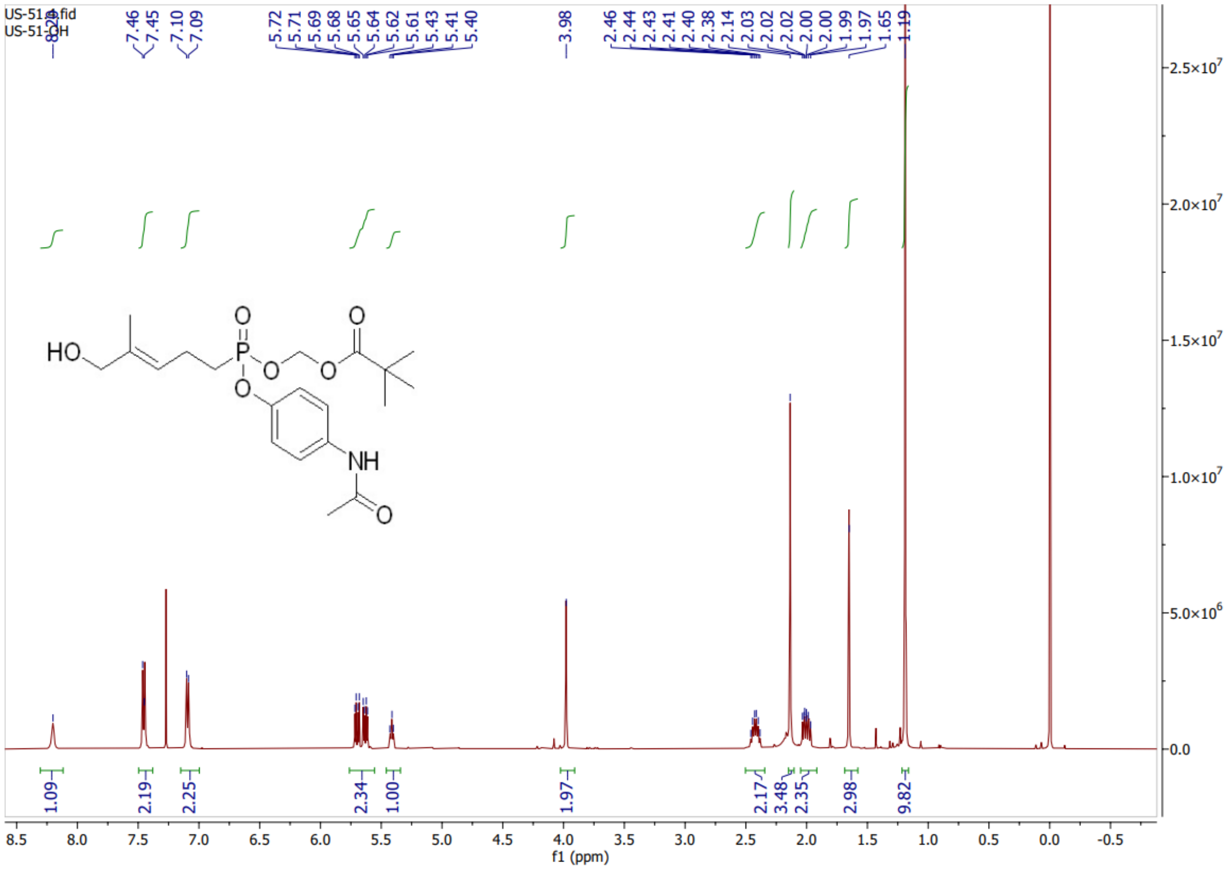

^{1}H NMR Spectrum of Compound **8d** ($CDCl_3$, 500 MHz)

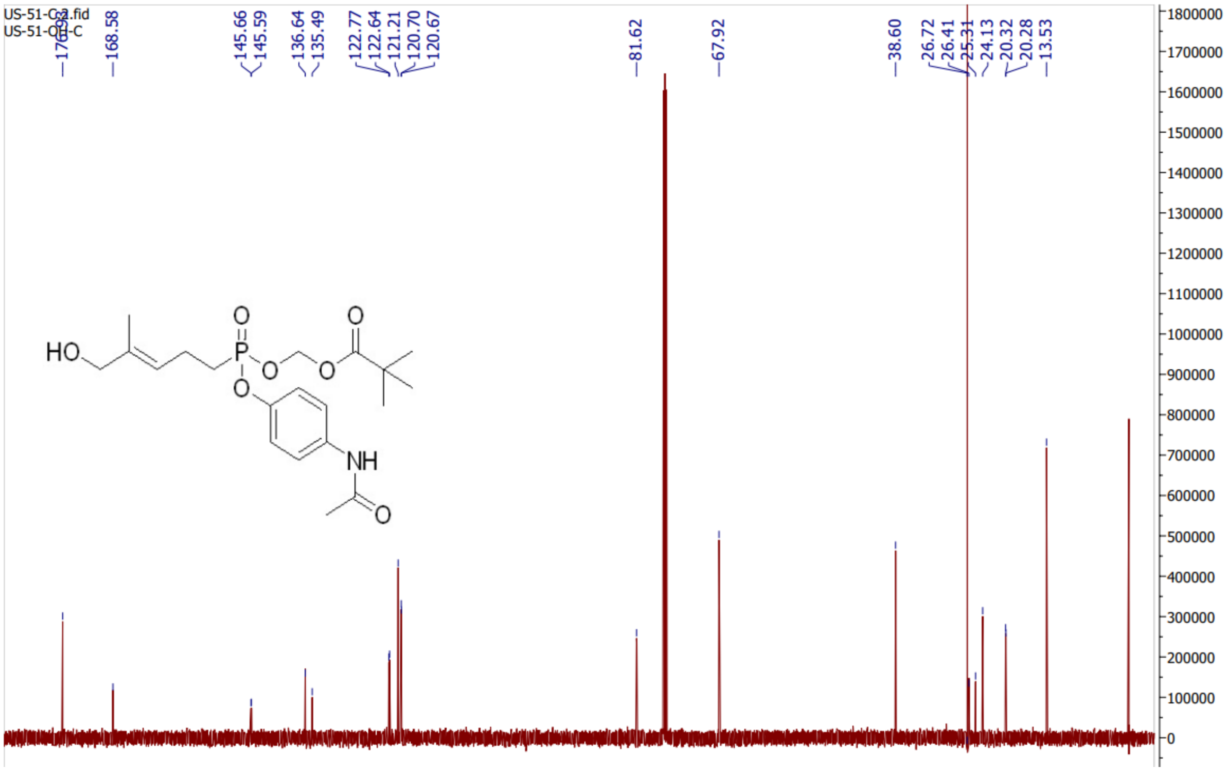

^{13}C NMR Spectrum of Compound **8d** ($CDCl_3$, 126 MHz)

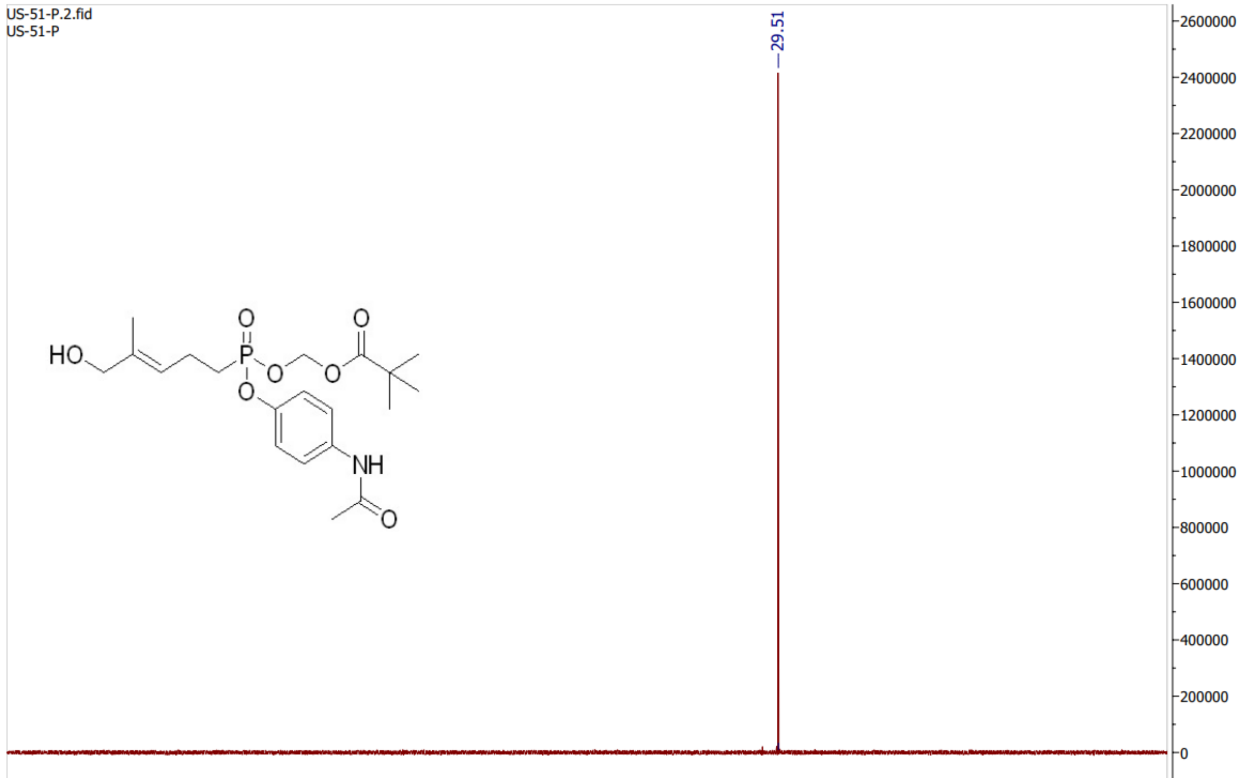

^{31}P NMR Spectrum of Compound **8d** ($CDCl_3$, 203 MHz)

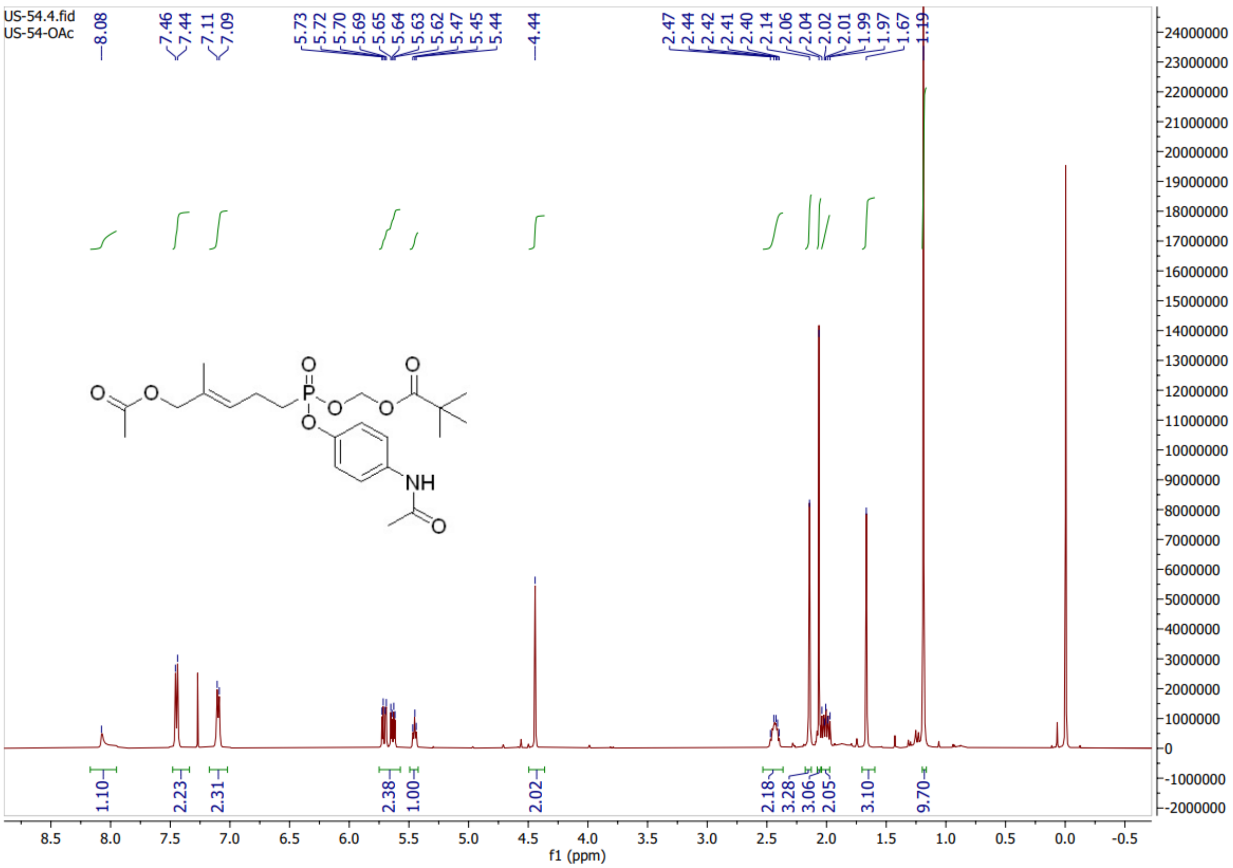

^{1}H NMR Spectrum of Compound **9d** ($CDCl_3$, 500 MHz)

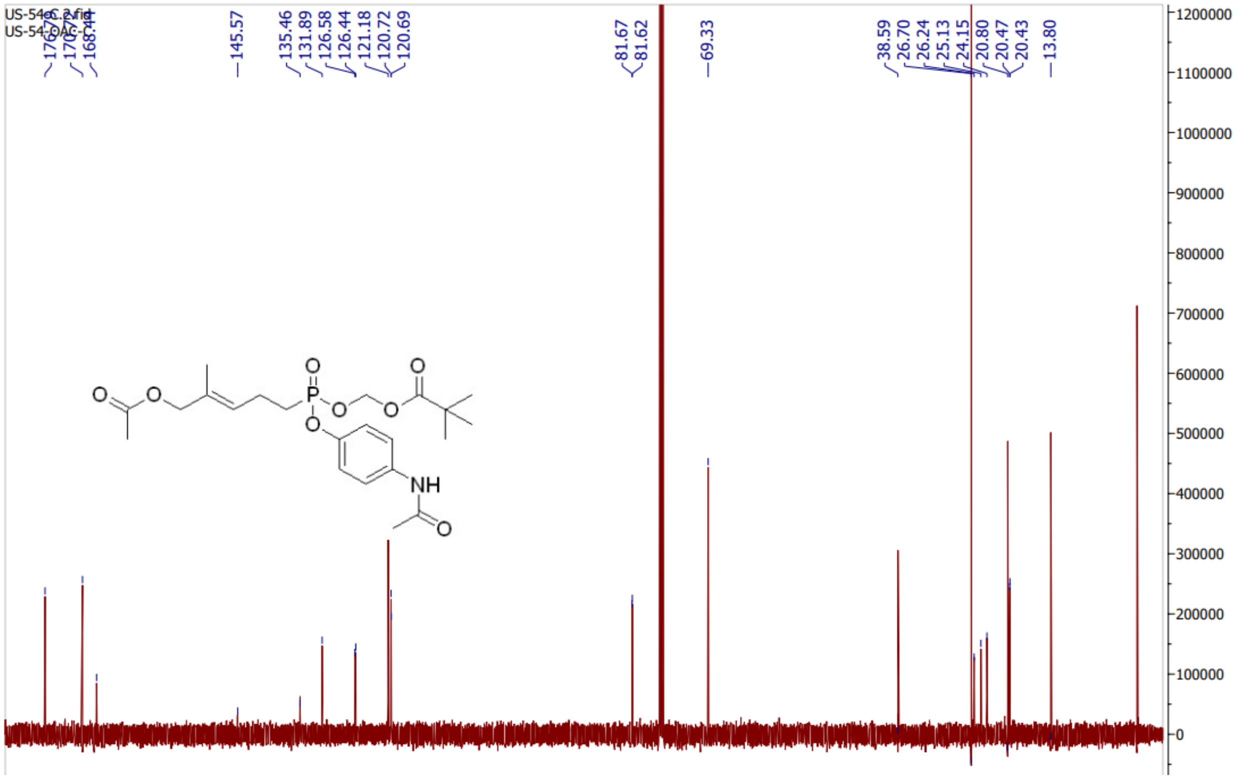

^{13}C NMR Spectrum of Compound **9d** ($CDCl_3$, 126 MHz)

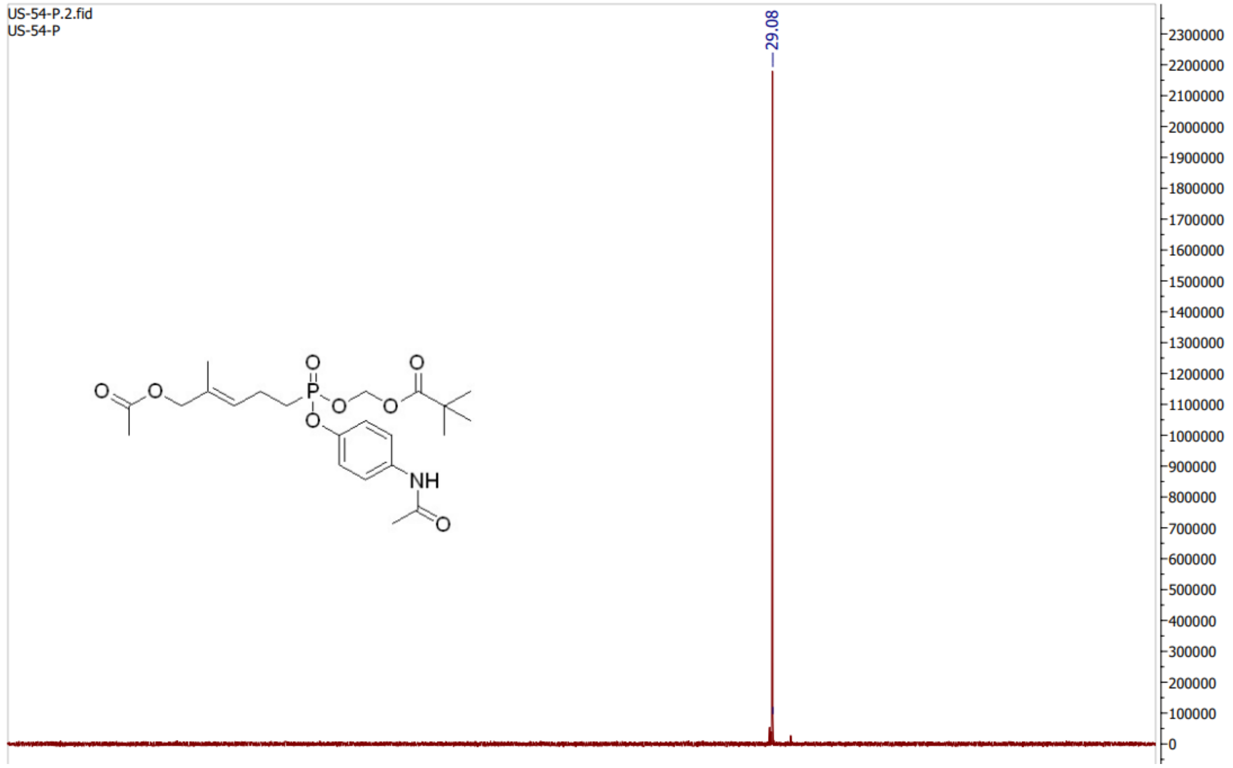

^{31}P NMR Spectrum of Compound **9d** ($CDCl_3$, 203 MHz)

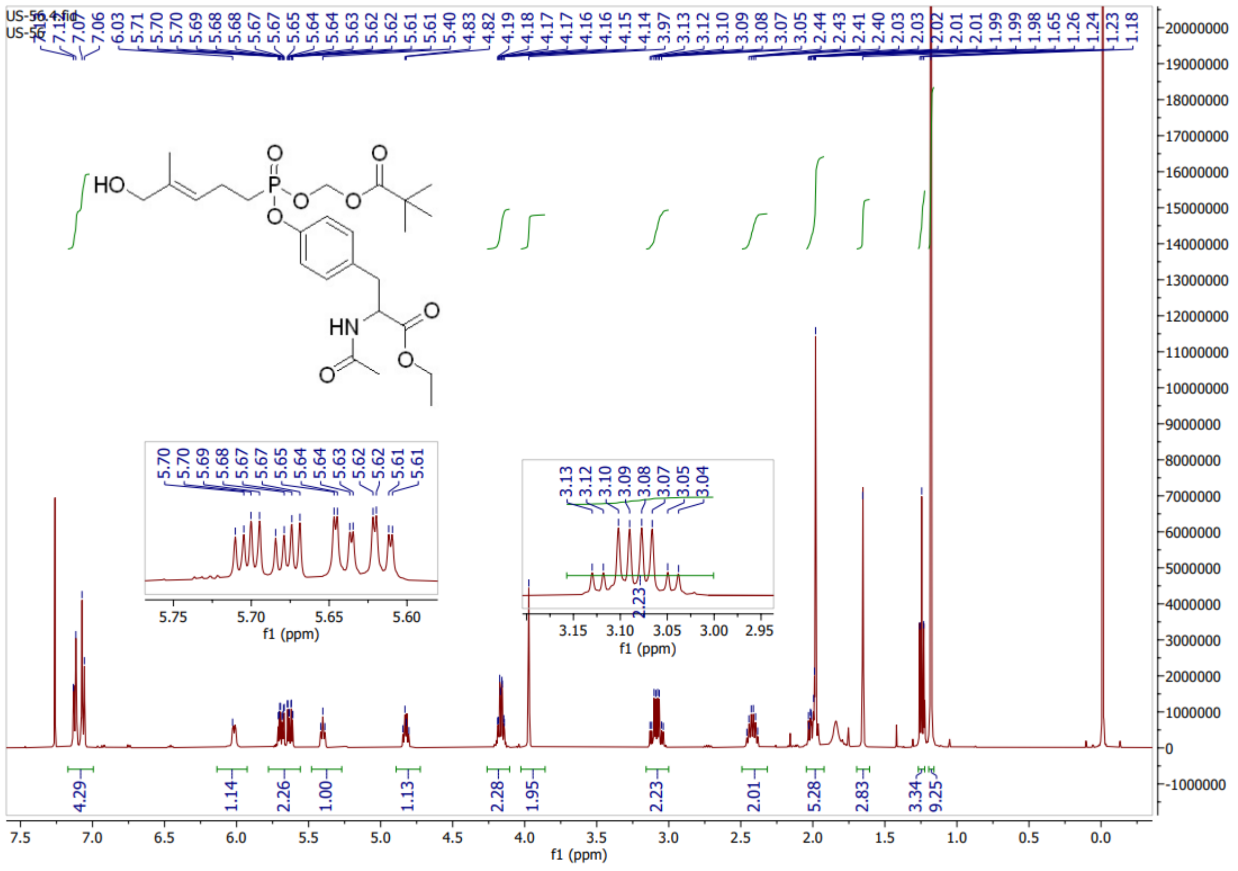

^{1}H NMR Spectrum of Compound **8e** ($CDCl_3$, 500 MHz)

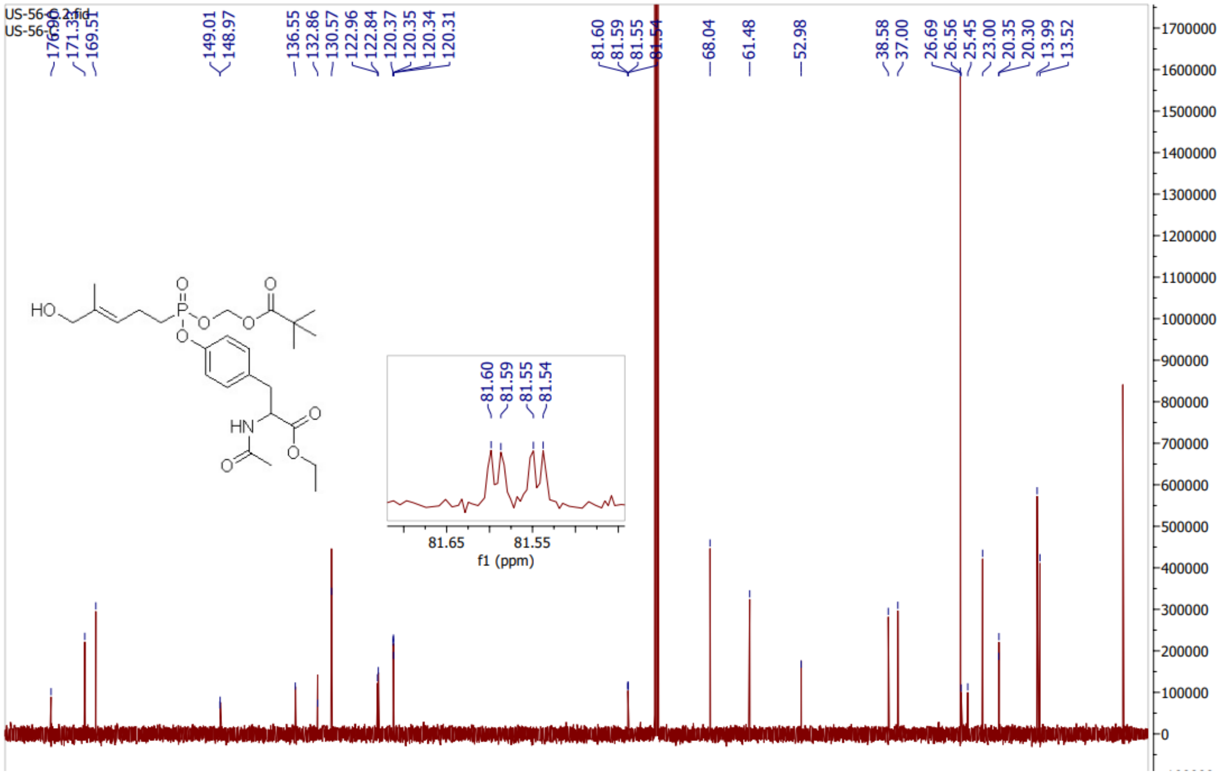

^{13}C NMR Spectrum of Compound **8e** ($CDCl_3$, 126 MHz)

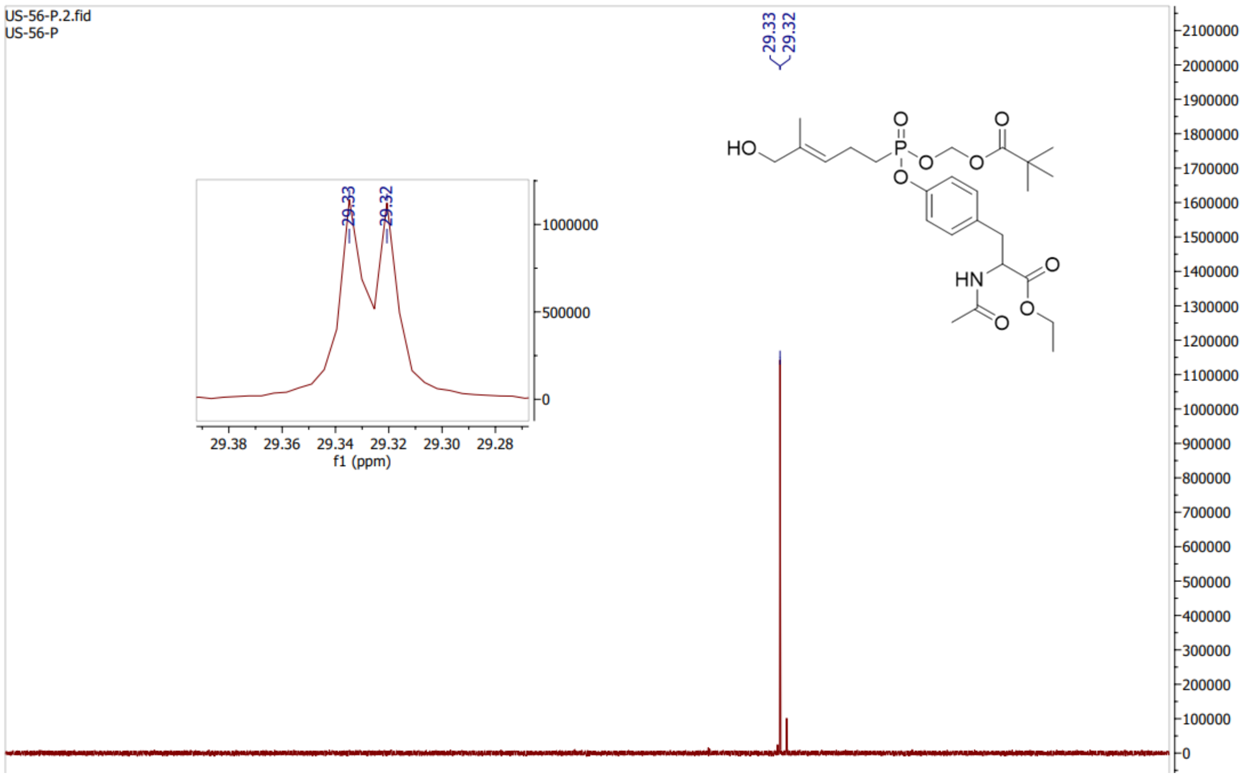

^{31}P NMR Spectrum of Compound **8e** (CDCl$_3$, 203 MHz)

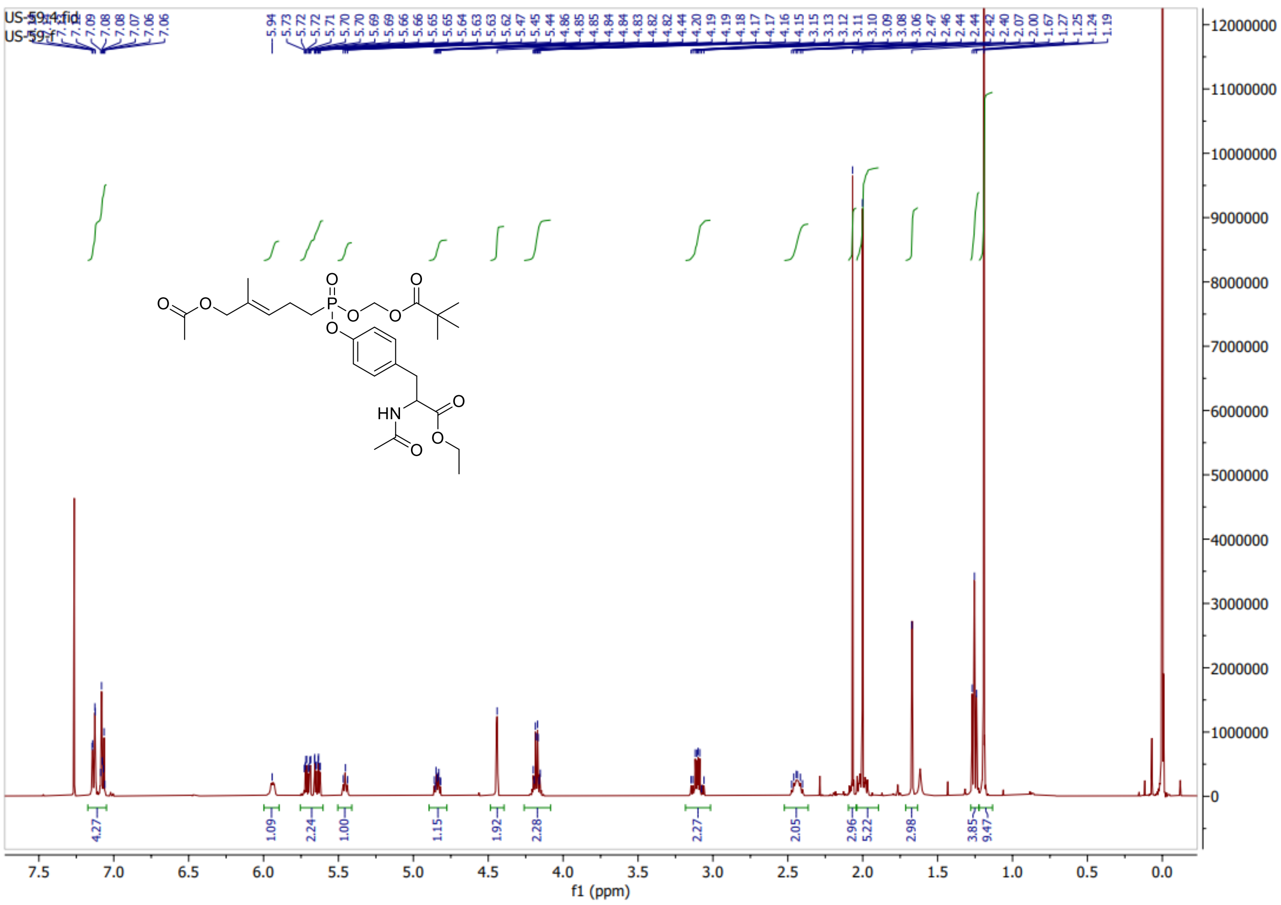

^{1}H NMR Spectrum of Compound **9e** ($CDCl_3$, 500 MHz)

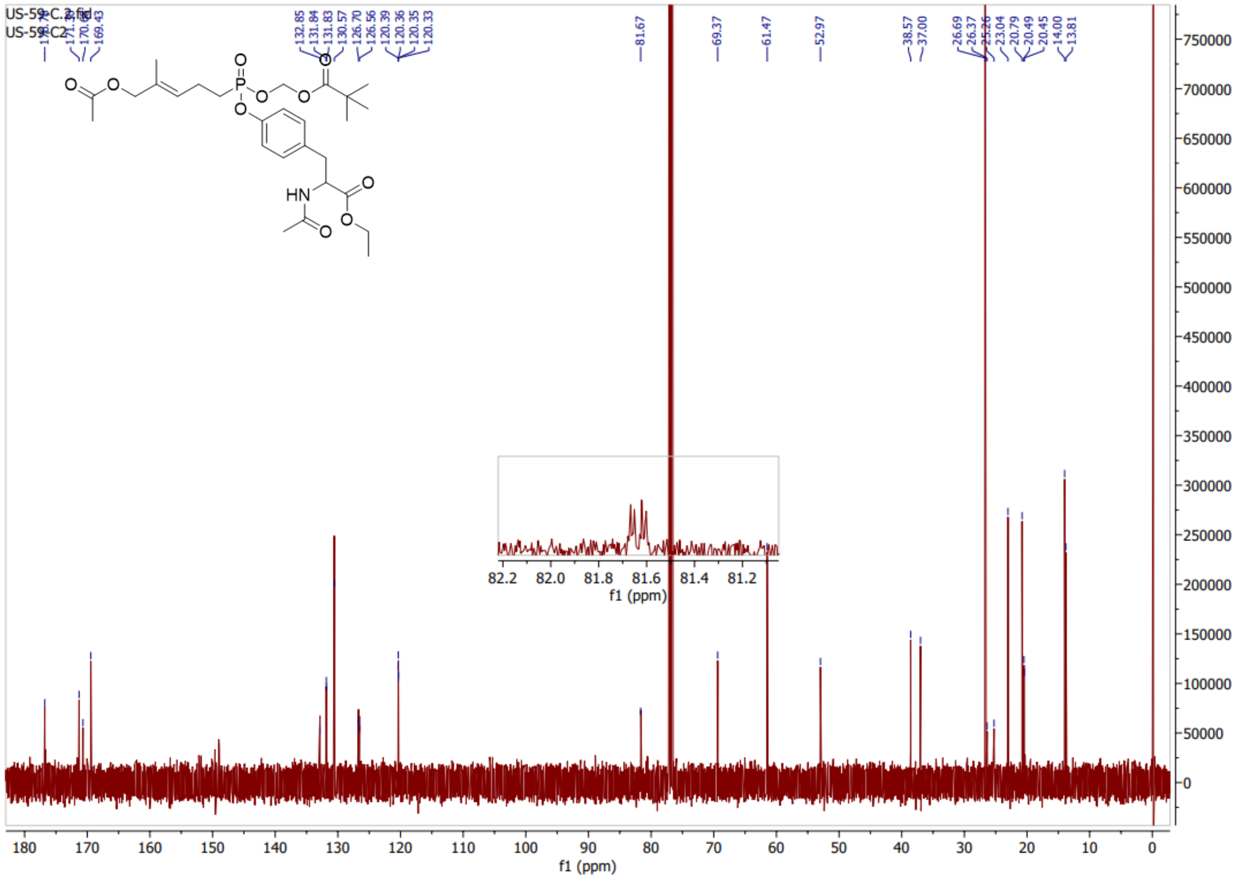

^{13}C NMR Spectrum of Compound **9e** ($CDCl_3$, 126 MHz)

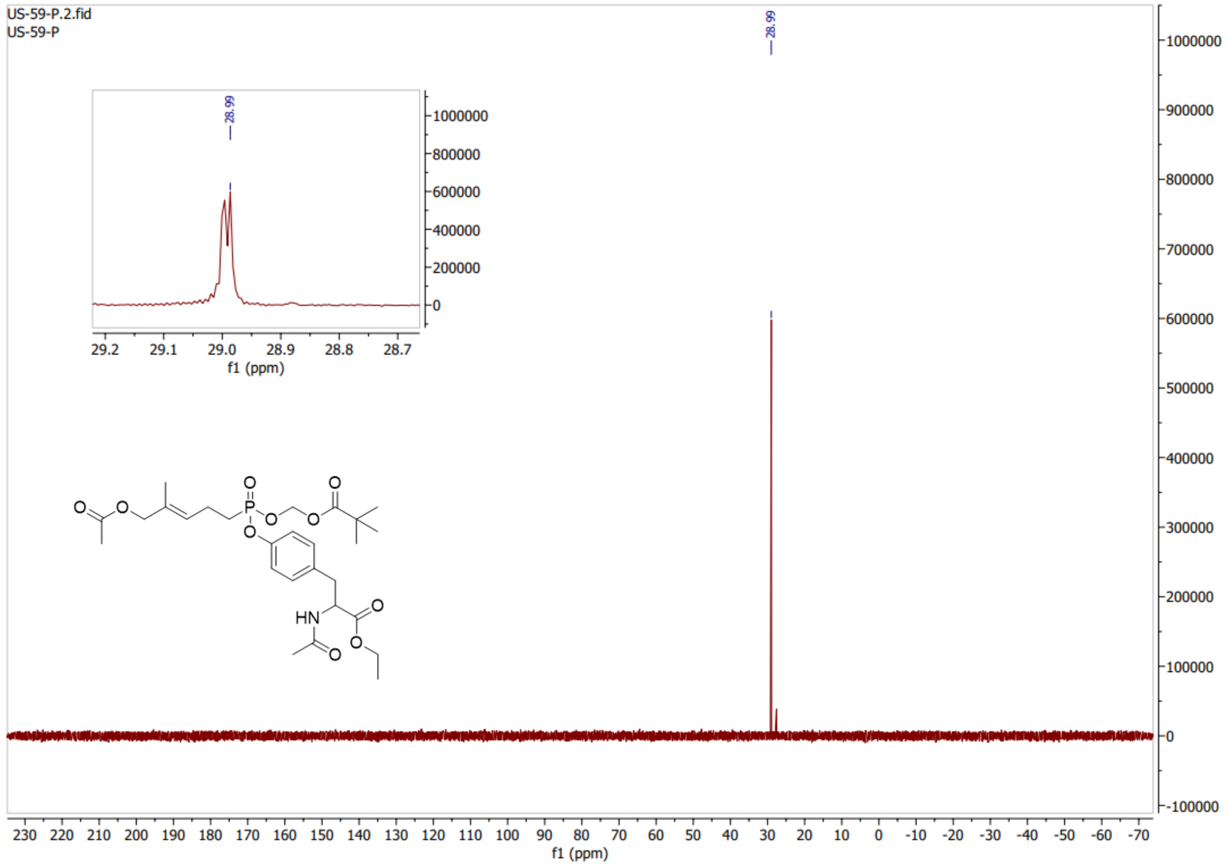

^{31}P NMR Spectrum of Compound **9e** ($CDCl_3$, 203 MHz)

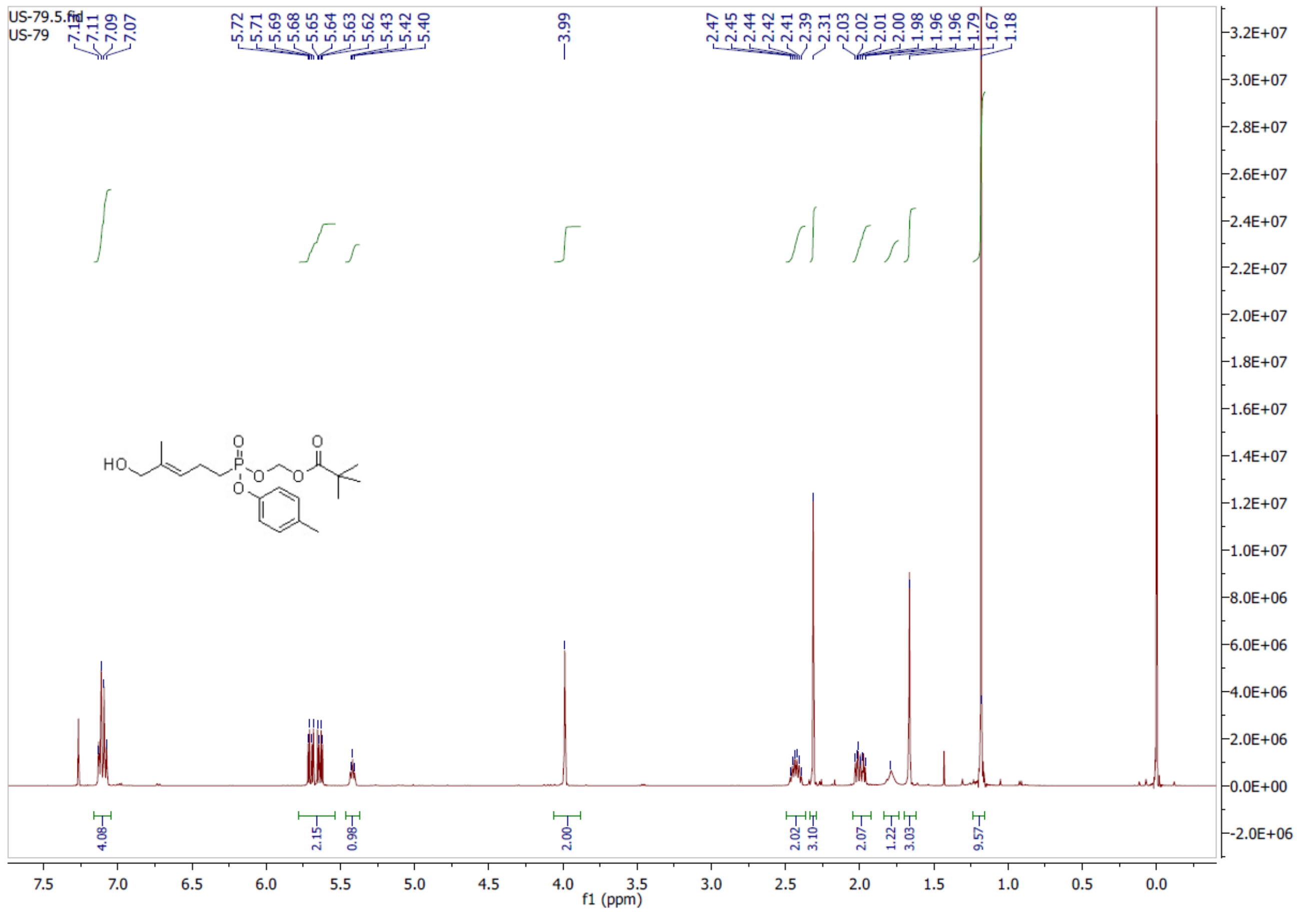

^{1}H NMR Spectrum of Compound **8f** ($CDCl_3$, 500 MHz)

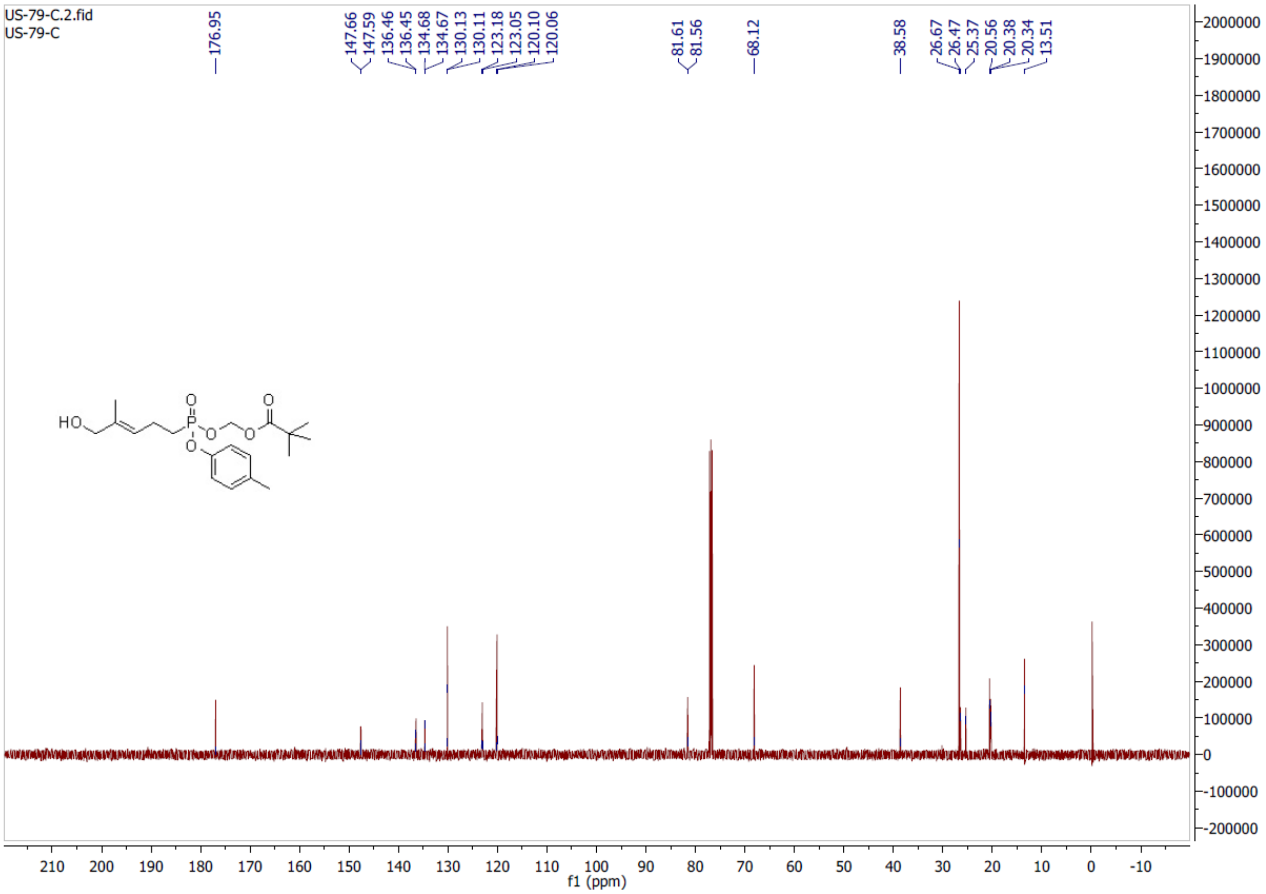

^{13}C NMR Spectrum of Compound **8f** ($CDCl_3$, 126 MHz)

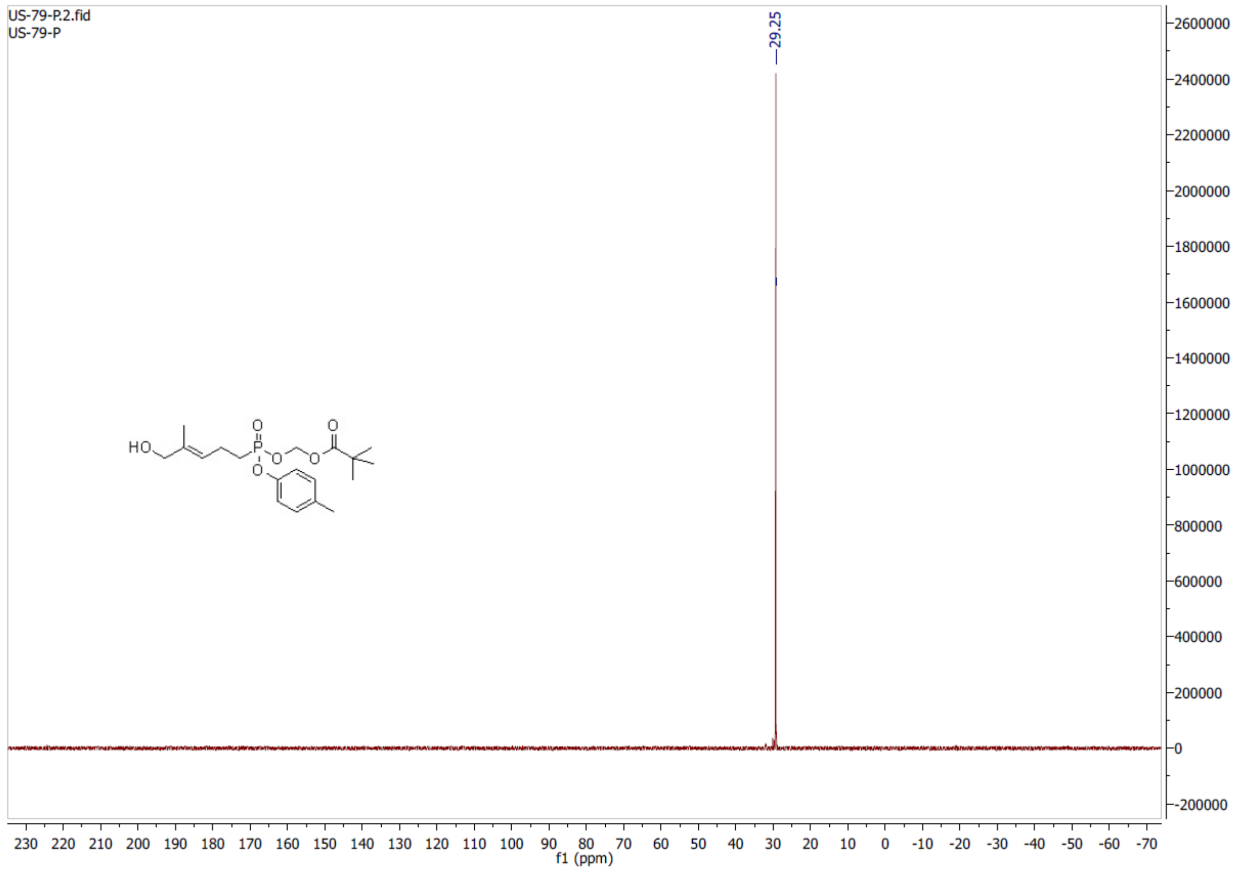

^{31}P NMR Spectrum of Compound **8f** ($CDCl_3$, 203 MHz)

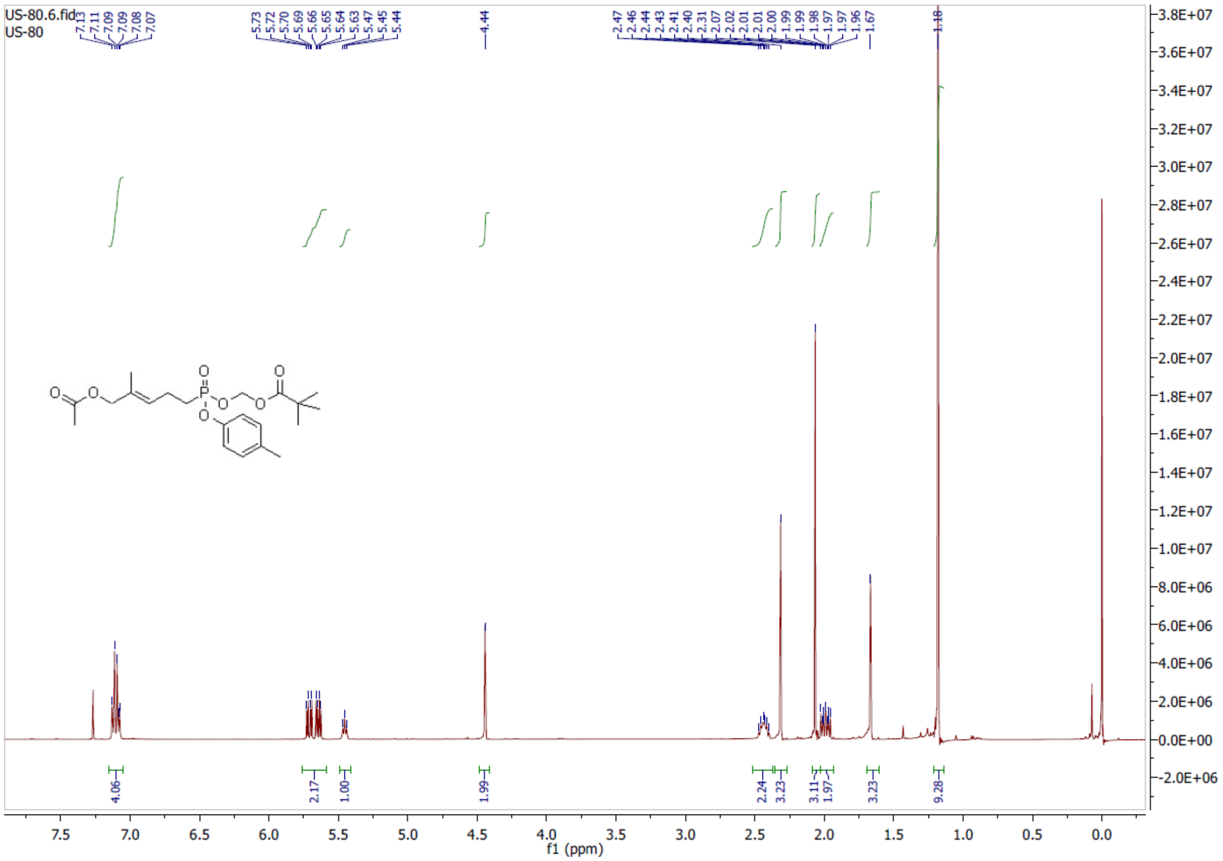

^{1}H NMR Spectrum of Compound **9f** ($CDCl_3$, 500 MHz)

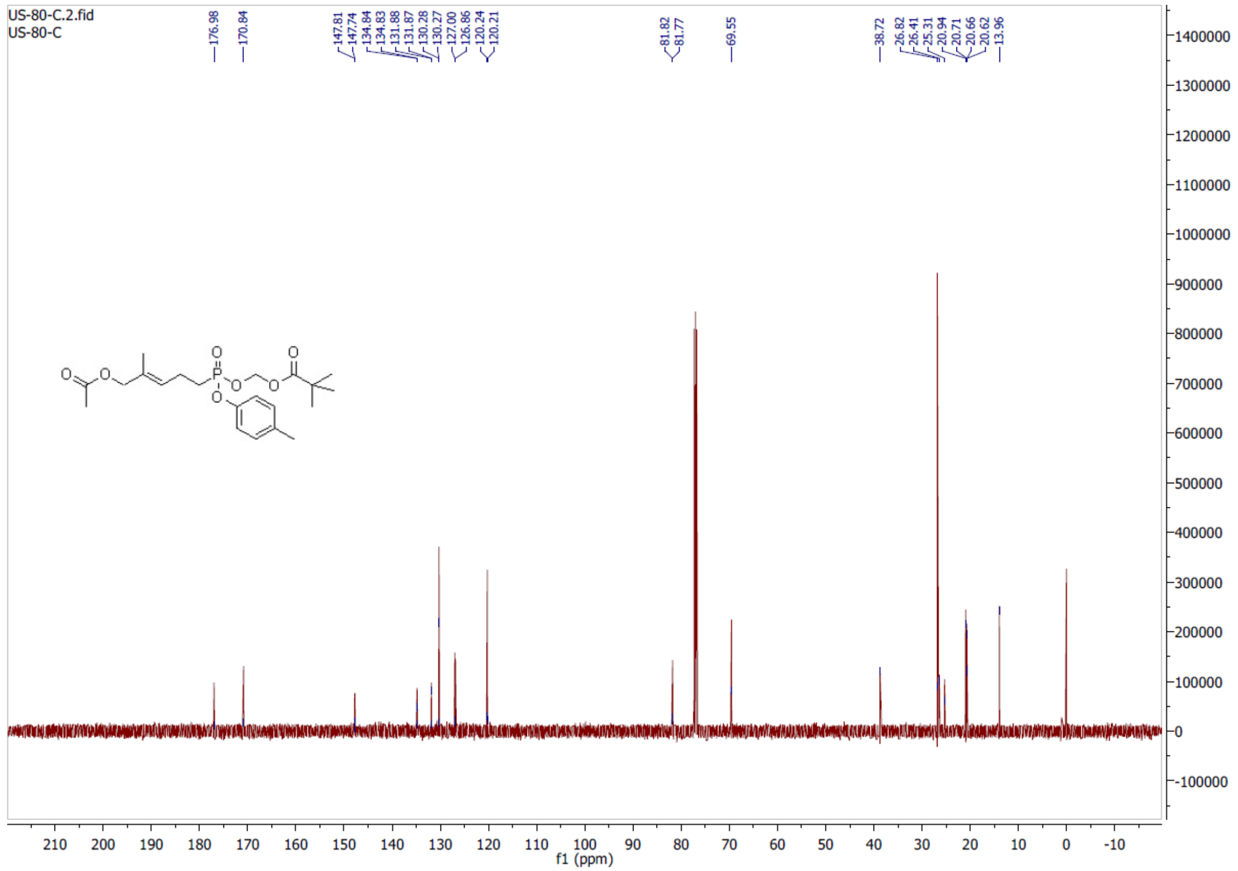

^{13}C NMR Spectrum of Compound **9f** ($CDCl_3$, 126 MHz)

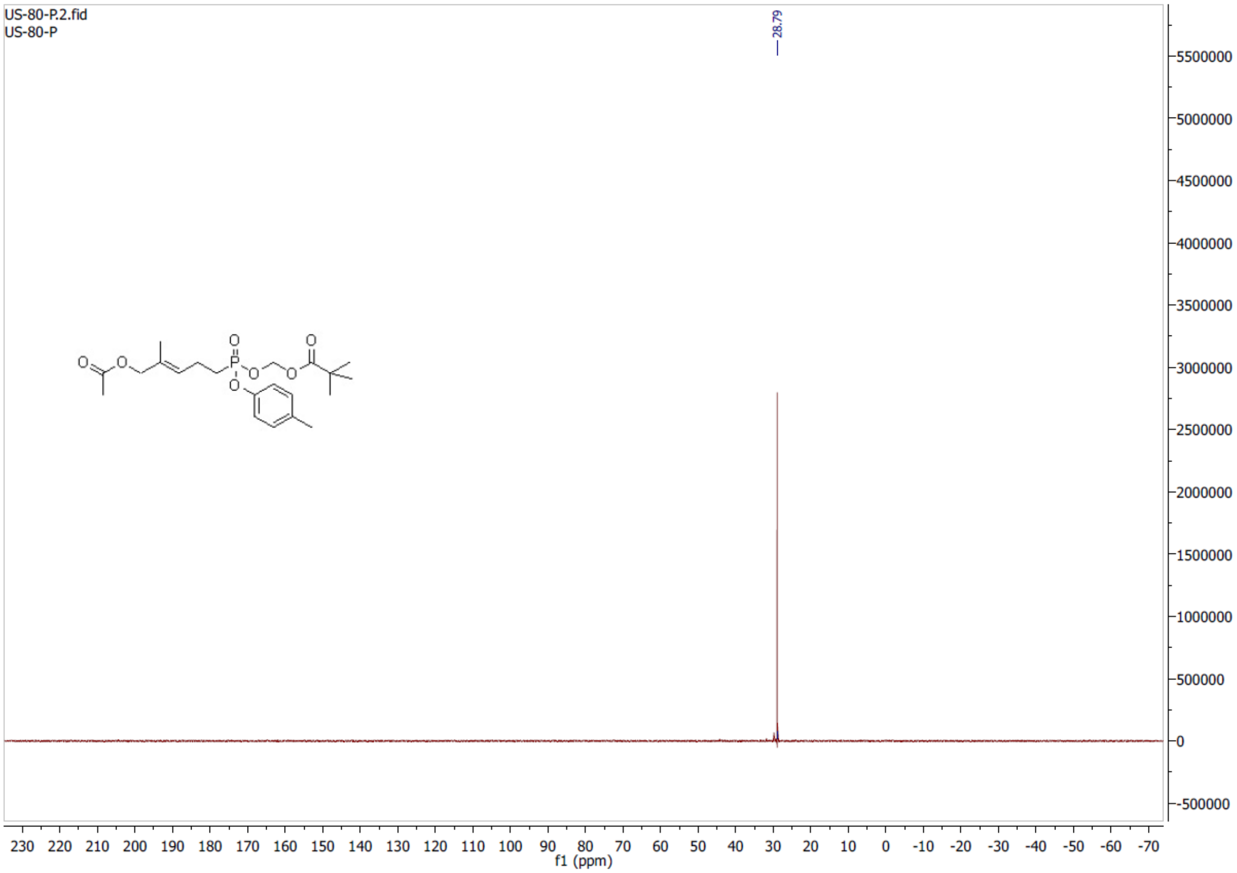

^{31}P NMR Spectrum of Compound **9f** ($CDCl_3$, 203 MHz)

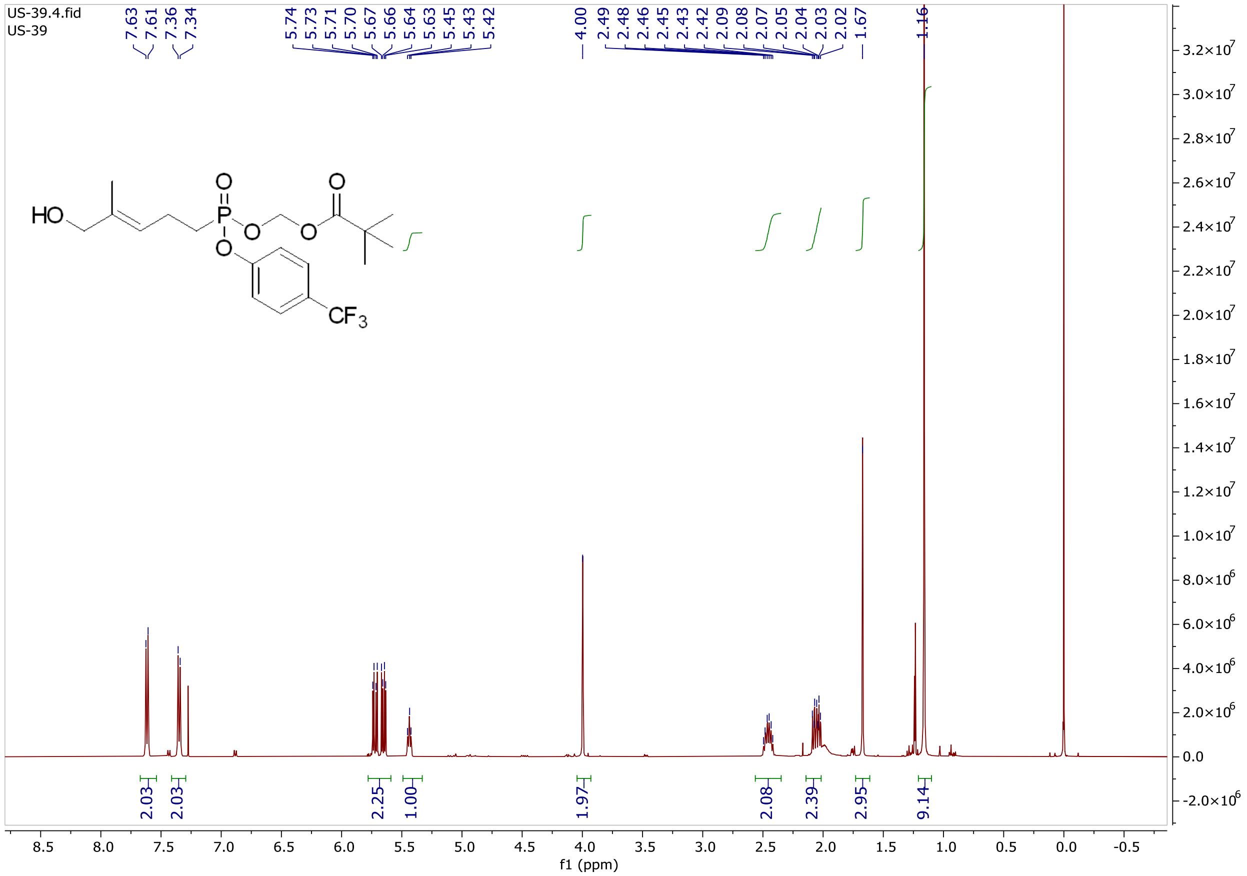

^{1}H NMR Spectrum of Compound **8g** ($CDCl_3$, 500 MHz)

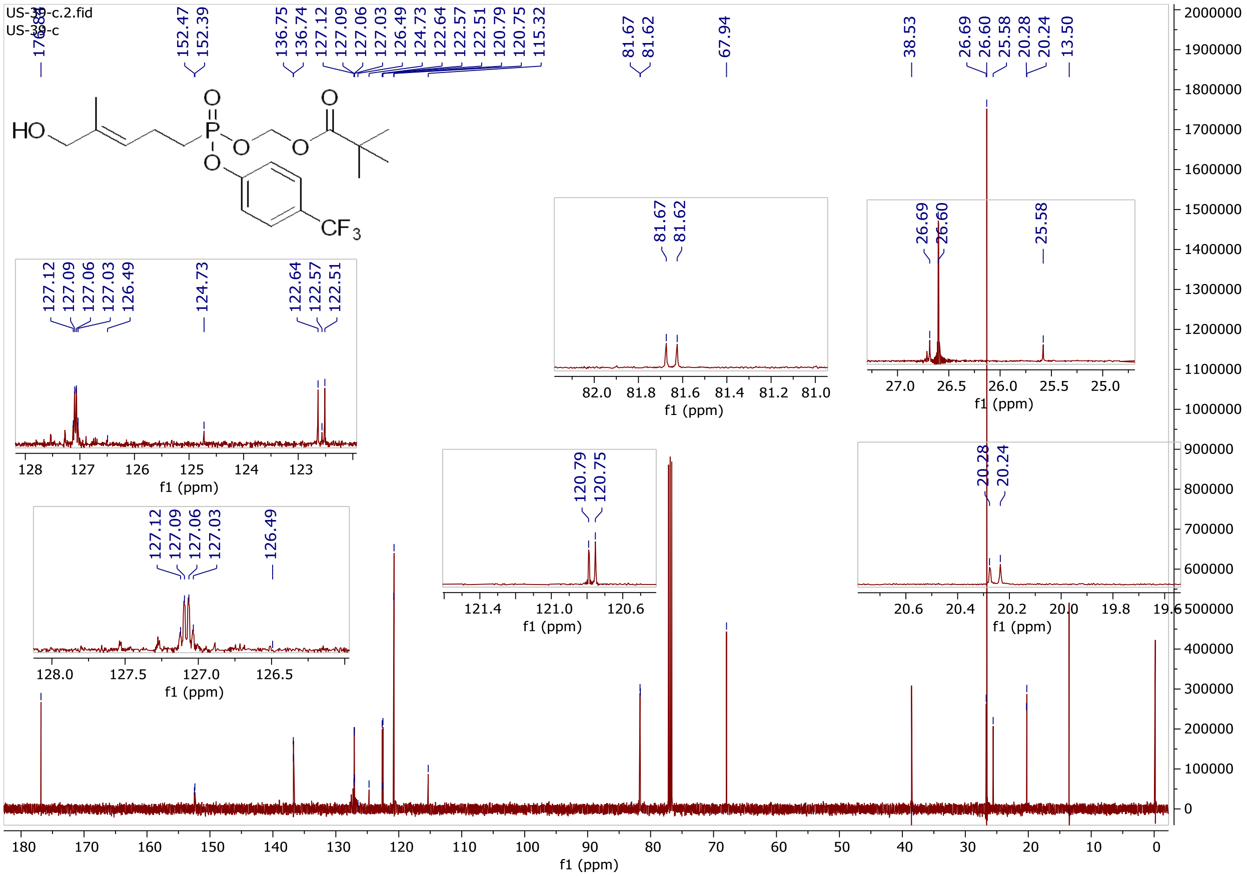

^{13}C NMR Spectrum of Compound **8g** ($CDCl_3$, 126 MHz)

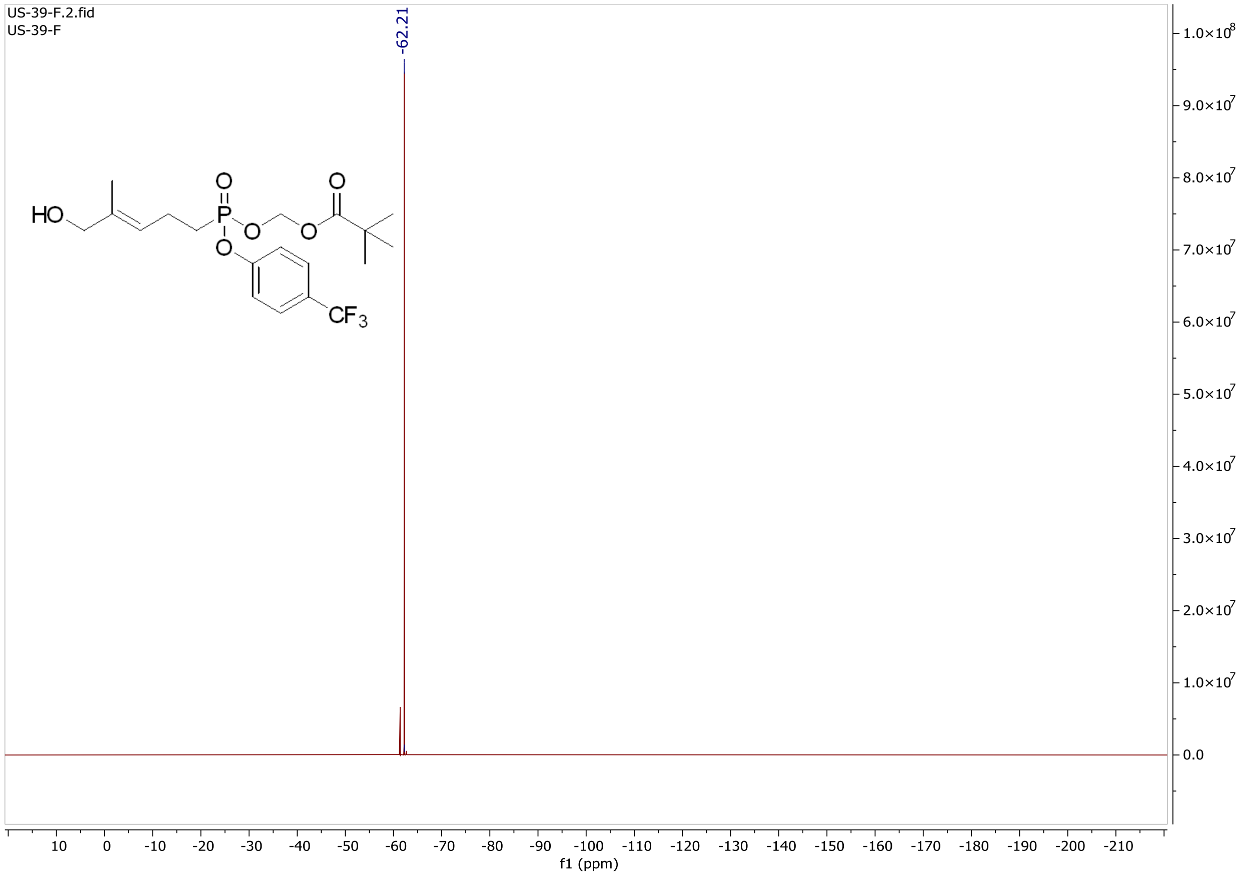

^{19}F NMR Spectrum of Compound **8g** ($CDCl_3$, 471 MHz)

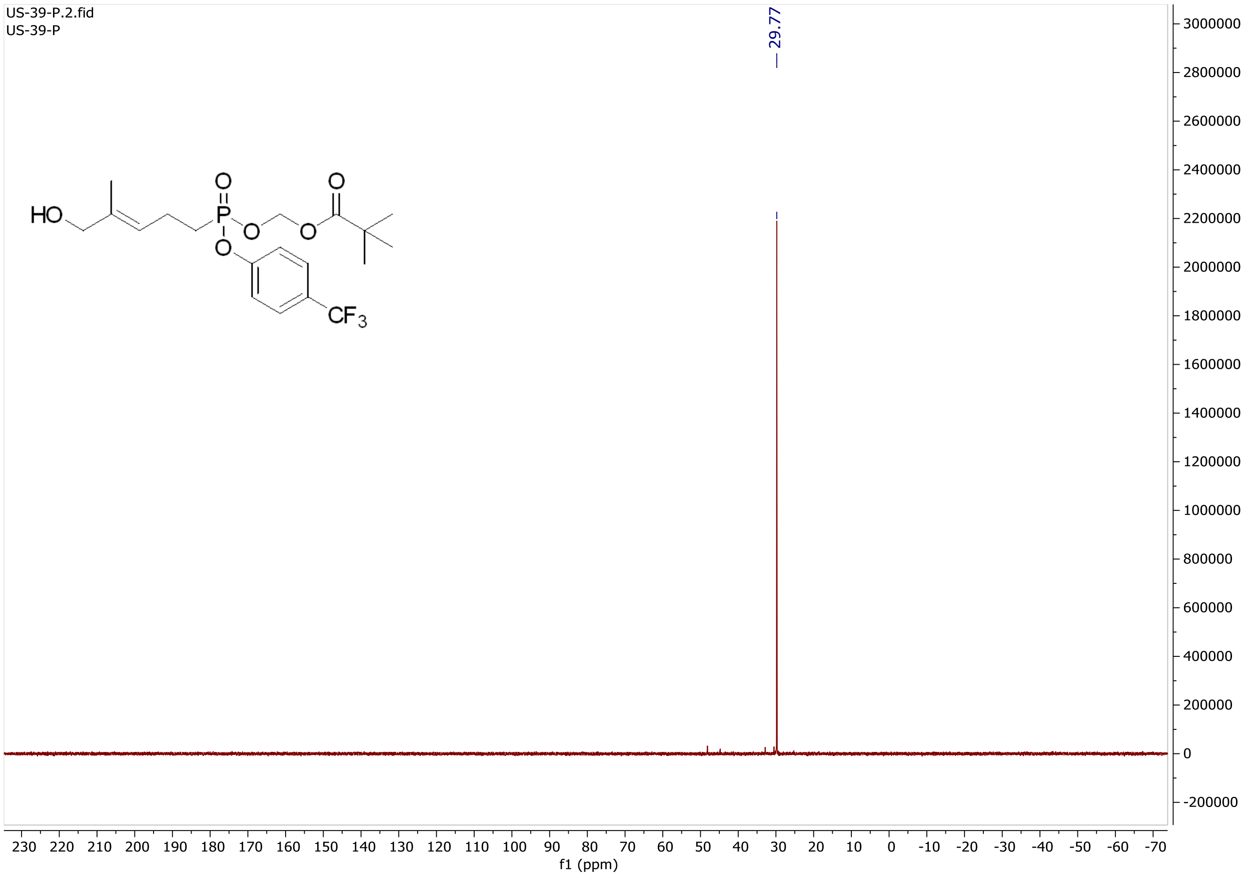

^{31}P NMR Spectrum of Compound **8g** ($CDCl_3$, 203 MHz)

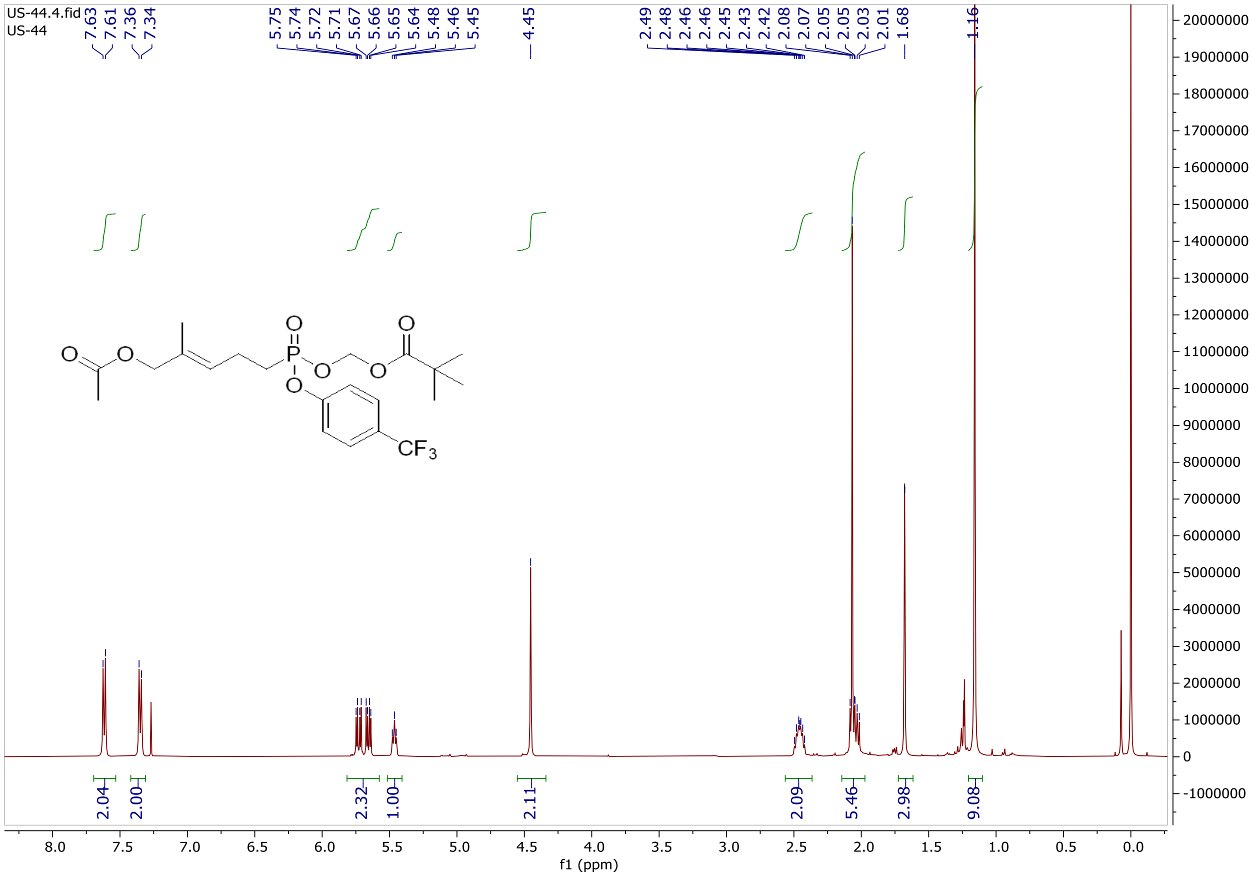

^{1}H NMR Spectrum of Compound **9g** ($CDCl_3$, 500 MHz)

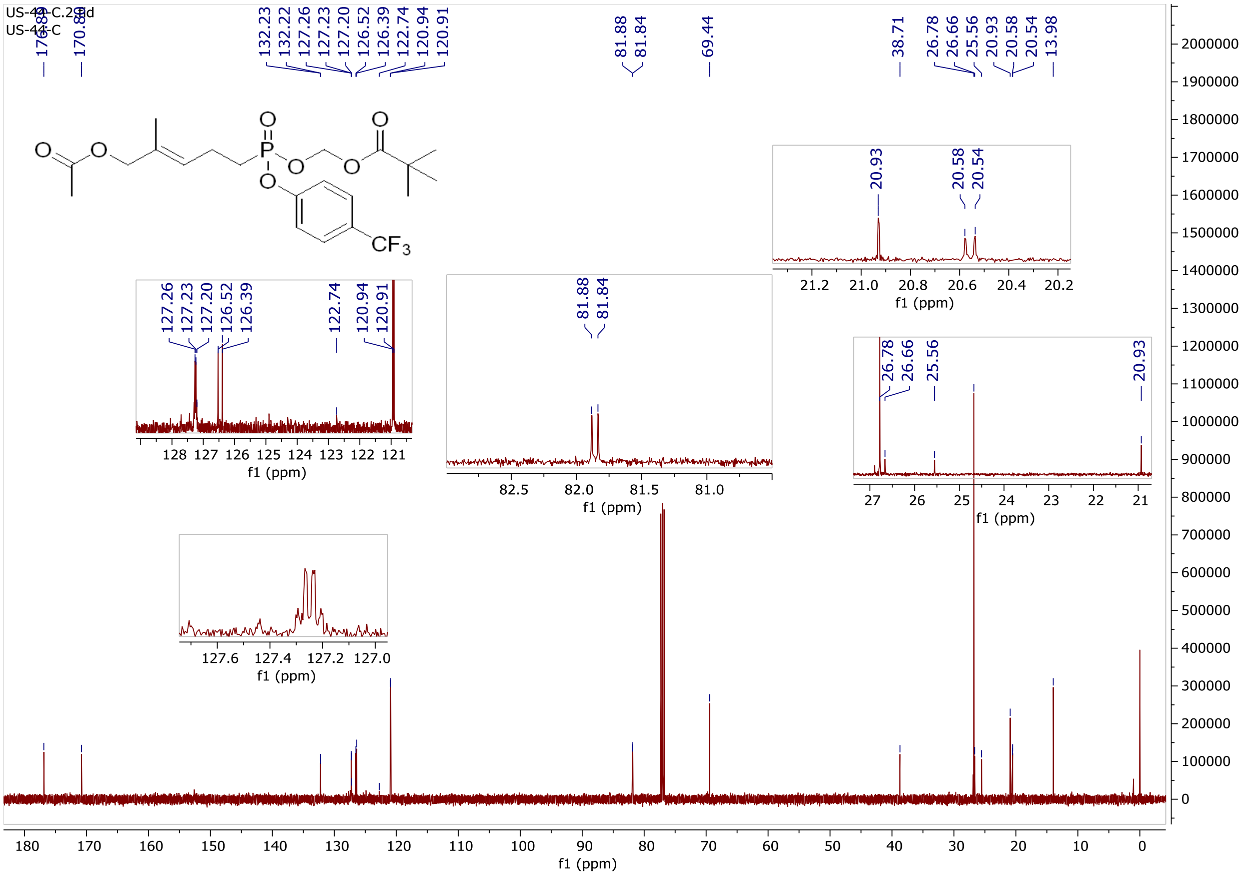

^{13}C NMR Spectrum of Compound **9g** ($CDCl_3$, 126 MHz)

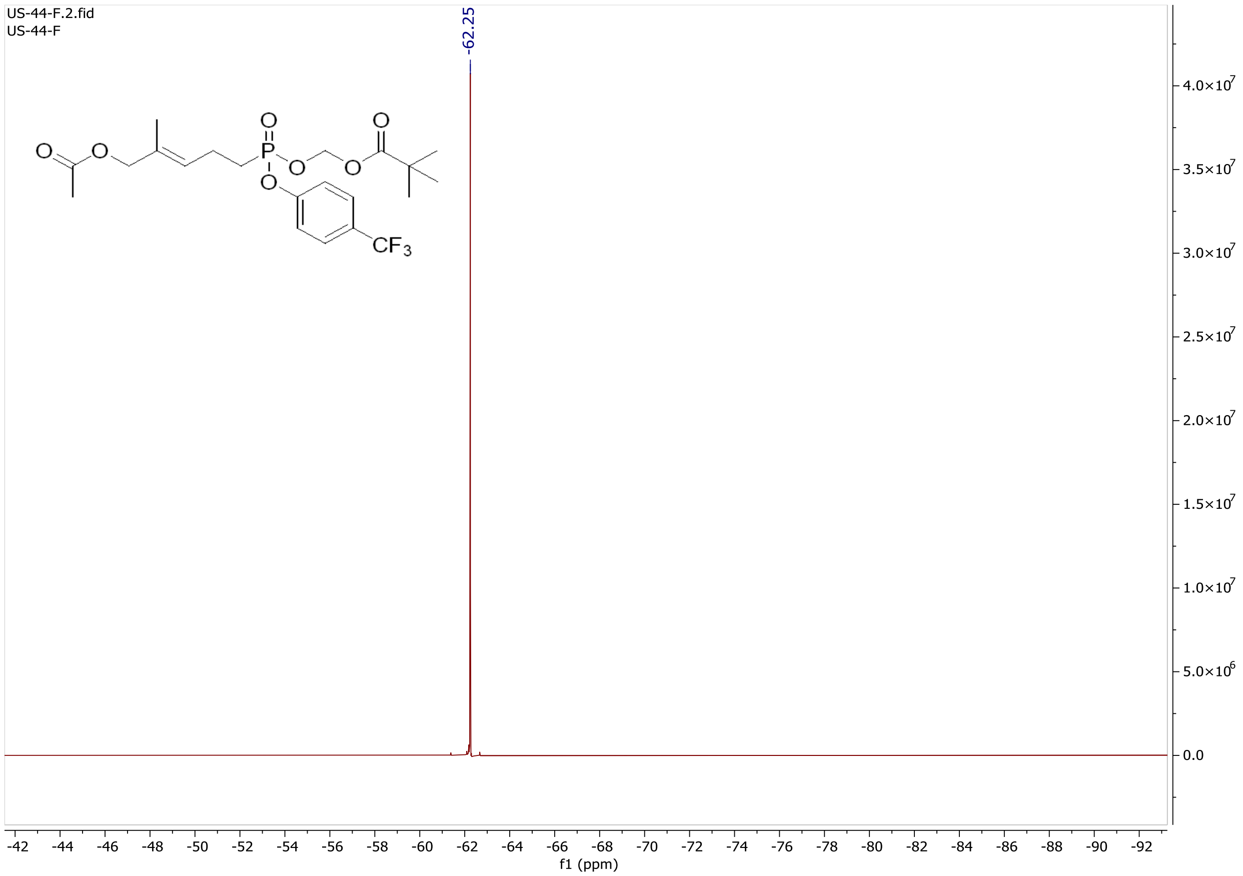

^{19}F NMR Spectrum of Compound **9g** ($CDCl_3$, 471 MHz)

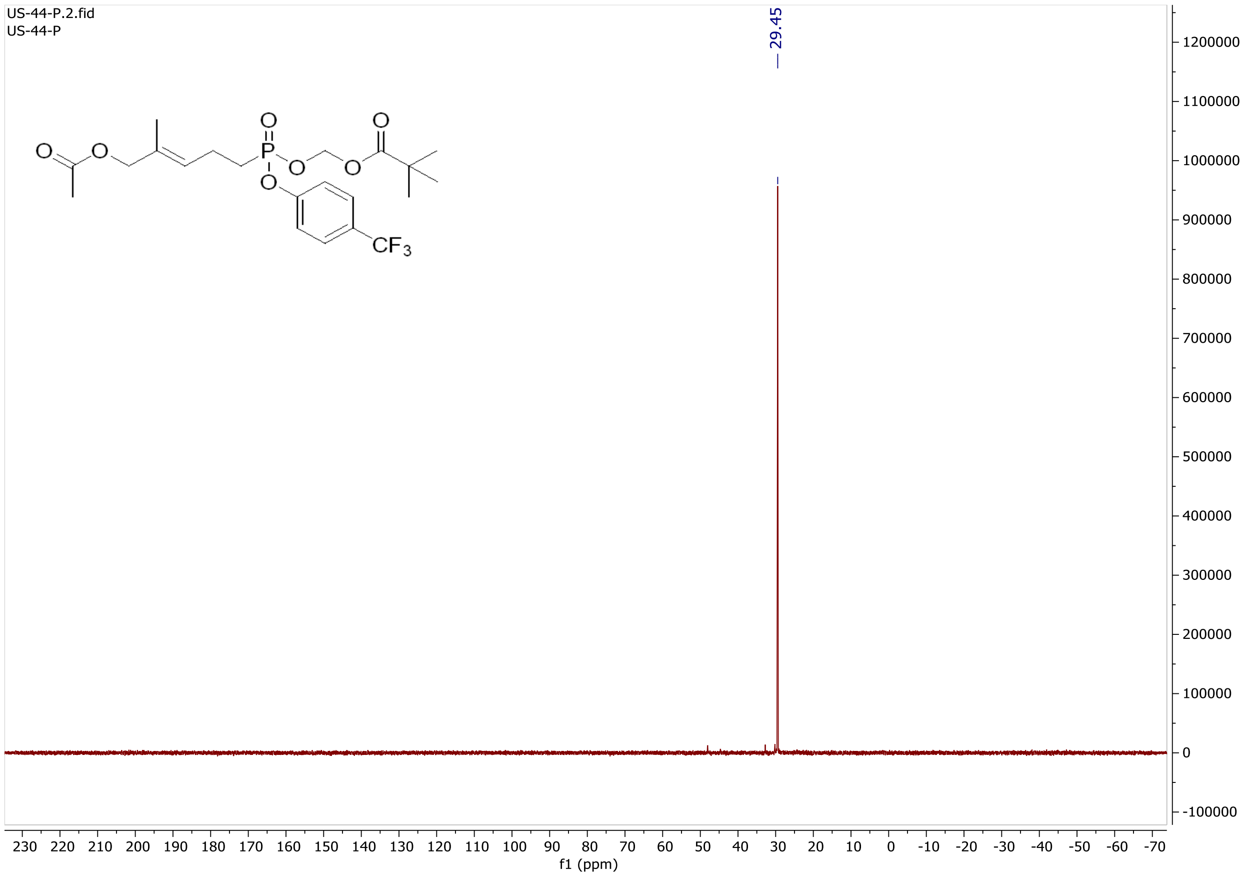

^{31}P NMR Spectrum of Compound **9g** ($CDCl_3$, 203 MHz)

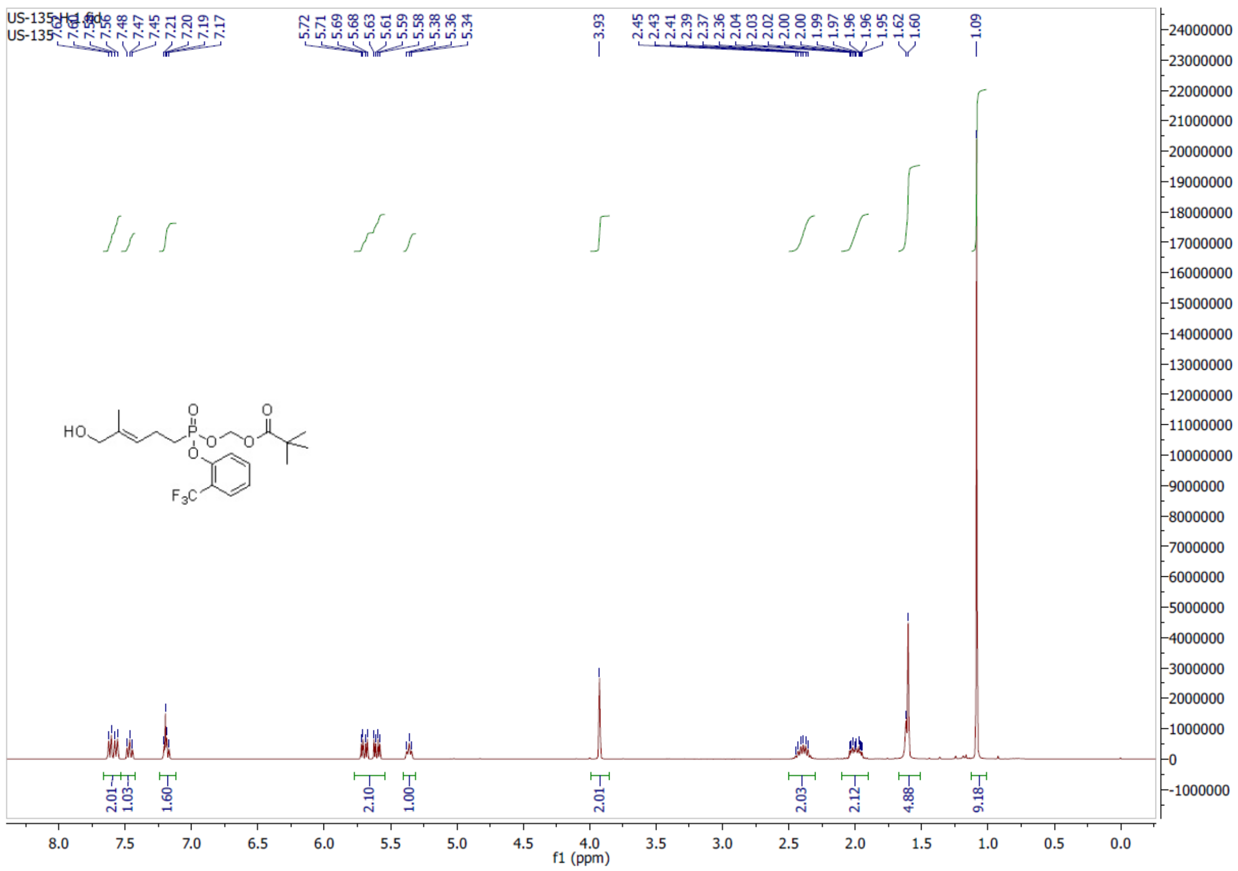

^{1}H NMR Spectrum of Compound **8h** ($CDCl_3$, 400 MHz)

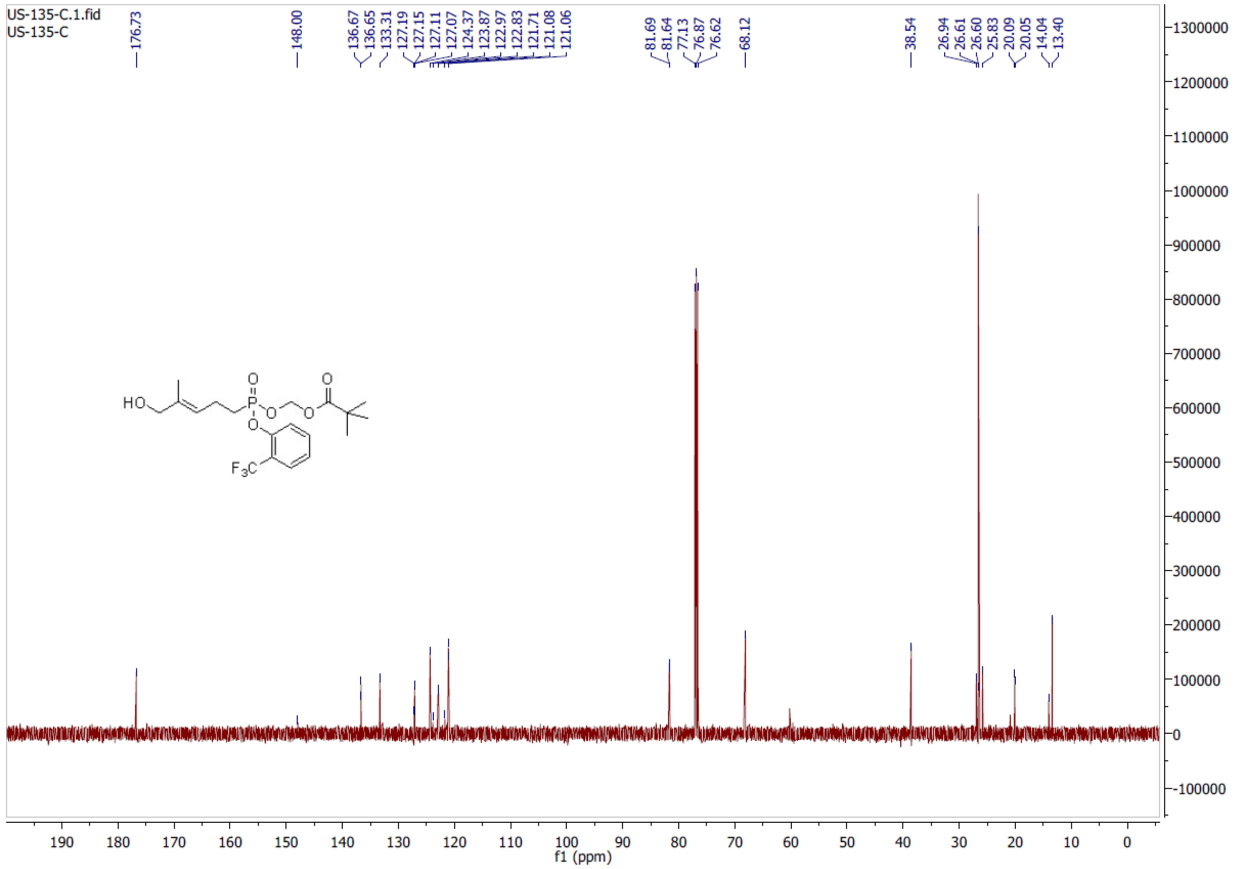

^{13}C NMR Spectrum of Compound **8h** ($CDCl_3$, 126 MHz)

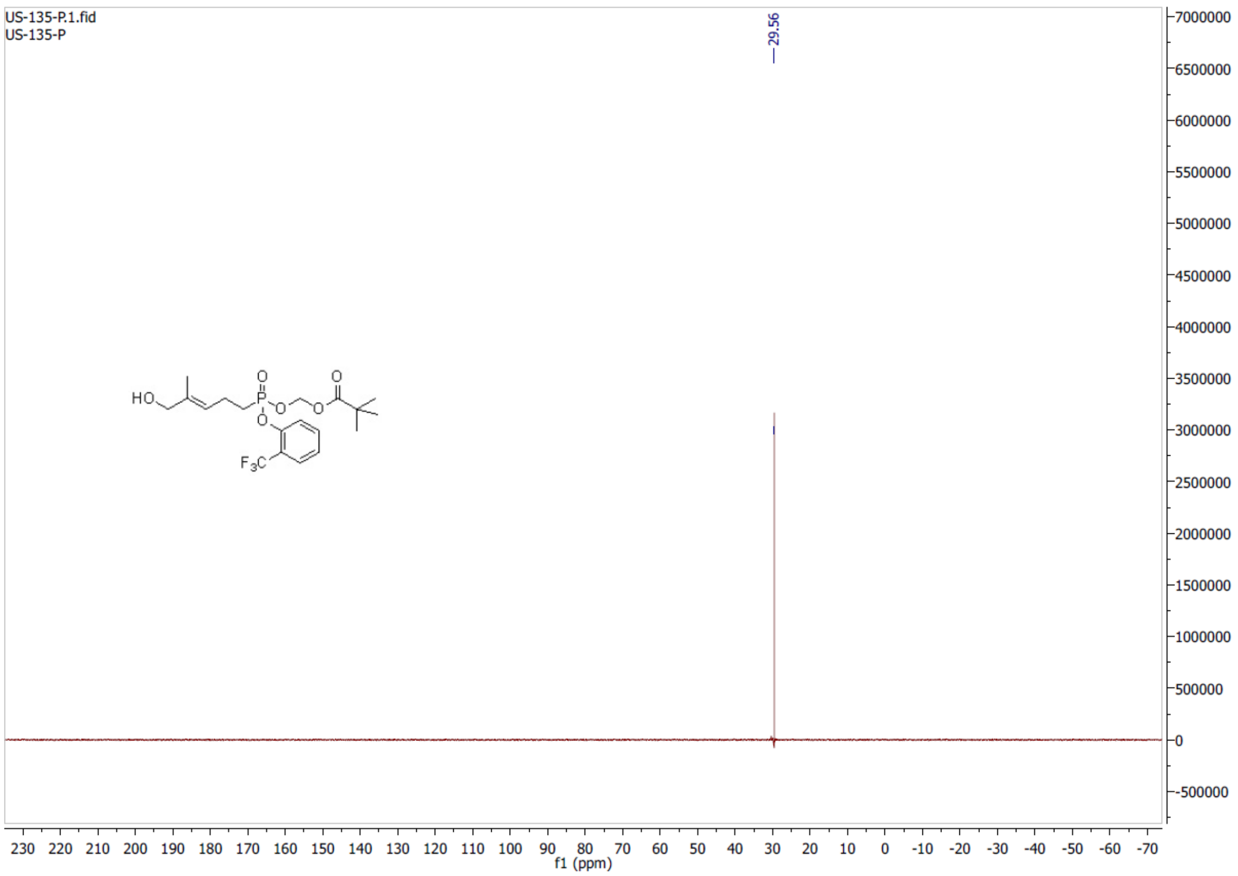

^{31}P NMR Spectrum of Compound **8h** ($CDCl_3$, 162 MHz)

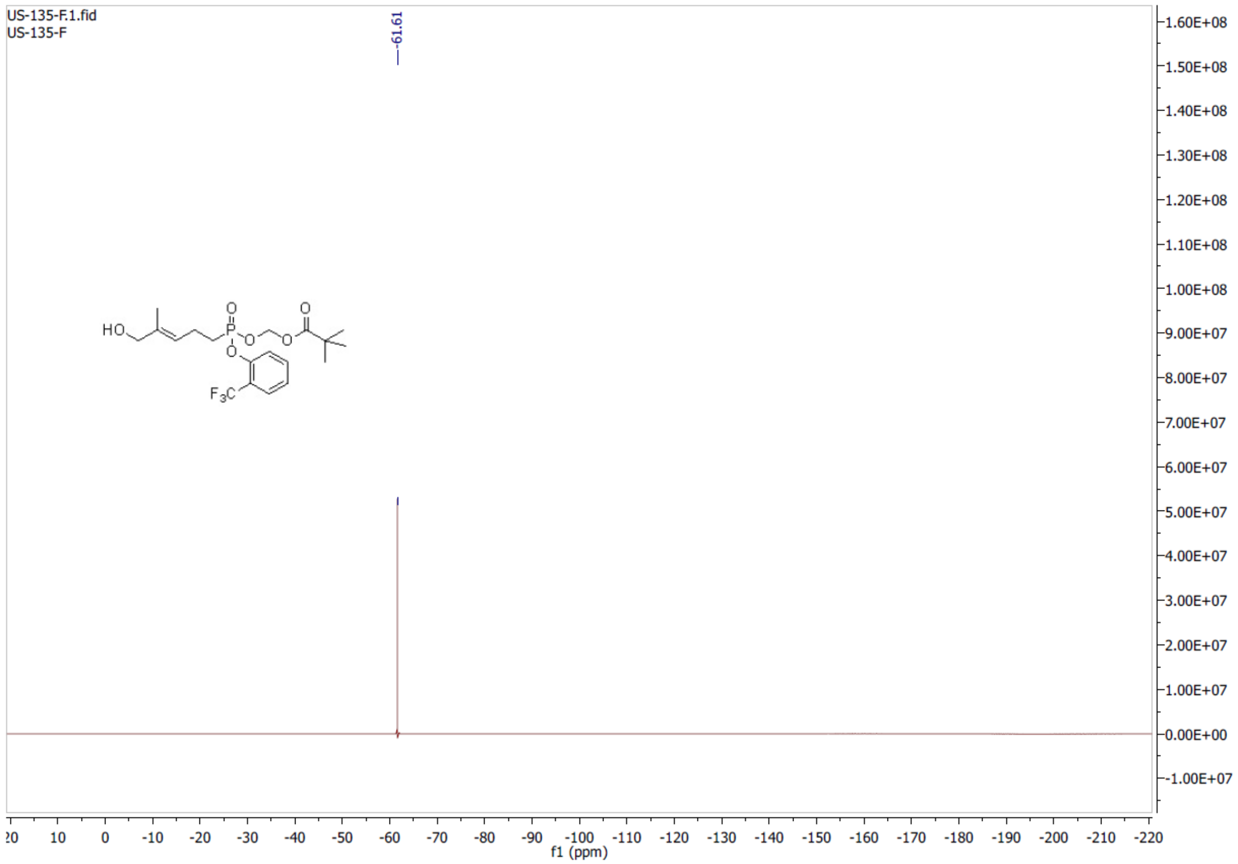

^{19}F NMR Spectrum of Compound **8h** ($CDCl_3$, 471 MHz)

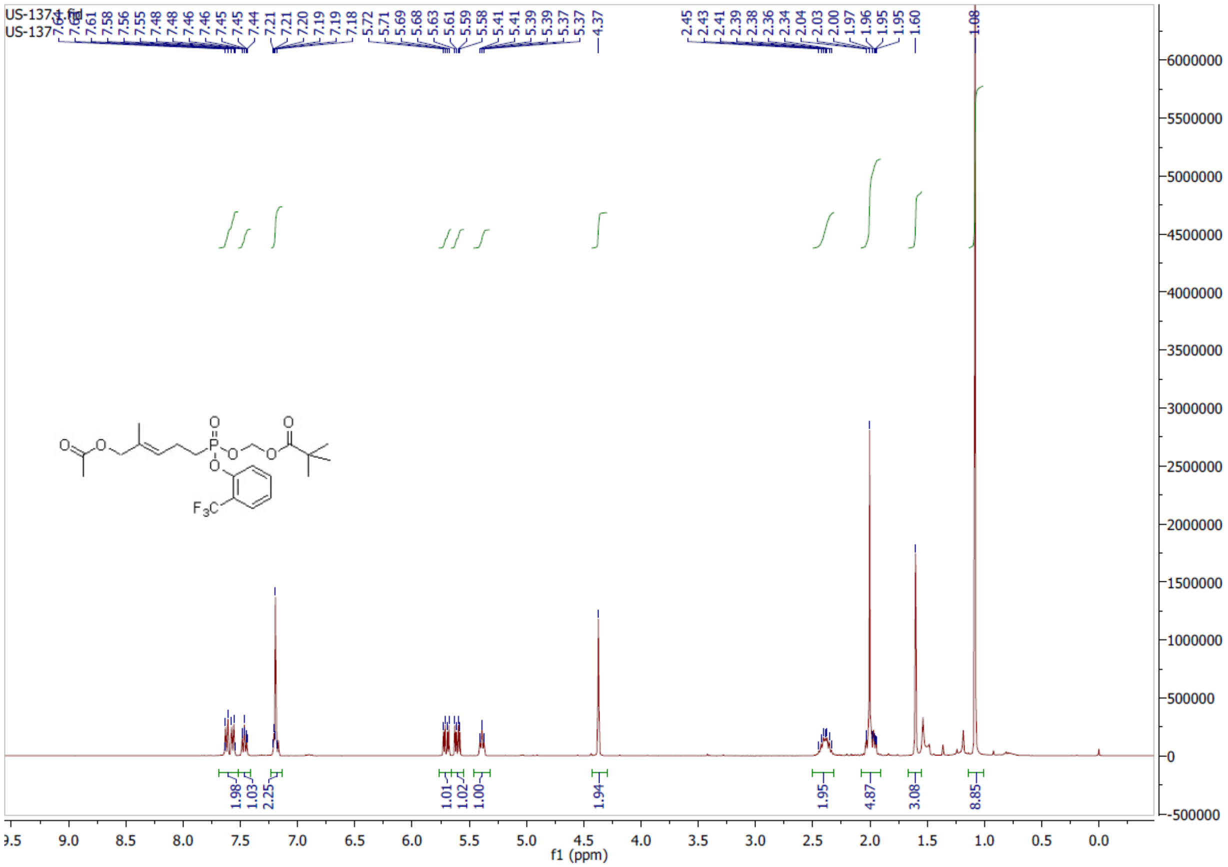

^{1}H NMR Spectrum of Compound **9h** ($CDCl_3$, 400 MHz)

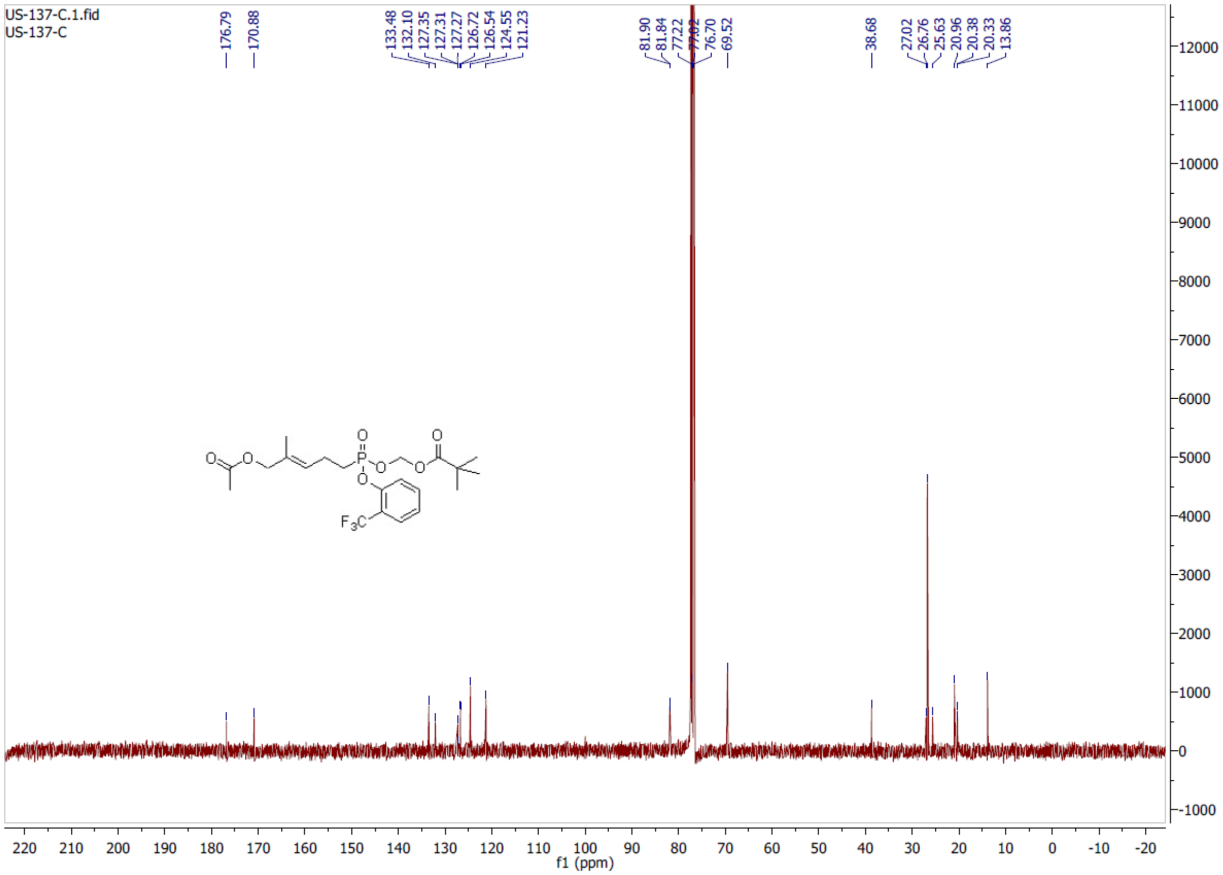

^{13}C NMR Spectrum of Compound **9h** ($CDCl_3$, 126 MHz)

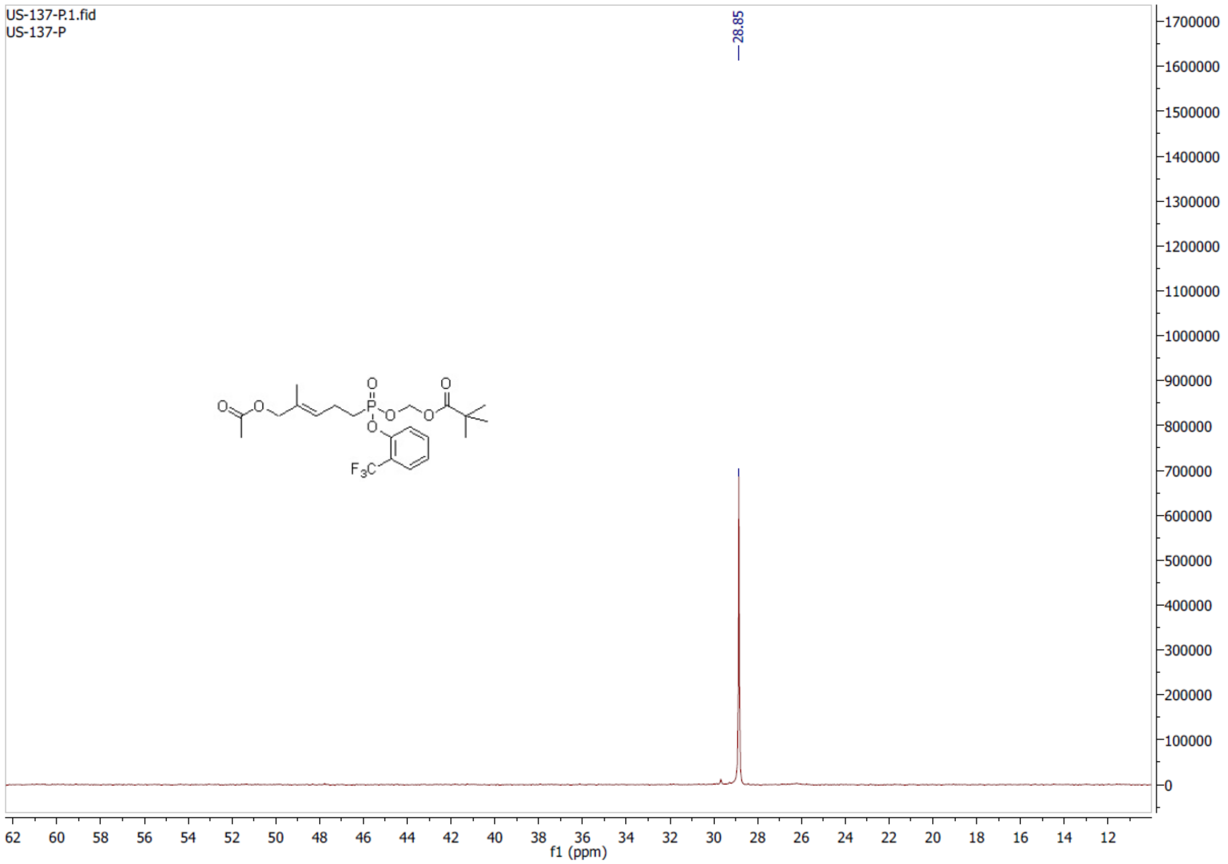

^{31}P NMR Spectrum of Compound **9h** ($CDCl_3$, 162 MHz)

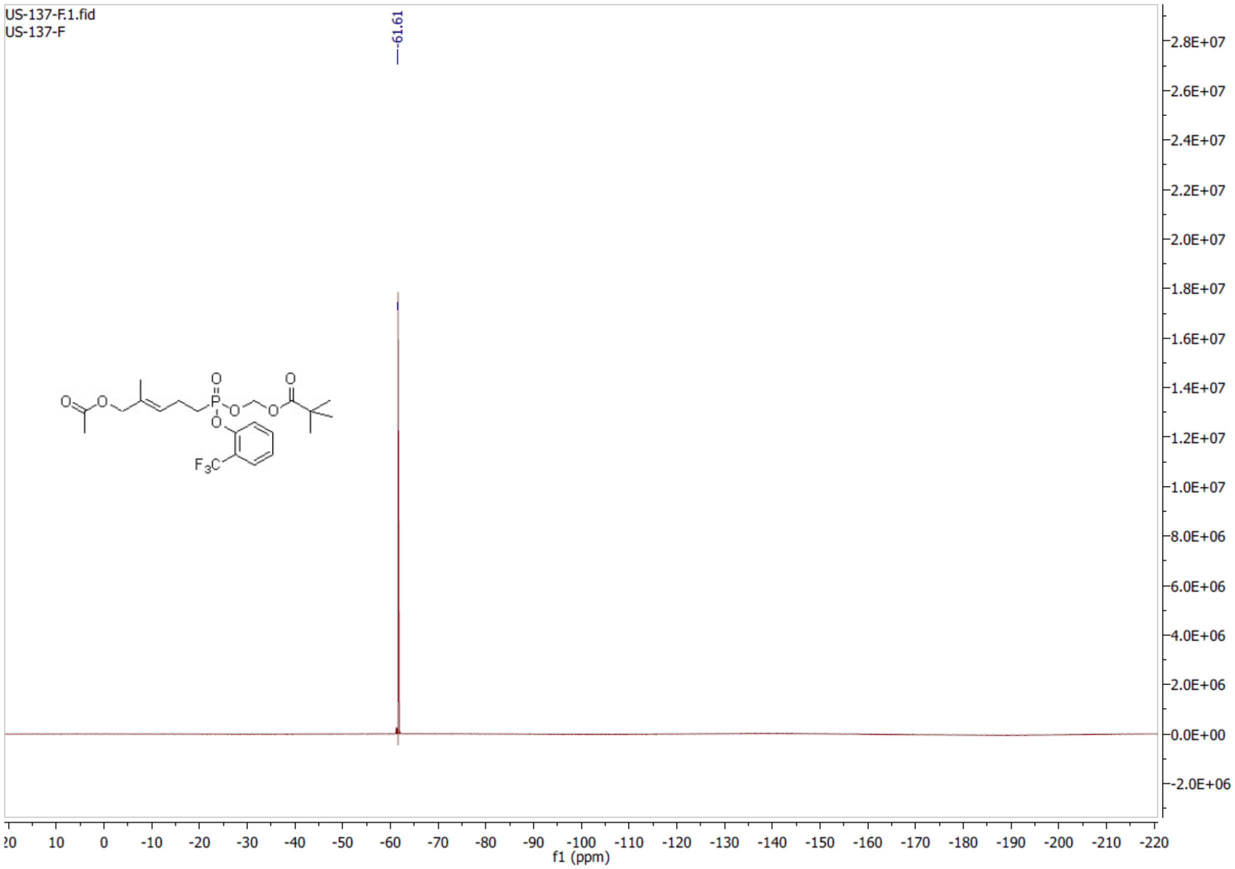

^{19}F NMR Spectrum of Compound **9h** ($CDCl_3$, 471 MHz)

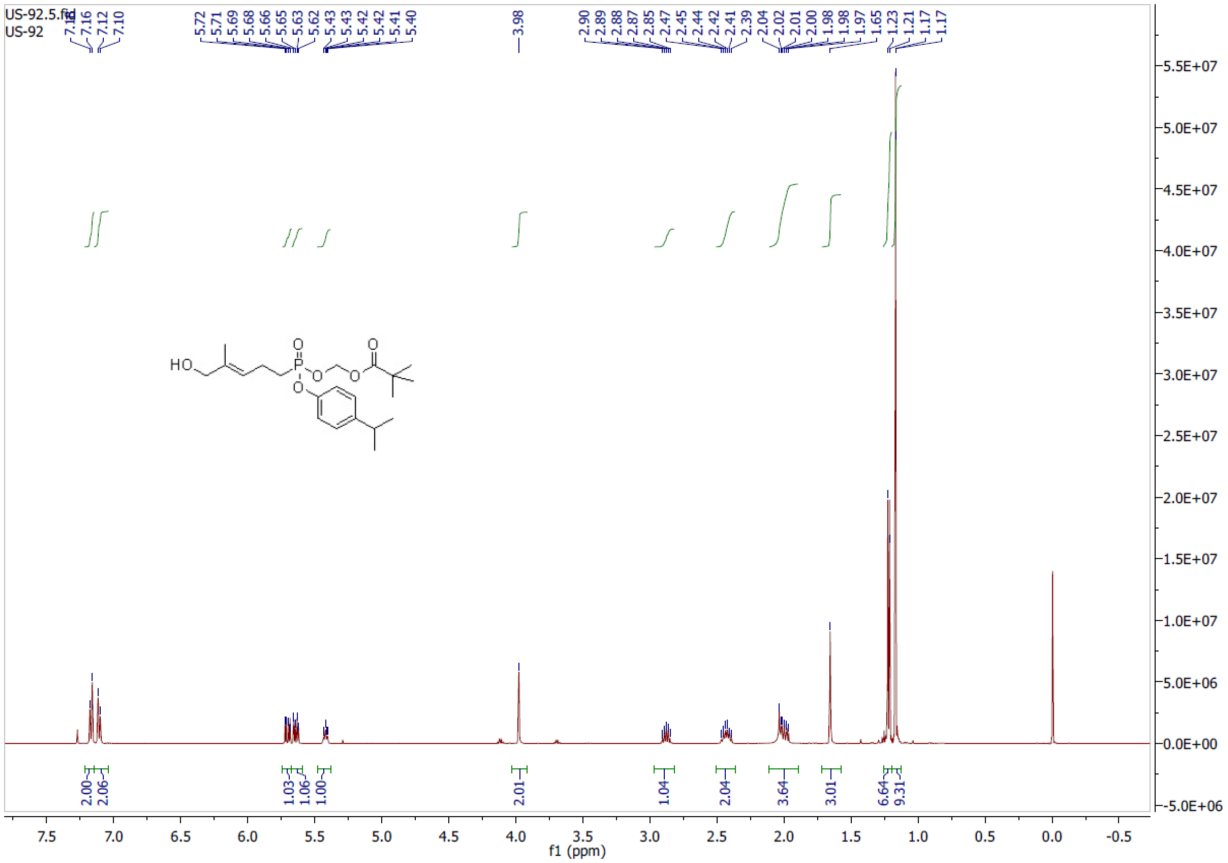

^{1}H NMR Spectrum of Compound **8i** ($CDCl_3$, 500 MHz)

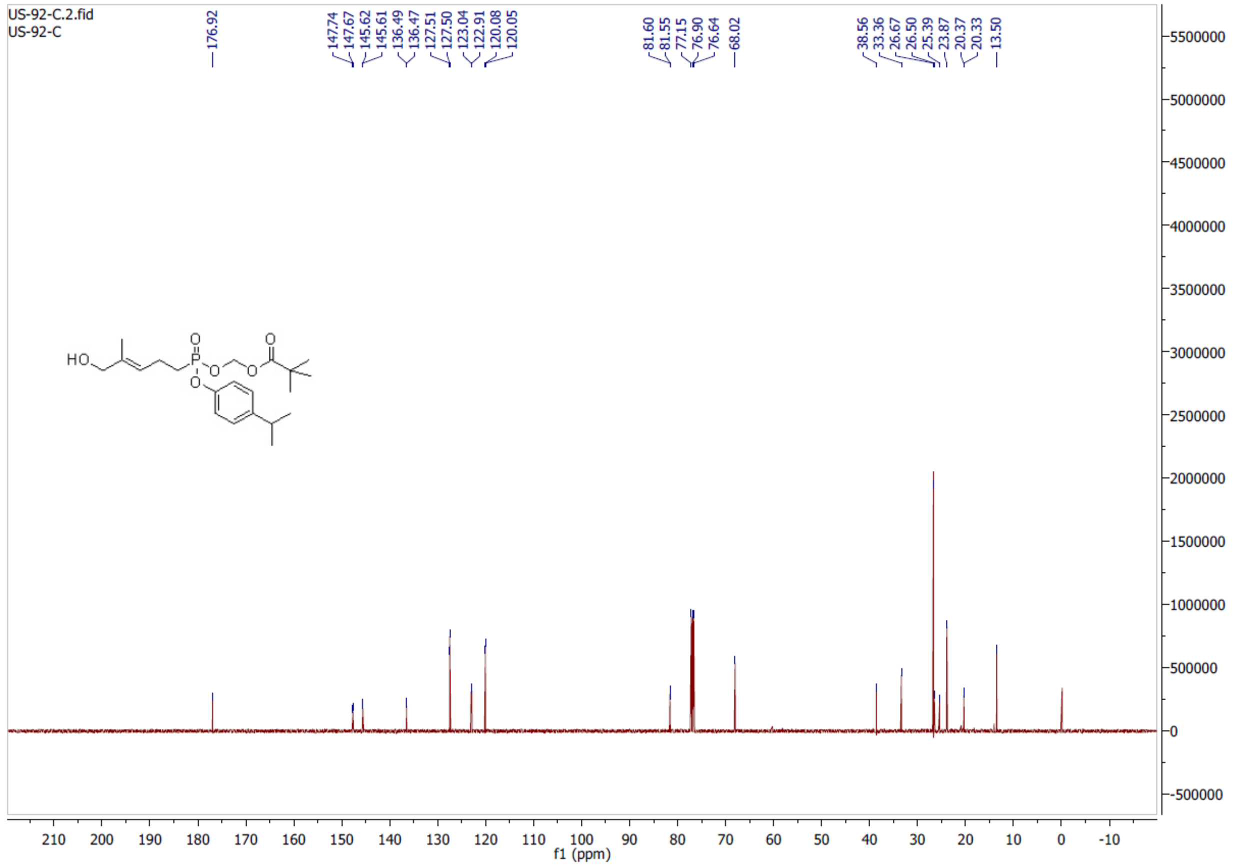

^{13}C NMR Spectrum of Compound **8i** ($CDCl_3$, 126 MHz)

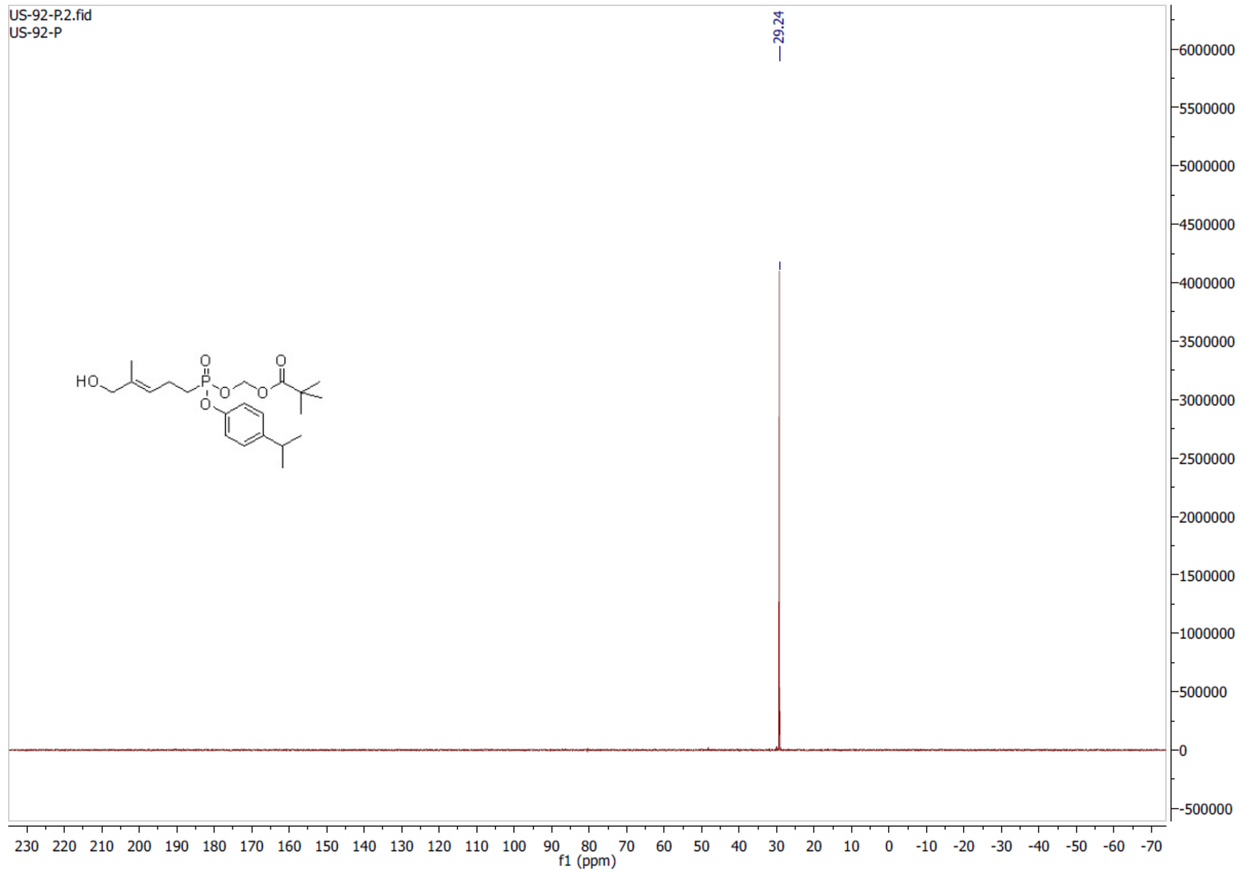

^{31}P NMR Spectrum of Compound **8i** ($CDCl_3$, 203 MHz)

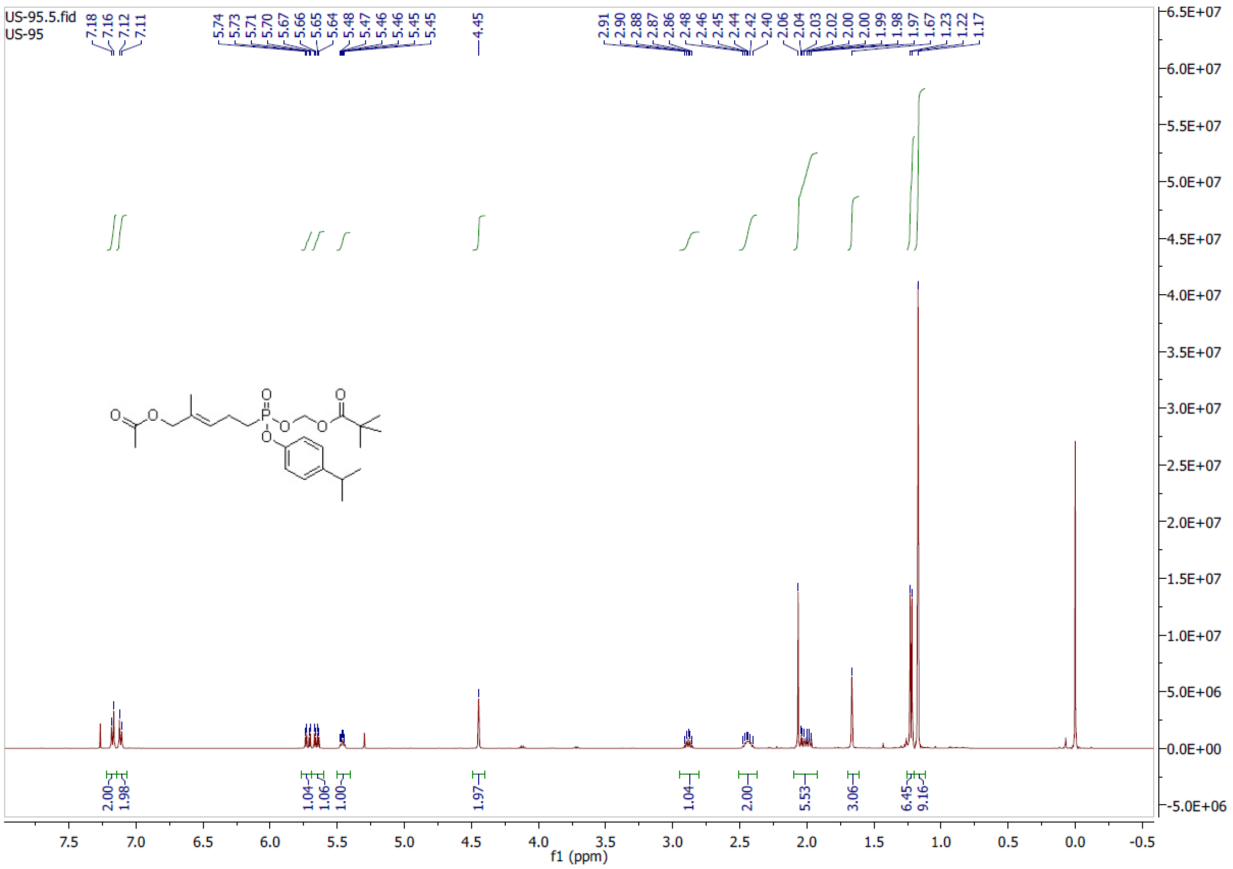

^{1}H NMR Spectrum of Compound **9i** ($CDCl_3$, 500 MHz)

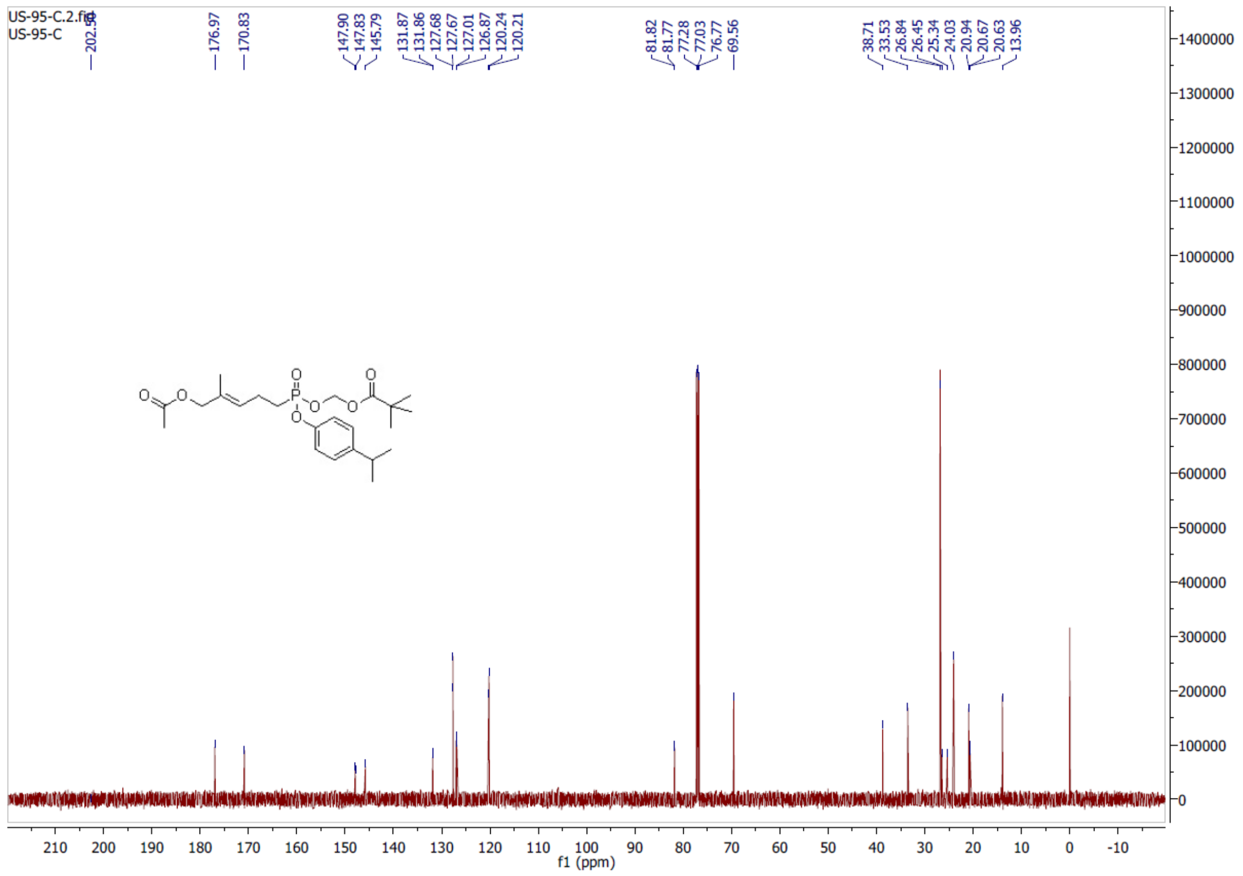

^{13}C NMR Spectrum of Compound **9i** ($CDCl_3$, 126 MHz)

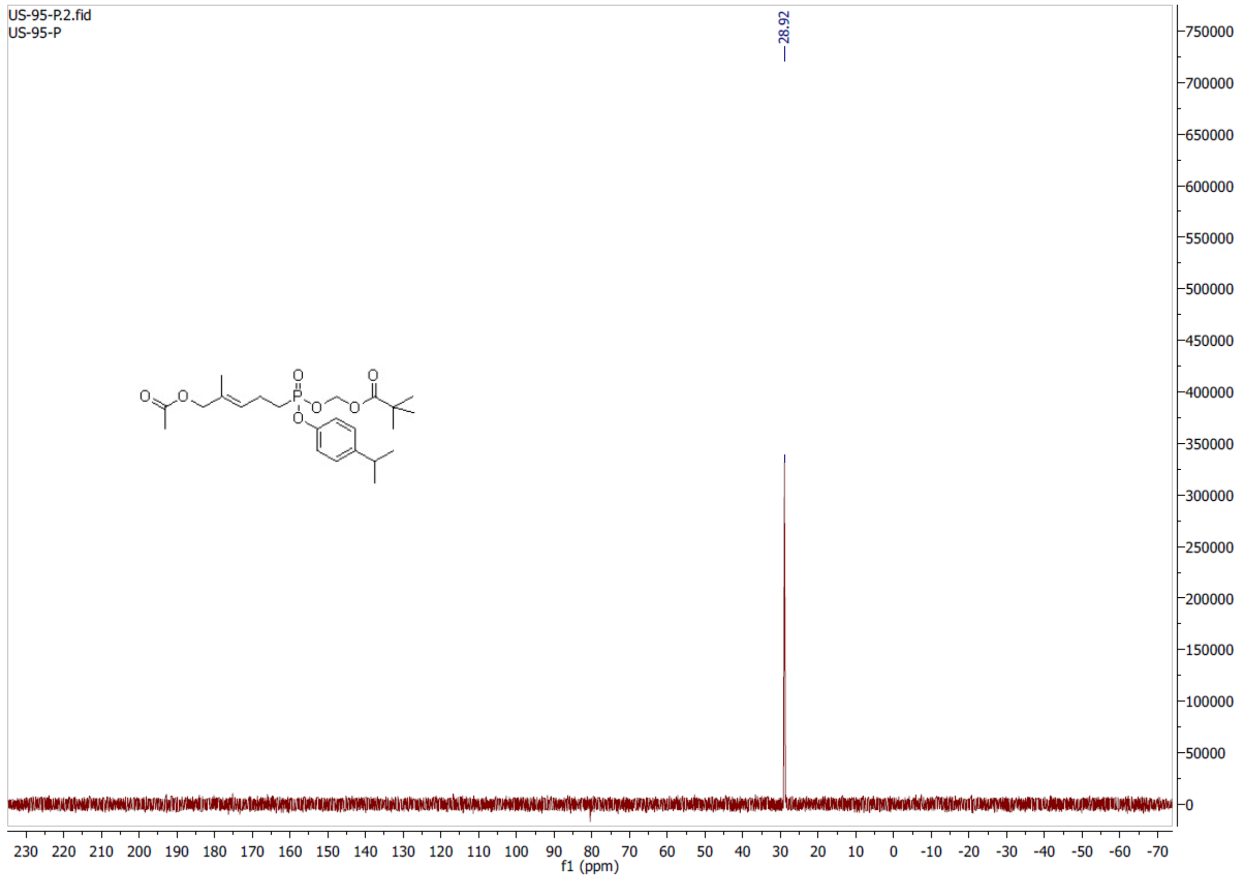

^{31}P NMR Spectrum of Compound **9i** ($CDCl_3$, 203 MHz)

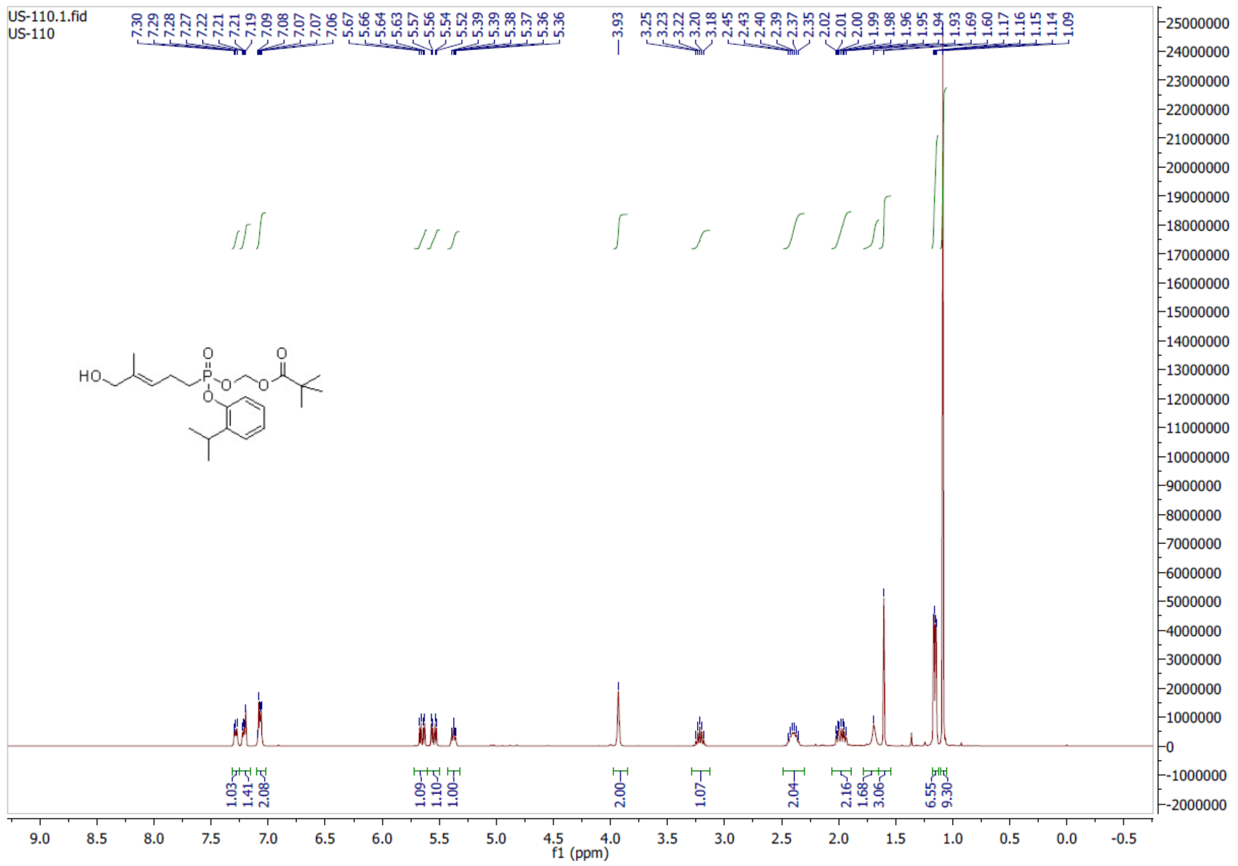

^{1}H NMR Spectrum of Compound **8j** ($CDCl_3$, 400 MHz)

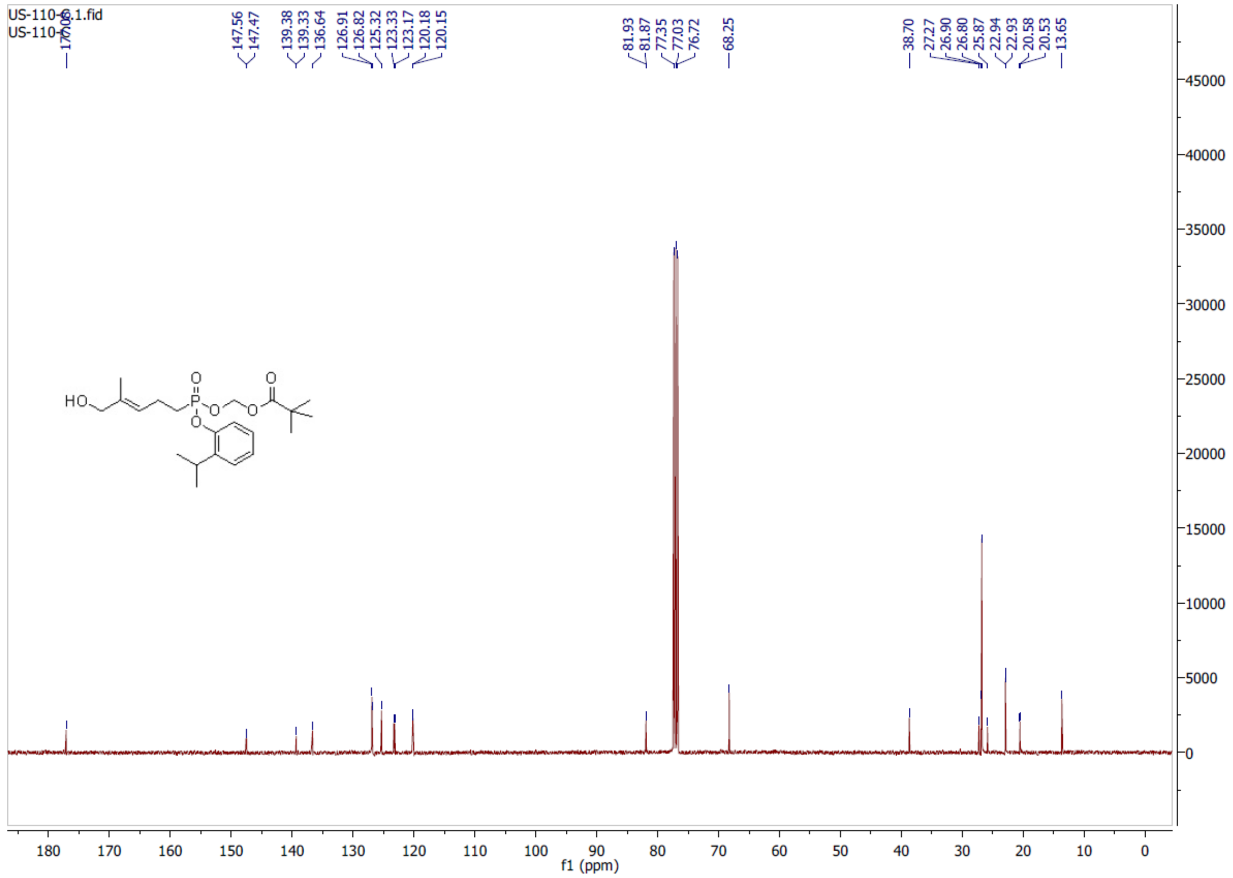

^{13}C NMR Spectrum of Compound **8j** ($CDCl_3$, 101 MHz)

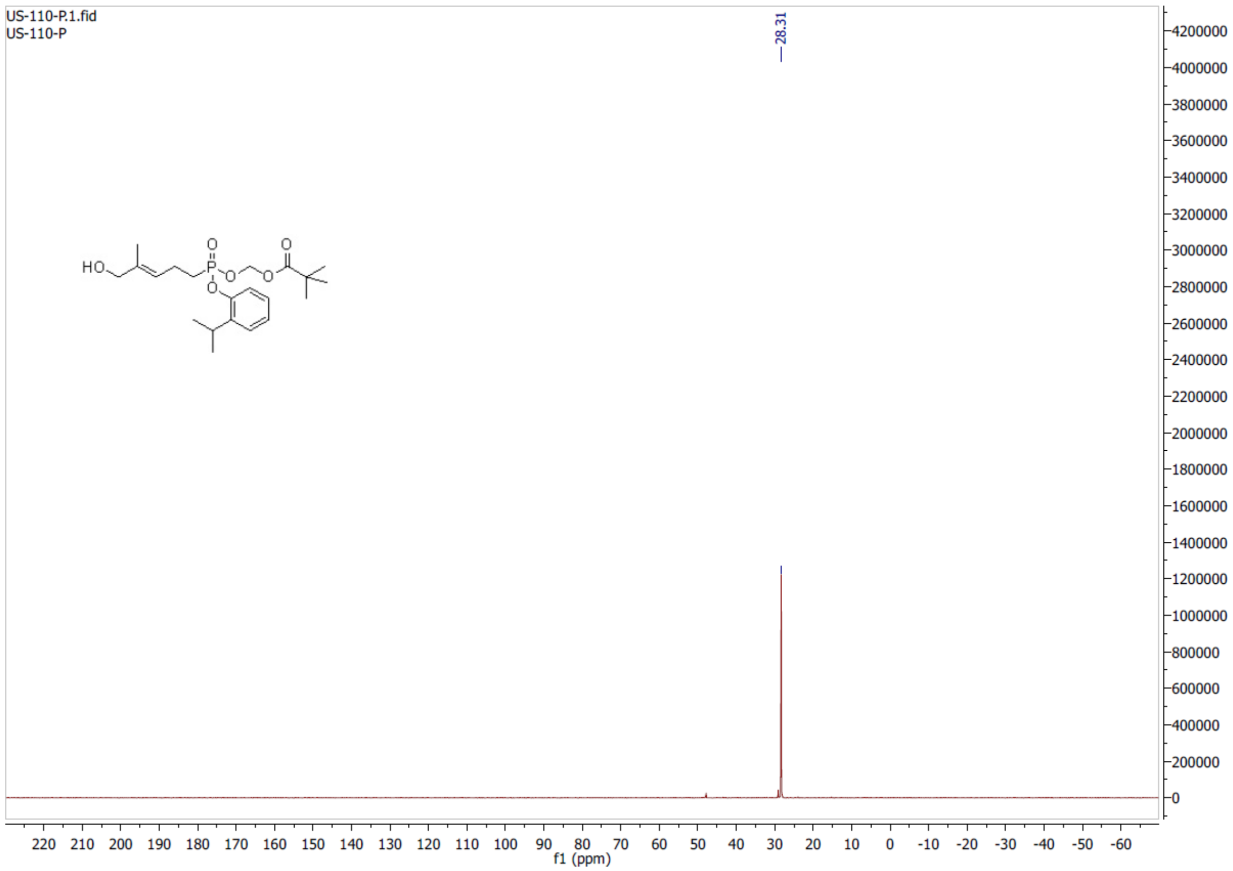

^{31}P NMR Spectrum of Compound **8j** ($CDCl_3$, 162 MHz)

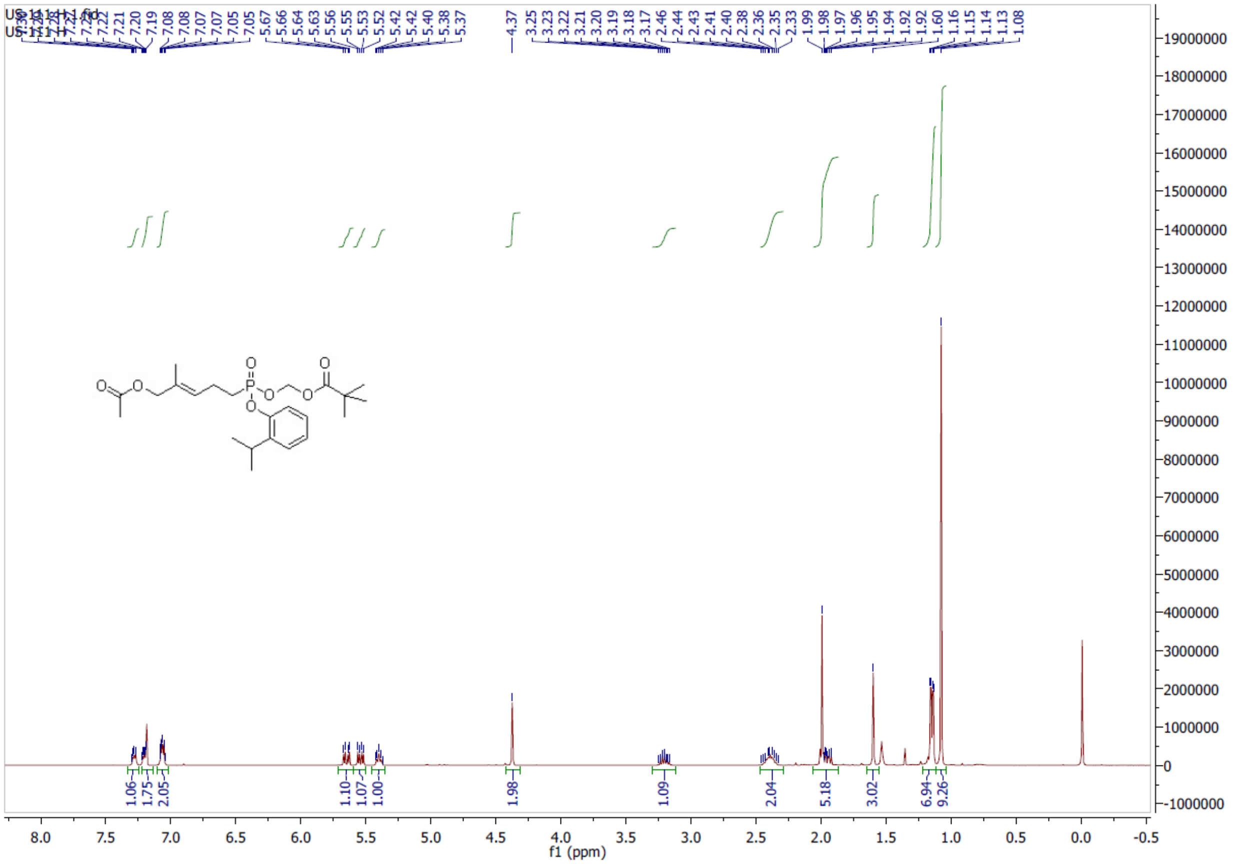

^{1}H NMR Spectrum of Compound **9j** ($CDCl_3$, 400 MHz)

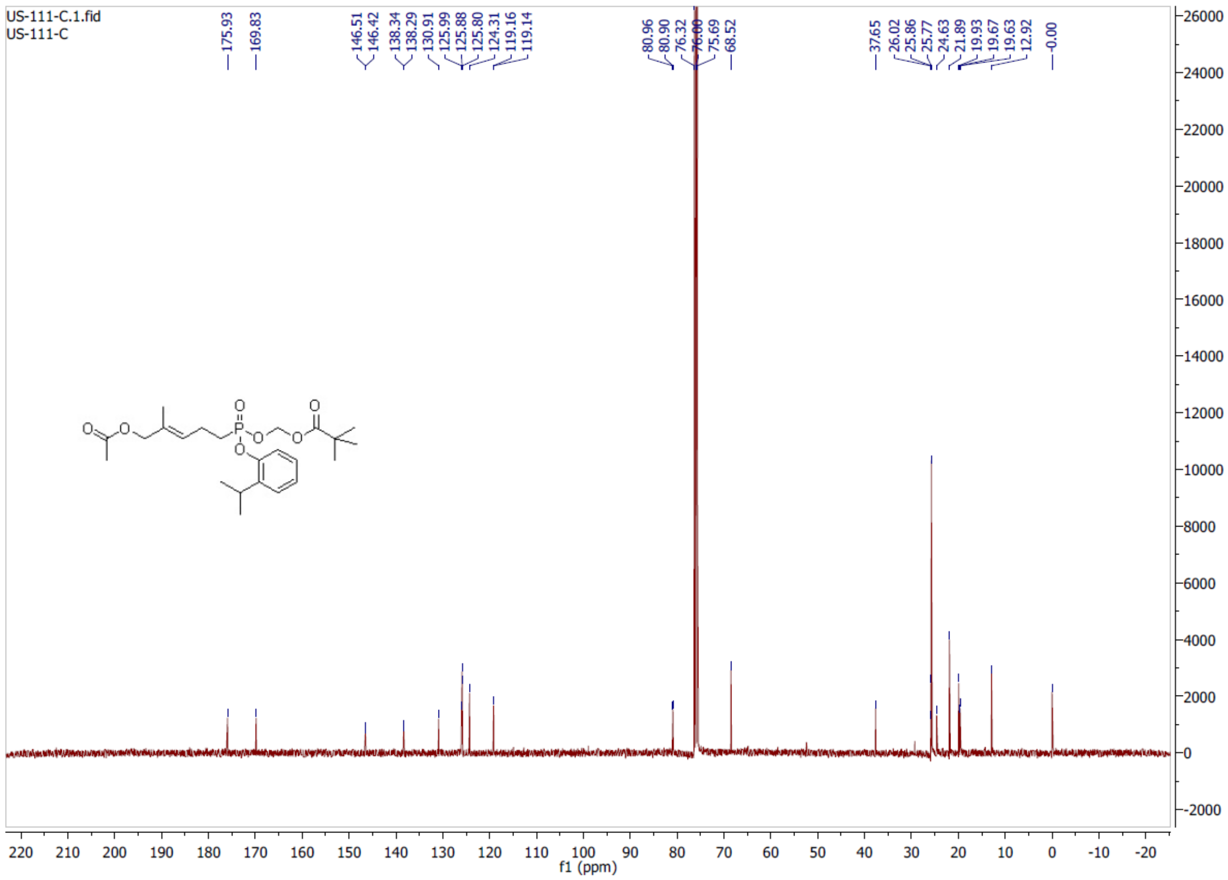

^{13}C NMR Spectrum of Compound **9j** ($CDCl_3$, 101 MHz)

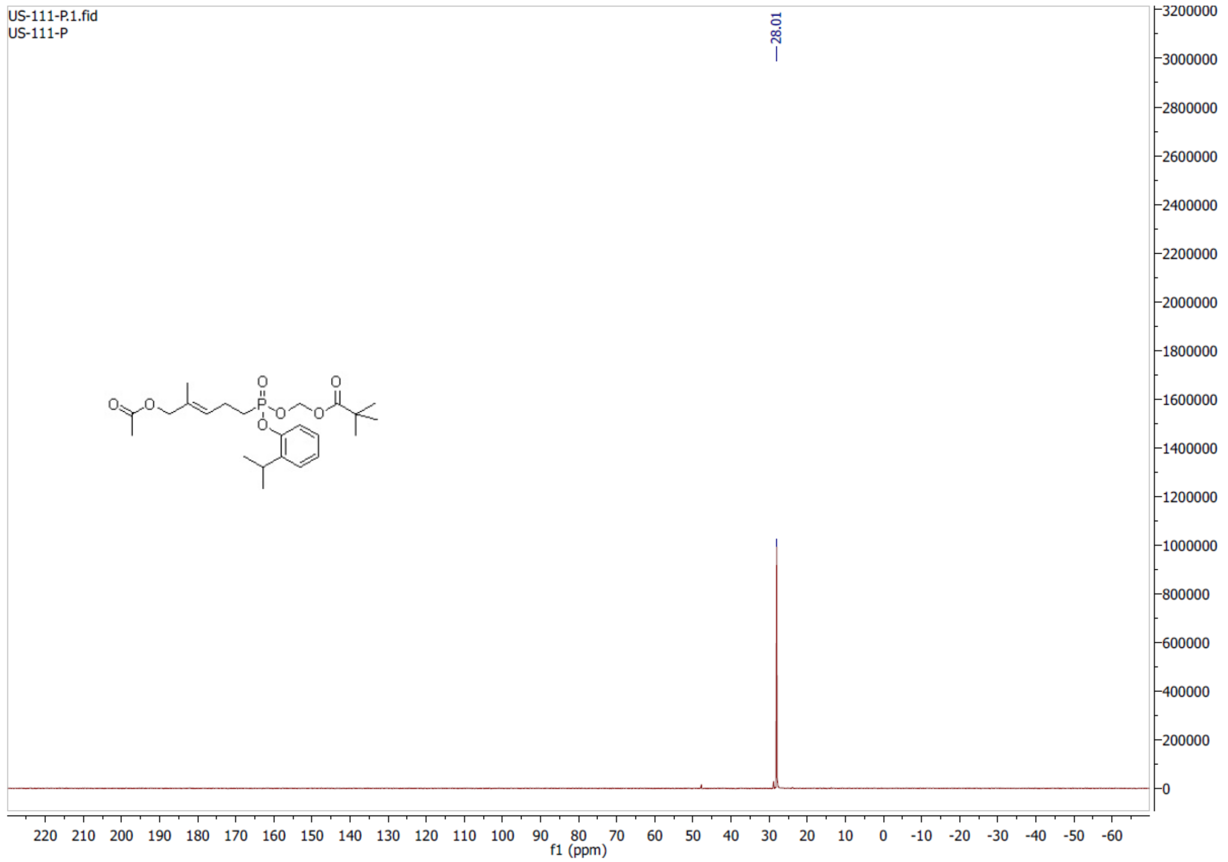

^{31}P NMR Spectrum of Compound **9j** ($CDCl_3$, 162 MHz)

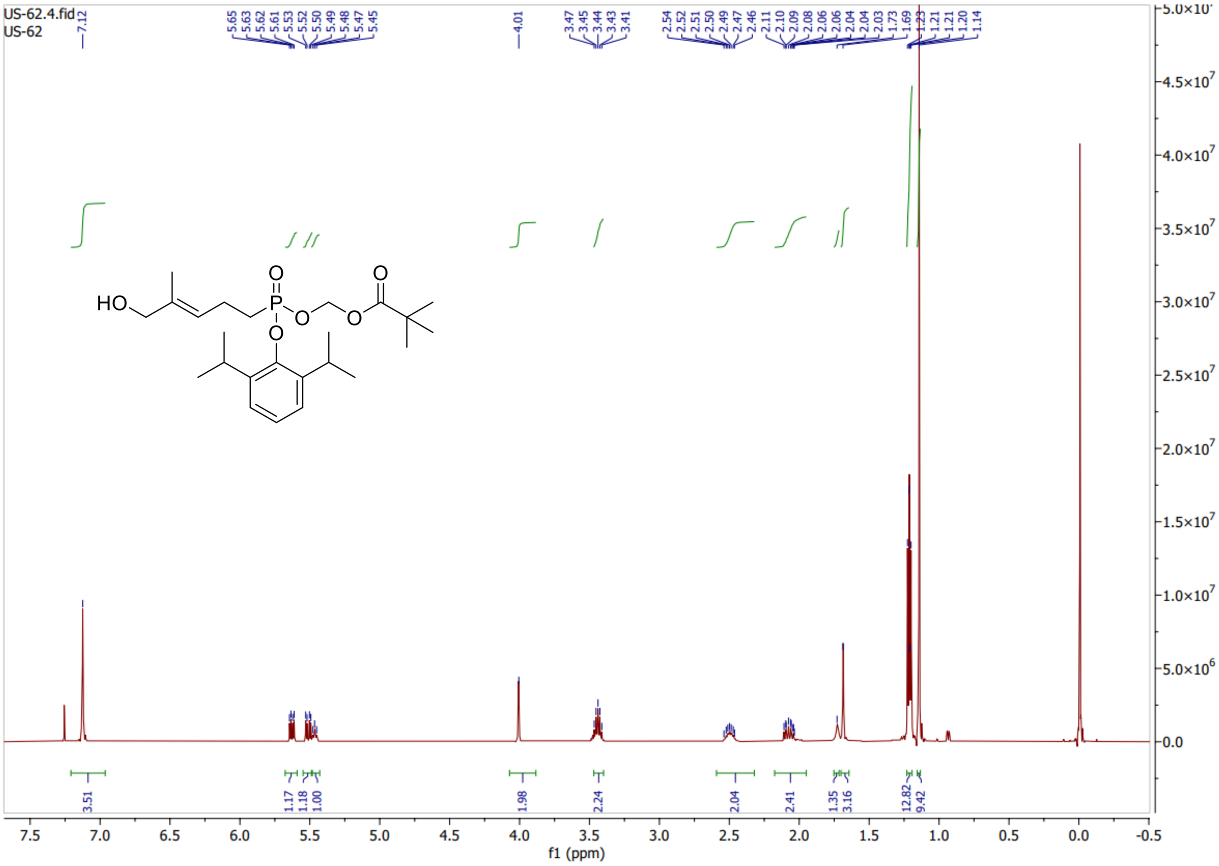

^{1}H NMR Spectrum of Compound **8k** ($CDCl_3$, 500 MHz)

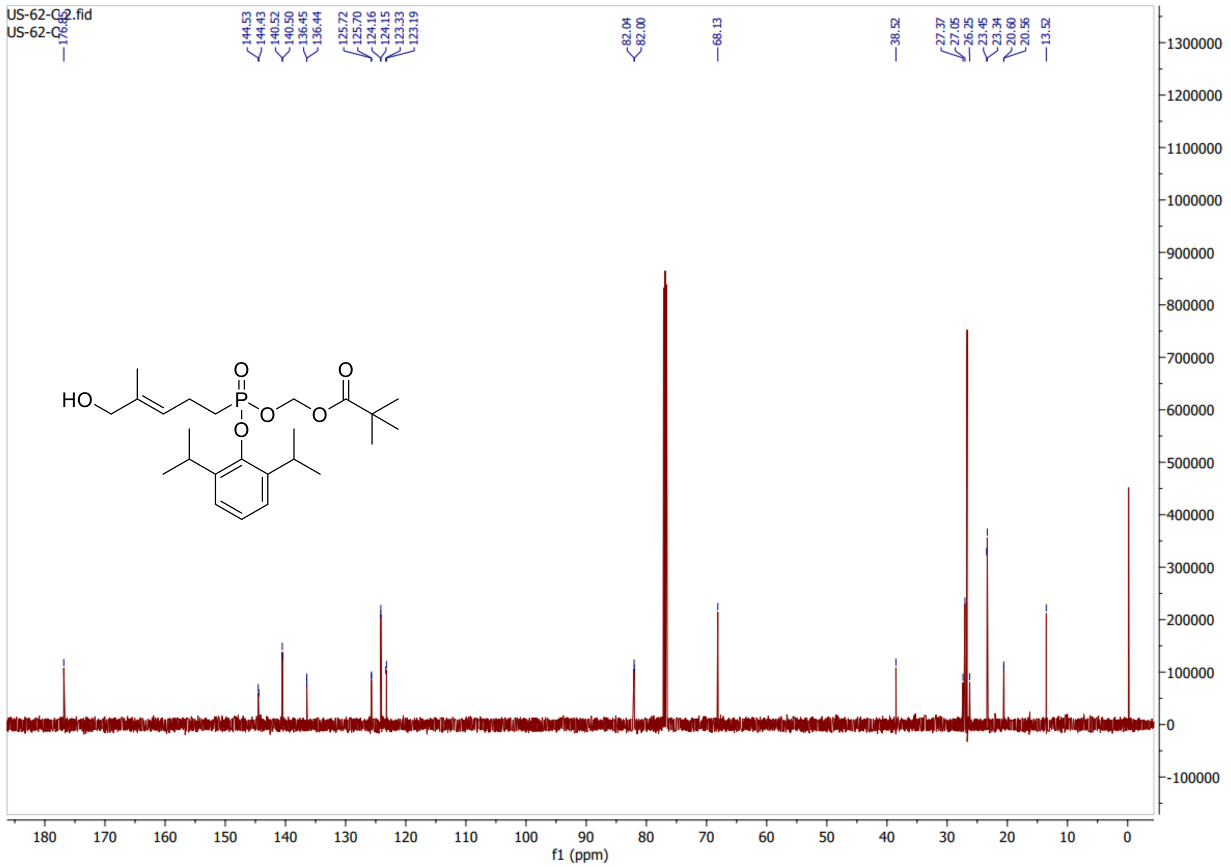

^{13}C NMR Spectrum of Compound **8k** ($CDCl_3$, 126 MHz)

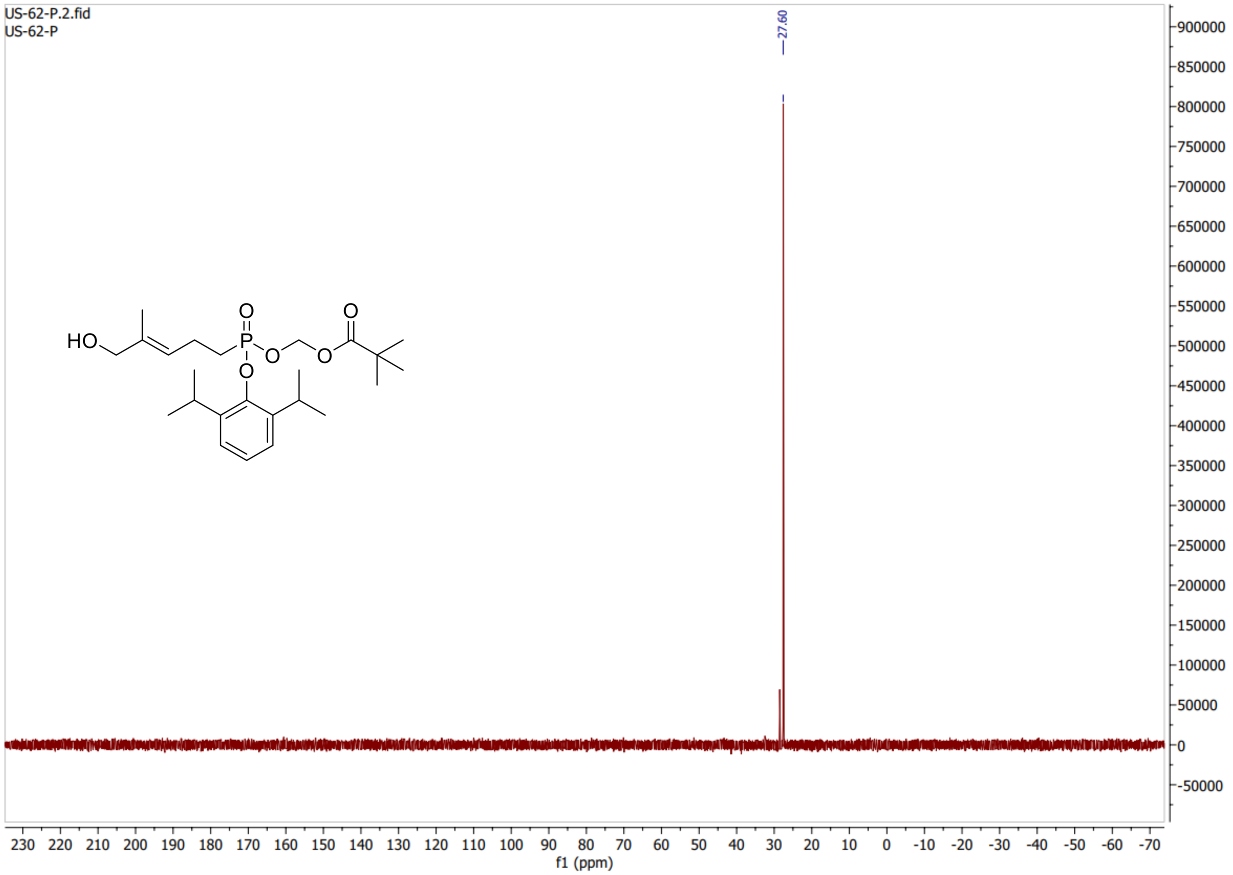

^{31}P NMR Spectrum of Compound **8k** ($CDCl_3$, 203 MHz)

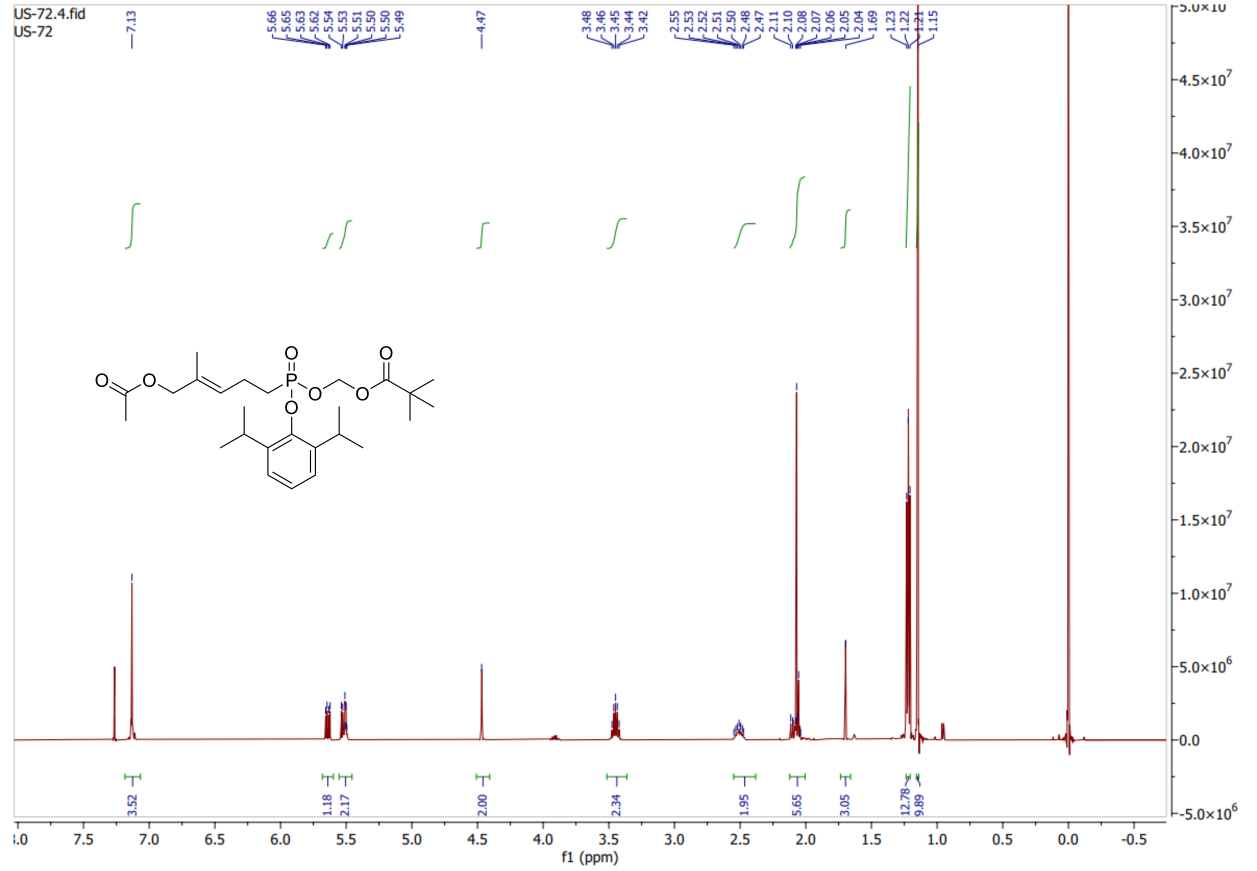

^{1}H NMR Spectrum of Compound **9k** ($CDCl_3$, 500 MHz)

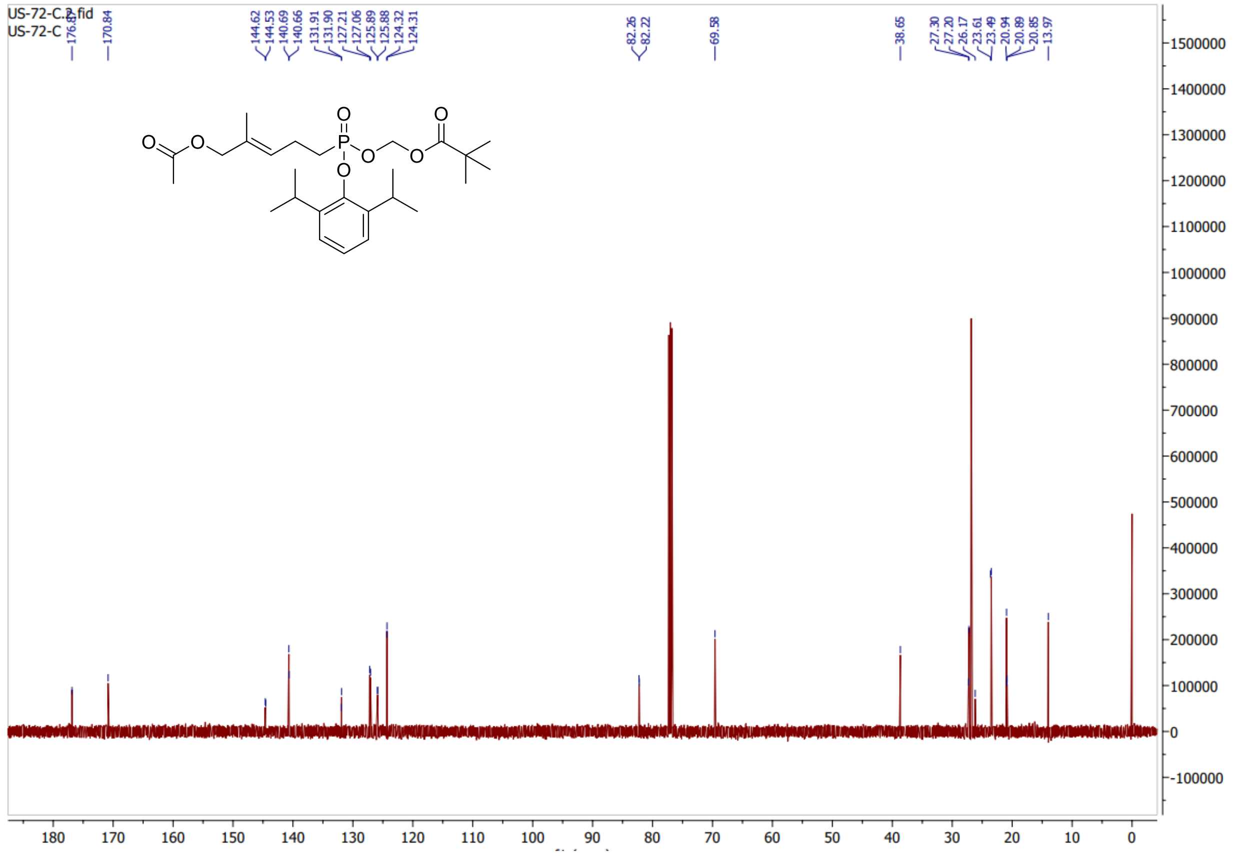

^{13}C NMR Spectrum of Compound **9k** ($CDCl_3$, 126 MHz)

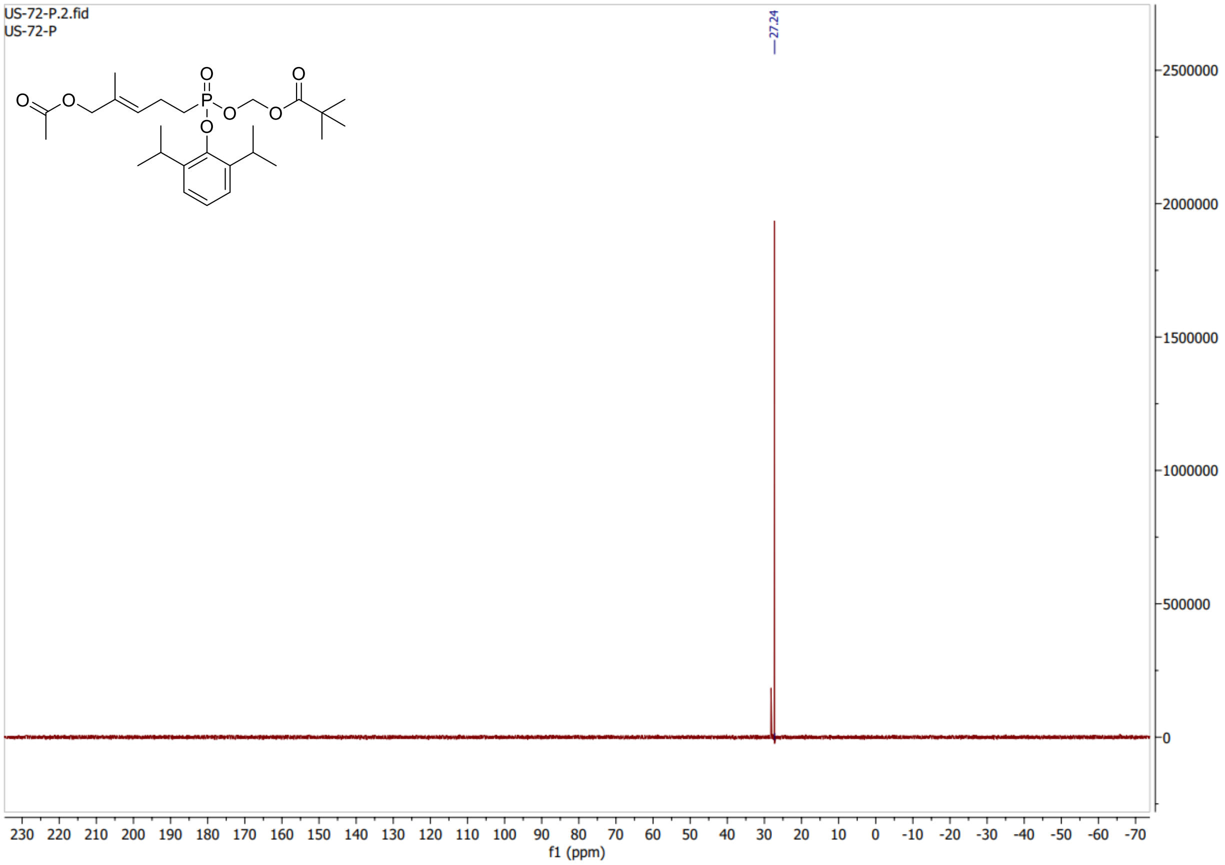

^{31}P NMR Spectrum of Compound **9k** ($CDCl_3$, 203 MHz)

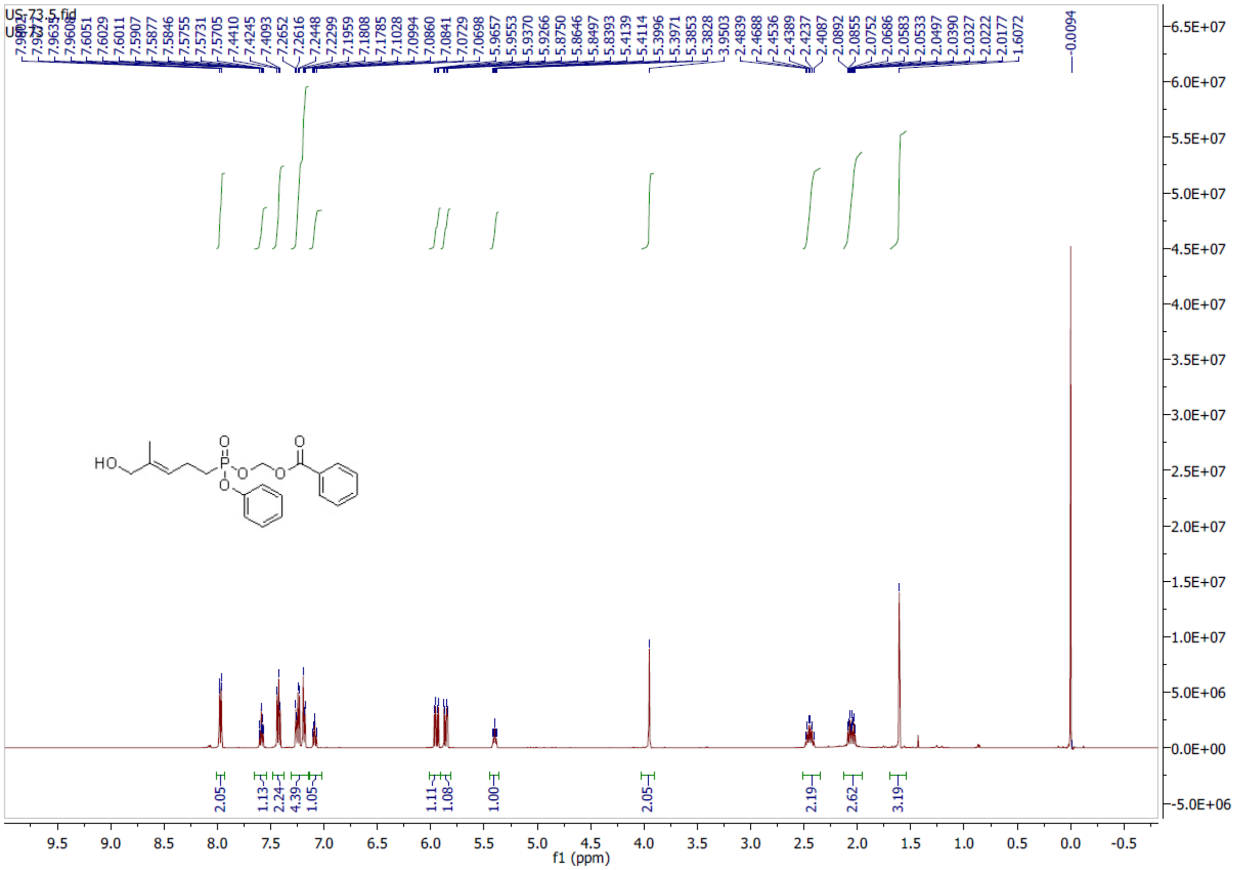

^{1}H NMR Spectrum of Compound **11x** ($CDCl_3$, 500 MHz)

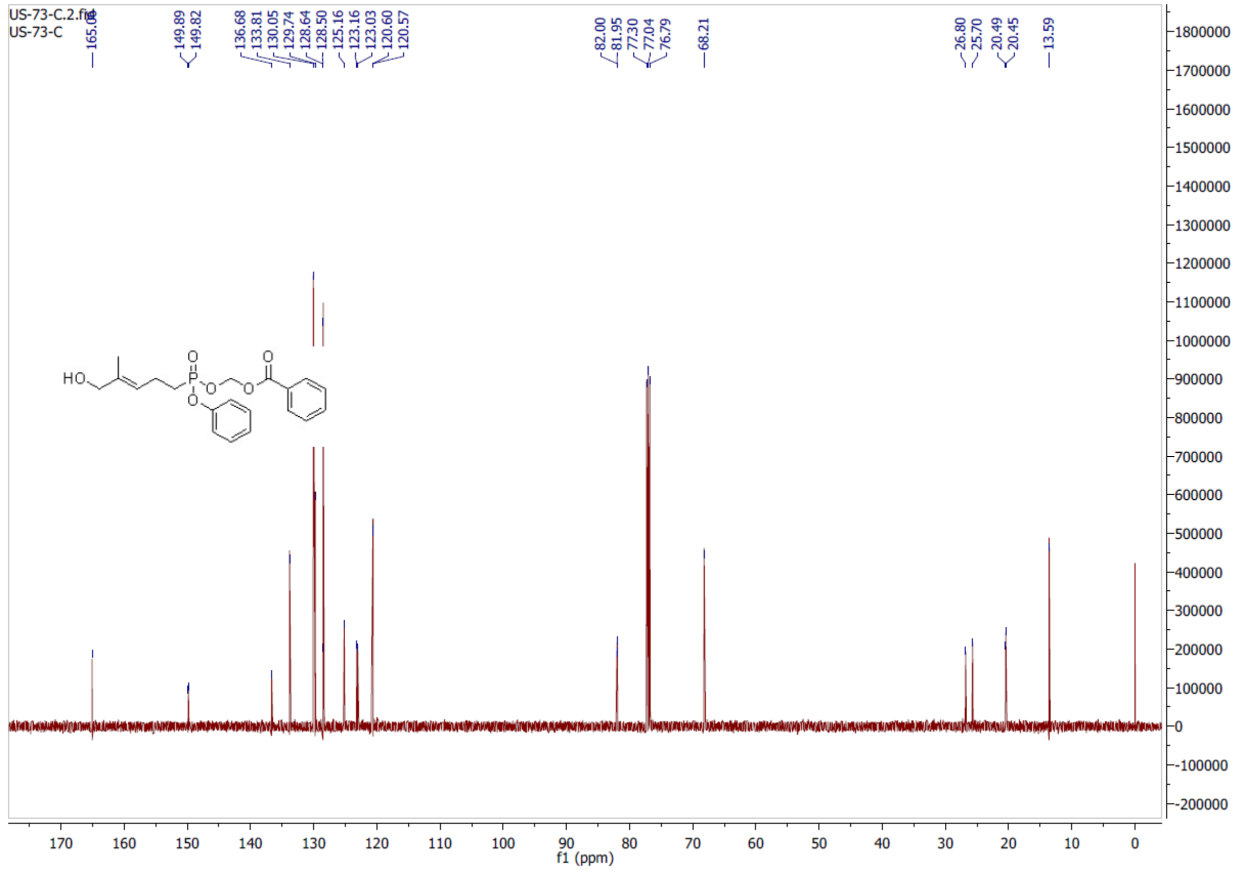

^{13}C NMR Spectrum of Compound **11x** ($CDCl_3$, 126 MHz)

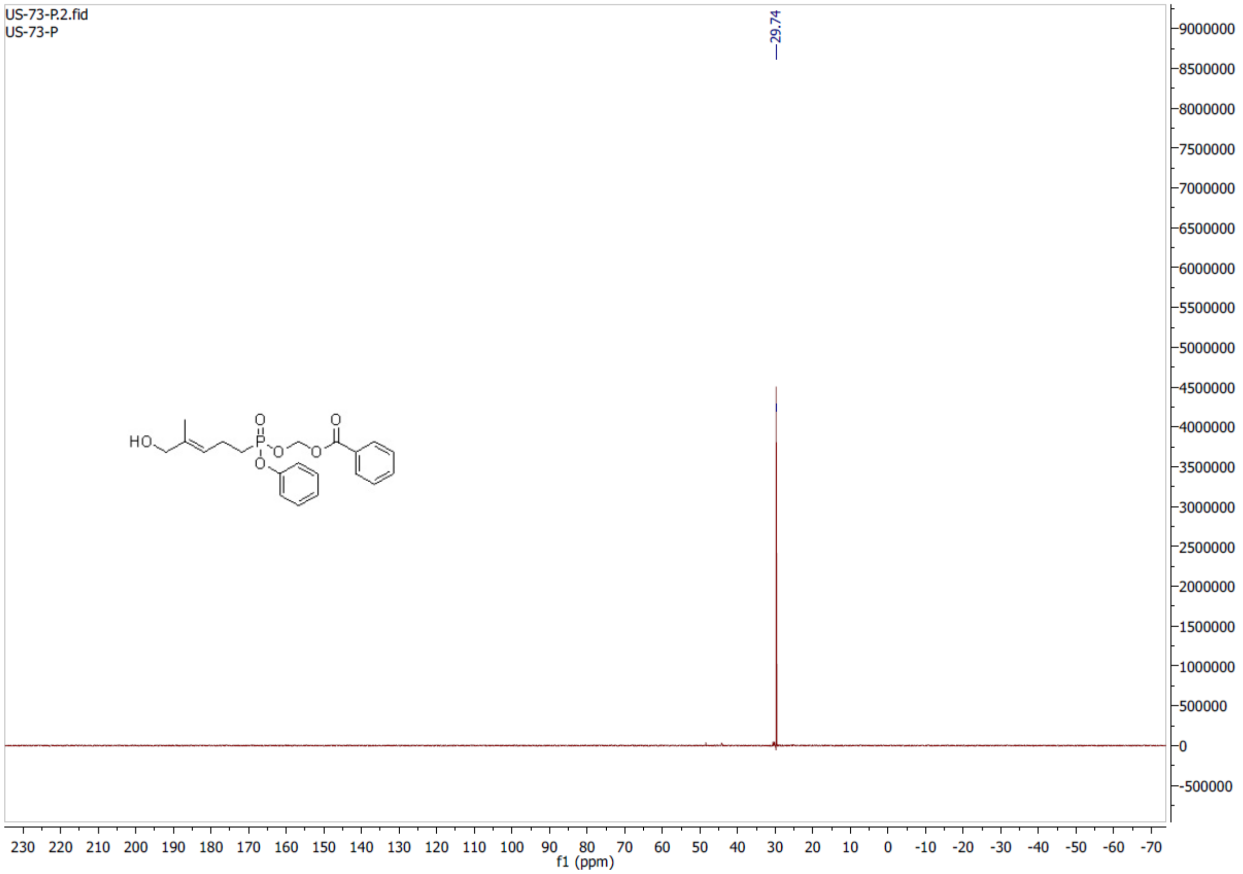

^{31}P NMR Spectrum of Compound **11x** ($CDCl_3$, 203 MHz)

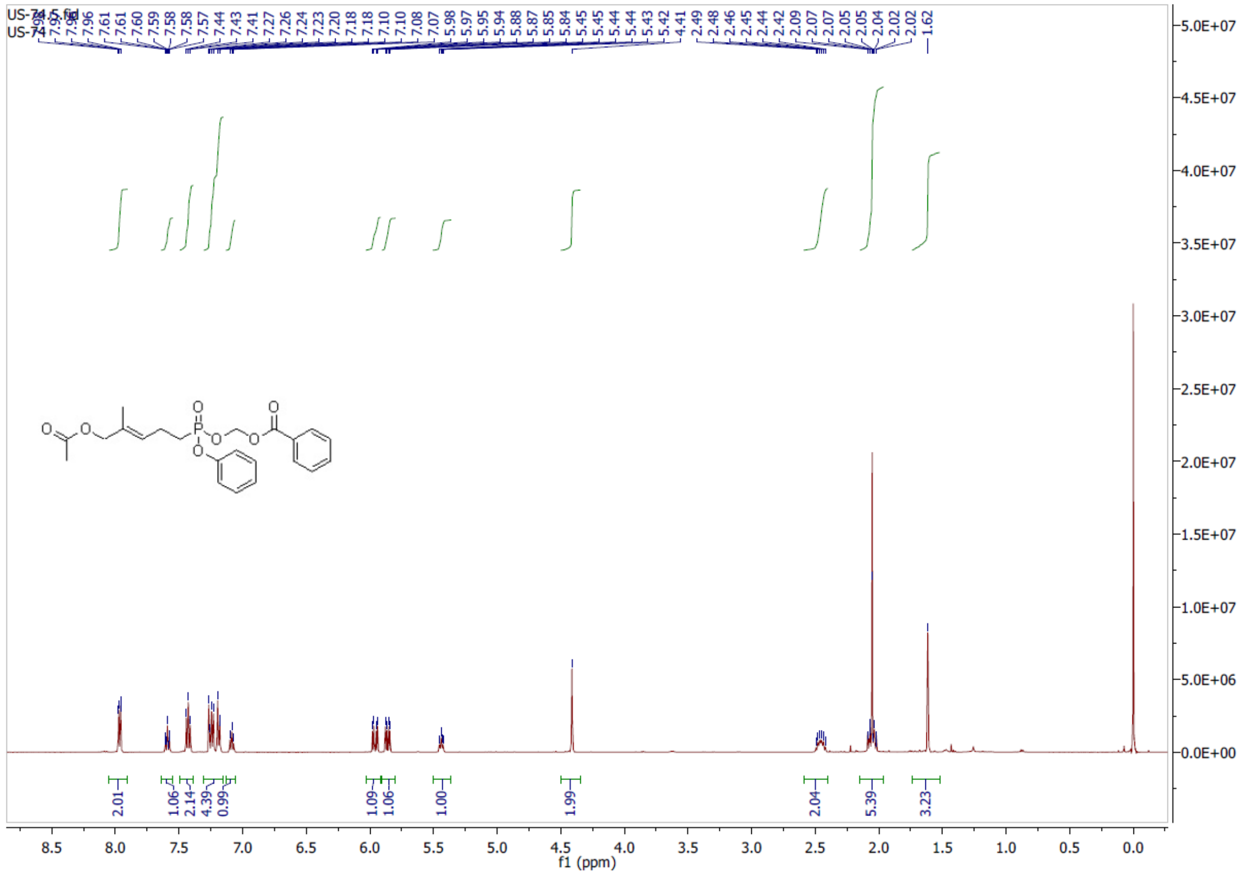

^{1}H NMR Spectrum of Compound **12x** ($CDCl_3$, 500 MHz)

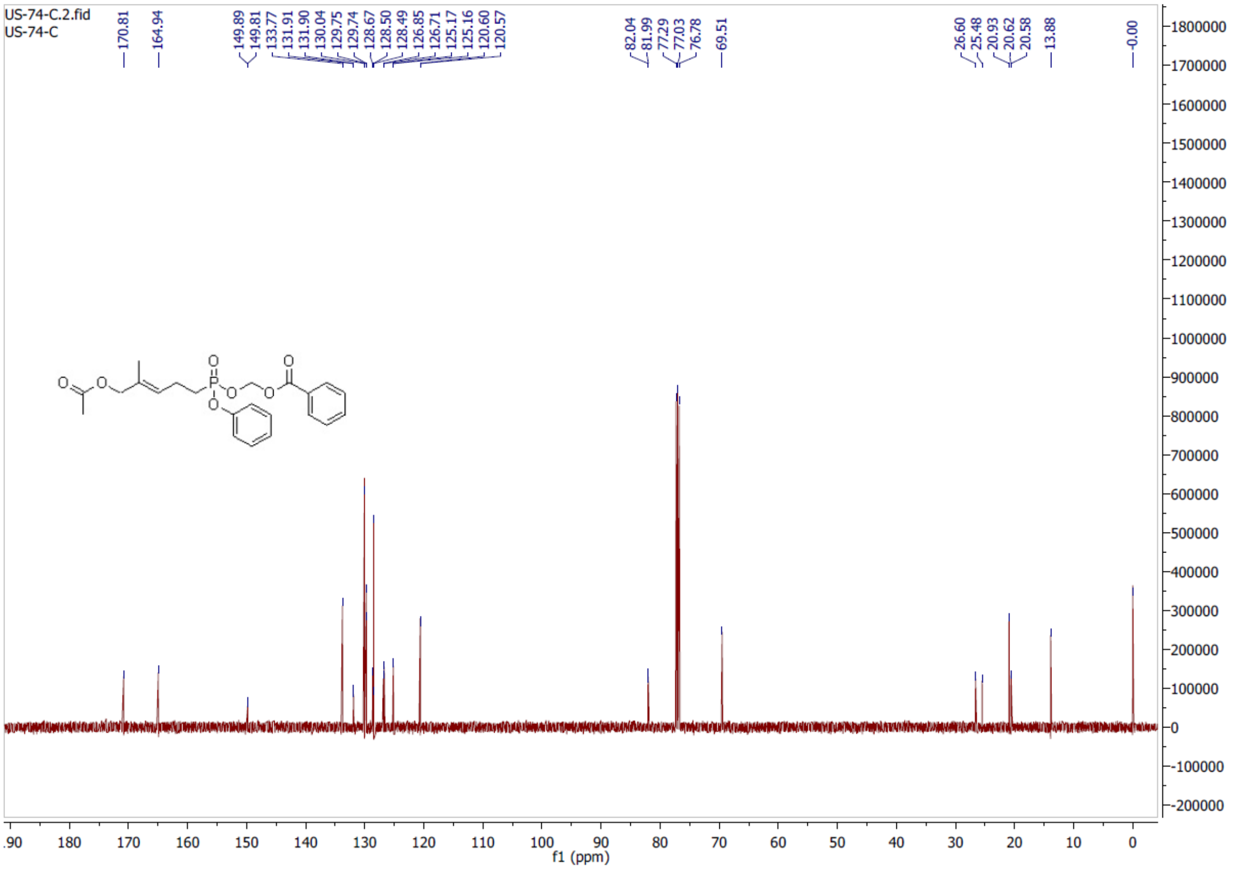

^{13}C NMR Spectrum of Compound **12x** ($CDCl_3$, 126 MHz)

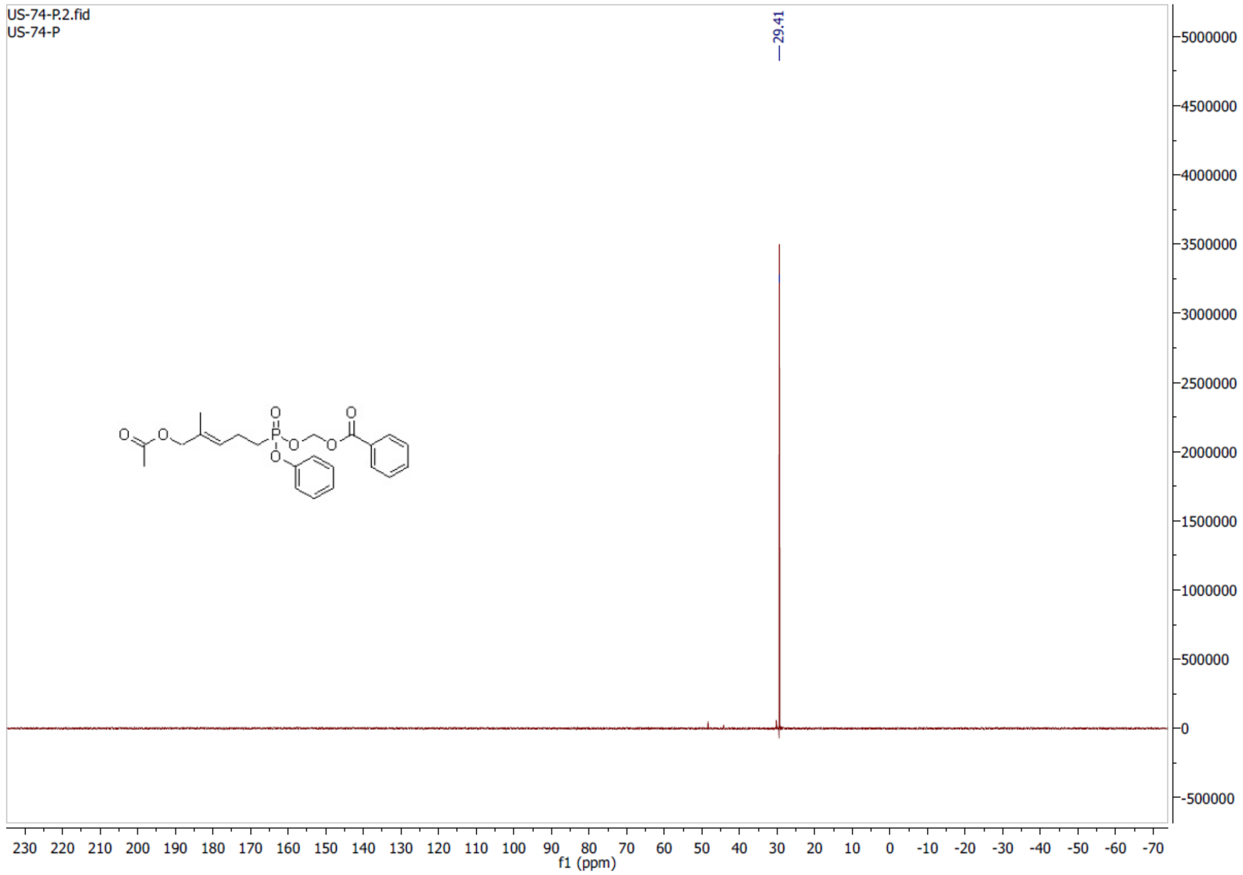

^{31}P NMR Spectrum of Compound **12x** ($CDCl_3$, 203 MHz)

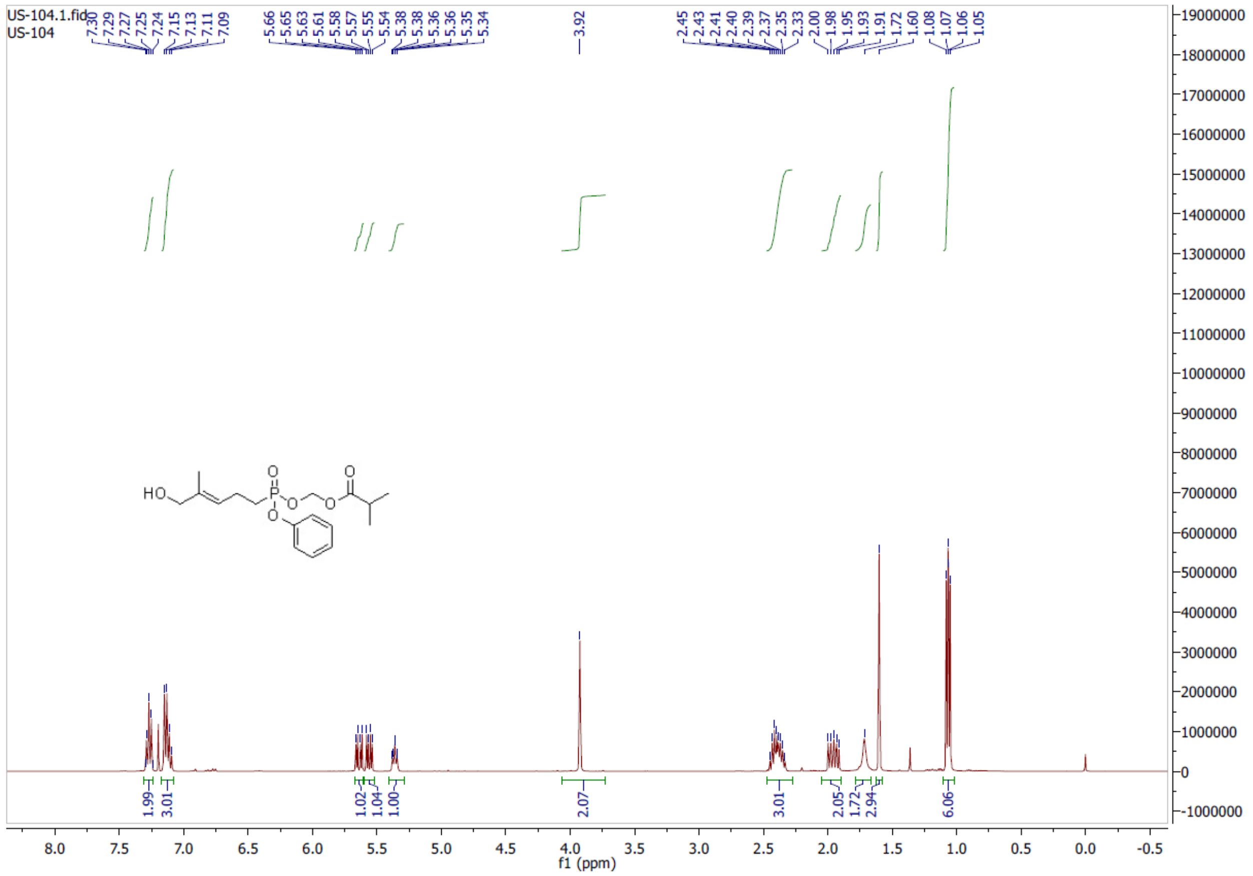

^{1}H NMR Spectrum of Compound **11y** ($CDCl_3$, 400 MHz)

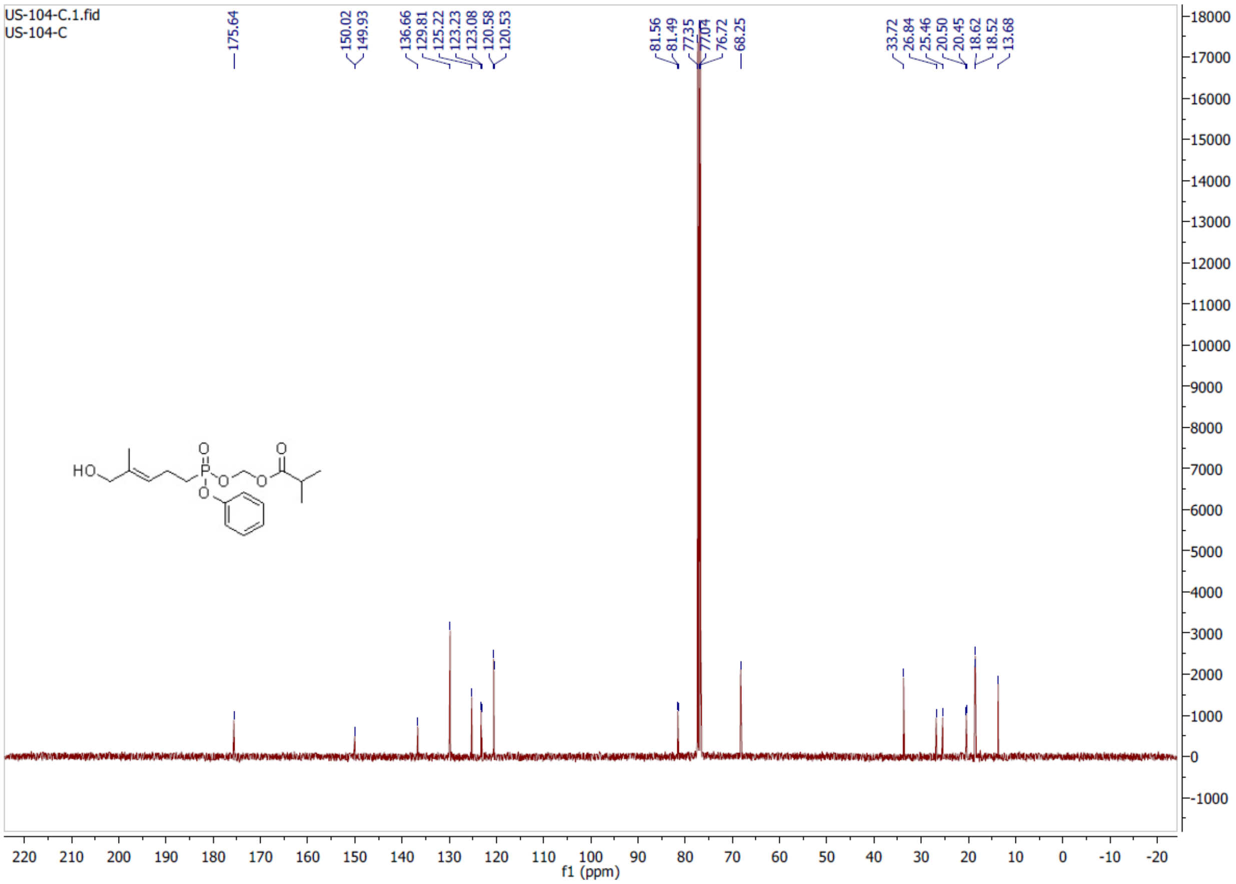

^{13}C NMR Spectrum of Compound **11y** ($CDCl_3$, 101 MHz)

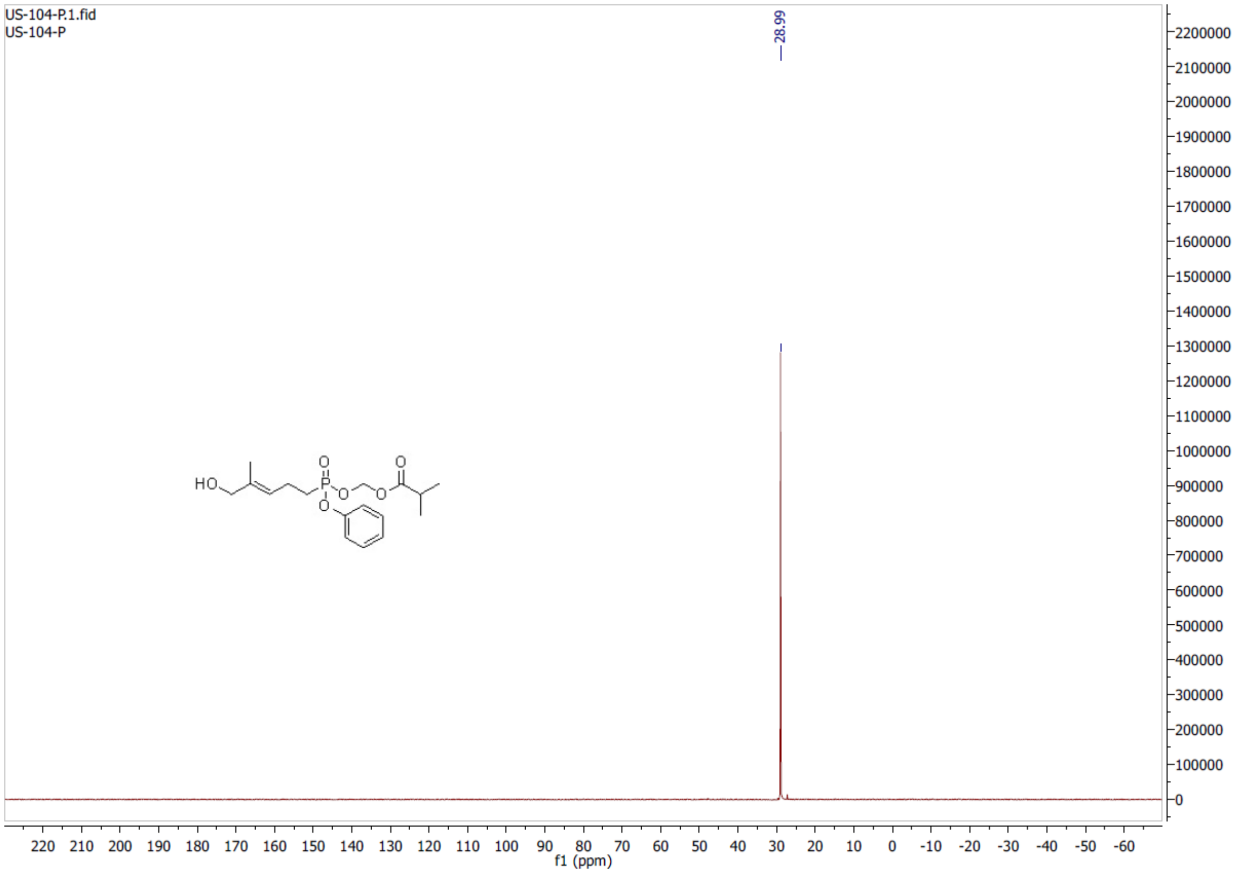

^{31}P NMR Spectrum of Compound **11y** ($CDCl_3$, 162 MHz)

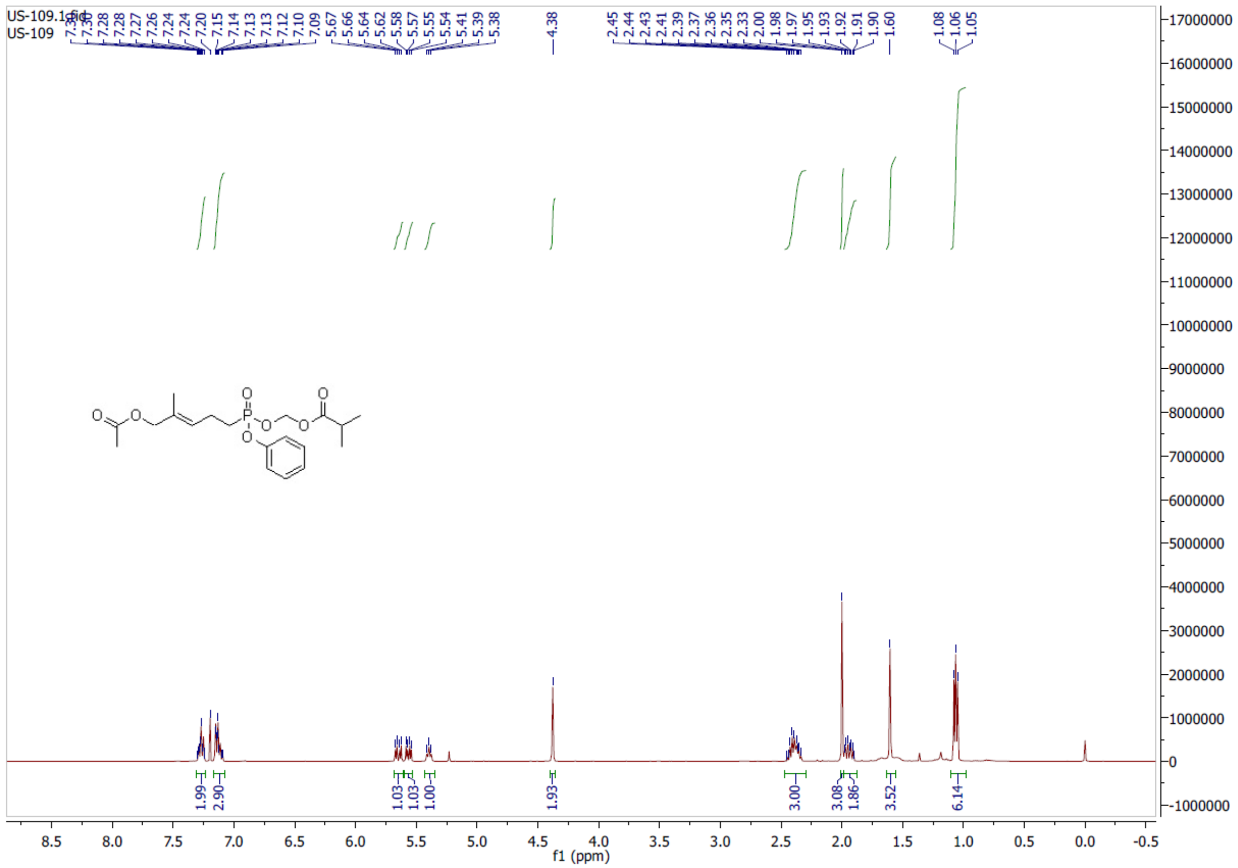

^{1}H NMR Spectrum of Compound **12y** ($CDCl_3$, 400 MHz)

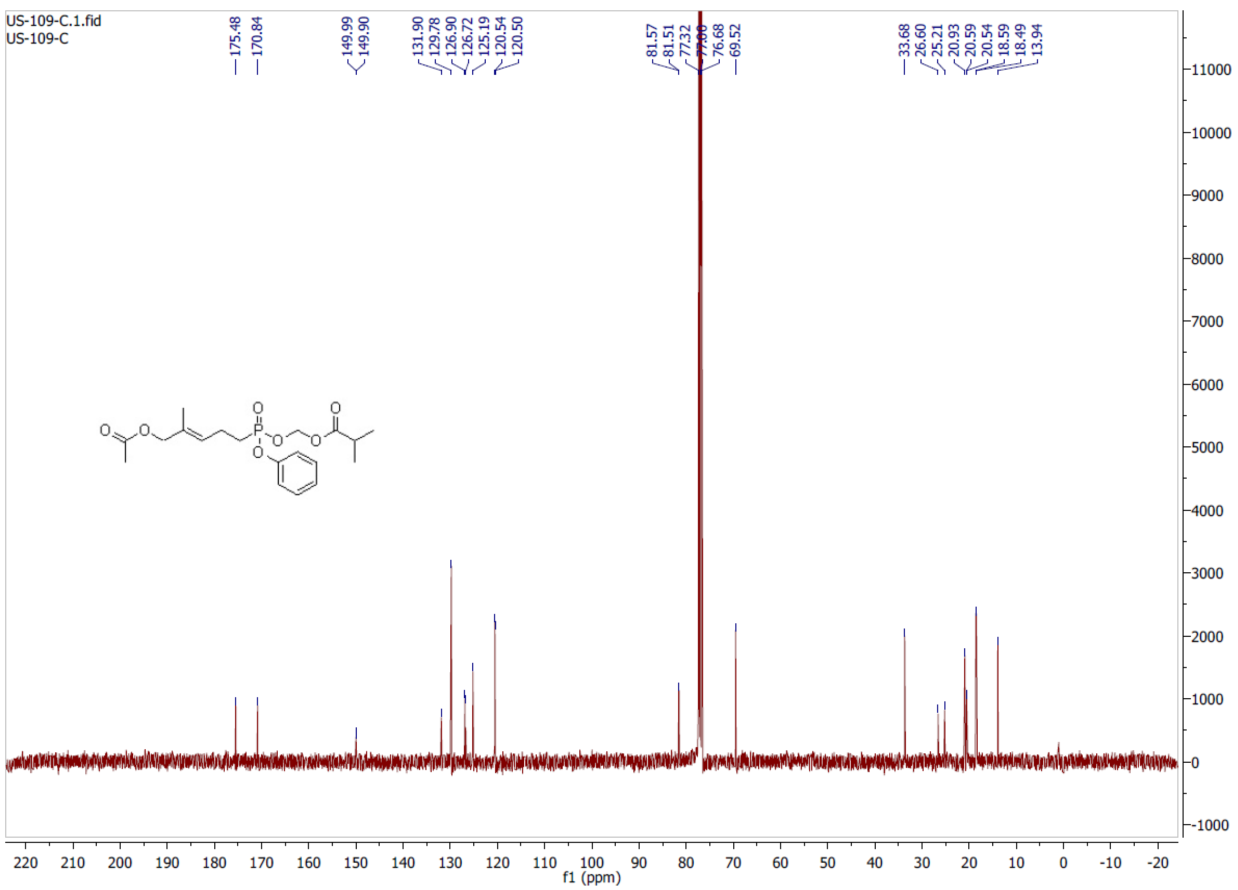

^{13}C NMR Spectrum of Compound **12y** ($CDCl_3$, 101 MHz)

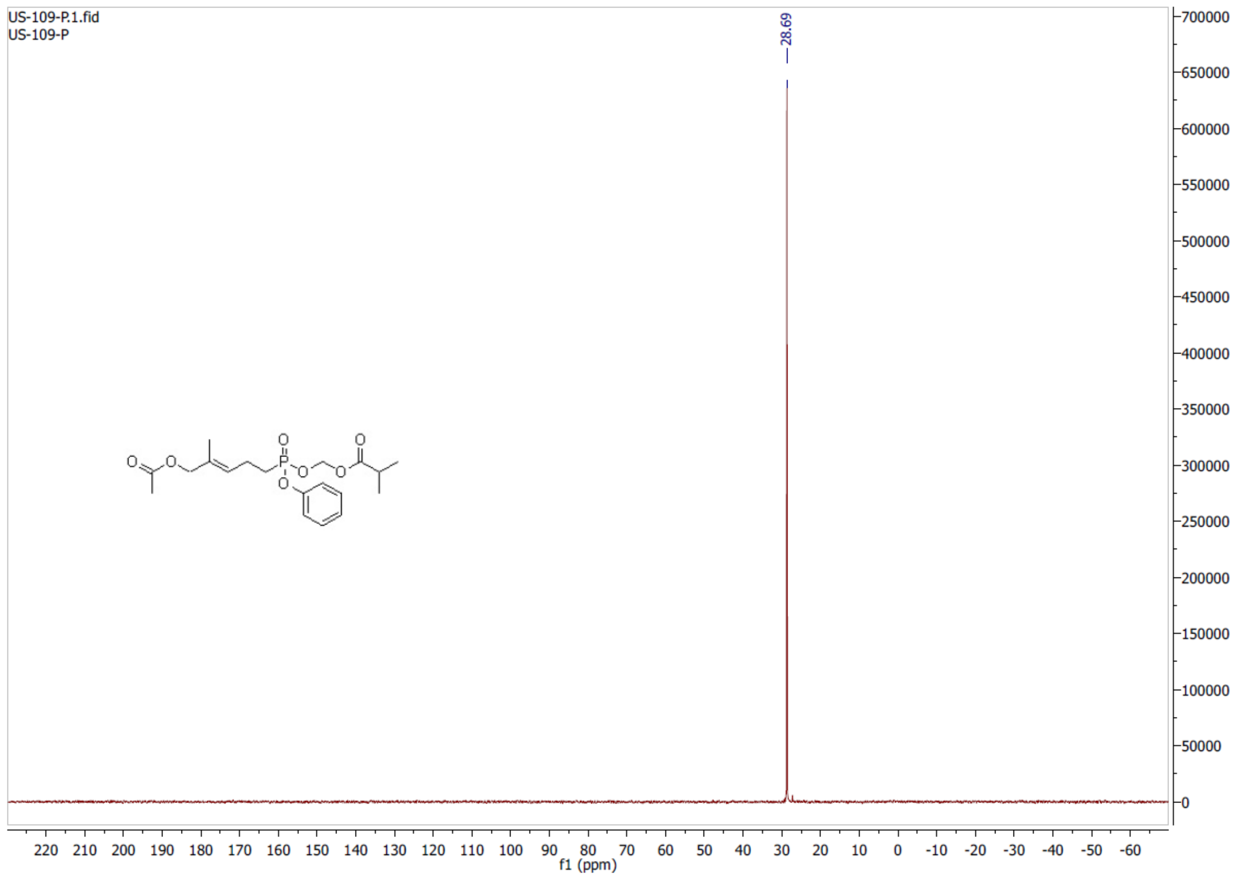

^{31}P NMR Spectrum of Compound **12y** ($CDCl_3$, 162 MHz)

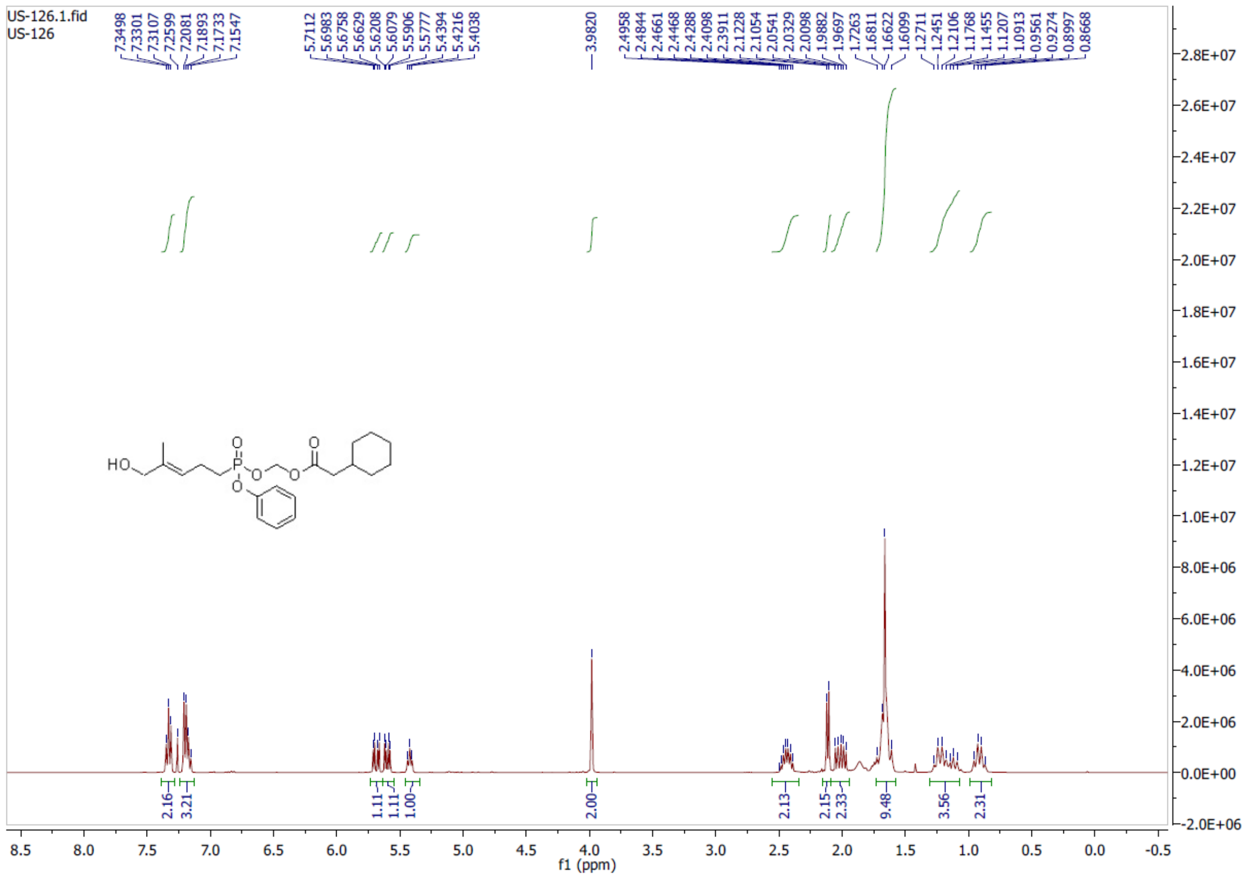

^{1}H NMR Spectrum of Compound **11z** ($CDCl_3$, 400 MHz)

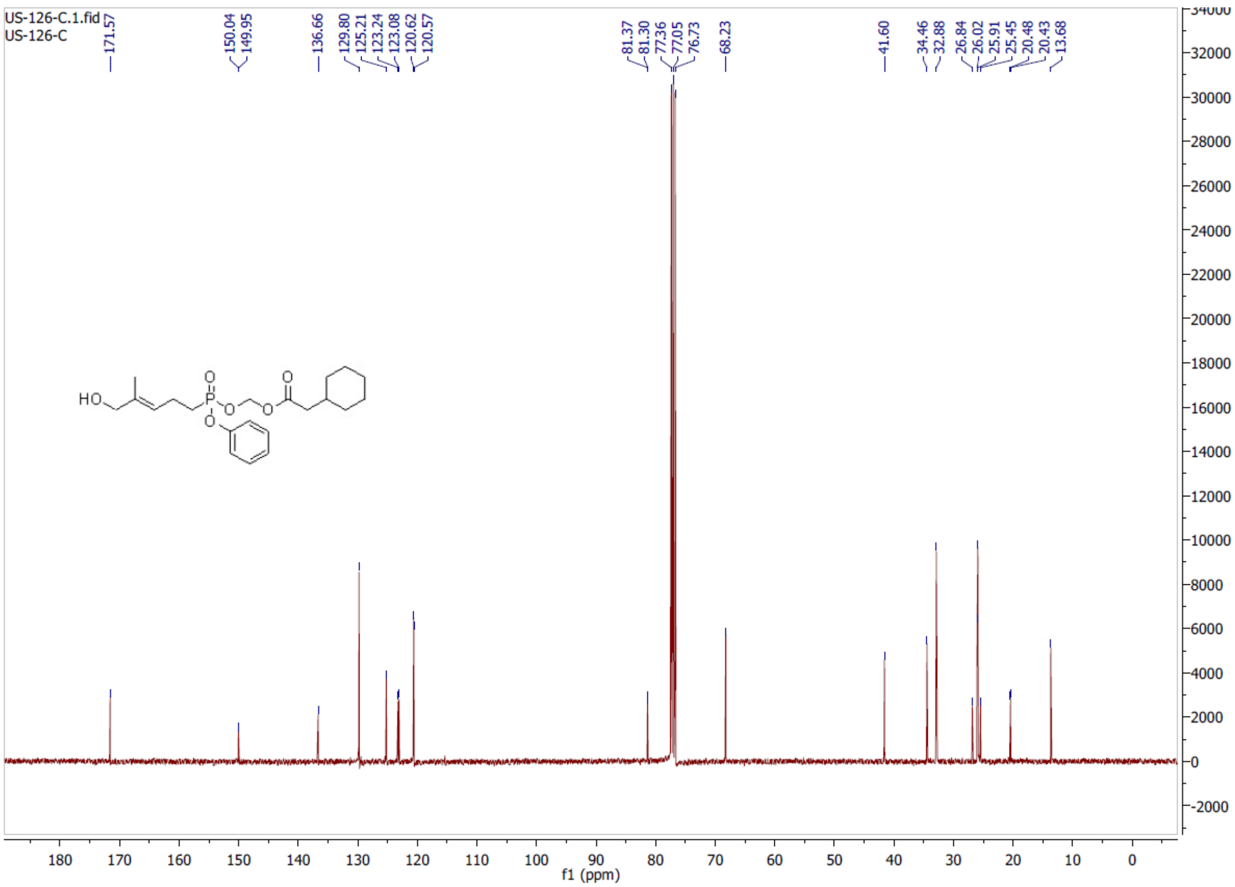

^{13}C NMR Spectrum of Compound **11z** ($CDCl_3$, 101 MHz)

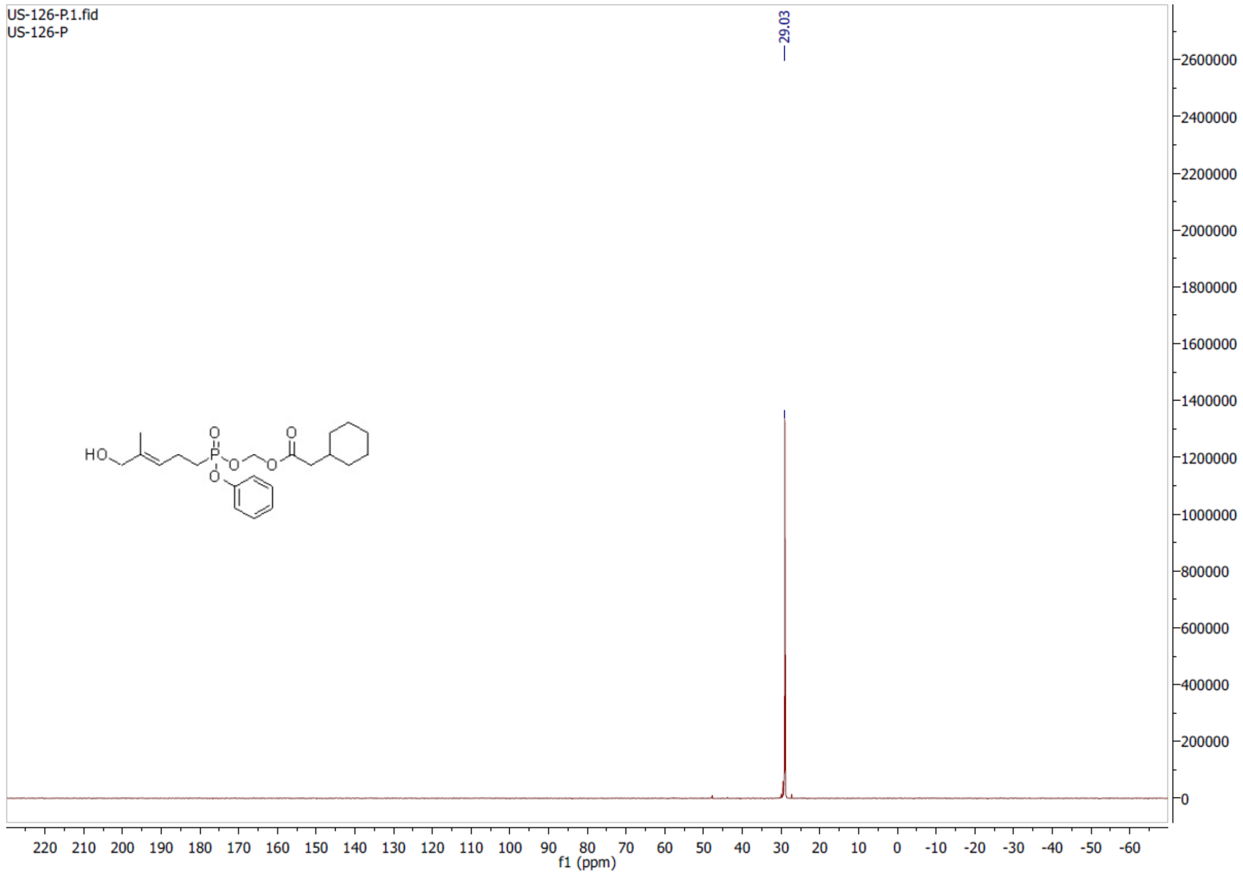

^{31}P NMR Spectrum of Compound **11z** ($CDCl_3$, 162 MHz)

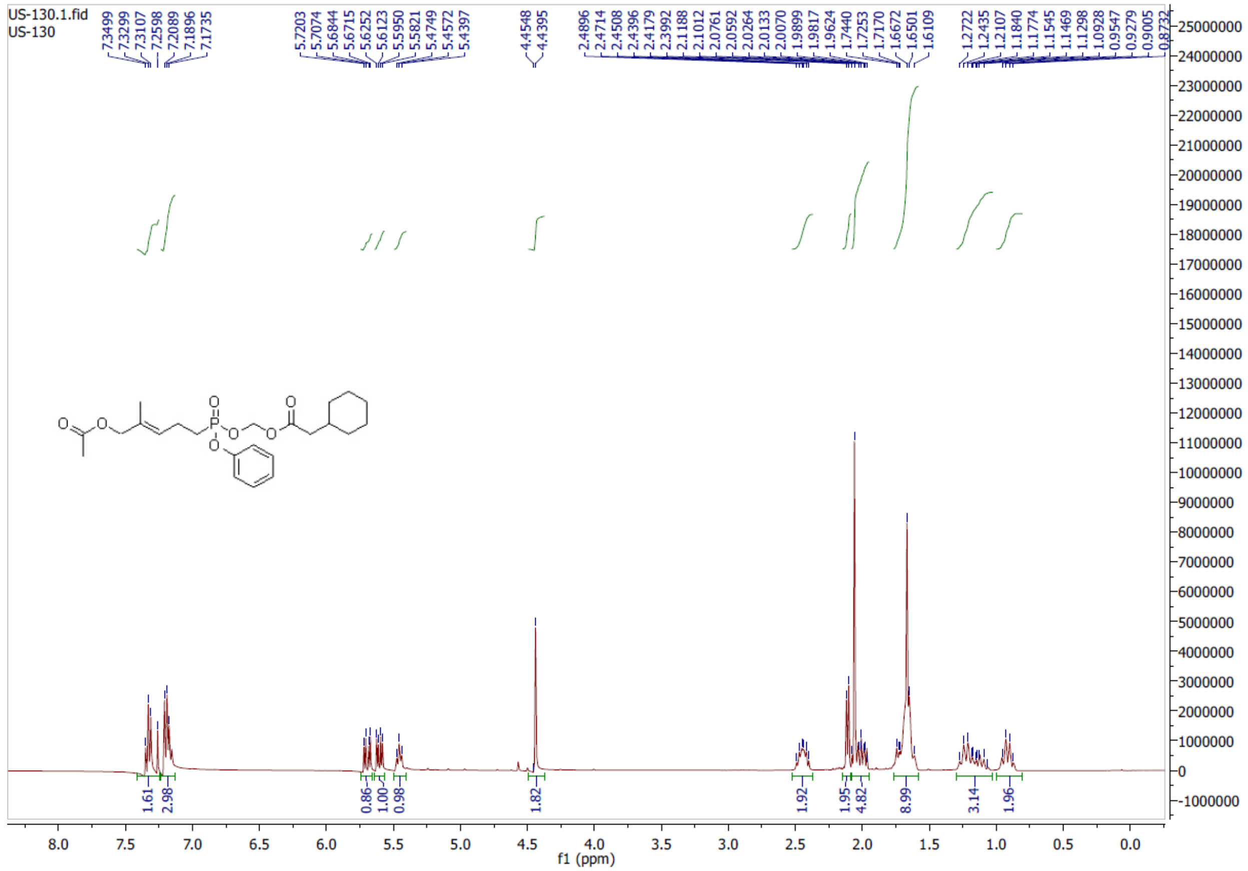

^{1}H NMR Spectrum of Compound **12z** ($CDCl_3$, 400 MHz)

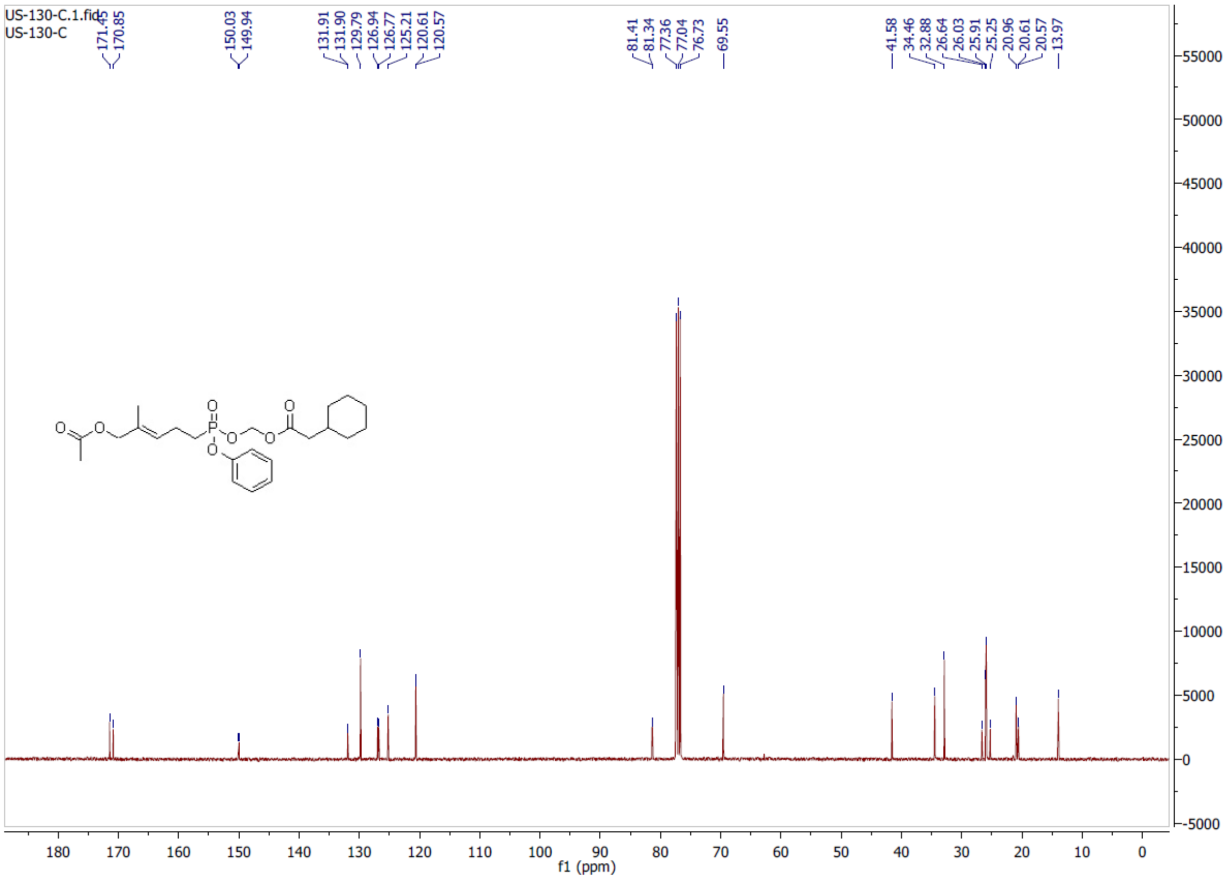

^{13}C NMR Spectrum of Compound **12z** ($CDCl_3$, 101 MHz)

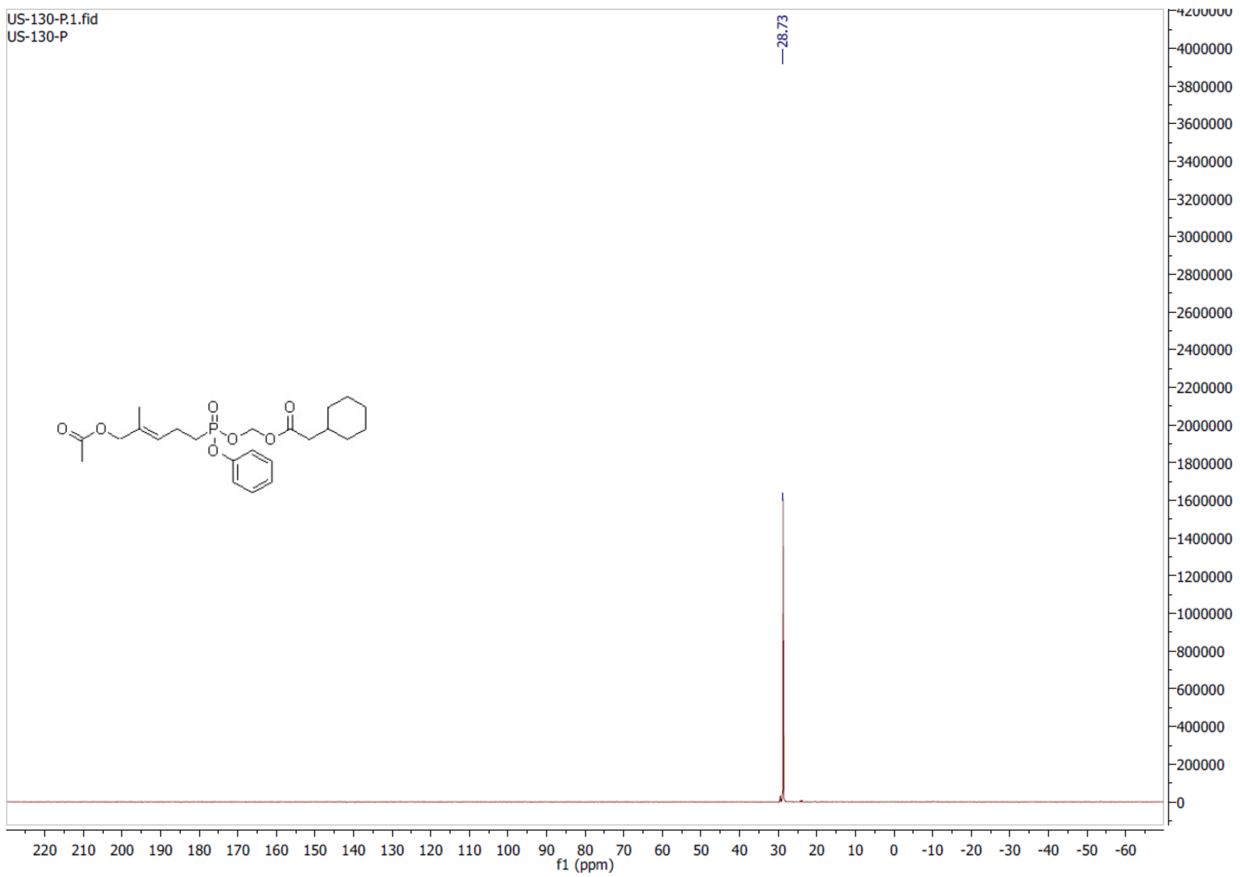

^{31}P NMR Spectrum of Compound **12z** ($CDCl_3$, 162 MHz)

```
Data File C:\Users\P...ents\ChemStation\1\Data\Wiemer\2022-10-3112-38-231US-OAcetyl-meoh.D
Sample Name: US-OAcetyl-meoh

=====================================================================
Acq. Operator   : SYSTEM
                                                  Location :     -
Injection Date  : 10/31/2022 12:38:23 PM               Inj :   1
Acq. Method     : US M-1.M
Analysis Method : C:\Users\Public\Documents\ChemStation\1\Methods\US M-1.M
Last changed    : 10/28/2022 9:15:20 AM by SYSTEM
Sample Info     : US-OAcetyl-meoh
```

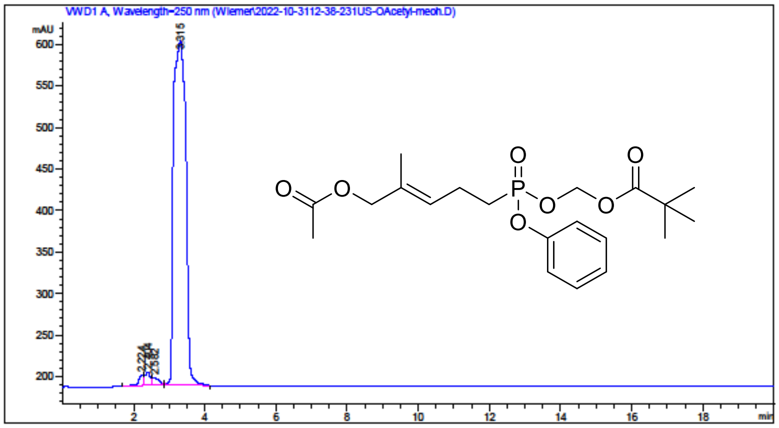


```
=====================================================================
                         Area Percent Report
=====================================================================

Sorted By             :      Signal
Multiplier            :      1.0000
Dilution              :      1.0000
Use Multiplier & Dilution Factor with ISTDs


Signal 1: VWD1 A, Wavelength=250 nm
```

Peak #	RetTime [min]	Type	Width [min]	Area [mAU*s]	Height [mAU]	Area %
1	2.224	BV	0.1461	124.87245	12.44156	1.2183
2	2.404	VV	0.1385	158.13829	15.83800	1.5428
3	2.582	VB	0.1786	105.27887	8.37838	1.0271
4	3.315	BV R	0.3150	9861.75098	413.83557	96.2118

Totals : 1.02500e4 450.49351

```
=====================================================================
```

Shared 1220 10/31/2022 3:01:40 PM SYSTEM Page 1 of 2

HPLC Chromatogram of Compound **9a**

Data File C:\Users\Public\Documents\ChemStation\1\Data\Wiemer\2022-11-0913-30-011US-29.D
Sample Name: US-29

===

Acq. Operator : SYSTEM
Location : -
Injection Date : 11/9/2022 1:30:01 PM
Inj : 1
Acq. Method : US M-1.M
Analysis Method : C:\Users\Public\Documents\ChemStation\1\Methods\US M-1.M
Last changed : 11/9/2022 1:45:50 PM by SYSTEM
(modified after loading)
Sample Info : US-29

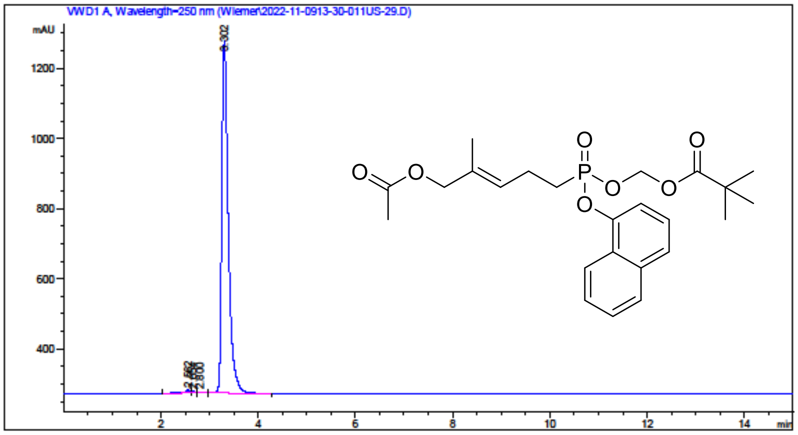

===

Area Percent Report

===

Sorted By : Signal
Multiplier : 1.0000
Dilution : 1.0000
Use Multiplier & Dilution Factor with ISTDs

Signal 1: VWD1 A, Wavelength=250 nm

Peak #	RetTime [min]	Type	Width [min]	Area [mAU*s]	Height [mAU]	Area %
1	2.562	BV	0.1454	88.49483	7.85472	0.9398
2	2.654	VV	0.0732	21.19735	4.10979	0.2251
3	2.800	VB	0.0941	13.71026	2.12983	0.1456
4	3.302	BB	0.1403	9292.67676	1001.32300	98.6894

Totals : 9416.07920 1015.41733

HPLC Chromatogram of Compound **9b**

Data File C:\Users\Public\Documents\ChemStation\1\Data\Wiemer\2022-12-0910-39-541US-40.D
Sample Name: US-40

===

Acq. Operator : SYSTEM
Sample Operator : SYSTEM
Acq. Instrument : Shared 1220 Location : -
Injection Date : 12/9/2022 10:39:54 AM Inj : 1
Inj Volume : No inj
Method : C:\Users\Public\Documents\ChemStation\1\Methods\US M-1.M
Last changed : 12/9/2022 9:27:23 AM by SYSTEM
(modified after loading)
Sample Info : US-40

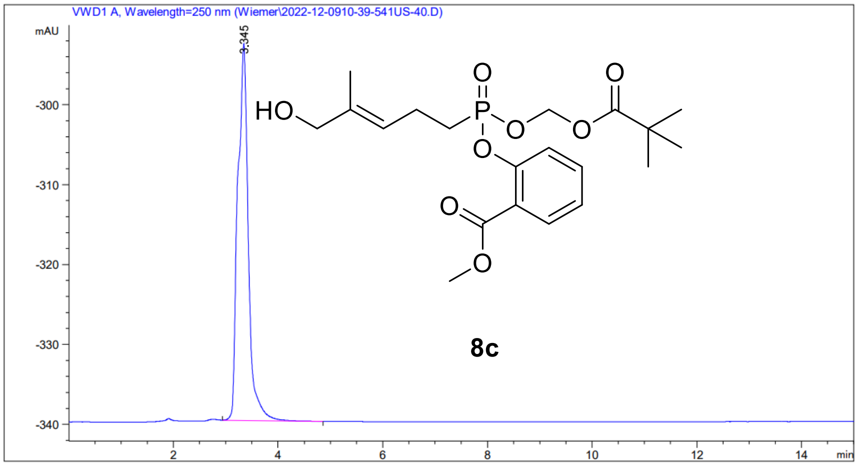

===
Area Percent Report
===

Sorted By : Signal
Multiplier : 1.0000
Dilution : 1.0000
Use Multiplier & Dilution Factor with ISTDs

Signal 1: VWD1 A, Wavelength=250 nm

Peak #	RetTime [min]	Type	Width [min]	Area [mAU*s]	Height [mAU]	Area %
1	3.345	BB	0.1944	663.87646	47.12072	100.0000
Totals :				663.87646	47.12072	

===
*** End of Report ***

Shared 1220 12/9/2022 10:54:59 AM SYSTEM Page 1 of 1

HPLC Chromatogram of Compound **8c**

Data File C:\Users\Public\Documents\ChemStation\1\Data\Wiemer\2022-12-0909-57-001US-43.D
Sample Name: US-43

===
Acq. Operator   : SYSTEM
Sample Operator : SYSTEM
Acq. Instrument : Shared 1220   Location :   -
Injection Date : 12/9/2022 9:57:00 AM   Inj :   1
                                               Inj Volume : No inj
Method   : C:\Users\Public\Documents\ChemStation\1\Methods\US M-1.M
Last changed   : 12/9/2022 9:27:23 AM by SYSTEM
                  (modified after loading)
Sample Info   : US-43

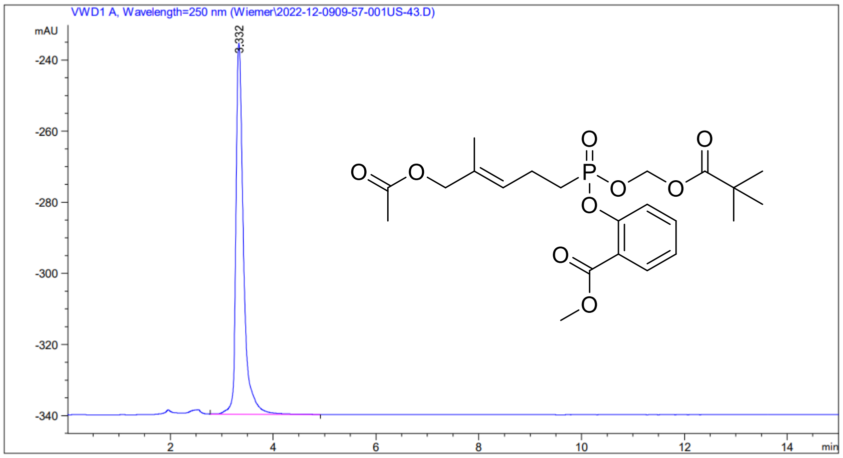

===
Area Percent Report
===

Sorted By : Signal
Multiplier : 1.0000
Dilution : 1.0000
Use Multiplier & Dilution Factor with ISTDs

Signal 1: VWD1 A, Wavelength=250 nm

Peak #	RetTime [min]	Type	Width [min]	Area [mAU*s]	Height [mAU]	Area %
1	3.332	BB	0.1545	1068.05627	104.27372	100.0000

Totals : 1068.05627 104.27372

===
*** End of Report ***

Shared 1220 12/9/2022 10:12:05 AM SYSTEM   Page 1 of 1

HPLC Chromatogram of Compound **9c**

```
=====================================================================
Acq. Operator   : SYSTEM
Sample Operator : SYSTEM
Acq. Instrument : Shared 1220                    Location :     -
Injection Date  : 1/4/2023 12:53:51 PM                Inj :   1
                                               Inj Volume : No inj
Method          : C:\Users\Public\Documents\ChemStation\1\Methods\US M-1.M
Last changed    : 12/9/2022 11:04:57 AM by SYSTEM
Sample Info     : US-51
```

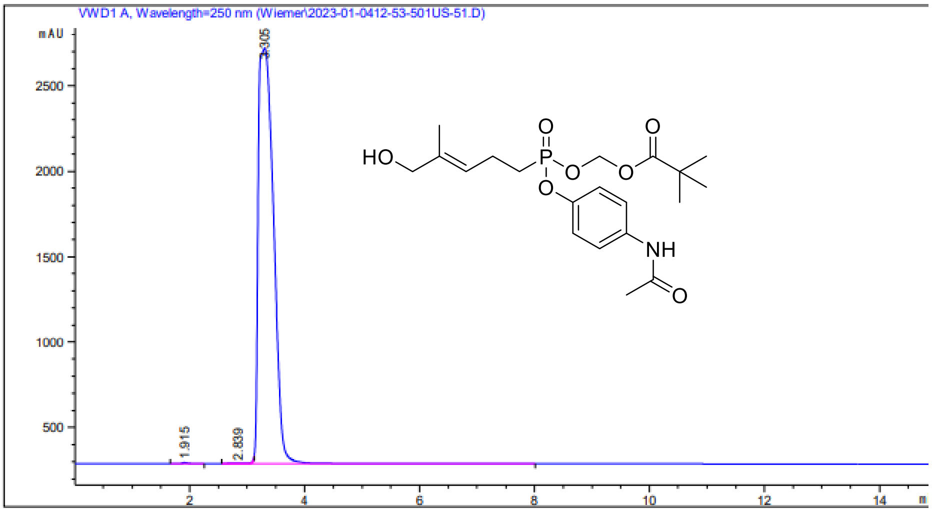


```
=====================================================================
                         Area Percent Report
=====================================================================

Sorted By             :      Signal
Multiplier            :      1.0000
Dilution              :      1.0000
Use Multiplier & Dilution Factor with ISTDs


Signal 1: VWD1 A, Wavelength=250 nm
```

Peak #	RetTime [min]	Type	Width [min]	Area [mAU*s]	Height [mAU]	Area %
1	1.915	BB	0.0711	29.07980	5.84159	0.0654
2	2.839	BV E	0.3380	60.14955	2.51493	0.1353
3	3.305	VB R	0.2760	4.43667e4	2430.15991	99.7993

Totals : 4.44559e4 2438.51643

HPLC Chromatogram of Compound **8d**

===

Acq. Operator : SYSTEM
Sample Operator : SYSTEM
Acq. Instrument : Shared 1220 Location : -
Injection Date : 1/4/2023 1:12:47 PM Inj : 1
Inj Volume : No inj
Method : C:\Users\Public\Documents\ChemStation\1\Methods\US M-1.M
Last changed : 12/9/2022 11:04:57 AM by SYSTEM
Sample Info : US-54

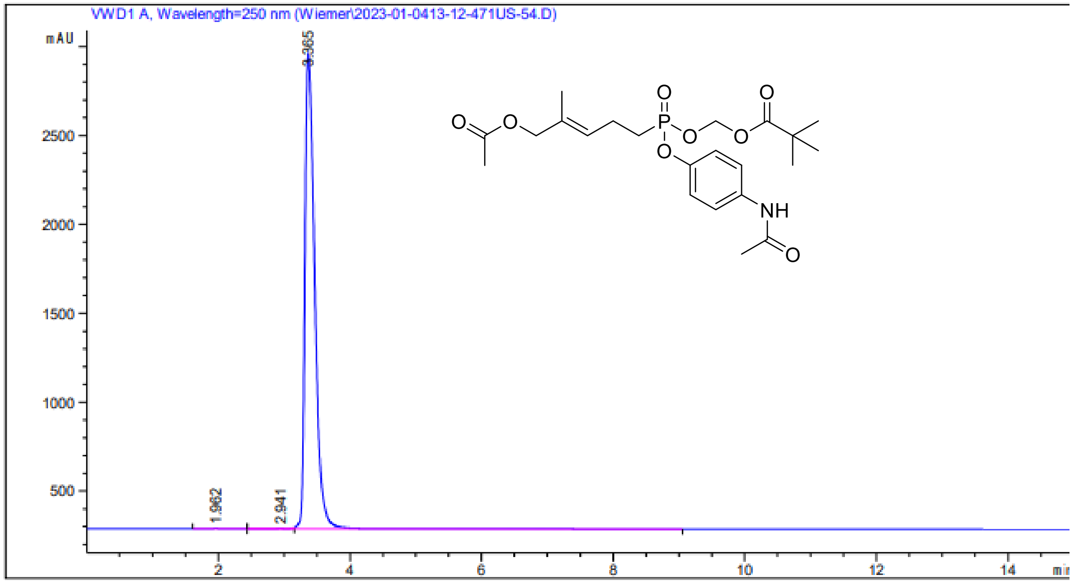

===

Area Percent Report

===

Sorted By : Signal
Multiplier : 1.0000
Dilution : 1.0000
Use Multiplier & Dilution Factor with ISTDs

Signal 1: VWD1 A, Wavelength=250 nm

Peak #	RetTime [min]	Type	Width [min]	Area [mAU*s]	Height [mAU]	Area %
1	1.962	BB	0.0790	23.85548	4.21779	0.0866
2	2.941	BV E	0.1952	36.65286	2.77333	0.1330
3	3.365	VB R	0.1610	2.74940e4	2673.00684	99.7804

Totals : 2.75545e4 2679.99795

HPLC Chromatogram of Compound **9d**

```
=====================================================================
Acq. Operator   : SYSTEM
Sample Operator : SYSTEM
Acq. Instrument : Shared 1220                    Location :    -
Injection Date  : 1/4/2023 1:39:22 PM                 Inj :   1
                                               Inj Volume : No inj
Method          : C:\Users\Public\Documents\ChemStation\1\Methods\US M-1.M
Last changed    : 12/9/2022 11:04:57 AM by SYSTEM
Sample Info     : US-56
```

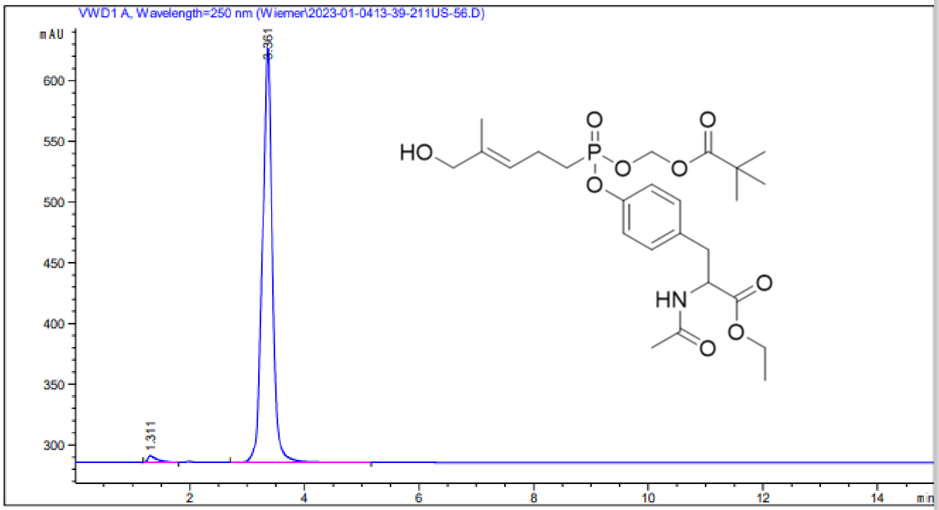


```
=====================================================================
                         Area Percent Report
=====================================================================

Sorted By             :      Signal
Multiplier            :      1.0000
Dilution              :      1.0000
Use Multiplier & Dilution Factor with ISTDs


Signal 1: VWD1 A, Wavelength=250 nm

Peak RetTime Type  Width     Area      Height     Area
  #   [min]        [min]   [mAU*s]     [mAU]       %
----|-------|----|-------|----------|----------|--------|
   1   1.311 BB    0.1479   58.00119    5.46287   1.4017
   2   3.361 BB    0.1708 4079.90356  340.47836  98.5983

Totals :                  4137.90475  345.94123
```

HPLC Chromatogram of Compound **8e**

```
=====================================================================
Acq. Operator   : SYSTEM
Sample Operator : SYSTEM
Acq. Instrument : Shared 1220                      Location :     -
Injection Date  : 1/4/2023 2:04:08 PM                   Inj :    1
                                                 Inj Volume : No inj
Method          : C:\Users\Public\Documents\ChemStation\1\Methods\US M-1.M
Last changed    : 12/9/2022 11:04:57 AM by SYSTEM
Sample Info     : US-59
```

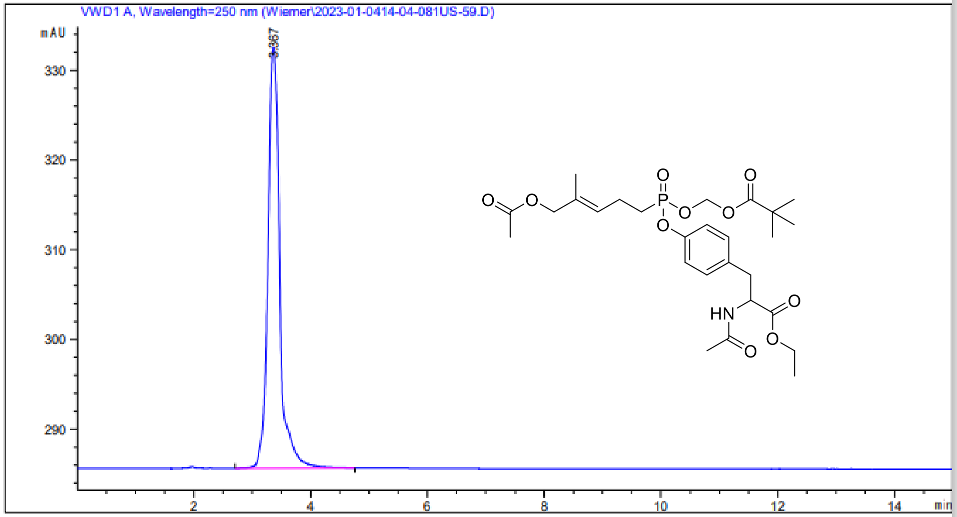


```
=====================================================================
                         Area Percent Report
=====================================================================

Sorted By             :      Signal
Multiplier            :      1.0000
Dilution              :      1.0000
Use Multiplier & Dilution Factor with ISTDs


Signal 1: VWD1 A, Wavelength=250 nm

Peak RetTime Type  Width     Area      Height     Area
  #   [min]        [min]   [mAU*s]     [mAU]        %
----|-------|----|-------|----------|----------|--------|
   1   3.367 BB    0.1982  659.68207   46.87363 100.0000

Totals :                   659.68207   46.87363
```

HPLC Chromatogram of Compound **9e**

Data File C:\Users\Public\Documents\ChemStation\1\Data\Wiemer\2023-03-0316-40-061US-79-2.D
Sample Name: US-79-2

===

Acq. Operator : SYSTEM
Sample Operator : SYSTEM
Acq. Instrument : Shared 1220 Location : -
Injection Date : 3/3/2023 4:40:07 PM Inj : 1
Inj Volume : No inj
Method : C:\Users\Public\Documents\ChemStation\1\Methods\US M-1.M
Last changed : 3/3/2023 4:21:06 PM by SYSTEM
(modified after loading)
Sample Info : US-79-2

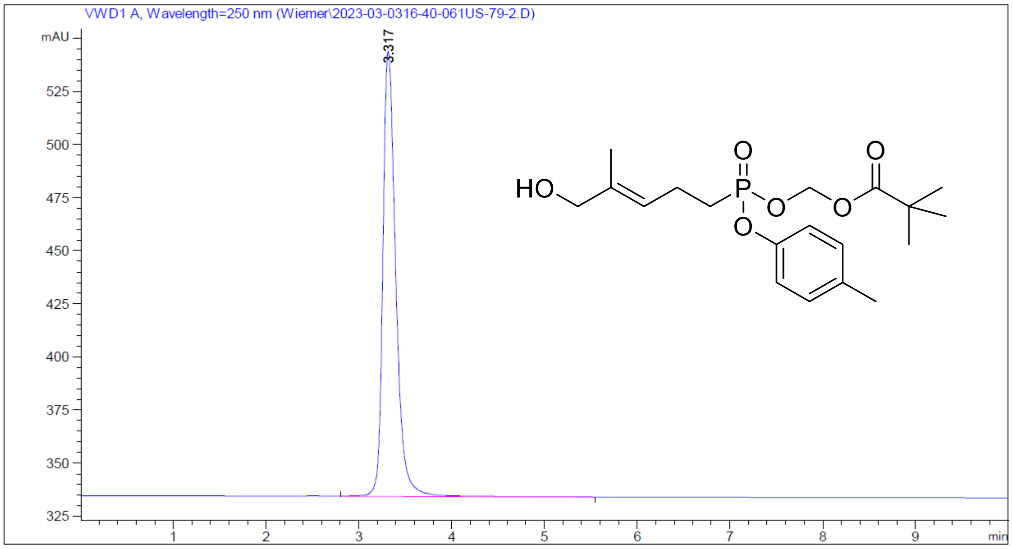

===

Area Percent Report

===

Sorted By : Signal
Multiplier : 1.0000
Dilution : 1.0000
Use Multiplier & Dilution Factor with ISTDs

Signal 1: VWD1 A, Wavelength=250 nm

Peak #	RetTime [min]	Type	Width [min]	Area [mAU*s]	Height [mAU]	Area %
1	3.317	BB	0.1476	2024.03870	209.63104	100.0000

Totals : 2024.03870 209.63104

===

*** End of Report ***

Shared 1220 3/3/2023 4:50:11 PM SYSTEM Page 1 of 1

HPLC Chromatogram of Compound **8f**

Data File C:\Users\Public\Documents\ChemStation\1\Data\Wiemer\2023-03-0412-51-101US-80.D
Sample Name: US-80

===

Acq. Operator : SYSTEM
Sample Operator : SYSTEM
Acq. Instrument : Shared 1220 Location : -
Injection Date : 3/4/2023 12:51:10 PM Inj : 1
Inj Volume : No inj
Method : C:\Users\Public\Documents\ChemStation\1\Methods\US M-1.M
Last changed : 3/4/2023 12:45:11 PM by SYSTEM
(modified after loading)
Sample Info : US-80

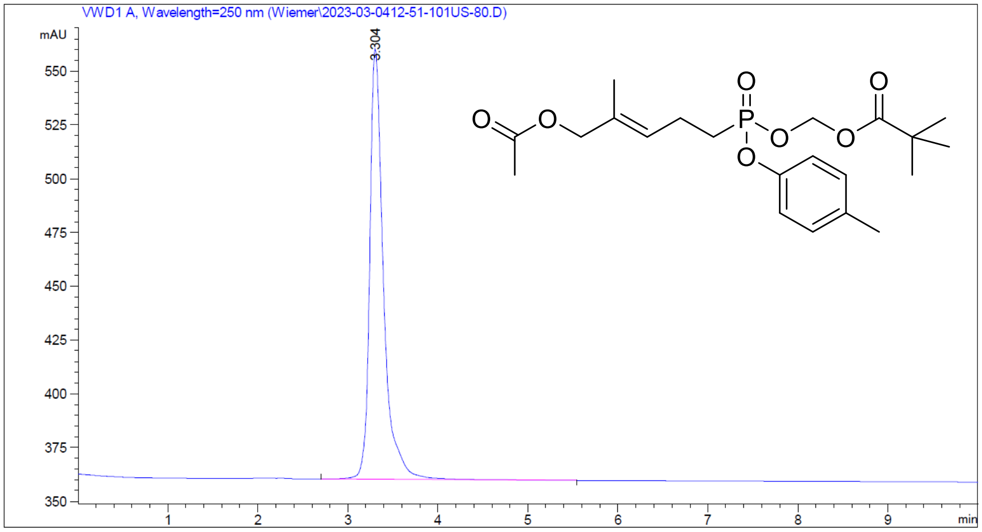

===

Area Percent Report

===

Sorted By : Signal
Multiplier : 1.0000
Dilution : 1.0000
Use Multiplier & Dilution Factor with ISTDs

Signal 1: VWD1 A, Wavelength=250 nm

Peak #	RetTime [min]	Type	Width [min]	Area [mAU*s]	Height [mAU]	Area %
1	3.304	BBA	0.1605	2135.22412	200.09702	100.0000

Totals : 2135.22412 200.09702

===

*** End of Report ***

HPLC Chromatogram of Compound **9f**

Data File C:\Users\Public\Documents\ChemStation\1\Data\Wiemer\2022-12-0910-19-031US-39.D
Sample Name: US-39

===
Acq. Operator : SYSTEM
Sample Operator : SYSTEM
Acq. Instrument : Shared 1220 Location : -
Injection Date : 12/9/2022 10:19:03 AM Inj : 1
Inj Volume : No inj
Method : C:\Users\Public\Documents\ChemStation\1\Methods\US M-1.M
Last changed : 12/9/2022 9:27:23 AM by SYSTEM
(modified after loading)
Sample Info : US-39

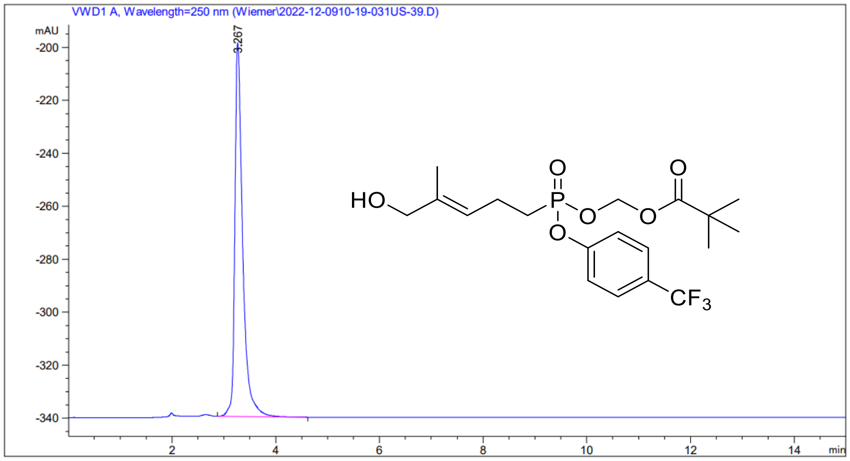

===
Area Percent Report
===

Sorted By : Signal
Multiplier : 1.0000
Dilution : 1.0000
Use Multiplier & Dilution Factor with ISTDs

Signal 1: VWD1 A, Wavelength=250 nm

Peak #	RetTime [min]	Type	Width [min]	Area [mAU*s]	Height [mAU]	Area %
1	3.267	BB	0.1546	1480.69641	140.90855	100.0000

Totals : 1480.69641 140.90855

===
*** End of Report ***

Shared 1220 12/9/2022 10:34:08 AM SYSTEM Page 1 of 1

HPLC Chromatogram of Compound **8g**

```
Data File C:\Users\Public\Documents\ChemStation\1\Data\Wiemer\2022-12-0909-34-041US-44-a.D
Sample Name: US-44-a

    =====================================================================
    Acq. Operator   : SYSTEM
    Sample Operator : SYSTEM
    Acq. Instrument : Shared 1220                    Location :     -
    Injection Date  : 12/9/2022 9:34:04 AM                Inj :    1
                                                   Inj Volume : No inj
    Method          : C:\Users\Public\Documents\ChemStation\1\Methods\US M-1.M
    Last changed    : 12/9/2022 9:27:23 AM by SYSTEM
                      (modified after loading)
    Sample Info     : US-44-a
```

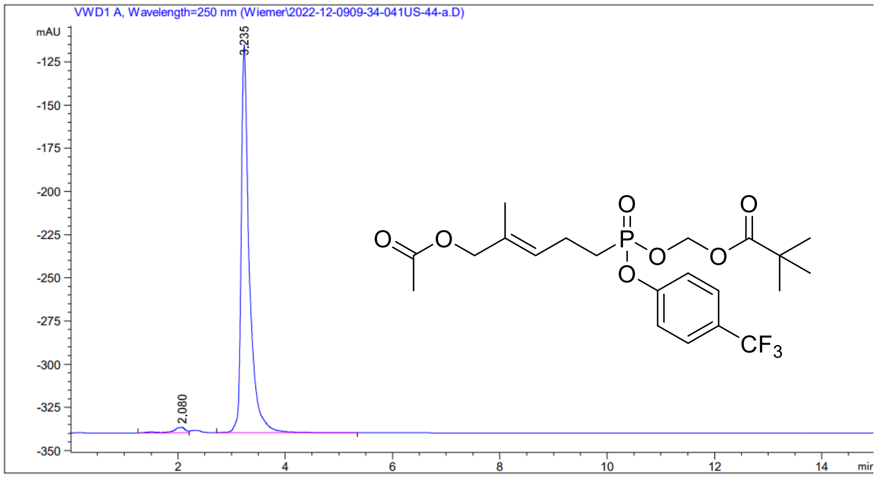


```
    =====================================================================
                             Area Percent Report
    =====================================================================

    Sorted By             :      Signal
    Multiplier            :      1.0000
    Dilution              :      1.0000
    Use Multiplier & Dilution Factor with ISTDs


    Signal 1: VWD1 A, Wavelength=250 nm

    Peak RetTime Type  Width     Area      Height     Area
      #   [min]        [min]   [mAU*s]     [mAU]        %
    ----|-------|----|-------|----------|----------|--------|
       1   2.080 VV R  0.2043   50.08727    3.10399   2.0761
       2   3.235 BB    0.1538 2362.45532  224.43343  97.9239

    Totals :                  2412.54259  227.53742


    =====================================================================
```


HPLC Chromatogram of Compound **9g**

Data File C:\Users\P...c\Documents\ChemStation\1\Data\Wiemer\2023-06-0614-47-401US-135-2.D
Sample Name: US-135-2

```
=====================================================================
Acq. Operator   : SYSTEM
Sample Operator : SYSTEM
Acq. Instrument : Shared 1220                    Location :     -
Injection Date  : 6/6/2023 2:47:40 PM                 Inj :   1
                                               Inj Volume : No inj
Acq. Method     : C:\Users\Public\Documents\ChemStation\1\Methods\US M-1.M
Last changed    : 6/6/2023 2:02:46 PM by SYSTEM
                  (modified after loading)
Analysis Method : C:\Users\Public\Documents\ChemStation\1\Methods\US M-1.M
Last changed    : 1/12/2023 1:10:48 PM by SYSTEM
Sample Info     : US-135-2

Additional Info : Peak(s) manually integrated
```

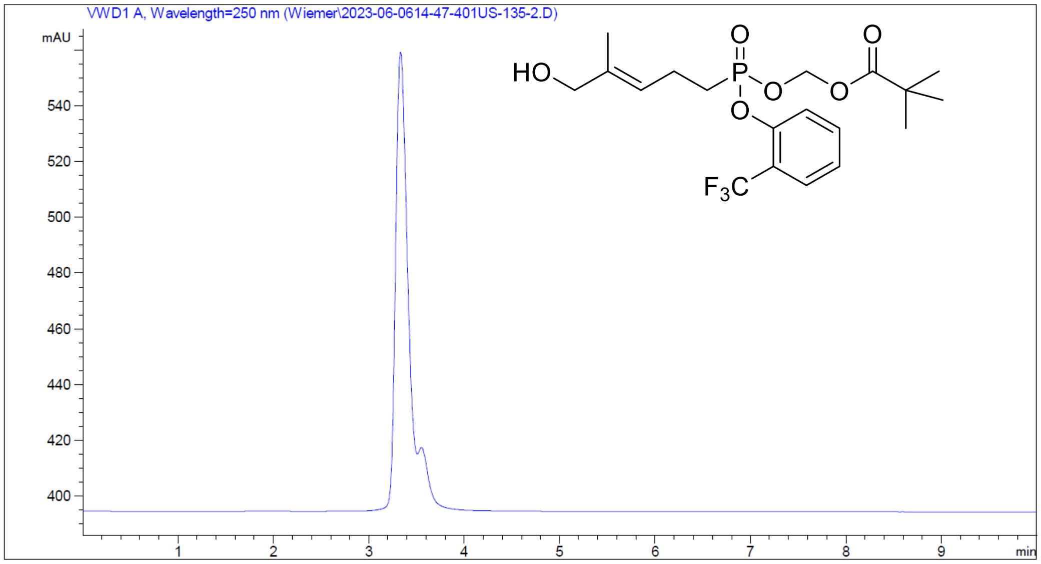


```
=====================================================================
                     Area Percent Report
=====================================================================

Sorted By             :      Signal
Multiplier            :      1.0000
Dilution              :      1.0000
Use Multiplier & Dilution Factor with ISTDs

No peaks found

=====================================================================
                         *** End of Report ***
```


HPLC Chromatogram of Compound **8h**

```
Data File C:\Users\P...c\Documents\ChemStation\1\Data\Wiemer\2023-06-0614-18-421US-137-2.D
Sample Name: US-137-2

=====================================================================
Acq. Operator   : SYSTEM
Sample Operator : SYSTEM
Acq. Instrument : Shared 1220                    Location :    -
Injection Date  : 6/6/2023 2:18:42 PM                 Inj :   1
                                               Inj Volume : No inj
Method          : C:\Users\Public\Documents\ChemStation\1\Methods\US M-1.M
Last changed    : 6/6/2023 2:02:46 PM by SYSTEM
                  (modified after loading)
Sample Info     : US-137-2
```

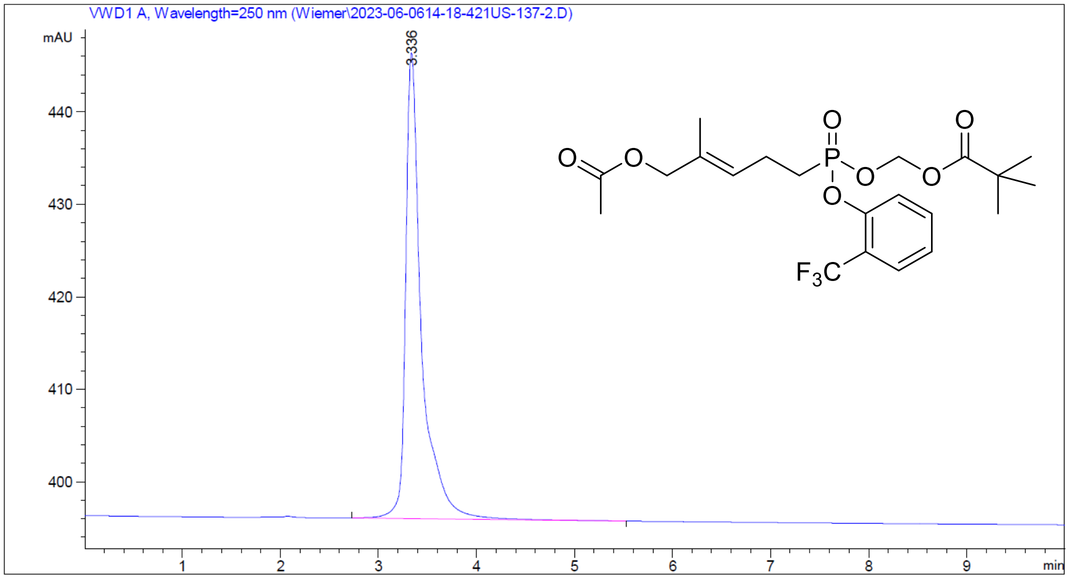


```
=====================================================================
                         Area Percent Report
=====================================================================

Sorted By             :      Signal
Multiplier            :      1.0000
Dilution              :      1.0000
Use Multiplier & Dilution Factor with ISTDs


Signal 1: VWD1 A, Wavelength=250 nm

Peak RetTime Type  Width     Area      Height     Area
  #   [min]        [min]   [mAU*s]     [mAU]        %
----|-------|----|-------|----------|----------|--------|
   1   3.336 BB    0.1649  569.00427   50.31355 100.0000

Totals :                   569.00427   50.31355

=====================================================================
                          *** End of Report ***
```

Shared 1220 6/6/2023 2:28:47 PM SYSTEM   Page   1 of 1

HPLC Chromatogram of Compound **9h**

Data File C:\Users\Public\Documents\ChemStation\1\Data\Wiemer\2023-03-2212-38-121US-92-2.D
Sample Name: US-92-2

```
=====================================================================
Acq. Operator   : SYSTEM
Sample Operator : SYSTEM
Acq. Instrument : Shared 1220                    Location :     -
Injection Date  : 3/22/2023 12:38:12 PM               Inj :   1
                                               Inj Volume : No inj
Method          : C:\Users\Public\Documents\ChemStation\1\Methods\US M-1.M
Last changed    : 3/22/2023 12:11:24 PM by SYSTEM
                  (modified after loading)
Sample Info     : US-92-2
```

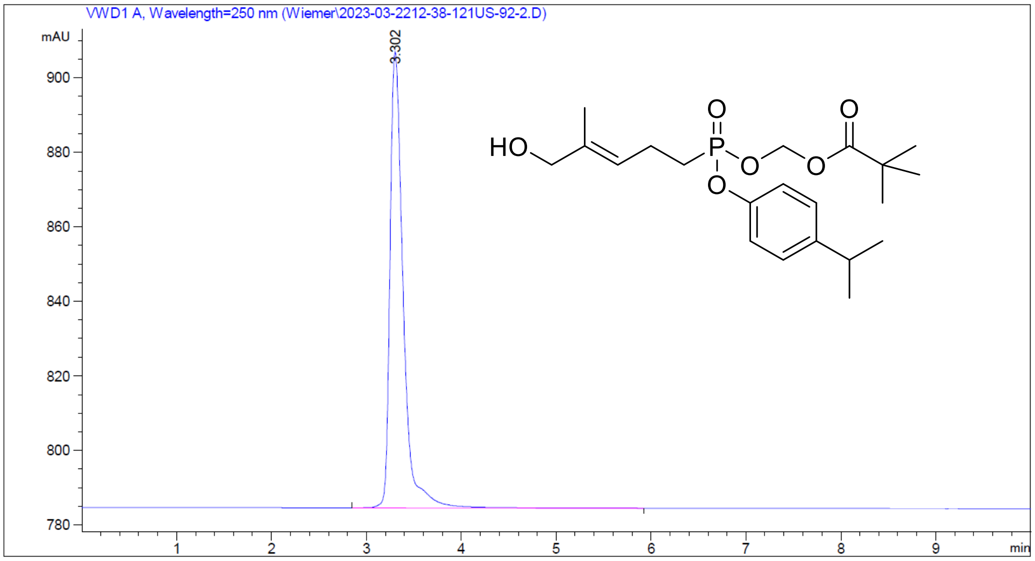


```
=====================================================================
                         Area Percent Report
=====================================================================

Sorted By             :      Signal
Multiplier            :      1.0000
Dilution              :      1.0000
Use Multiplier & Dilution Factor with ISTDs


Signal 1: VWD1 A, Wavelength=250 nm

Peak RetTime Type  Width     Area      Height     Area
  #   [min]        [min]   [mAU*s]     [mAU]       %
----|-------|----|-------|----------|----------|--------|
   1   3.302 BB    0.1465 1180.43372  122.36578 100.0000

Totals :                  1180.43372  122.36578


=====================================================================
                          *** End of Report ***
```


HPLC Chromatogram of Compound **8i**

Data File C:\Users\Public\Documents\ChemStation\1\Data\Wiemer\2023-03-2212-51-181US-95.D
Sample Name: US-95

===

Acq. Operator : SYSTEM
Sample Operator : SYSTEM
Acq. Instrument : Shared 1220 Location : -
Injection Date : 3/22/2023 12:51:18 PM Inj : 1
Inj Volume : No inj
Method : C:\Users\Public\Documents\ChemStation\1\Methods\US M-1.M
Last changed : 3/22/2023 12:11:24 PM by SYSTEM
(modified after loading)
Sample Info : US-95

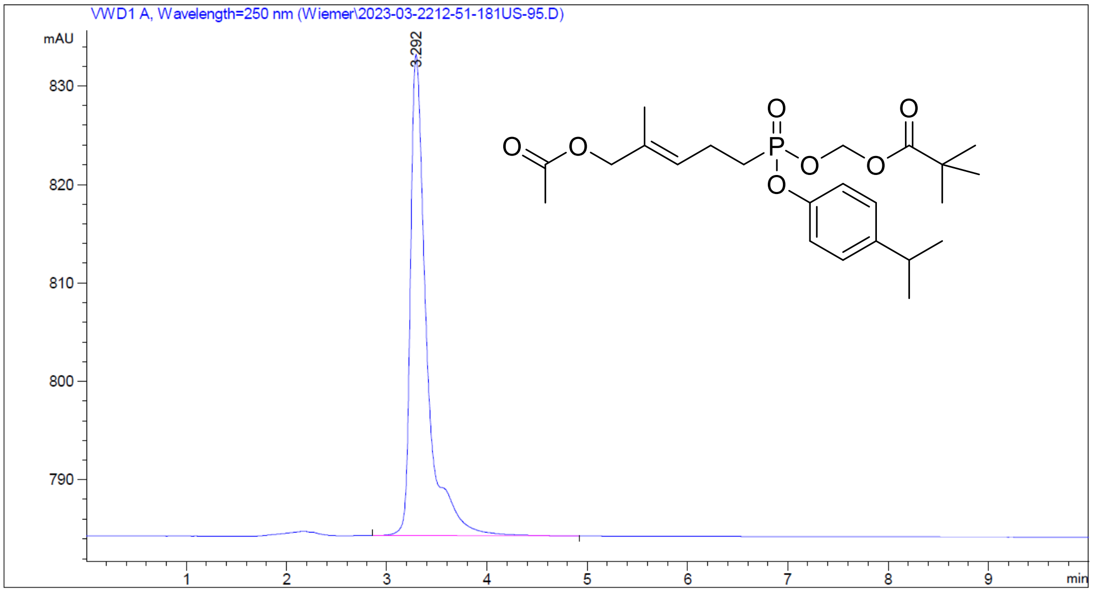

===

Area Percent Report

===

Sorted By : Signal
Multiplier : 1.0000
Dilution : 1.0000
Use Multiplier & Dilution Factor with ISTDs

Signal 1: VWD1 A, Wavelength=250 nm

Peak #	RetTime [min]	Type	Width [min]	Area [mAU*s]	Height [mAU]	Area %
1	3.292	BB	0.1654	550.21790	48.86282	100.0000

Totals : 550.21790 48.86282

===

*** End of Report ***

Shared 1220 3/22/2023 1:01:23 PM SYSTEM Page 1 of 1

HPLC Chromatogram of Compound **9i**

Data File C:\Users\Public\Documents\ChemStation\1\Data\Wiemer\2023-04-2010-46-491US-110.D
Sample Name: US-110

===

Acq. Operator : SYSTEM
Sample Operator : SYSTEM
Acq. Instrument : Shared 1220 Location : -
Injection Date : 4/20/2023 10:46:49 AM Inj : 1
Inj Volume : No inj
Method : C:\Users\Public\Documents\ChemStation\1\Methods\US M-1.M
Last changed : 4/20/2023 9:18:58 AM by SYSTEM
(modified after loading)
Sample Info : US-110

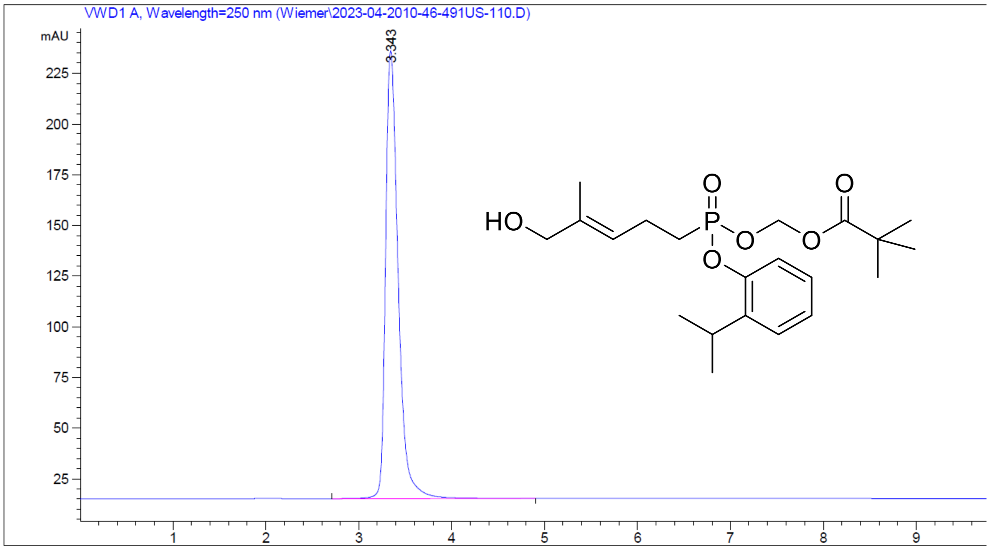

===

Area Percent Report

===

Sorted By : Signal
Multiplier : 1.0000
Dilution : 1.0000
Use Multiplier & Dilution Factor with ISTDs

Signal 1: VWD1 A, Wavelength=250 nm

Peak #	RetTime [min]	Type	Width [min]	Area [mAU*s]	Height [mAU]	Area %
1	3.343	BB	0.1451	2083.60815	220.84514	100.0000
Totals :				2083.60815	220.84514	

===

*** End of Report ***

Shared 1220 4/20/2023 10:56:54 AM SYSTEM Page 1 of 1

HPLC Chromatogram of Compound **8j**

```
Data File C:\Users\P...c\Documents\ChemStation\1\Data\Wiemer\2023-04-2013-23-021US-111-3.D
Sample Name: US-111-3

    =====================================================================
    Acq. Operator   : SYSTEM
    Sample Operator : SYSTEM
    Acq. Instrument : Shared 1220                    Location :     -
    Injection Date  : 4/20/2023 1:23:03 PM                Inj :   1
                                                   Inj Volume : No inj
    Method          : C:\Users\Public\Documents\ChemStation\1\Methods\US M-1.M
    Last changed    : 4/20/2023 1:14:51 PM by SYSTEM
                      (modified after loading)
    Sample Info     : US-111-3
```

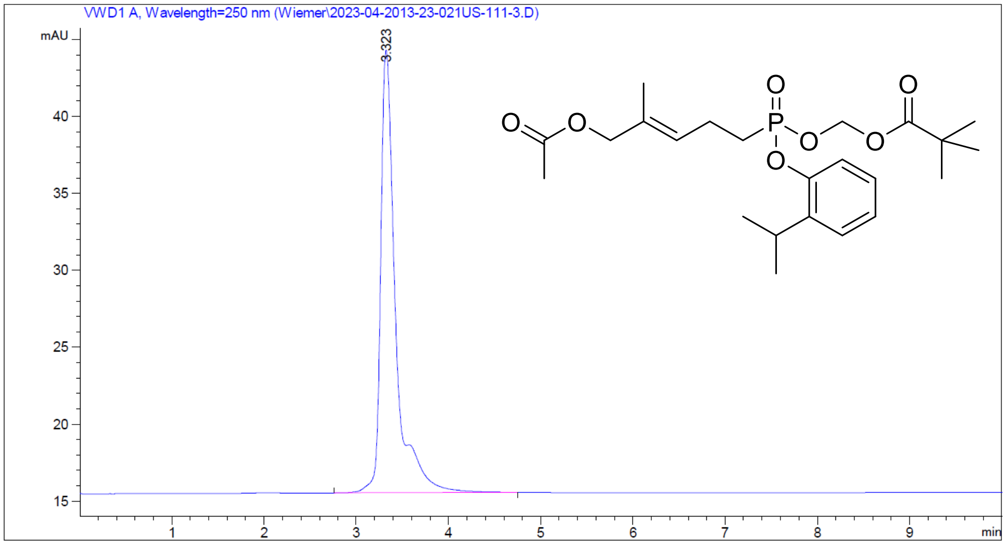


```
    =====================================================================
                             Area Percent Report
    =====================================================================

    Sorted By             :      Signal
    Multiplier            :      1.0000
    Dilution              :      1.0000
    Use Multiplier & Dilution Factor with ISTDs


    Signal 1: VWD1 A, Wavelength=250 nm

    Peak RetTime Type  Width     Area      Height     Area
      #   [min]        [min]   [mAU*s]     [mAU]        %
    ----|-------|----|-------|----------|----------|--------|
       1   3.323 BV R  0.1674  324.11255   28.77904 100.0000

    Totals :                   324.11255   28.77904


    =====================================================================
                              *** End of Report ***

Shared 1220 4/20/2023 1:33:08 PM SYSTEM                                Page    1 of 1
```

HPLC Chromatogram of Compound **9j**

===

Acq. Operator : SYSTEM
Sample Operator : SYSTEM
Acq. Instrument : Shared 1220 Location : -
Injection Date : 1/16/2023 3:58:10 PM Inj : 1
Inj Volume : No inj
Method : C:\Users\Public\Documents\ChemStation\1\Methods\US M-1.M
Last changed : 1/16/2023 3:37:50 PM by SYSTEM
(modified after loading)
Sample Info : US-62-2

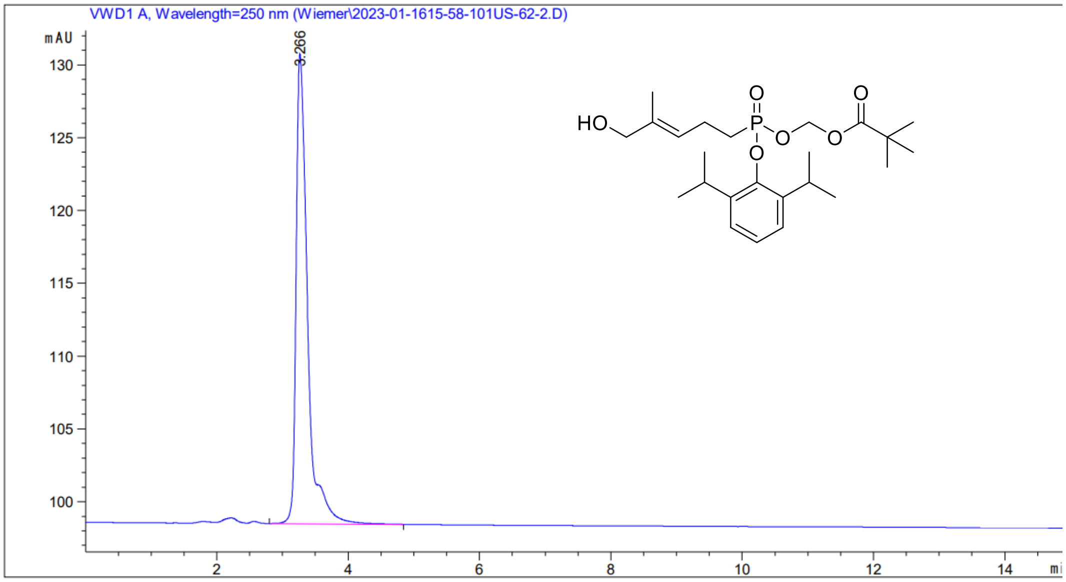

===

Area Percent Report

===

Sorted By : Signal
Multiplier : 1.0000
Dilution : 1.0000
Use Multiplier & Dilution Factor with ISTDs

Signal 1: VWD1 A, Wavelength=250 nm

Peak #	RetTime [min]	Type	Width [min]	Area [mAU*s]	Height [mAU]	Area %
1	3.266	BB	0.1887	389.26984	32.26735	100.0000

Totals : 389.26984 32.26735

===

*** End of Report ***

Shared 1220 1/16/2023 4:13:15 PM SYSTEM Page 1 of 1

HPLC Chromatogram of Compound **8k**

```
=====================================================================
Acq. Operator   : SYSTEM
Sample Operator : SYSTEM
Acq. Instrument : Shared 1220                    Location :     -
Injection Date  : 2/7/2023 4:08:41 PM                 Inj :   1
                                               Inj Volume : No inj
Acq. Method     : C:\Users\Public\Documents\ChemStation\1\Methods\US M-1.M
Last changed    : 2/7/2023 3:59:21 PM by SYSTEM
                  (modified after loading)
Analysis Method : C:\Users\Public\Documents\ChemStation\1\Methods\US M-1.M
Last changed    : 2/7/2023 4:20:09 PM by SYSTEM
                  (modified after loading)
Sample Info     : US-72-3

Additional Info : Peak(s) manually integrated
```

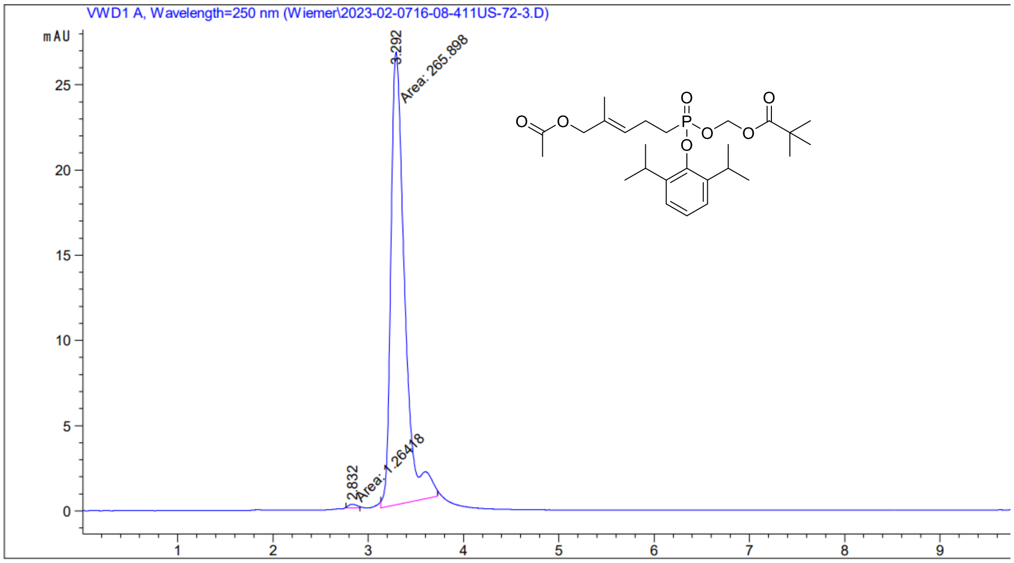


```
=====================================================================
                      Area Percent Report
=====================================================================

Sorted By             :      Signal
Multiplier            :      1.0000
Dilution              :      1.0000
Use Multiplier & Dilution Factor with ISTDs


Signal 1: VWD1 A, Wavelength=250 nm
```

Peak #	RetTime [min]	Type	Width [min]	Area [mAU*s]	Height [mAU]	Area %
1	2.832	MM	0.1035	1.26418	2.03546e-1	0.4732
2	3.292	MM	0.1669	265.89816	26.55308	99.5268

HPLC Chromatogram of Compound **9k**

```
Data File C:\Users\Public\Documents\ChemStation\1\Data\Wiemer\2023-02-1113-01-571US-73.D
Sample Name: US-73

    =====================================================================
    Acq. Operator   : SYSTEM
    Sample Operator : SYSTEM
    Acq. Instrument : Shared 1220                    Location :     -
    Injection Date  : 2/11/2023 1:01:58 PM                Inj :   1
                                                   Inj Volume : No inj
    Method          : C:\Users\Public\Documents\ChemStation\1\Methods\US M-1.M
    Last changed    : 2/11/2023 12:53:27 PM by SYSTEM
                      (modified after loading)
    Sample Info     : US-73
```

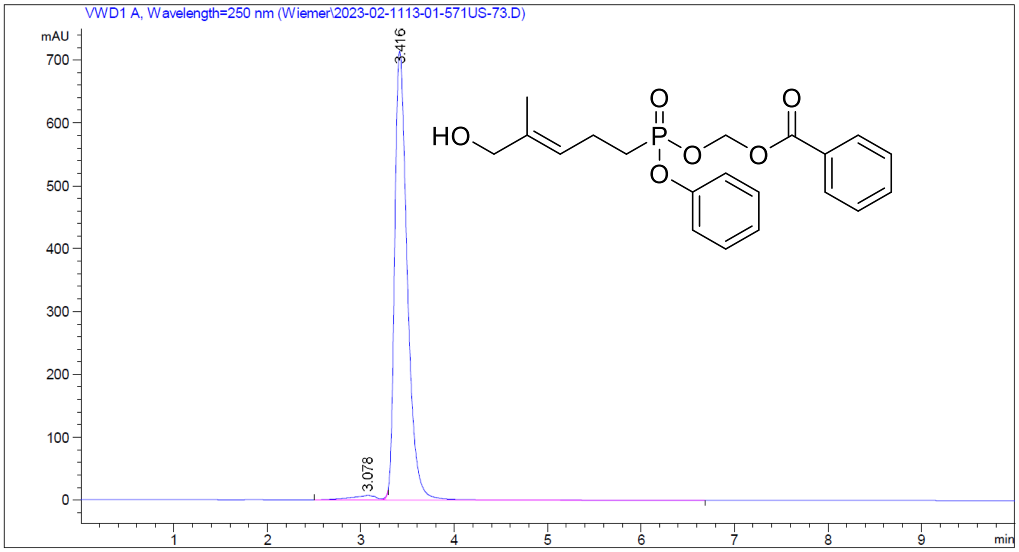


```
    =====================================================================
                             Area Percent Report
    =====================================================================

    Sorted By             :      Signal
    Multiplier            :      1.0000
    Dilution              :      1.0000
    Use Multiplier & Dilution Factor with ISTDs


    Signal 1: VWD1 A, Wavelength=250 nm

    Peak RetTime Type  Width     Area      Height     Area
      #   [min]        [min]   [mAU*s]     [mAU]        %
    ----|-------|----|-------|----------|----------|--------|
       1   3.078  BV E  0.2378  137.30270    7.35315   2.0040
       2   3.416  VB R  0.1437 6714.03613  714.03271  97.9960

    Totals :                   6851.33884  721.38586


    =====================================================================
```


HPLC Chromatogram of Compound **11x**

Data File C:\Users\Public\Documents\ChemStation\1\Data\Wiemer\2023-02-1410-55-051US-74.D
Sample Name: US-74

===

Acq. Operator : SYSTEM
Sample Operator : SYSTEM
Acq. Instrument : Shared 1220 Location : -
Injection Date : 2/14/2023 10:55:05 AM Inj : 1
Inj Volume : No inj
Method : C:\Users\Public\Documents\ChemStation\1\Methods\US M-1.M
Last changed : 2/14/2023 10:48:57 AM by SYSTEM
(modified after loading)
Sample Info : US-74

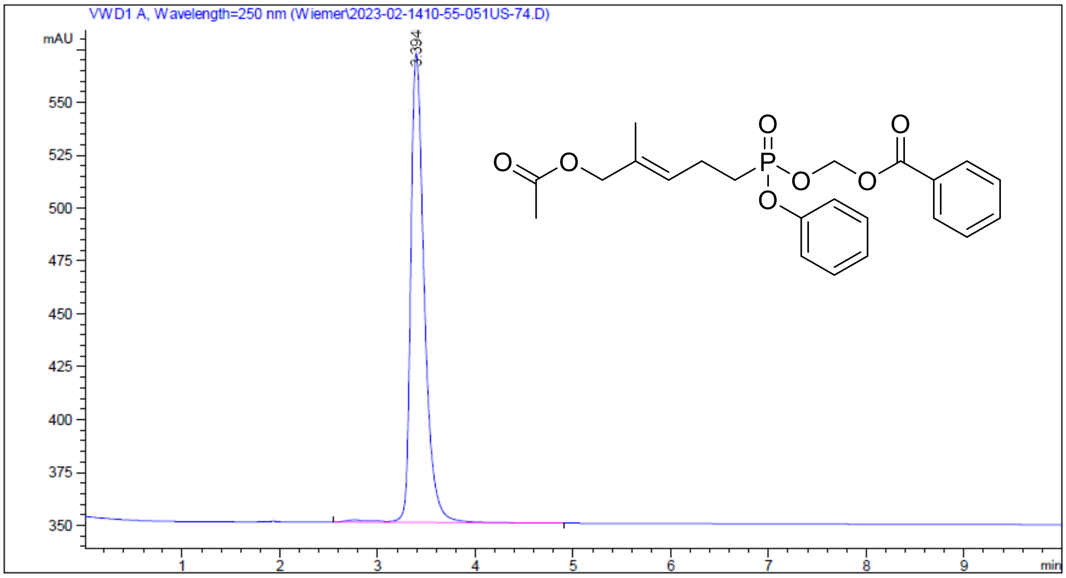

===

Area Percent Report

===

Sorted By : Signal
Multiplier : 1.0000
Dilution : 1.0000
Use Multiplier & Dilution Factor with ISTDs

Signal 1: VWD1 A, Wavelength=250 nm

Peak #	RetTime [min]	Type	Width [min]	Area [mAU*s]	Height [mAU]	Area %
1	3.394	VB R	0.1483	2154.03003	221.78348	100.0000

Totals : 2154.03003 221.78348

===

*** End of Report ***

Shared 1220 2/14/2023 11:05:13 AM SYSTEM Page 1 of 1

HPLC Chromatogram of Compound **12x**

```
Data File C:\Users\Public\Documents\ChemStation\1\Data\Wiemer\2023-04-2009-44-131US-104.D
Sample Name: US-104

    =====================================================================
    Acq. Operator   : SYSTEM
    Sample Operator : SYSTEM
    Acq. Instrument : Shared 1220                     Location :     -
    Injection Date  : 4/20/2023 9:44:13 AM                 Inj :   1
                                                    Inj Volume : No inj
    Method          : C:\Users\Public\Documents\ChemStation\1\Methods\US M-1.M
    Last changed    : 4/20/2023 9:18:58 AM by SYSTEM
                      (modified after loading)
    Sample Info     : US-104
```

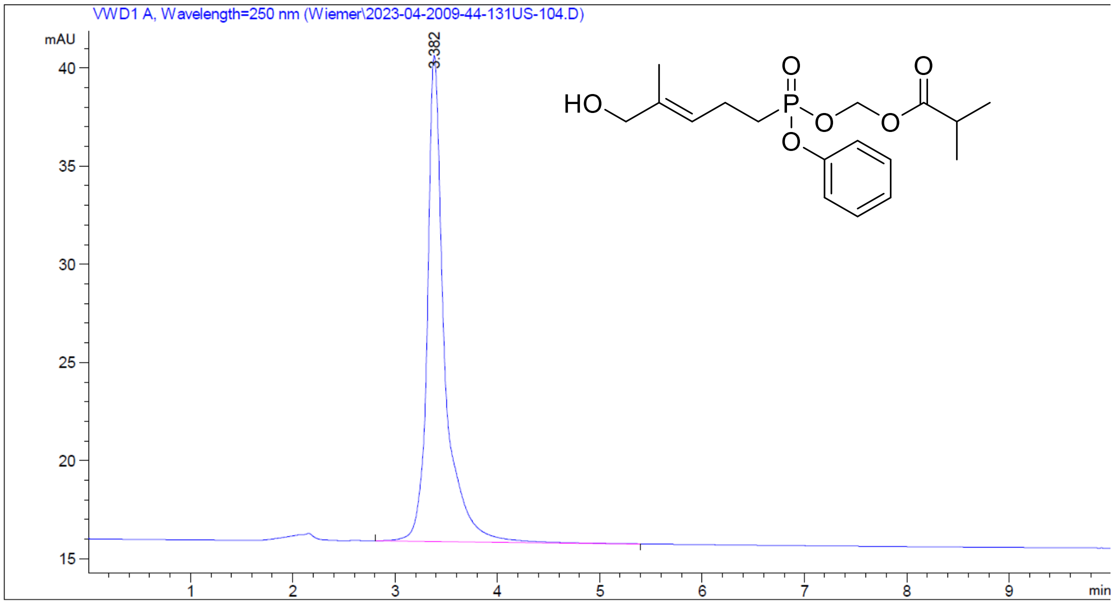


```
    =====================================================================
                             Area Percent Report
    =====================================================================

    Sorted By             :      Signal
    Multiplier            :      1.0000
    Dilution              :      1.0000
    Use Multiplier & Dilution Factor with ISTDs


    Signal 1: VWD1 A, Wavelength=250 nm

    Peak RetTime Type  Width     Area      Height     Area
      #   [min]        [min]   [mAU*s]     [mAU]        %
    ----|-------|----|-------|----------|----------|--------|
       1   3.382 BB    0.1695  289.40192   24.74163 100.0000

    Totals :                   289.40192   24.74163


    =====================================================================
                              *** End of Report ***
```

Shared 1220 4/20/2023 9:54:20 AM SYSTEM   Page   1 of 1

HPLC Chromatogram of Compound **11y**

Data File C:\Users\Public\Documents\ChemStation\1\Data\Wiemer\2023-04-2010-04-341US-109.D
Sample Name: US-109

===

Acq. Operator : SYSTEM
Sample Operator : SYSTEM
Acq. Instrument : Shared 1220 Location : -
Injection Date : 4/20/2023 10:04:34 AM Inj : 1
Inj Volume : No inj
Method : C:\Users\Public\Documents\ChemStation\1\Methods\US M-1.M
Last changed : 4/20/2023 9:18:58 AM by SYSTEM
(modified after loading)
Sample Info : US-109

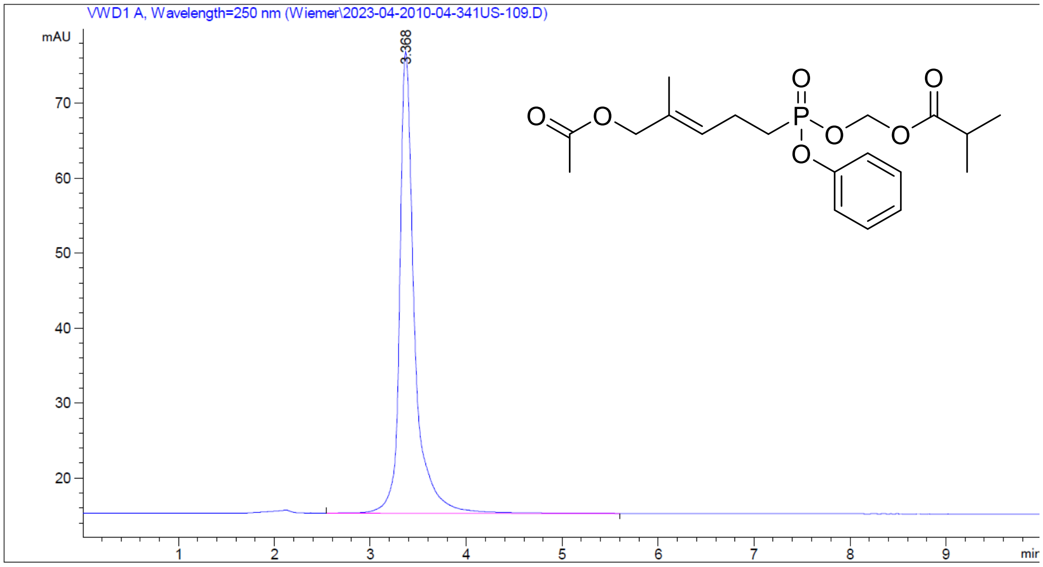

===

Area Percent Report

===

Sorted By : Signal
Multiplier : 1.0000
Dilution : 1.0000
Use Multiplier & Dilution Factor with ISTDs

Signal 1: VWD1 A, Wavelength=250 nm

Peak #	RetTime [min]	Type	Width [min]	Area [mAU*s]	Height [mAU]	Area %
1	3.368	BB	0.1599	663.51428	61.49715	100.0000

Totals : 663.51428 61.49715

===

*** End of Report ***

Shared 1220 4/20/2023 10:14:39 AM SYSTEM Page 1 of 1

HPLC Chromatogram of Compound **12y**

Data File C:\Users\Public\Documents\ChemStation\1\Data\Wiemer\2023-05-2209-06-521US-126.D
Sample Name: US-126

===

Acq. Operator : SYSTEM
Sample Operator : SYSTEM
Acq. Instrument : Shared 1220 Location : -
Injection Date : 5/22/2023 9:06:53 AM Inj : 1
Inj Volume : No inj
Method : C:\Users\Public\Documents\ChemStation\1\Methods\US M-1.M
Last changed : 5/22/2023 8:57:11 AM by SYSTEM
(modified after loading)
Sample Info : US-126

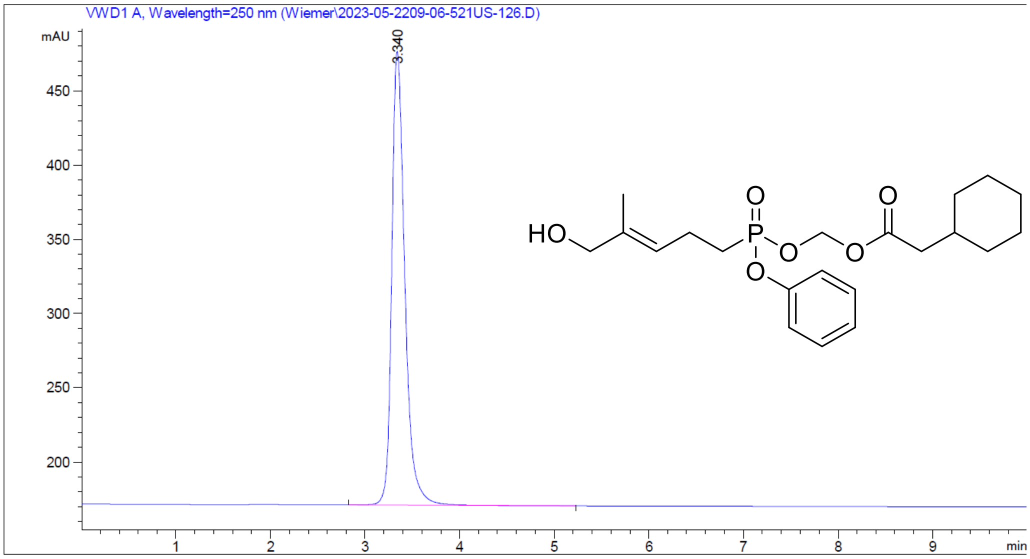

===

Area Percent Report

===

Sorted By : Signal
Multiplier : 1.0000
Dilution : 1.0000
Use Multiplier & Dilution Factor with ISTDs

Signal 1: VWD1 A, Wavelength=250 nm

Peak #	RetTime [min]	Type	Width [min]	Area [mAU*s]	Height [mAU]	Area %
1	3.340	BBA	0.1473	2940.54492	305.61176	100.0000

Totals : 2940.54492 305.61176

===

*** End of Report ***

Shared 1220 5/22/2023 9:17:00 AM SYSTEM Page 1 of 1

HPLC Chromatogram of Compound **11z**

Data File C:\Users\P...c\Documents\ChemStation\1\Data\Wiemer\2023-06-0112-59-541US-130-3.D
Sample Name: US-130-3

===

Acq. Operator : SYSTEM
Sample Operator : SYSTEM
Acq. Instrument : Shared 1220 Location : -
Injection Date : 6/1/2023 12:59:54 PM Inj : 1
Inj Volume : No inj
Method : C:\Users\Public\Documents\ChemStation\1\Methods\US M-1.M
Last changed : 6/1/2023 12:11:52 PM by SYSTEM
(modified after loading)
Sample Info : US-130-3

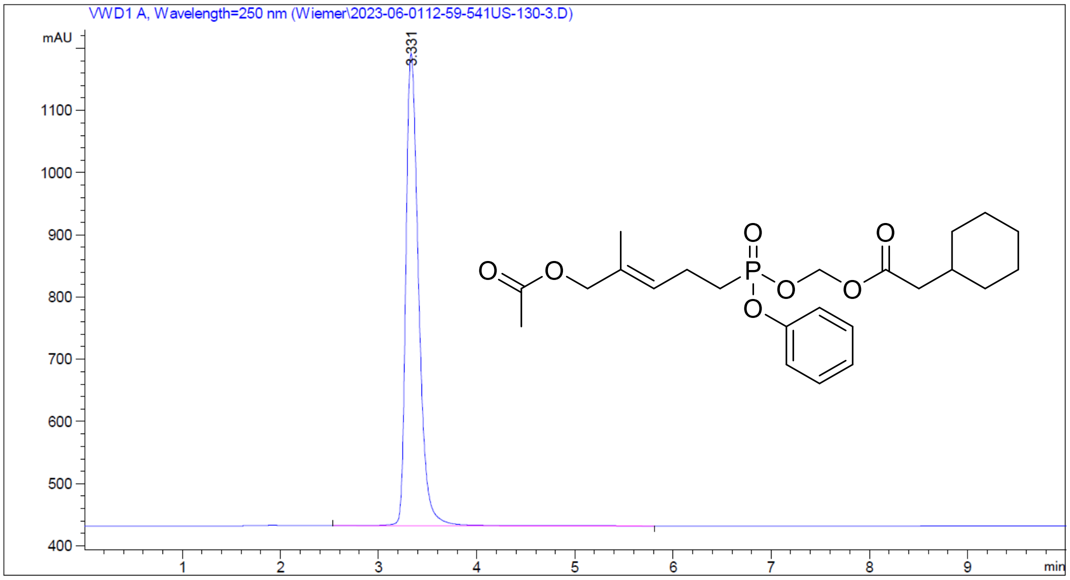

===

Area Percent Report

===

Sorted By : Signal
Multiplier : 1.0000
Dilution : 1.0000
Use Multiplier & Dilution Factor with ISTDs

Signal 1: VWD1 A, Wavelength=250 nm

Peak #	RetTime [min]	Type	Width [min]	Area [mAU*s]	Height [mAU]	Area %
1	3.331	BB	0.1392	6853.97021	759.88025	100.0000

Totals : 6853.97021 759.88025

===

*** End of Report ***

HPLC Chromatogram of Compound **12z**

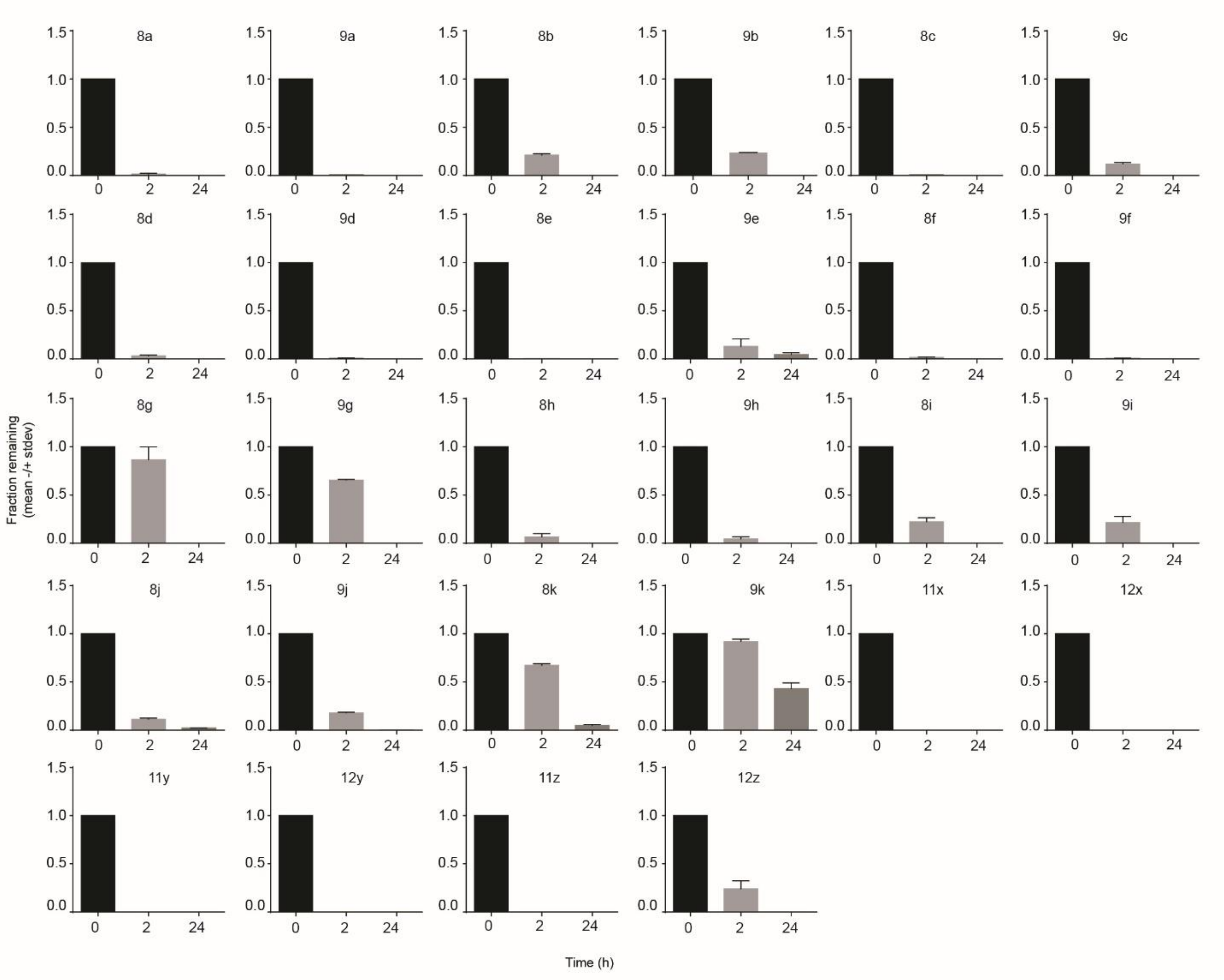

Figure S1. Stability in 50% human plasma of all tested compounds at 2- and 24-hour time points.

K562 cells uptake and metabolism study

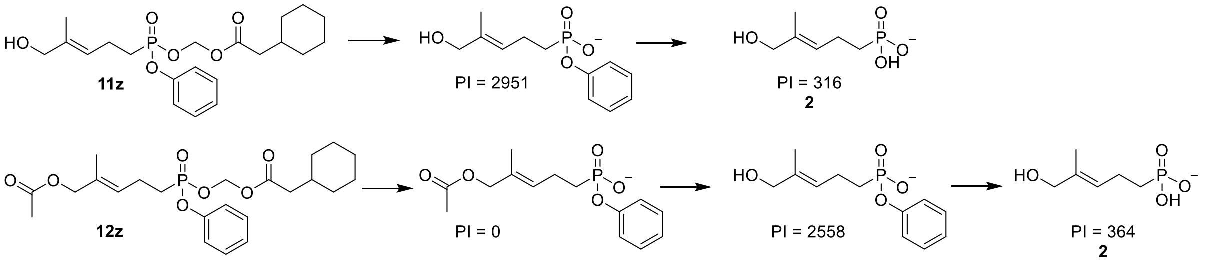

Plasma metabolism study

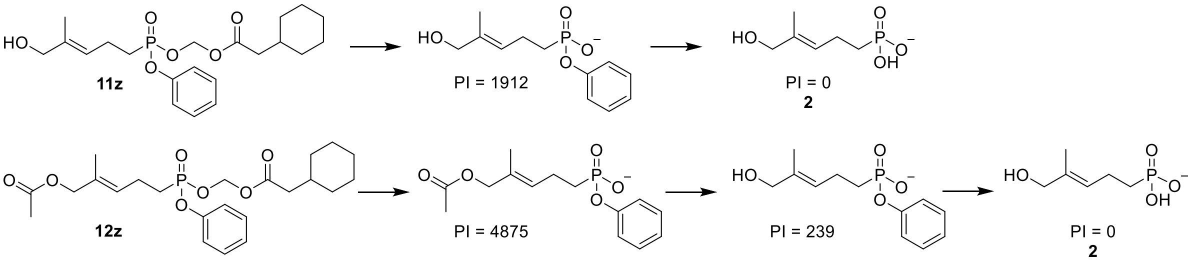

Figure S2. K562 and plasma metabolism study of compounds **11z** and **12z** at the 1 hour time point.

Table S1. K562 uptake and metabolism data.[1]

Comp		M_{mi}	t_R	*m/z*	PI	PI_2/PI_{ma}[2]
**8a**	Monoanion	255.0792	1.34, 2.63	255.0808	28733	0.13
	Cmpd **2**	179.0479	1.29	179.0486	3644	
**9a**	Ac-monoanion	297.0897	2.95	297.0894	695	
	Monoanion	255.0792	1.34, 2.63	255.0808	30734	0.14
	Cmpd **2**	179.0479	1.29	179.0486	4176	
**11x**	Monoanion	255.0792	1.34, 2.63	255.0808	35586	0.12
	Cmpd **2**	179.0479	1.29	179.0486	4406	
**12x**	Ac-monoanion	297.0897	2.93	297.0894	447	
	Monoanion	255.0792	1.34, 2.63	255.0808	21621	0.19
	Cmpd **2**	179.0479	1.29	179.0486	4129	
**8i**	Monoanion	297.1261	2.96, 3.04	297.1270	29403	0.16
	Cmpd **2**	179.0479	1.29	179.0486	4623	
**9i**	Ac-monoanion	339.1367	3.35	339.1379	510	
	Monoanion	297.1261	2.96, 3.04	297.1270	58565	0.12
	Cmpd **2**	179.0479	1.29	179.0486	6861	
**8k**	Monoanion	339.1731	3.25	339.1710	57899	< 0.01
	Cmpd **2**	179.0479	1.29	179.0486	71	
**9k**	Ac-monoanion	381.1836	3.6	381.1816	3954	
	Monoanion	339.1731	3.25	339.1710	30321	< 0.01
	Cmpd **2**	179.0479	1.29	179.0486	32	
**11z**	Monoanion[3]	255.0792	1.36, 2.63	255.0803	2951	0.11
	Cmpd **2**	179.0479	1.29	179.0486	316	
**12z**	Ac-monoanion	297.0897	2.94	NA	0	
	Monoanion	255.0792	1.35, 2.62	255.0803	2558	0.14
	Cmpd **2**	179.0479	1.29	179.0486	364	

[1]M_{mi} = monoisotopic mass, t_R = retention time, m/z = mass to charge ratio, PI = integrated peak intensity.
[2]numbers represent the ratio of the compound **2** peak intensity to that of the corresponding monoanion (ma) intermediate.
[3]data for compounds **11z** and **12z** was collected at a different time than the other compounds and therefore the peak intensities may not be directly comparable.

Table S1. K562 uptake and metabolism data.[1]

Table S2. Plasma Metabolism data[1]

Cmpd		M_{mi}	t_R	m/z	PI	PI_2/PI_{ma}[2]
**8a**	Monoanion	255.0792	2.61	255.0808	18435	0.0021
	Cmpd **2**	179.0479	1.29	179.0486	68	
**9a**	Ac-monoanion	297.0897	2.95	297.0894	8914	
	Monoanion	255.0792	2.61	255.0808	8153	0.0058
	Cmpd **2**	179.0479	1.29	179.0486	48	
**11x**	Monoanion	255.0792	2.61	255.0808	12285	0.0032
	Cmpd **2**	179.0479	1.29	179.0486	40	
**12x**	Ac-monoanion	297.0897	2.93	297.0894	15857	
	Monoanion	255.0792	2.61	255.0808	132	0.1287
	Cmpd **2**	179.0479	1.29	179.0486	17	
**8i**	Monoanion	297.1261	3.04	297.1270	14136	0.0015
	Cmpd **2**	179.0479	1.29	179.0486	22	
**9i**	Ac-monoanion	339.1367	3.36	339.1379	6864	
	Monoanion	297.1261	3.03	297.1270	7959	0.0018
	Cmpd **2**	179.0479	1.29	179.0486	15	
**8k**	Monoanion	339.1731	3.26	339.1710	15312	0.0009
	Cmpd **2**	179.0479	1.29	179.0486	15	
**9k**	Ac-monoanion	381.1836	3.61	381.1816	744	
	Monoanion	339.1731	3.25	339.1710	998	0.0170
	Cmpd **2**	179.0479	1.29	179.0486	15	
**11z**	Monoanion[3]	255.0792	2.63	255.0803	1912	0
	Cmpd **2**	179.0479	1.29	N/A	0	
**12z**	Ac-monoanion	297.089	2.94	297.0858	4875	
	Monoanion	255.0792	2.62	255.0803	239	0
	Cmpd **2**	179.0479	1.29	N/A	0	

[1]M_{mi} = monoisotopic mass, t_R = retention time, m/z = mass to charge ratio, PI = integrated peak intensity.
[2]numbers represent the ratio of the compound **2** peak intensity to that of the corresponding monoanion (ma) intermediate.
[3]data for compounds **11z** and **12z** was collected at a different time than the other compounds and therefore the peak intensities may not be directly comparable.

Table S2. Plasma Metabolism data[1]
